# Comparative safety and effectiveness of perinatal antiretroviral therapies for HIV-infected women and their children: Systematic review and network meta-analysis including different study designs

**DOI:** 10.1371/journal.pone.0198447

**Published:** 2018-06-18

**Authors:** Areti Angeliki Veroniki, Jesmin Antony, Sharon E. Straus, Huda M. Ashoor, Yaron Finkelstein, Paul A. Khan, Marco Ghassemi, Erik Blondal, John D. Ivory, Brian Hutton, Kevin Gough, Brenda R. Hemmelgarn, Erin Lillie, Afshin Vafaei, Andrea C. Tricco

**Affiliations:** 1 Li Ka Shing Knowledge Institute, St. Michael’s Hospital, Toronto, Ontario, Canada; 2 Department of Medicine, University of Toronto, Toronto, Ontario, Canada; 3 The Hospital for Sick Children, Toronto, Ontario, Canada; 4 Department of Paediatrics, University of Toronto, Toronto, Ontario, Canada; 5 Department of Pharmacology and Toxicology, University of Toronto, Toronto, Ontario, Canada; 6 School of Epidemiology, Public Health and Preventive Medicine, Faculty of Medicine, University of Ottawa, Ottawa, Ontario, Canada; 7 Ottawa Hospital Research Institute, Center for Practice Changing Research, The Ottawa Hospital–General Campus, Ottawa, Ontario, Canada; 8 Departments of Medicine and Community Health Sciences, University of Calgary, Calgary, Alberta, Canada; 9 Epidemiology Division, Dalla Lana School of Public Health, University of Toronto, Toronto, Ontario, Canada; University of British Columbia, CANADA

## Abstract

**Background:**

Nearly all newly infected children acquire Human Immunodeficiency virus (HIV) via mother-to-child transmission (MTCT) during pregnancy, labour or breastfeeding from untreated HIV-positive mothers. Antiretroviral therapy (ART) is the standard care for pregnant women with HIV. However, evidence of ART effectiveness and harms in infants and children of HIV-positive pregnant women exposed to ART has been largely inconclusive. The aim of our systematic review and network meta-analysis (NMA) was to evaluate the comparative safety and effectiveness of ART drugs in children exposed to maternal HIV and ART (or no ART/placebo) across different study designs.

**Methods:**

We searched MEDLINE, EMBASE, and Cochrane Central Register of Controlled Trials (inception until December 7, 2015). Primary outcomes were any congenital malformations (CMs; safety), including overall major and minor CMs, and mother-to-child transmission (MTCT; effectiveness). Random-effects Bayesian pairwise meta-analyses and NMAs were conducted. After screening 6,468 citations and 1,373 full-text articles, 90 studies of various study designs and 90,563 patients were included.

**Results:**

The NMA on CMs (20 studies, 7,503 children, 16 drugs) found that none of the ART drugs examined here were associated with a significant increase in CMs. However, zidovudine administered with lamivudine and indinavir was associated with increased risk of preterm births, zidovudine administered with nevirapine was associated with increased risk of stillbirths, and lamivudine administered with stavudine and efavirenz was associated with increased risk of low birth weight. A NMA on MTCT (11 studies, 10,786 patients, 6 drugs) found that zidovudine administered once (odds ratio [OR] = 0.39, 95% credible interval [CrI]: 0.19–0.83) or twice (OR = 0.43, 95% CrI: 0.21–0.68) was associated with significantly reduced risk of MTCT.

**Conclusions:**

Our findings suggest that ART drugs are not associated with an increased risk of CMs, yet some may increase adverse birth events. Some ART drugs (e.g., zidovudine) effectively reduce MTCT.

## Introduction

Human immunodeficiency virus (HIV) infection is the second most common cause of death among young people worldwide [1]. In 2014, 2.6 million children aged 15 years and younger were living with HIV globally, with 220,000 children newly infected [1]. Nearly all newly infected children acquire HIV via mother-to-child transmission (MTCT) during pregnancy, labour or breastfeeding from untreated HIV-positive mothers [2]. The standard of care for pregnant women with HIV is antiretroviral therapy (ART) [2]. Treatment with highly-active ART (HAART), usually including three or more ART medications, can reduce MTCT to less than 2% [3]. There are six major ART drug classes: 1) nucleoside reverse transcriptase inhibitors (NRTIs), 2) non- NRTIs, 3) protease inhibitors, 4) integrase inhibitors, 5) fusion inhibitor, and 6) co-receptor inhibitors [4]. The effectiveness of these drugs in reducing MTCT is well-established. However, there is conflicting evidence about possible adverse effects in the neonate [3, 5, 6] (e.g., congenital malformations [7–10], and preterm delivery [6, 11]). Hence, for HIV-positive pregnant women, it is recommended that ART drugs be initiated as soon as possible in pregnancy [12–15]. Through a systematic review and network meta-analysis (NMA), we sought to evaluate the comparative safety and effectiveness of ART drugs in children who were exposed to ART (or no ART).

## Methods

The study protocol was registered with PROSPERO (CRD42014009071) and published in an open-access journal [16]. We revised our protocol by incorporating feedback from the research team and policy-makers from Health Canada, a department of the federal government, who posed the query for this study. Our methods are described briefly here; details can be found in the protocol publication [16] and S1 Appendix. Minor updates to the published protocol are described in S1 Appendix. Our NMA conforms to ISPOR guidance [17] and the PRISMA-NMA extension (S2 Appendix) [18].

### Eligibility criteria

We included studies involving children of mothers who were HIV positive during pregnancy and were administered any of 24 ART medications within 6 drug classes approved for use in Canada (S3 Appendix). The comparators were placebo, no ART treatment, or any other of the 24 ART medications alone or in combination. Randomized controlled trials (RCTs), quasi-RCTs, non-RCTs, interrupted-time-series, controlled before-after studies, and observational studies (cohort, case-control) were eligible for inclusion. We used companion reports of included studies for supplementary information.

The primary safety outcome was any congenital malformation (CM), including overall major and minor CMs, and the primary effectiveness outcome was MTCT of HIV. Secondary safety outcomes were infant/child death, preterm births, stillbirths, and low birth weight (see S4 Appendix for outcome definitions and age of children in each). No limitations were imposed on publication status, language of dissemination, study setting, duration of study follow-up or period of study conduct.

### Search strategy and selection criteria

The literature search was developed by an experienced librarian (Dr. Perrier) and peer-reviewed by another librarian (Ms. Skidmore)[19]. We searched electronic databases (MEDLINE, EMBASE, and the Cochrane Central Register of Controlled Trials) from inception until December 7, 2015 (S5 Appendix) [19]. We also searched the grey literature, including trial protocol registries, dissertation databases, and conference proceedings [20]. We scanned the reference lists of the included studies and relevant reviews to identify further potentially relevant studies. All outcome data are available via the Open Science Framework repository [21].

Citations captured in the search were screened after two calibration exercises resulted in 72% agreement among reviewers. Before commencing full-text screening, another two calibration exercises were completed resulting in 65% agreement. All titles/abstracts and full-texts were screened by pairs of reviewers working independently. Discrepancies were resolved through discussion. The same approach was used to abstract data and appraise study quality. When deemed necessary, we contacted authors to obtain additional information (S6 Appendix).

### Quality appraisal

We used the Cochrane Effective Practice and Organisation of Care (EPOC) risk-of-bias tool for experimental and quasi-experimental studies [22] and the Newcastle-Ottawa Scale study for observational studies [23]. For each outcome including ≥10 studies, we assessed small-study effects and publication bias using the comparison-adjusted funnel plot under the fixed-effect meta-analysis model [24]. Prior to drawing a funnel plot, we ordered all ART drugs from oldest to newest according to Health Canada/Federal Drug Administration approval dates. To overcome part of the correlations due to multi-arm trials, we plotted only the data points corresponding to the basic parameters of each study (treatment comparisons with a common comparator). The common comparator was placebo/no treatment or the oldest treatment comparator.

### Statistical analysis

Random-effects meta-analysis and NMA models were applied for each outcome using the odds ratio (OR) to account for the anticipated methodological and clinical between-study heterogeneity [25]. Outcome data were pooled using Bayesian hierarchical models, and the Markov Chain Monte Carlo algorithm.

We categorised network nodes considering specific drugs and drug categories. Our team composed of clinicians, epidemiologists, pharmacists, and statisticians pre-specified the network treatment nodes. When an ART medication was not specified in a category (e.g., HAART when three or more specific drugs were included in a single arm, ART duo-therapy when two drugs were included in a single arm, and ART mono-therapy when one drug was included in a single arm), this was included in meta-analysis only. Many of the included studies failed to report dosages, and hence we were unable to account for differences in drug dosages. When multiple dosages for the same treatment were reported in an eligible study, we combined the data for this treatment into a single group by summing sample sizes and numbers of people with events across doses [26].

Prior to preforming NMA, the transitivity and consistency assumptions were assessed [27–31]. We assessed transitivity for both specific drugs and drug categories using maternal age, baseline risk, low and middle-income countries (LMIC), percentage of patients with antenatal care, use of illicit drugs, and CD4+ count as potential treatment effect modifiers. We present the mean of each continuous potential effect modifier and the mode of each categorical potential effect modifier for each pairwise comparison and outcome in tables [27]. It should be noted that differences in the included number of studies between the specific drug and drug category NMAs of the same outcome may happen due to inconsistent reporting of the individual studies. For each outcome, the network consistency was evaluated using the random-effects design-by-treatment interaction model [28, 29]. If the global test suggested inconsistency, we assessed local consistency in specific network paths using the loop-specific method [30, 31]. In both pairwise meta-analysis and NMA, we assumed common within-network between-study variance (τ^2^) across treatment comparisons, whereas in the loop-specific approach each closed loop was assessed assuming common within-loop τ^2^. This approach helped the estimation of τ^2^ in treatment comparisons including a single study, and it was clinically reasonable, since the treatments were all pharmacological medications.

When statistically significant inconsistency was detected, we checked for data errors and if none were identified, network meta-regression and sensitivity analysis were conducted. We explored important heterogeneity and/or inconsistency considering the treatment effect modifiers described above. In particular, for the primary outcomes, we performed network meta-regression for age and baseline risk (i.e., using placebo/no ART as a control group), assuming a common fixed coefficient across treatment comparisons. Sensitivity analyses were conducted on the primary outcomes restricting to studies with: >50% of patients that received antenatal care, patients from LMIC, large study size (i.e., >300 patients), patients with a history of illicit drug use, patients with a history of smoking, patients with a history of alcohol use, patients with tuberculosis co-infection, patients with low CD4+ count (<200), and studies which had low risk of bias (according to the adequacy of follow-up and comparability components for observational studies and randomisation and incomplete outcome data components for RCTs). Finally, for the primary outcomes, we applied the model suggested by Schmitz et al. [32] to combine randomised data with non-randomised data. In this model, we did not use a bias adjustment to account for over-precision or for over-/under-estimation, as we were uncertain about the magnitude of bias introduced from the observational studies.

We present the summary treatment effect estimates with a 95% credible interval (CrI) for each pair of treatments. For the NMA estimates, we also present a 95% predictive interval, which captures the magnitude of τ^2^ and presents the interval within which we would expect the treatment effect of a future study to lie [33, 34]. Under the consistency assumption, the safety and effectiveness of ART medications were ranked using the surface under the cumulative ranking (SUCRA) curve [35], and SUCRAs were depicted in a rank-heat plot [36]. We measured goodness-of-fit using the posterior mean of the residual deviance, the deviance information criterion, and the magnitude of the between-study variance [37, 38].

Meta-analyses and NMAs were performed in OpenBUGS [39], assuming non-informative priors for all model parameters and a half-normal prior distribution for the between-study standard deviation (τ~*N*(0,1), τ>0). The models were run for at least 100,000 iterations to ensure model convergence, which was checked by visual inspection of the mixing of two chains, after discarding the first 10,000 iterations and thinning of 10. The design-by-treatment interaction model was applied in Stata using the *network* command [40].

## Results

### Literature search

**Fig 1:**
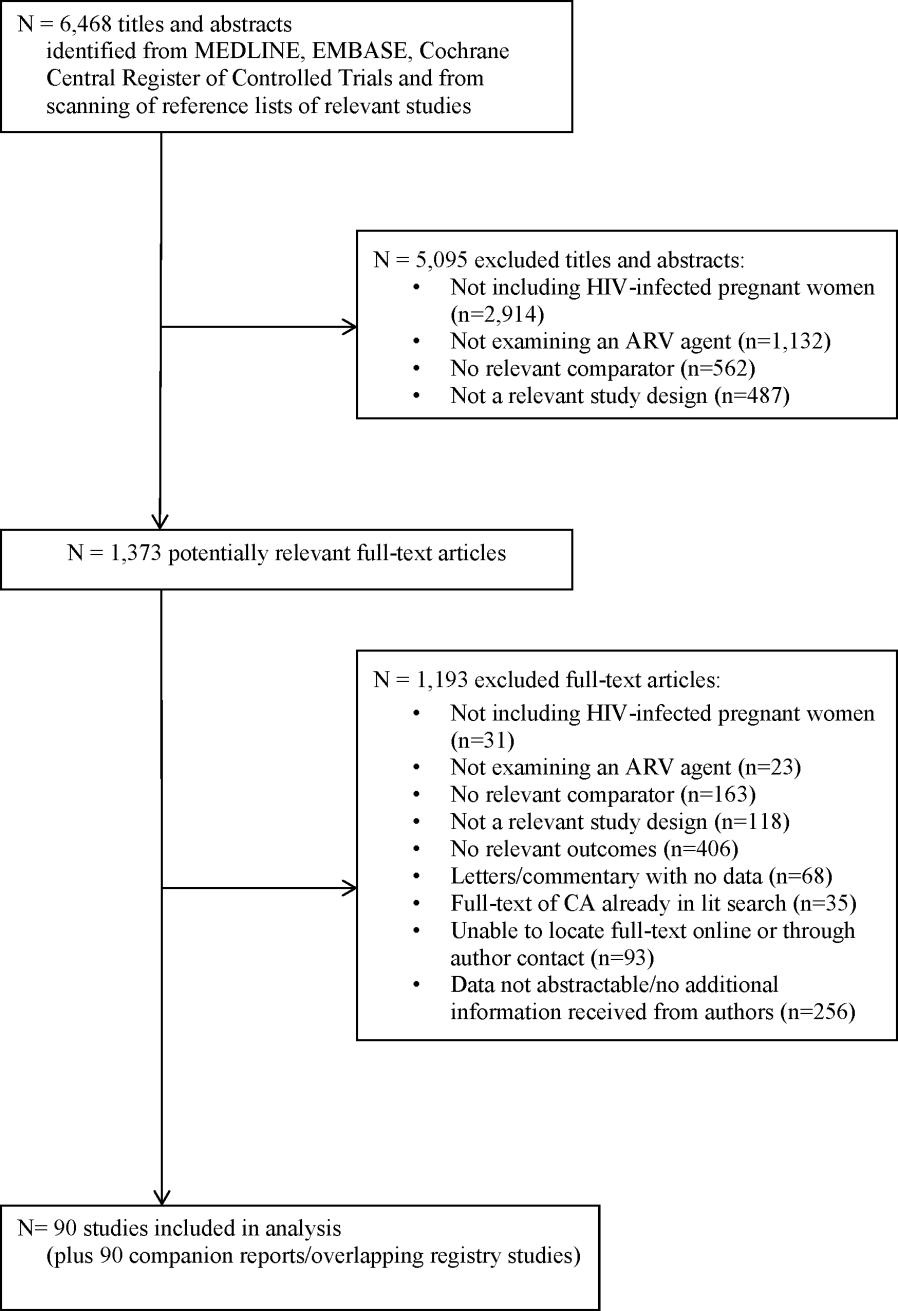
Preferred reporting items for systematic review and meta-analyses study flow: Literature search and study selection.

After screening 6,468 citations and 1,373 full-text articles, 90 studies were included, and 90 companion reports provided supplementary information (Fig 1). Two studies and two companion reports were conference abstracts [41–44]. One companion report was published in German [45]. Full references for the included studies are available in S7 Appendix.

### Study, patient, and intervention characteristics

Studies were conducted from 1995 until 2015, most commonly in Africa (32%), followed by Europe (22%) and North America (21%; Table 1, S8 Appendix). Most study designs were cohort (80%), followed by RCTs (19%) and case control (1%). Study size ranged from 8 to 13,537 participants (Table 1, S9 Appendix). Mean maternal age ranged from 23 to 34 years. The HIV stages varied among women, as did studies including women with various risk factors and comorbidities (S10 Appendix). Zidovudine was the most frequently studied drug (42.1%) followed by Lamivudine (21.9%; Table 1, S10 Appendix).

**Table 1:** Study, patient, and interventions characteristics.

CHARACTERISTICS		Count (%)
Total # patients: 90,563 Mean number of patients (range):1,006 (8–13,537) Mean maternal age in years (range): 27 (23.3–33.6)*		
**STUDY CHARACTERISTICS**		
**Year of Publication **		**90 (100%)**†[46, 47]
		2010–2015	36 (40%)
		2005–2009	25 (28%)
		2000–2004	18 (20%)
		1995–1999	11 (12%)
**Geographic Region**		**90 (100%)**†[46, 47]
		Africa	29 (32%)
		Europe	20 (22%)
		Northern America	19 (21%)
		Asia	9 (10%)
		Southern America	6 (7%)
		Multi-continental	5 (6%)
		Central America and Caribbean	2 (2%)
**Study Design**		**90 (100%)**†[46, 47]
		RCT	17 (19%)
		Cohort study (including registries)	72 (80%)
		Case control study	1 (1%)
**Study Conduct Site**		**90 (100%)**†[46, 47]
		Multicenter	47 (52%)
		Single-center	43 (48%)
**PATIENT CHARACTERISTICS**		
**Sample Size**		**90(100%)**†[46, 47]
		0–99	22 (24%)
		100–299	25 (28%)
		300–499	13 (15%)
		500–999	14 (16%)
		1000–4999	11 (12%)
		5000–9999	4 (4%)
		>10000	1 (1%)
**Mean Maternal Age**		**90 (100%)**†[46, 47]
		23–25 years	9 (10%)
		26–28 years	25 (28%)
		29–31 years	16 (18%)
		32–34 years	2 (2%)
		Not Reported	38 (42%)
**Risk factors/Effect Modifiers/Comorbidities Reported**		**183 (100%)**ǂ
		Patients with antenatal care	59 (32.2%)
		Patients with low CD4+ count (<200)	41 (22.4%)
		Use of illicit drugs	28 (15.3%)
		Low to middle income economy country (LMIC)	24 (13.1%)
		Use of Tobacco	14 (7.7%)
		Use of alcohol	11 (6.0%)
		Tuberculosis co-infection	6 (3.3%)
**Outcomes Examined: Frequency** **ǂ**		**224 (100%)ǂ**
		Pre-term delivery	63 (28%)
		Stillbirth	43 (19%)
		Low birth weight, small for gestational age	52 (23%)
		Congenital malformations	28 (13%)
		Infant/child death	19 (8%)
		Mother-to-child transmission of HIV	17 (8%)
		Small head and short length	2 (1%)
**INTERVENTION CHARACTERISTICS**		
**Specific Drugs [Brand name]**		**Outcomes**	**447 (100%)ǂ**^**§**^
***Nukes (Nucleoside Reverse Transcriptase Inhibitors [NRTIs])***		
AZT (ZDV)^‖^ [Retrovir]		CM, MTCT, Infant/Child death, Preterm, Stillbirths, LBW	188 (42.1%)
3TC [Epivir]		CM, Infant/Child death, Preterm, Stillbirths, LBW	98 (21.9%)
d4T [Zerit]		CM, Preterm, Stillbirths, LBW	23 (5.1%)
ddl [Videx EC (enteric coated)]		CM, Preterm, Stillbirths	9 (2.0%)
ABC [Ziagen]		CM, Infant/Child death, Preterm, Stillbirths, LBW	8 (1.8%)
TDF [Viread]		Infant/Child death, Preterm, Stillbirths, LBW	6 (1.3%)
***Non-Nukes [NNRTIs]***		
NVP [Viramune]	CM, MTCT, Infant/Child death, Preterm, Stillbirths, LBW	35 (7.8%)
EFV [Sustiva]	CM, Preterm, Stillbirths, LBW	7 (1.6%)
***Protease Inhibitors***		
LPV (LOP)^‖^ [Kaletra]	CM, Infant/Child death, Preterm, Stillbirths, LBW	25 (5.6%)
r (RIT)^‖^ [Norvir]	CM, Infant/Child death, Preterm, Stillbirths, LBW	25 (5.6%)
NFV (NLF)^‖^ [Viracept]	CM, Preterm, Stillbirths, LBW	14 (3.1%)
IND [Crixivian]	Preterm, Stillbirths, LBW	7 (1.6%)
SAQ [Invirase]	Stillbirths	2 (0.5%)

Notes

*Mean age is average of reported medians and means for 52 studies.

^†^Includes unpublished studies.

ǂ Multiple interventions and outcomes reported per study.

^**§**^Specific drug count includes drugs administered in combination or as monotherapy for treatment interventions at pregnancy.

^‖^Abbreviations in brackets are used in this review.

Abbreviations: ART, Antiretroviral Therapy; CM, Congenital Malformation; HAART, Highly Active Anti-Retroviral Therapy, MTCT, Mother to Child Transmission of HIV; NA, Not Applicable; RCT, Randomized Control Trial Specific Drug abbreviations: ABC, Abacavir; ddI, Didanosine; IND, Indinavir; 3TC, Lamivudine; LPV (LOP), Lopinavir; NVP, Nevirapine; NFV (NLF) Nelfinavir; SAQ, Saquinavir, d4T,Stavudine; EFV, Sustiva; r (RIT), Ritonavir; AZT (ZDV), Zidovudine.

### Quality appraisal

**Fig 2:**
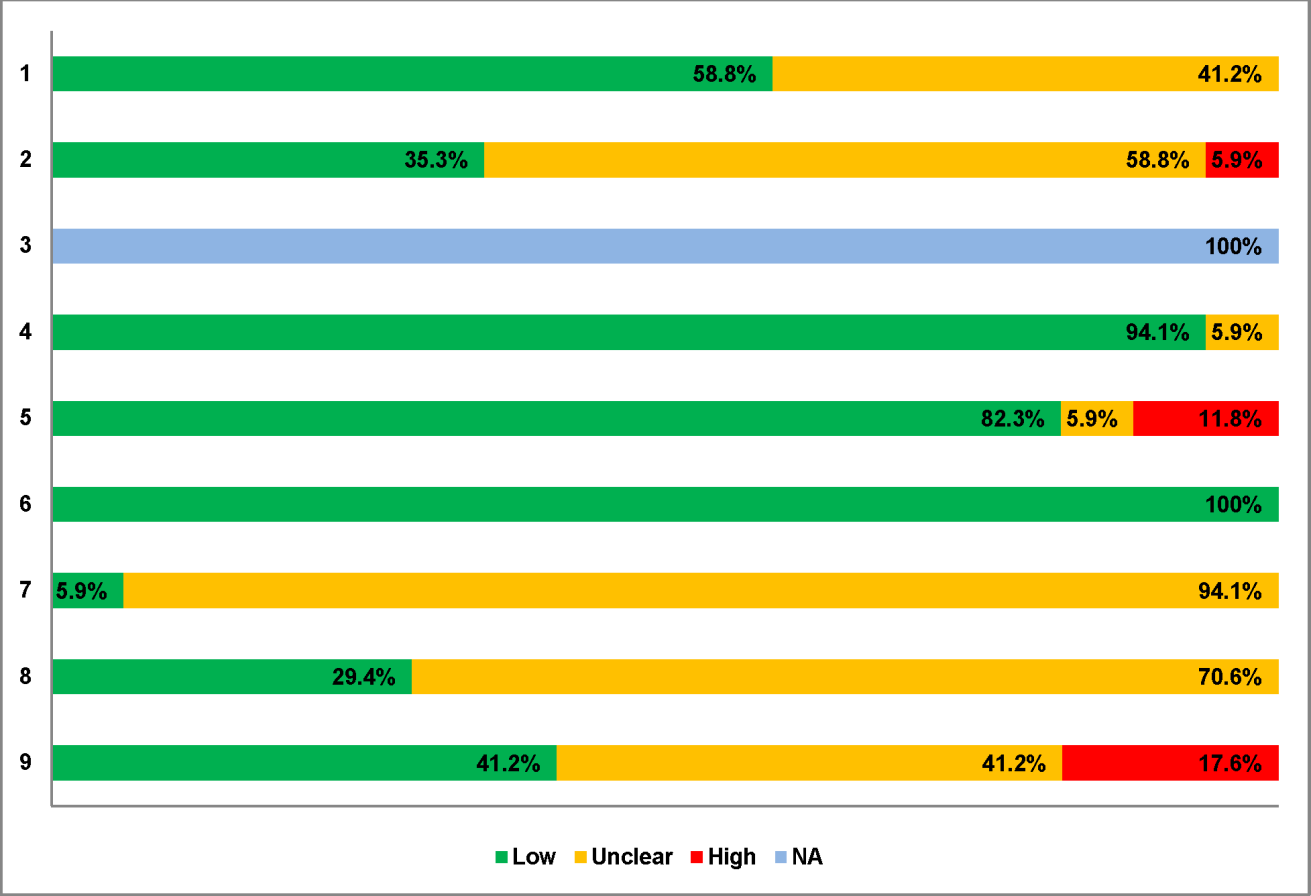
Aggregate EPOC risk-of-bias appraisal results. Abbreviations: EPOC, Effective Practice and Organization of Care; High, High risk of bias; Low, Low risk of bias; Unclear, Unclear risk of bias; NA, Not Applicable. Items: 1. Random sequence generation. 2. Allocation concealment. 3. Similar baseline outcome measures. 4. Similar baseline characteristics. 5. Incomplete outcome data. 6. Blinding. 7. Contamination. 8. Selective outcome reporting. 9. Other bias.

Across 17 RCTs, 41% had an unclear risk-of-bias for random sequence generation and 65% scored an unclear/high risk-of-bias due to a lack of allocation concealment (S11 Appendix, Fig 2). Regarding contamination between treatment groups, 94% were at an unclear risk-of-bias, while 71% were at an unclear risk-of-bias due to selective outcome reporting. For other risk-of-bias criteria, including methodological issues related to study design, 59% were assessed as having an unclear/high risk-of-bias.

**Fig 3:**
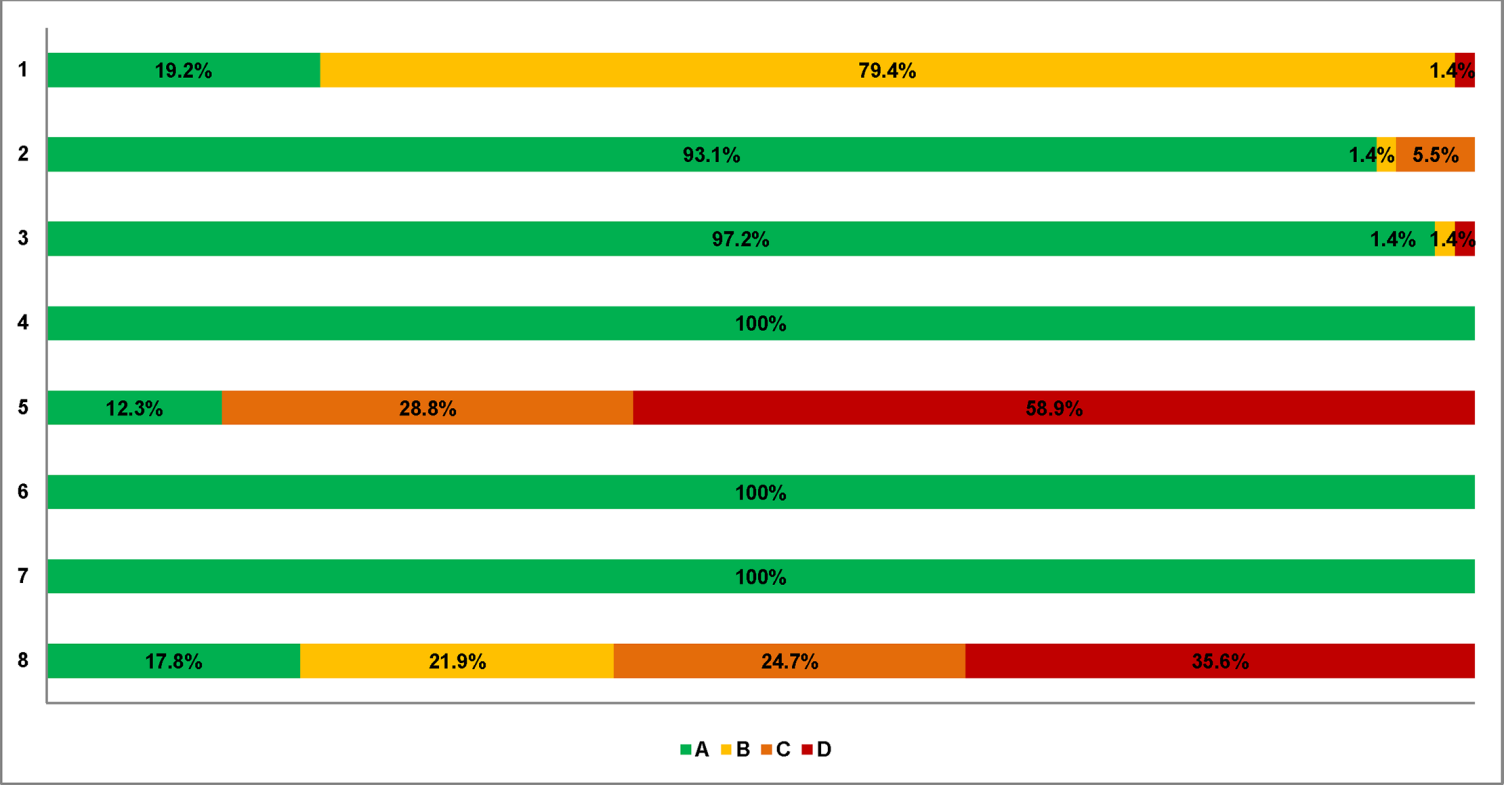
Aggregate Newcastle-Ottawa Scale results. Items: 1. Representativeness of the exposed cohort: A, Truly representative of the average HIV-infected pregnant woman taking ART in the community; B, somewhat representative of the average HIV-infected pregnant woman taking ART in the community; D, no description of the derivation of the cohort. 2. Selection of the non-exposed cohort: A, drawn from the same community as the exposed cohort; B, drawn from a different source; C, no description of the derivation of the non-exposed cohort. 3. Ascertainment of exposure: A, Secure record (e.g., medical records, surgical records); B, Structured interview; D, No description. 4. Demonstration that outcome of interest was not present at start of study: A, Yes; D, No description. 5. Comparability of cohorts on the basis of the design or analysis: A, study controls for age and one other important factor; C, study controls for any other important factor; D, study does not control for any important factor or it is not described. 6. Assessment of outcome: A, Independent OR blind assessment; D, No description. 7. Was follow-up period long enough for outcomes to occur: A, yes; D, No description. 8. Adequacy of follow-up of cohorts: A, complete follow-up—all subjects accounted for; B, subjects lost to follow-up unlikely to introduce bias—small number lost, or description provided of those lost; C, follow-up rate is inadequate and no description of those lost; D, no statement.

**Fig 4:**
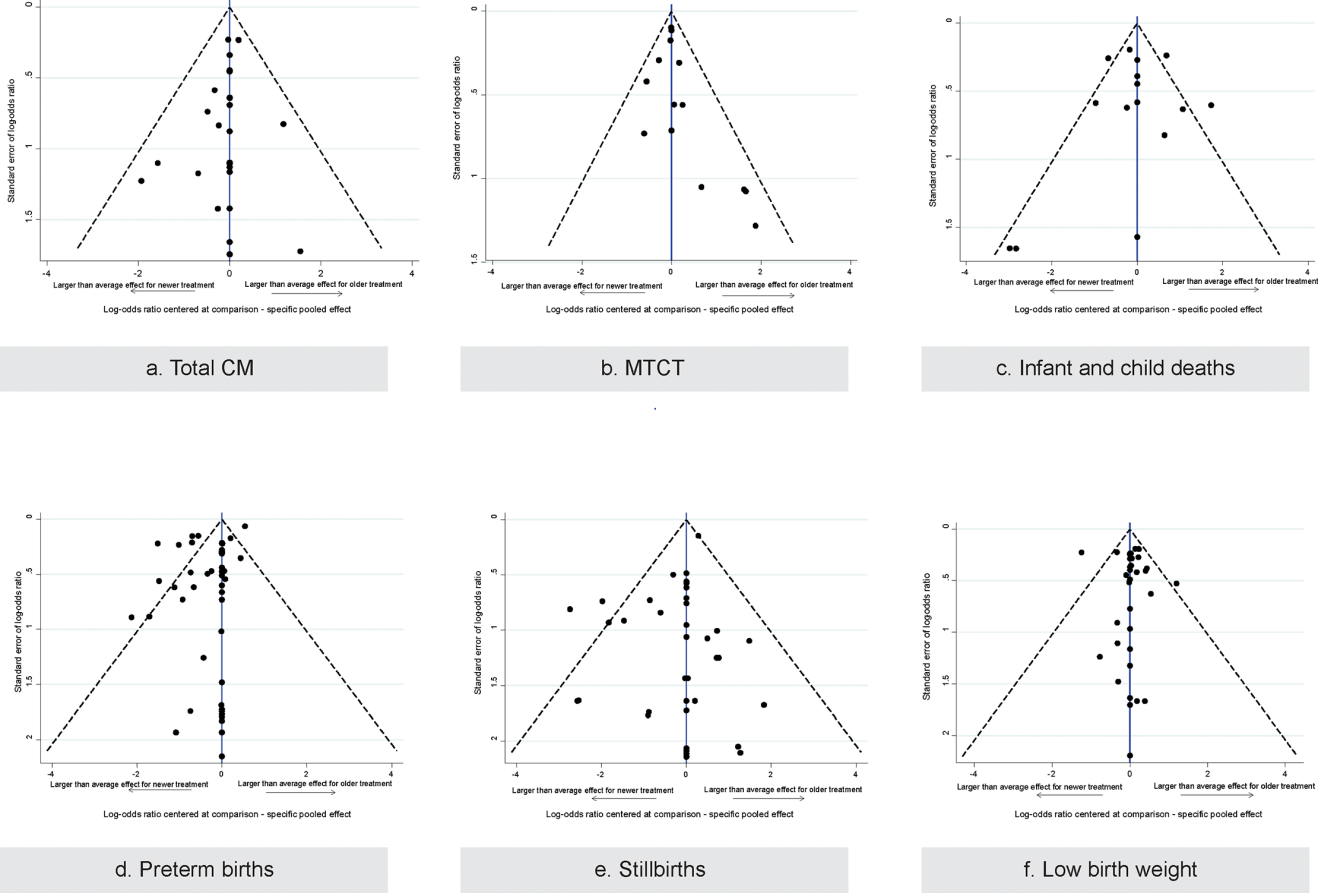
Funnel plots including specific antiretroviral drugs by outcomes reported. Funnel Plots including Antiretroviral Specific Drugs by Outcomes reported. Legend: A) Total Congenital Malformations—20 studies (9 RCTs, 11 Cohorts), # 7503 patients, # 16 treatments B) Mother-to-Child Transmission of HIV–# 11 studies (3 RCTs, 8 Cohorts), # 10786 patients, # 6 treatments C) Infant and child deaths–#13 studies (8 RCTs, 5 Cohorts), # 11385 patients, # 8 treatments D) Preterm births– 35 studies (8 RCTs, 1 Case-control, 26 Cohorts), # 20576 patients, # 17 treatments E) Stillbirths –26 studies (13 RCTs, 13 Cohorts), # 17507 patients, # 20 treatments, F) Low birth weight– 30 studies (10 RCTs, 19 Cohorts, 1 Case-control), # 21848 patients, # 16 treatments.

Across the 72 cohort studies and the single case-control study, 59% did not control for any confounding variables and 36% did not report the number of patients lost to follow-up (S12 Appendix, Fig 3). Comparison-adjusted funnel plots were symmetrical, except for MTCT, infant and child deaths, and preterm births outcomes (S13 Appendix, Fig 4).

### Data synthesis

**Fig 5:**
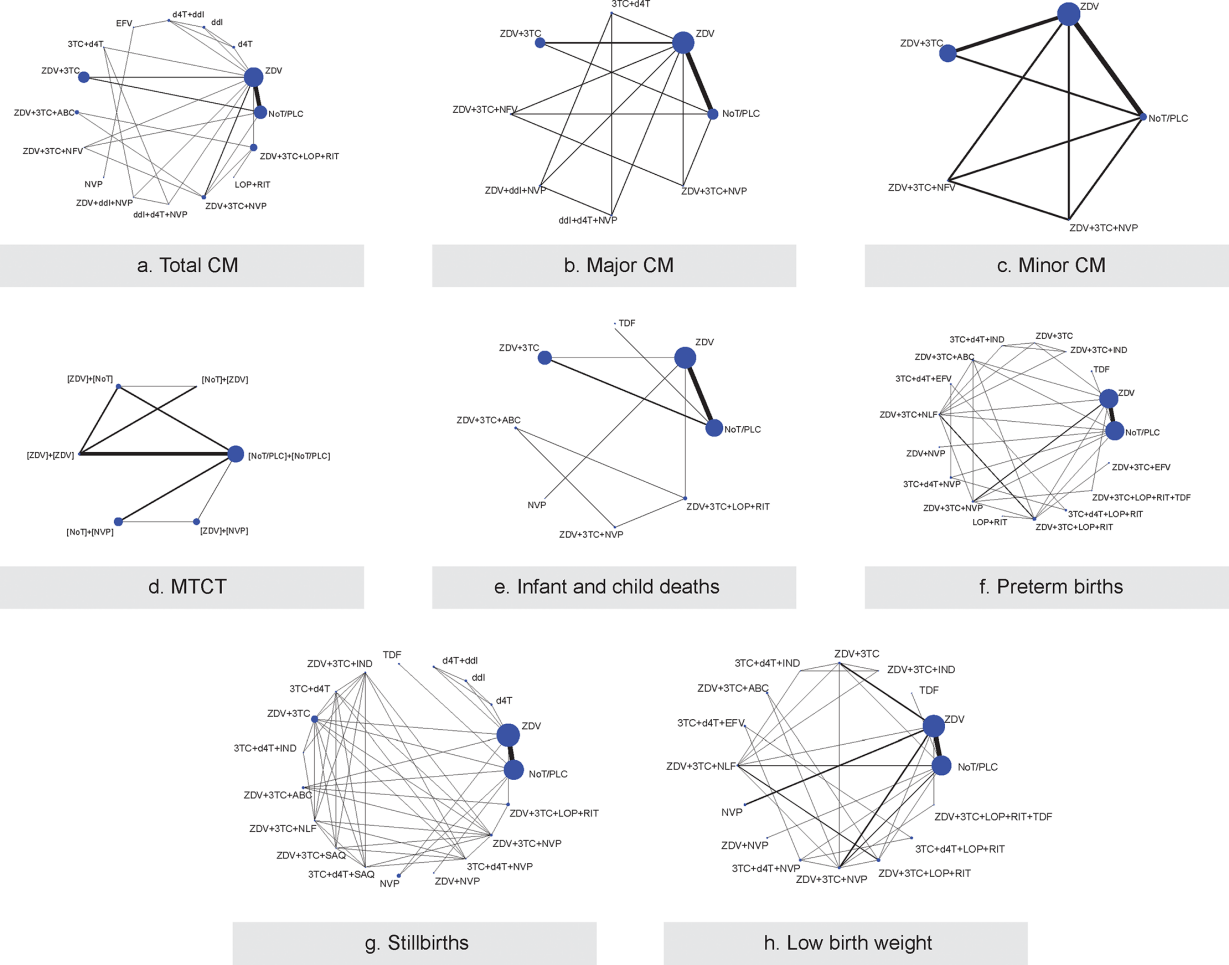
Network diagrams of antiretroviral medications for each outcome. Legend: A) Total Congenital Malformation B) Major Congenital Malformation C) Minor Congenital Malformation D) Mother to Child Transmission E) Infant and child deaths F) Preterm births G) Stillbirths H) Low birth weight. Each node represents an antiretroviral medication and each line represents a direct comparison between medications. The nodes are weighted according to the number of patients in each medication, and the lines are weighted according to the number of studies included in the direct comparison. Abbreviations: ABC, Abacavir; ddI, Didanosine; CM, Congenital Malformations; IND, Indinavir; 3TC, Lamivudine; LOP, Lopinavir; MTCT, Mother to Child Transmission; NVP, Nevirapine; NLF, Nelfinavir; NoT, No Treatment; PLC, Placebo; SAQ, Saquinavir; d4T, Stavudine; EFV, Sustiva; RIT, Ritonavir; ZDV, Zidovudine.

Two NMAs were conducted for each outcome, first by specific drugs and then by drug categories. Network plots for specific drugs are depicted in Fig 5, whereas ART drug category network plots are depicted in S14 Appendix. Across all outcomes, transitivity assessment suggested discernible variation across treatment comparisons regarding CD4+ count, illicit drug use, baseline risk, and LMIC (S15 Appendix). Unless otherwise noted, a design-by-treatment interaction model suggested consistency in the networks (S16 and S17 Appendices). Alternatively, a loop-specific approach pointed out loops potentially responsible for inconsistency (S18 Appendix).

**Fig 6:**
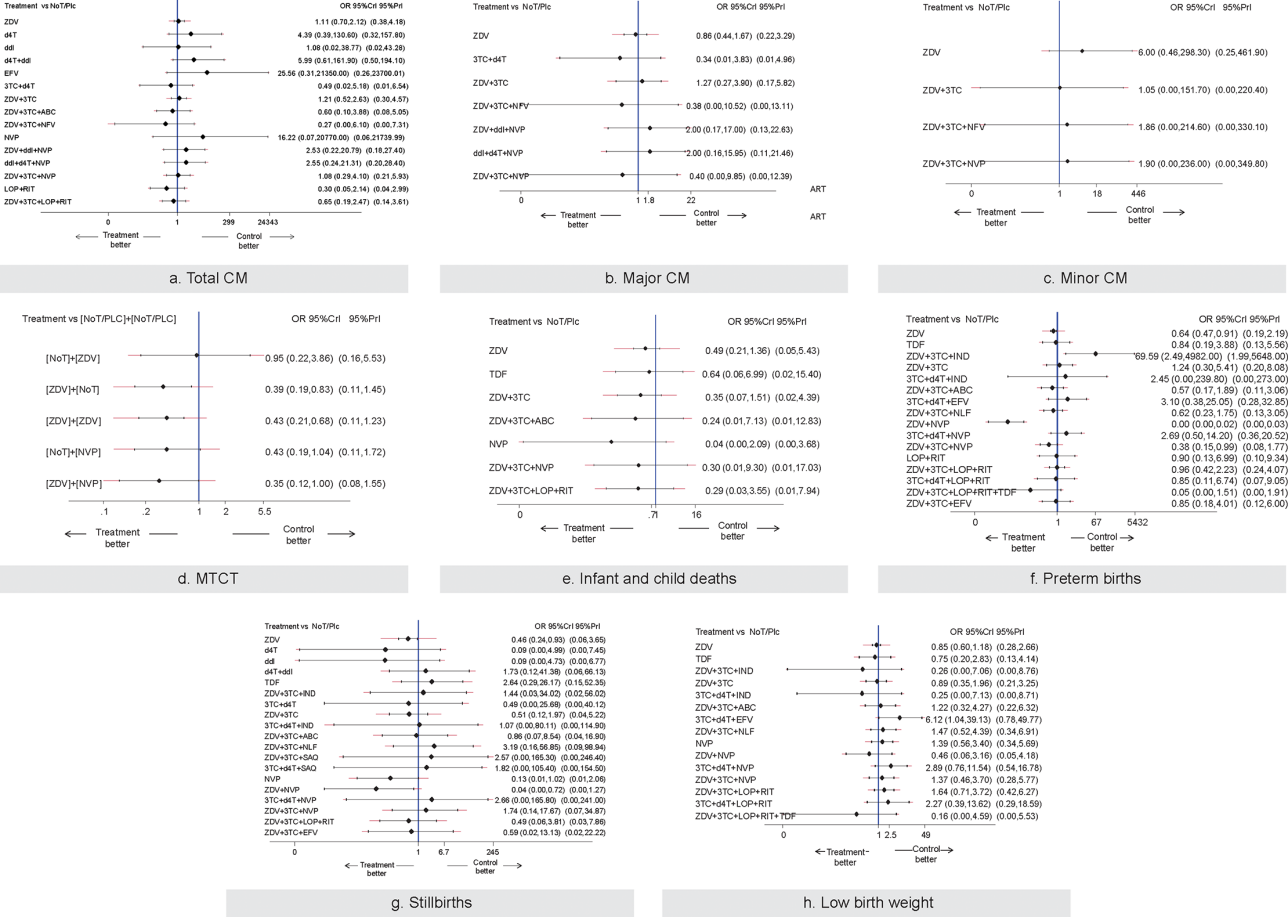
Forest plots for each outcome separately using specific antiretroviral therapy drugs. Legend: (τ^2^ = Between-Study Variance) A) Total Congenital Malformation (τ^2^ = 0.15, 95% CrI 0.00–1.14) B) Major Congenital Malformations (τ^2^ = 0.13, 95% CrI 0.00–1.66) C) Minor Congenital Malformations (τ^2^ = 0.40, 95% CrI 0.00–4.48) D) Mother to Child Transmission (τ^2^ = 0.12, 95% CrI 0.00–1.22) E) Infant and child deaths (τ^2^ = 0.83, 95% CrI 0.18–3.41) F) Preterm births(τ^2^ = 0.31, 95% CrI 0.15–0.73) G) Stillbirths (τ^2^ = 0.77, 95% CrI 0.19–2.32) H) Low birth weight. (τ^2^ = 0.25, 95% CrI 0.08–0.75). The black horizontal lines represent the credible intervals for the summary odds ratios for each specific drug comparison and the red horizontal lines represent the predictive intervals. The blue vertical line is the line of no effect. Abbreviations: ABC, Abacavir; CrI, Credible Intervals; ddI, Didanosine; IND, Indinavir; 3TC, Lamivudine; LOP, Lopinavir; NVP, Nevirapine; NLF, Nelfinavir; NoT, No Treatment; Plc, Placebo; PrI, Predictive Interval; SAQ, Saquinavir; d4T, Stavudine; EFV, Sustiva; RIT, Ritonavir; ZDV, Zidovudine.

**Fig 7:**
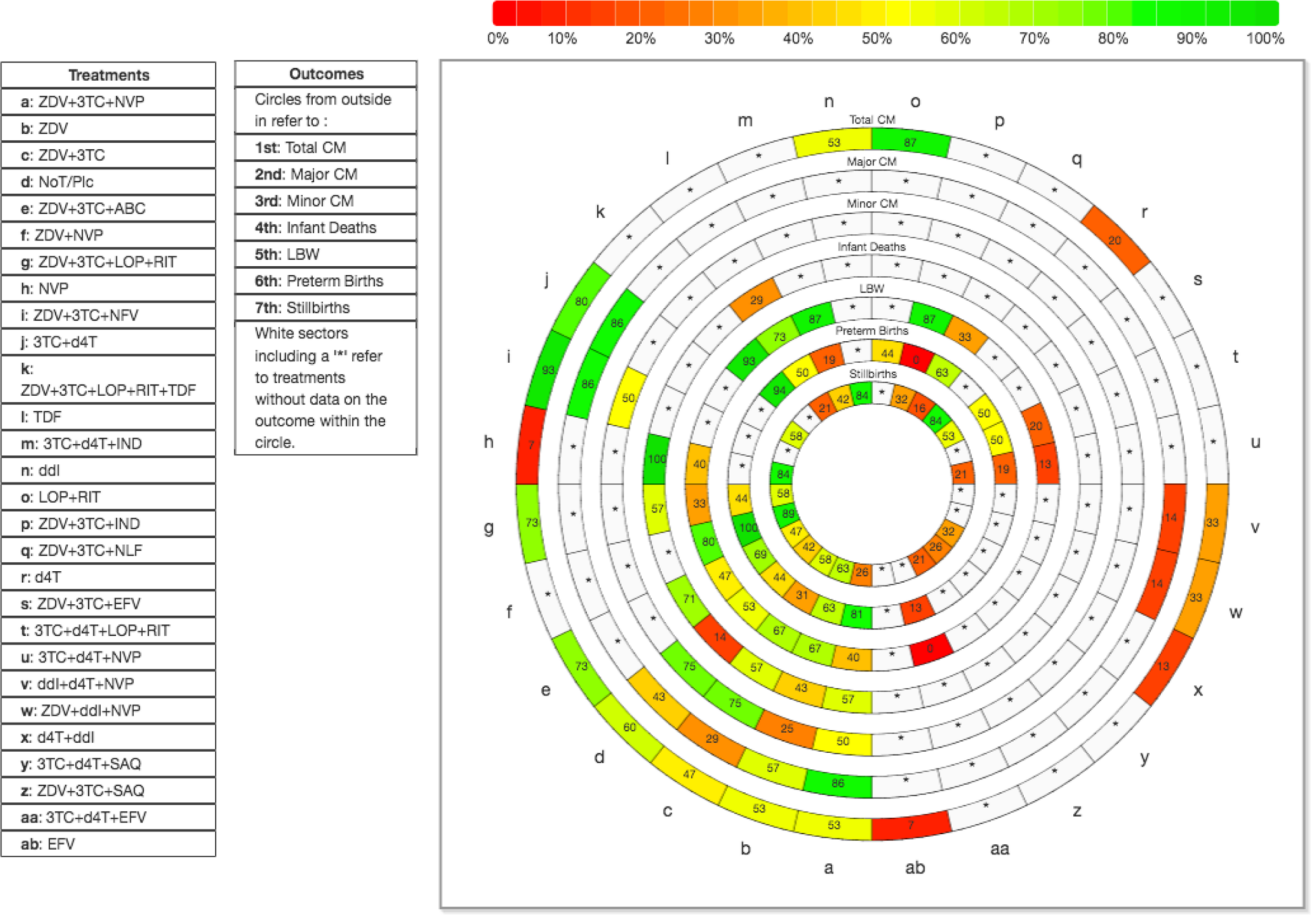
Rank-heat plot of 28 antiretroviral treatments (28 radii) and seven safety outcomes (seven concentric circles). Each sector is colored according to the SUCRA value of the corresponding treatment and outcome using the transformation of three colors red (0%), yellow (50%), and green (100%). Abbreviations: ABC, Abacavir; CM, Congenital Malformations; ddI, Didanosine; IND, Indinavir; 3TC, Lamivudine; LOP, Lopinavir; LBW, Low Birth Weight; NVP, Nevirapine; NLF, Nelfinavir; SAQ, Saquinavir; d4T, Stavudine; SUCRA, surface under the cumulative ranking curve; EFV, Sustiva; RIT, Ritonavir; ZDV, Zidovudine.

In the following sections, we present the consistent statistically significant NMA results by outcome for specific drugs compared with control (no ART/placebo) (Fig 6), while the drug categories NMA results are presented in S17 and S19 Appendices. Further details regarding: treatment group risk for each outcome, subgroup analyses, meta-regression analyses, sensitivity analyses, Schmitz model analyses, SUCRAs, and inconsistent NMA results are available in S20, S21 and S22 Appendices. A rank-heat plot based on SUCRA values is presented in Fig 7.

#### Congenital malformations: Total CM

NMA including 16 specific drugs, 20 studies (9 RCTs, 11 cohort studies), and 7,503 children (*τ*^2^ = 0.15, 95% CrI 0.00–1.14) suggested that none of the specific ART drugs was associated with a significant increased risk of CM across the 120 ART drug comparisons. Multiple meta-regression, subgroup, and sensitivity analyses were conducted for specific ART drugs and the direction of results was consistent with our NMA (S21 Appendix).

NMA was conducted including 4 drug categories, 17 studies (8 RCTs, 9 cohort studies), and 7,833 children (τ^2^ = 0.33, 95% CrI 0.01–1.35). Across the six ART category comparisons, none of the ART categories was associated with significant increased risk of CM, but the design-by-treatment interaction model suggested significant inconsistency. Multiple meta-regression, subgroup, and sensitivity analyses were conducted for ART categories, where evidence of inconsistency was found across all meta-regression analyses and sensitivity analysis for adequacy of follow-up and incomplete outcome data for RCTs. The additional analysis results were not importantly different from the NMA results (S22 Appendix).

#### Congenital malformations: Major CM

NMA including 8 specific drugs, 9 studies (3 RCTs, 6 cohort studies), and 2,808 children (*τ*^2^ = 0.13, 95% CrI 0.00–1.66) suggested that none of the specific ART drugs significantly increased the risk of major CM across the 28 ART drug comparisons. Meta-regression, subgroup, and sensitivity analyses’ results did not change from NMA results when specific ART drugs were considered (S21 Appendix).

The NMA including 4 drug categories, 9 studies (3 RCTs, 6 cohort studies), and 3,475 children (τ^2^ = 0.06, 95% CrI 0.00–0.87), showed that no drug category significantly increased the risk of major CM. The meta-regression, subgroup, and sensitivity analyses’ results did not change from the NMA results when ART categories were considered (S22 Appendix).

#### Congenital malformations: Minor CM

Only 2 cohort studies including 69 children and 5 specific drugs were available for minor CMs (τ^2^ = 0.40, 95% CrI 0.00–4.48). The same 2 studies informed 4 drug categories with 87 children (τ^2^ = 0.60, 95% CrI 0.00–5.01). None of the 10 NMA specific drug comparisons and 6 NMA ART category comparisons reached statistical significance (S21 and S22 Appendices).

#### Mother-to-child transmission of HIV

NMA including 6 specific drugs, 11 studies (3 RCTs, 8 cohort studies), and 10,786 children (*τ*^2^ = 0.12, 95% CrI 0.00–1.22) suggested that zidovudine administered once (OR = 0.39, 95% CrI: 0.19–0.83) and twice (OR = 0.43, 95% CrI 0.21–0.68) significantly reduced the risk of MTCT. The funnel plot suggested that the results for specific ART drugs might be influenced by publication bias and small-study effects, which may importantly impact the estimated treatment effects (Fig 4). This was explored through the Schmitz model, meta-regression, subgroup, and sensitivity analyses, which suggested no significant results, or that only zidovudine administered twice significantly reduced the risk of MTCT, or that no treatment plus nevirapine, zidovudine administered once, and zidovudine+nevirapine significantly reduced the risk of MTCT (S21 Appendix).

None of the specific ART drugs were associated with reduced risk of MTCT after a) restricting to large studies (4 cohort studies, 9,731 children, 5 drugs, *τ*^2^ = 0.16, 95% CrI 0.00–3.15), b) restricting to studies with antenatal care (3 RCTs, 3 cohort studies, 4,248 children, 4 drugs, *τ*^2^ = 0.14, 95% CrI 0.00–1.93), and c) adjusting for baseline risk of MTCT (estimated regression coefficient on OR scale: 1.16, 95% CrI 0.27–2.25; *τ*^2^ = 0.15, 95% CrI 0.00–1.52) (S21 Appendix).

The four sensitivity analyses restricting to: a) CD4+ count studies (1 RCT, 5 cohort studies, 5,457 children, 5 specific drugs, *τ*^2^ = 0.32, 95% CrI 0.00–3.36), b) studies with a low risk of randomisation generation bias and adjusting for confounding variables (2 RCTs, 6 cohort studies, 10,300 children, 6 drugs, *τ*^2^ = 0.14, 95% CrI 0.00–1.89), c) studies with a low risk of bias for incomplete outcome data and adequacy of follow-up (3 RCTs, 4 cohort studies, 1,214 children, 4 drugs, *τ*^2^ = 0.52, 95% CrI 0.00–3.33), and d) studies including a small proportion of women who used illicit drugs (13–20%; 1 RCT, 4 cohort studies 3,892 patients, 4 drugs), suggested that only zidovudine administered twice significantly reduced risk of MTCT. Applying the Schmitz model, the combination of no treatment and nevirapine (OR = 0.43, 95% CrI: 0.20–0.94), zidovudine administered once (OR = 0.35, 95% CrI: 0.16–0.67), and zidovudine plus nevirapine (OR = 0.35, 95% CrI: 0.14–0.89) significantly reduced risk of MTCT (*τ*^2^ = 0.58, 95% CrI 0.00–4.57). Similar results to the Schmitz model were observed when cohort studies were analysed (8 studies, 9,967 children, 6 specific drugs, *τ*^2^ = 0.07, 95% CrI 0.00–1.23), where also zidovudine administered twice (OR = 0.22, 95% CrI: 0.08–0.52) significantly reduced risk of MTCT. Restricting analysis to RCTs (3 studies, 819 children, 2 specific drugs, *τ*^2^ = 0.41, 95% CrI 0.02–3.17), zidovudine administered twice did not reduce risk of MTCT (S21 Appendix).

NMA was conducted including 6 drug categories, 12 studies (3 RCTs, 9 cohort studies), and 14,967 children. However, statistically significant heterogeneity was evident (τ^2^ = 0.66, 95% CrI 0.20–2.05). The design-by-treatment interaction model suggested statistically significant inconsistency (S17 Appendix), and the funnel plot suggested that the results might be influenced by small-study effects (S13 Appendix). The NMA results are outlined in S17 Appendix. Consistency was identified only when we restricted to studies with higher methodological quality (adequacy of follow-up and incomplete outcome data for RCTs; 3 RCTs, 4 cohort studies, 1214 children, 4 drug categories), antenatal care (3 RCTs, 3 cohort studies, 5,782 children, 6 drug categories, τ^2^ = 0.14, 95% CrI 0.00–1.98), women who used illicit drugs (1 RCT, 5 cohort studies, 5,695 children, 6 drug categories, τ^2^ = 0.92, 95% CrI 0.07–4.27), or CD4+ count (1 RCT, 5 cohort studies, 8,437 children, 6 drug categories, τ^2^ = 0.68, 95% CrI 0.06–3.24; S22 Appendix).

#### Infant and child deaths

NMA including 8 specific drugs, 13 studies (8 RCTs, 5 cohort studies), and 11,385 children suggested none of the specific ART drugs significantly reduced the risk of child deaths (τ^2^ = 0.83, 95% CrI 0.18–3.41) (S16 Appendix). Similarly, the NMA including 4 drug categories, 15 studies (7 RCTs, 8 cohort studies), and 11,451 children, suggested none of the ART categories significantly reduced the risk of child deaths (τ^2^ = 0.62, 95% CrI 0.13–2.43) (S17 Appendix).

The funnel plots suggested that the results might be influenced by publication bias and small-study effects, while the design-by-treatment interaction model suggested significant inconsistency when the ART categories were included in the NMA. These factors may importantly affect the treatment effect estimation and were explored through subsequent sensitivity analyses. We conducted sensitivity analysis including infants only, where no evidence of inconsistency was found, but the results did not importantly change from NMA (NMA sensitivity with drugs: 7 RCTs, 3 cohort studies, 4,169 infants, 6 drugs, *τ*^2^ = 0.73, 95% CrI 0.01–4.02 (S16 Appendix), NMA sensitivity with drug categories: 7 RCTs, 6 cohort studies, 4,944 infants, 4 treatments, τ^2^ = 0.29, 95% CrI 0.00–2.16) (S17 Appendix).

#### Preterm births

NMA including 17 specific drugs, 35 studies (8 RCTs, 1 case-control study, 26 cohort studies), and 20,576 infants found that compared to mothers receiving no ART, there were significantly fewer preterm births in mothers receiving the following specific ART drugs: zidovudine administered once (OR = 0.64, 95% CrI: 0.47–0.91), zidovudine combined with lamivudine and nevirapine (OR = 0.38, 95% CrI: 0.15–0.99), and zidovudine combined with nevirapine (OR = 0.00, 95% CrI: 0.00–0.02) (S16 Appendix). Compared to mothers receiving no ART, there were significantly more preterm births in mothers receiving zidovudine combined with lamivudine and indinavir (OR = 69.59, 95% CrI: 2.49–4982.00). The NMA including 17 specific drugs, 35 studies (8 RCTs, 1 case-control study, 26 cohort studies), and 20,576 infants, showed statistically significant heterogeneity (τ^2^ = 0.31, 95% CrI 0.15–0.73) and funnel plot asymmetry (S16 Appendix). This was explored with sensitivity analysis including large studies (>300 infants). In this analysis (9 specific drugs, 16 studies [5 RCTs, 11 cohort studies], 18,536 infants; *τ*^2^ = 0.44, 95% CrI 0.17–1.31), the combination of zidovudine, lamivudine, and nevirapine was no longer statistically significant (OR = 0.24, 95% CrI: 0.03–2.19), and the combination of zidovudine, lamivudine, and indinavir was no longer included in the NMA because the larger studies did not examine this combination (S21 Appendix).

We also attempted a NMA with 40 studies (5 RCTs, 35 cohort studies), 36,727 infants, and 4 drug categories, but there was evidence of significant heterogeneity (τ^2^ = 0.70, 95% CrI 0.39–1.27) and inconsistency (S17 Appendix). This was explored through sensitivity analysis including large studies (4 drug categories, 17 studies [3 RCTs, 14 cohort studies], 33,571 infants), but inconsistency was still evident (S22 Appendix).

#### Stillbirths

NMA including 20 specific drugs, 26 studies (13 RCTs, 13 cohort studies; *τ*^2^ = 0.77, 95% CrI 0.19–2.32), and 17,507 neonates found that zidovudine administered once (OR = 0.46, 95% CrI: 0.24–0.93), and zidovudine plus nevirapine (OR = 0.04, 95% CrI: 0.00–0.72) significantly reduced stillbirth risk (S16 Appendix).

The NMA including 4 drug categories, 33 studies (21 cohort studies, 12 RCTs; τ^2^ = 0.92, 95% CrI 0.33–2.28), and 21,545 neonates, showed that any ART mono-therapy significantly reduced the risk of stillbirths (OR = 0.43, 95% CrI: 0.23–0.83) (S17 Appendix).

#### Low birth weight

NMA including 16 specific drugs, 30 studies (19 cohort studies, 1 case-control study, 10 RCTs; *τ*^2^ = 0.25, 95% CrI 0.08–0.75), and 21,848 children found that compared to children in mothers who received no ART, significantly more children in mothers receiving a combination of lamivudine, stavudine, and efavirenz had low birth weight (OR = 6.12, 95% CrI: 1.04–39.13) (S16 Appendix).

We attempted a NMA combining ART drugs into 4 categories, and including 35 studies (7 RCTs, 28 cohort studies), and 31,319 children, but there was evidence of significant heterogeneity (τ^2^ = 0.23, 95% CrI 0.10–0.50), significant network inconsistency, and funnel plot asymmetry (S17 Appendix).

#### Short length and small head

Only one cohort study reported the proportion of 1,088 total neonates born with a short length. Statistically significantly more neonates of mothers who received zidovudine had a short length than those receiving control (OR = 166.67, 95% CrI: 111.11–250.00). Another cohort study reported the proportion of 62 neonates born with a small head for mothers receiving any ART versus HAART; the results were not statistically significant (OR = 0.63, 95% CrI: 0.06–6.33) (S16 and S17 Appendices).

## Discussion

### Summary of evidence

We found that none of the specific ART drugs or ART drug categories was associated with an increased risk of CMs. However, a combination of zidovudine, lamivudine, and indinavir was associated with increased risk of preterm births, a combination of zidovudine and nevirapine was associated with increased risk of stillbirths, and a combination of lamivudine, stavudine and efavirenz was associated with increased risk of low birth weight. Regarding MTCT, zidovudine administered once or twice significantly reduced the risk of MTCT. None of the included studies examined treatment with HAART in the specific ART drug analysis. Our results can be used to inform and guide ART during pregnancy.

We were unable to report on associations between specific ART and major CMs, minor CMs, short length, and small head due to a lack of studies reporting these outcomes. We were unable to draw conclusions regarding HAART in our ART category analysis for: MTCT, infant/child deaths, low birth weight, and preterm births including ART mono- and duo-therapy, because there was evidence of statistically significant heterogeneity, inconsistency, and small-study effects.

A recent systematic review and NMA comparing ART regimens in 71 RCTs with patients aged >12 years found that dolutegravir and low-dose efavirenz were more tolerable and more effective than standard-dose efavirenz, and that dolutegravir is slightly superior with respect to viral suppression efficacy and tolerability [48]. A meta-analysis of ten observational studies reported that exposure during pregnancy to ART including protease inhibitors was associated with an increased risk of preterm birth [49]. This is aligned with our NMA finding that significantly more preterm births occurred in mothers receiving an ART combination including indinavir (i.e. zidovudine plus lamivudine plus indinavir) compared to no ART. A Cochrane review of 25 RCTs with no meta-analysis found that nevirapine, and zidovudine alone or in combination with lamivudine administered to mothers during gestation, labour, or while breastfeeding, and/or given to their babies after birth significantly reduced MTCT [50]. Also, zidovudine administered during the antenatal period, followed by zidovudine administered with lamivudine intrapartum and postpartum for one week and nevirapine given to infants within 72 hours of delivery and zidovudine for one week, may be the most effective short ART course. Another Cochrane review without meta-analysis included three RCTs and six observational studies, and showed that zidovudine, lamivudine, lopinavir with ritonavir significantly reduced MTCT [51]. These findings are in agreement with our results for zidovudine when ART is administered maternally, but lamivudine and lopinavir with ritonavir were not assessed in our NMA for MTCT. Another meta-analysis of 16 cohort studies comparing efavirenz versus non-efavirenz containing regimens administered at the first trimester of pregnancy was not associated with increased risk of overall birth defects [52]. Efavirenz was included in two trials assessing total CM in our study, and the NMA suggested no statistical difference from no ART.

### Strengths and limitations

Strengths of our study include rigorous methods following guidance from The Cochrane Collaboration’s Handbook for systematic reviews [26], and ISPOR for NMAs [17]. To our knowledge, this is the first systematic review comparing different ART medications in a single model, including both observational and RCTs, and assessing both infant/child safety and MTCT effectiveness.

Several limitations are worth noting. At the request of Health Canada, the commissioner of the review, we have included all ART interventions available in the included studies; however, some of these interventions may be out-of-date from current treatment recommendations. We were unable to confirm whether the transitivity assumption held for all NMAs due to variation in confounding variables across studies. We attempted to explore this through meta-regression, subgroup, and sensitivity analyses. However, meta-regression analyses have low power to detect treatment effect modifiers and the use of a random-effects model to estimate the between-study heterogeneity may result in low statistical power especially in the presence of rare events (e.g., congenital malformations) [53, 54]. Also, the included studies represent different time periods (from 1994 to today) possibly confounding drug selection. Many important confounding variables were not reported across the included studies. Examples include: maternal viral load, adherence, drug resistance, the timing of therapy relative to delivery, concurrent medications, treatment route (orally/intravenously), dosage and duration for ART medications. As such, we considered those variables to be balanced across treatment comparisons, and that oral and intravenous routes, and dosages of the same ART drug are equivalent. Some comparison adjusted-funnel plots were asymmetric, suggesting that small studies show exaggerated safety (or efficacy for MTCT) for newer treatments. To remedy that, we conducted sensitivity analyses restricted to studies with ≥300 patients. However, asymmetry in funnel plots may also be prevalent due to variability within or between treatment comparisons. Indeed, the studies were highly heterogeneous regarding country of conduct (high-income versus LMIC), study design (RCT versus non-randomized), and other factors, as noted in S21 and S22 Appendices. To capture potential risks of synthesizing different study designs, we used the Schmitz model [32, 55, 56]. We were unable to apply NMA combining specific ART drugs in broader categories as the design-by-treatment interaction model suggested a violation of the consistency assumption. This is because the different ART drugs represent different formulations and cannot be considered equivalent, and hence cannot be combined in one treatment node [57].

In terms of future research, the long-term health outcomes of children exposed to both HIV and multiple ART drugs *in-utero*, during delivery or while breastfeeding remain largely unknown. Studies can be improved through a number of factors. RCTs could be improved by using adequate random sequence generation and allocation concealment, a pre-specified protocol to reduce selective outcome reporting, and minimizing contamination [58]. Observational studies can be improved by adjusting for important confounding variables and by accounting for patients lost to follow-up. Future studies should examine the important unique outcomes that were rarely reported across the included studies; major CMs, minor CMs, short length, and small head.

## Conclusions

The results of our systematic review found that none of the specific drugs examined were associated with an increased risk of CMs. We found that zidovudine administered once or twice significantly reduced the risk of MTCT. However, zidovudine administered with lamivudine and indinavir was associated with increased risk of preterm births, zidovudine administered with nevirapine was associated with increased risk of stillbirths, and lamivudine administered with stavudine and efavirenz was associated with increased risk of low birth weight. Our results can be used by patients who are taking ART medications during pregnancy, as well as their clinicians and policy-makers who make decisions on these drugs at a regulatory and population level.

## Supporting information

S1 Appendix Protocol registration on PROSPERO. (DOCX)

S2 Appendix PRISMA NMA checklist. (DOCX)

S3 Appendix Eligible antiretroviral medication. (DOCX)

S4 Appendix Outcomes. (DOCX)

S5 Appendix Search strategy. (DOCX)

S6 Appendix Study selection. (DOCX)

S7 Appendix Included studies. (DOCX)

S8 Appendix Study characteristics. (DOCX)

S9 Appendix Patient characteristics. (DOCX)

S10 Appendix Interventions compared in the analyses. (DOCX)

S11 Appendix Cochrane EPOC Risk-of-bias appraisal results (n = 17 RCTs). (DOCX)

S12 Appendix Newcastle-Ottawa Scale appraisal results. (DOCX)

S13 Appendix Funnel plots including antiretroviral drug categories by outcomes reported. (DOCX)

S14 Appendix Network diagrams for anti-retroviral therapy drug categories. (DOCX)

S15 Appendix Transitivity assessment results. (DOCX)

S16 Appendix Network meta-analysis and meta-analysis results for specific antiretroviral drugs by outcomes. (DOCX)

S17 Appendix Network meta-analysis and meta-analysis results for antiretroviral drug categories by outcomes. (DOCX)

S18 Appendix Plots from inconsistency assessment using the loop-specific approach. (DOCX)

S19 Appendix Forests plots for antiretroviral therapy drug categories versus no treatment/placebo for each outcome. (DOCX)

S20 Appendix Characteristics of the treatment nodes per outcome along with their surface under the cumulative ranking curve values. (DOCX)

S21 Appendix Additional analyses: Specific drugs. (DOCX)

S22 Appendix Additional analyses: Drug categories. (DOCX)
