# Supplementary material for: Comparative safety and effectiveness of perinatal antiretroviral therapies for HIV-infected women and their children: Systematic review and network meta-analysis including different study designs
Source: PLoS One. 2018 Jun 18;13(6):e0198447. doi: 10.1371/journal.pone.0198447 (PMC6005568; doi:10.1371/journal.pone.0198447)
Supplement: S1 Appendix — (DOCX) [file pone.0198447.s001.docx]

# S1 Appendix. Protocol registration on PROSPERO

Andrea Tricco, Jesmin Antony, Huda Ashoor, Areti Angeliki Veroniki, Brian Hutton, Brenda Hemmelgarn, David Moher, Yaron Finkelstein, Kevin Gough, Sharon Straus. Safety and effectiveness of antiretroviral therapies for HIV-infected women and their infants and children: protocol for a systematic review and network meta-analysis. PROSPERO 2014:CRD42014009071 Available from <http://www.crd.york.ac.uk/PROSPERO/display_record.asp?ID=CRD42014009071>

**Disseminated through peer reviewed journal publication**

Tricco AC, Antony J, Veroniki AA, Ashoor H, Hutton B, Hemmelgarn BR, Moher D, Finkelstein Y, Gough K, Straus SE. [Safety and effectiveness of antiretroviral therapies for HIV-infected women and their infants and children: protocol for a systematic review and network meta-analysis](https://systematicreviewsjournal.biomedcentral.com/articles/10.1186/2046-4053-3-51). Systematic reviews. 2014 May 25;3(1):51.

**Modifications from the Protocol to the Systematic Review**

|  | **Published in Protocol** | **Published in Systematic Review** |
| --- | --- | --- |
| **Outcomes** | Primary outcomes are MTCT and major CM (overall and by specific type). | Primary outcomes are MTCT and any CM (overall only). |
| **Information sources and literature search** | The secondary source of potentially relevant material will be a search of the gray literature, including dissertation databases (ProQuest Dissertations and Theses Database), clinical trial registries (for example, World Health Organization International Clinical Trials Search Portal), and conference abstracts from selected  international symposia on HIV. Literature saturation will be achieved by contacting antiretroviral drug manufacturers, scanning the reference lists of included studies and relevant reviews, and contacting authors who are prolific in HIV research. | ART manufacturers and authors prolific in HIV research were not specifically contacted. |
| **Data items and data collection process** | Patient characteristics collected:  mean age of the mother and infant/child, gestational stage when antiretroviral  medication administered,  family history of congenital malformations,  breastfeeding,  maternal HIV viral load,  maternal CD4 count,  history of other sexually transmitted diseases,  chorioamnionitis,  prolonged rupture of membranes,  mode of delivery,  postpartum or intrapartum hemorrhage, history of previous stillbirth,  consumption of folate,  tobacco during pregnancy,  alcohol during pregnancy | Patient characteristics abstracted were revised. Actual patient characteristics collected:  total # of women,  mean age (yrs),  gestational age,  HIV-1 definition,  HIV-1 stage,  HIV-1 duration (mos),  medication administered,  mode of delivery,  LMIC,  Illicit drug use,  alcohol consumption,  smoking,  TB co-infection,  low CD4 count,  antenatal care |
| **Methodological quality/risk of bias appraisal** | The risk of bias of experimental and quasi-experimental studies will be appraised using the Cochrane Effective Practice and Organization of Care tool for assessing risk of bias. The methodological quality of observational studies will be appraised using the Newcastle-Ottawa Scale. Publication bias will be assessed using funnel plots. Finally, studies reporting harms will be appraised  using the McHarm tool. | Did not use McHarm tool. The comparison-adjusted funnel plot has been applied including all studies contributing to the NMA in a single plot, increasing the power to detect publication bias and small-study effects. |
| **Synthesis of included studies** | Fixed and random-effects meta-analysis will be conducted separately for studies including patients receiving single or combinations of antiretroviral medications for RCTs.  We will perform network meta-analysis employing the methodology of multivariate meta-analysis in Stata using the mvmeta command.  We will estimate the magnitude of statistical heterogeneity using the restricted maximum likelihood and the Q-profile method to estimate the 95% CI. The proportion of variability that is due to heterogeneity rather than sampling error will also be quantified using the I^2^ measure.  A sequential approach will be used for the network meta-analysis, first restricted to RCTs (which will be considered the primary analysis that we will base our conclusions on), second adding quasi-experimental data, and then finally incorporating data from observational studies. Such analyses will allow the determination of the contribution of non-randomised studies to the findings from RCTs.  Summary estimates will be displayed along with their 95% confidence intervals (CI). | Random-effects meta-analysis and NMA models were applied for each outcome to account for the anticipated methodological and clinical between-study heterogeneity. We repeated all analyses for ART medications and ART categories separately.  Outcome data were pooled using Bayesian hierarchical models, and the Markov Chain Monte Carlo algorithm for both pairwise meta-analysis and NMA.  Since we analysed all data in a Bayesian setting, we calculated the posterior distribution for the between-study standard deviation (we assumed a half-normal prior distribution for the between-study standard deviation ($\tau\sim N\left( 0,1 \right), \tau>0$)). We presented the median and 95% credible interval for the between-study variance. We do not present the I^2^ measure, as it requires the $\tau^{2}$ to be estimated in a frequentist setting (and usually under the DerSimonian and Laird approach).  We used the Schmitz et al. model [1] to combine randomised with non-randomised data, allowing for different heterogeneity parameters in different study designs. We did not introduce bias adjustment to account for over-precision or for over-/under-estimation, as we were uncertain about the magnitude of bias that might have been introduced from including the observational studies. The model provides NMA estimates for the combined data, as well as for randomised, and non-randomised data separately.  We present the summary treatment effect estimates with a 95% credible interval (CrI) for each pair of treatments. For the NMA effect estimates, we also present a 95% predictive interval (PrI), which captures the magnitude of $\tau^{2}$ and presents the interval within which we would expect the treatment effect of a future study to lie. |
