# Supplementary material for: Comparative safety and effectiveness of perinatal antiretroviral therapies for HIV-infected women and their children: Systematic review and network meta-analysis including different study designs
Source: PLoS One. 2018 Jun 18;13(6):e0198447. doi: 10.1371/journal.pone.0198447 (PMC6005568; doi:10.1371/journal.pone.0198447)
Supplement: S3 Appendix — (DOCX) [file pone.0198447.s003.docx]

# S3 Appendix. Eligible Antiretroviral Medication

| **Common name** | **Abbreviation** | **Brand name** | **Approval Date** |
| --- | --- | --- | --- |
| ***Nukes (Nucleoside Reverse Transcriptase Inhibitors [NRTIs])*** | | |  |
| Abacavir, | ABC | Ziagen | 9/10/2001 |
| Didanosine, | ddI | Videx EC (enteric-coated) | 10/4/2001 |
| Emtricitabine*, | FTC | FTC; Emtriva | 11/21/2005 |
| Lamivudine, | 3TC | 3TC; Epivir | 8/26/2003 |
| Stavudine, | d4T | Zerit | 12/15/1997 |
| Tenofovir | TDF | Viread | 3/18/2003 |
| Zidovudine | AZT (ZDV) | Retrovir | 12/31/1991 |
| ***Non-nukes [NNRTIs]*** | | |  |
| Delaviridine | DLV | Rescriptor | 7/22/1998 |
| Efavirenz | EFV | Sustiva | 7/4/2002 |
| Etravirine | ETR | Intelence | 11/20/2012 |
| Nevirapine | NVP | Viramune | 5/6/2011 |
| Rilpivirine | RPV | Edurant | 7/21/2011 |
| ***Protease inhibitors*** | | |  |
| Atazanavir | ATZ | Reyataz | 5/2/2007 |
| Darunavir | DRV | Prezista | 11/19/2013 |
| Fosamprenavir | FPV | Telzir | 10/12/2004 |
| Indinavir | IND | Crixivan | 9/13/1996 |
| Lopinavir† | LPV (LOP) | Kaletra | 6/10/2008† |
| Nelfinavir | NFV (NLF) | Viracept | 12/30/2003 |
| Ritonavir | RIT; R | Norvir | 9/27/2010 |
| Saquinavir | SAQ | Invirase | 3/31/2006 |
| Tipranavir | TPV | Aptivus | 11/21/2005 |
| ***Integrase inhibitor*** | | |  |
| Raltegravir | RAL | Isentress | 12/6/2012 |
| ***Fusion inhibitors*** | | |  |
| Enfuvirtide, T-20 | ENF | Fuzeon | 7/14/2003 |
| ***Co-receptor inhibitor/CCR5 antagonist*** | | |  |
| Maraviroc | MVC | Celsentri | 12/21/2009 |
| **Notes:** Abbreviations in brackets are used in this review.  *is only available in Canada co-formulated with tenofovir, as Truvada. †Co-formulated with Ritonavir | | | |
