# Supplementary material for: Comparative safety and effectiveness of perinatal antiretroviral therapies for HIV-infected women and their children: Systematic review and network meta-analysis including different study designs
Source: PLoS One. 2018 Jun 18;13(6):e0198447. doi: 10.1371/journal.pone.0198447 (PMC6005568; doi:10.1371/journal.pone.0198447)
Supplement: S4 Appendix — (DOCX) [file pone.0198447.s004.docx]

# S4 Appendix. Outcomes

| **Outcome** | **Definition/Measure/Cut-off value** |
| --- | --- |
| **Major congenital malformation (CM)** | Malformation present at birth with surgical, medical, functional, or cosmetic importance |
| **Minor congenital malformation** | CM that does not qualify to be classified as a Major CM |
| **Mother-to-Child Transmission of HIV (MTCT)** | As defined by study authors and evidenced by AIDS-defining events before and beyond 18 months of age and at least one or more positive screening for HIV-1 (HIV-1 PCR, Western blot assay, p24 antigen, HIV-1 ELISA) |
| **Infant/Child deaths** | Infant/child mortality at 6 months, 12, or 18 months; or infant mortality during <78 weeks follow up; or as defined by study authors ^a^ |
| **Preterm Birth** | Delivery at <37 weeks gestation; or as defined by study authors (no cut-off value reported) |
| **Stillbirths** | Loss of pregnancy >20 weeks from last menstrual period (gestational age of fetus); or defined by study authors as stillbirth, spontaneous abortion, miscarriage, or fetal demise |
| **Low Birth Weight (LBW)** | Birth weight <2,500 g; or <10^th^ percentile; or birth weight z score ≤−2 for gestational age and gender |
| **Small Head** | Diagnosis of microcephaly at birth |
| **Short Length** | Birth height z score ≤−2 for gestational age and gender |

^a^ One included study (Habib 2008) described infant deaths as perinatal mortality which could include stillbirths
