# Supplementary material for: Comparative safety and effectiveness of perinatal antiretroviral therapies for HIV-infected women and their children: Systematic review and network meta-analysis including different study designs
Source: PLoS One. 2018 Jun 18;13(6):e0198447. doi: 10.1371/journal.pone.0198447 (PMC6005568; doi:10.1371/journal.pone.0198447)
Supplement: S5 Appendix — (DOCX) [file pone.0198447.s005.docx]

# S5 Appendix. Search Strategy

Database: Ovid MEDLINE(R) <1946 to February Week 4 2014>, Ovid MEDLINE(R) In-Process & Other Non-Indexed Citations <March 10, 2014>

Search Strategy:

------------------------------------------------------------------------

1 exp HIV Infections/ [ HIV terms ]

2 exp HIV/

3 HIV.tw.

4 HIV-1$.tw.

5 HIV-2$.tw.

6 HIV1.tw.

7 HIV2.tw.

8 (HIV adj infect$).tw.

9 "human immunodeficiency virus$".tw.

10 "human immunedeficiency virus$".tw.

11 "human immuno-deficiency virus$".tw.

12 "human immune-deficiency virus$".tw.

13 ((human adj immun$) and (deficiency adj virus$)).tw.

14 "acquired immunodeficiency syndrome".tw.

15 "acquired immunedeficiency syndrome".tw.

16 "acquired immuno-deficiency syndrome".tw.

17 "acquired immune-deficiency syndrome".tw.

18 ((acquired adj immun$) and (deficiency adj syndrome)).tw.

19 "Sexually Transmitted Diseases, Viral"/

20 or/1-19

21 Maternal-Fetal Exchange/ [ Pregnancy / breastfeeding terms ]

22 exp Fetus/

23 exp Pregnancy/

24 (adult adj2 child$).tw.

25 (adult adj2 infant$).tw.

26 antenatal$.tw.

27 ante-natal$.tw.

28 (disease adj transmission$).tw.

29 fetomaternal.tw.

30 foetomaternal.tw.

31 feto-maternal.tw.

32 foeto-maternal.tw.

33 fetus$.tw.

34 foetus$.tw.

35 gestation$.mp.

36 inutero.tw.

37 (in adj utero).tw.

38 intraueterin$.tw.

39 (intra adj uterin$).tw.

40 (mother adj2 child$).tw.

41 (mother adj2 infant$).tw.

42 (mother adj2 fetal).tw.

43 (mother adj2 foetal).tw.

44 (mother adj2 fetus$).tw.

45 (mother adj2 foetus$).tw.

46 (maternal adj2 child$).tw.

47 (maternal adj2 fetal).tw.

48 (maternal adj2 foetal).tw.

49 (maternal adj2 fetus$).tw.

50 (maternal adj2 infant$).tw.

51 MTCT.tw.

52 pregnan$.tw.

53 prenatal$.tw.

54 pre-natal$.tw.

55 perinatal$.tw.

56 peri-natal$.tw.

57 prepartum.tw.

58 pre-partum.tw.

59 prebirth.tw.

60 pre-birth.tw.

61 transplacenta$.tw.

62 trans-placenta$.tw.

63 (vertical adj transmission).tw.

64 Infectious Disease transmission, Vertical/

65 Breast Feeding/

66 Lactation/

67 Milk, Human/

68 breastfe$.tw.

69 (breast$ adj fed$).tw.

70 (breast adj feed$).tw.

71 breastmilk$.tw.

72 (breast$ adj milk$).tw.

73 lactat$.tw.

74 (mother$ adj milk$).tw.

75 (human adj milk$).tw.

76 (maternal$ adj milk$).tw.

77 (nursing adj mother$).tw.

78 (nursing adj baby).tw.

79 (nursing adj babies).tw.

80 (nursing adj infant$).tw.

81 (nursing adj newborn$).tw.

82 (nursing adj neonat$).tw.

83 (nursing adj neo-nat$).tw.

84 (nursing adj perinat$).tw.

85 (nursing adj peri-nat$).tw.

86 (nursing adj premie).tw.

87 (nursing adj premies).tw.

88 (nursing adj prematur$).tw.

89 (nursing adj preterm$).tw.

90 (nursing adj pre-term$).tw.

91 or/21-90

92 exp HIV Infections/dt [Drug Therapy]

93 exp Anti-HIV Agents/

94 HIV Protease Inhibitors/

95 Integrase Inhibitors/

96 Reverse Transcriptase Inhibitors/

97 anti-HIV$.tw.

98 anti-AIDs.tw.

99 (anti-retroviral$ and AIDs).tw.

100 (anti-retroviral$ and HIV$).tw.

101 (antiretroviral$ and AIDs).tw.

102 (antiretroviral$ and HIV$).tw.

103 (HIV adj2 inhibitor$).tw.

104 (HIV-1 adj2 inhibitor$).tw.

105 (HIV1 adj2 inhibitor$).tw.

106 (HIV-2 adj2 inhibitor$).tw.

107 (HIV2 adj2 inhibitor$).tw.

108 HAART.tw.

109 (integrase adj inhibitor$).tw.

110 "nucleoside reverse transcriptase inhibitor$".tw.

111 "non-nucleoside reverse transcriptase inhibitor$".tw.

112 "nonnucleoside reverse transcriptase inhibitor$".tw.

113 NRTI$.tw.

114 NNRTI$.tw.

115 (protease and AIDs).tw.

116 (protease and HIV$).tw.

117 (proteinase and AIDs).tw.

118 (proteinase and HIV$).tw.

119 darunavir$.mp.

120 prezista.tw.

121 darunavir.rn. [ CAS Registry ]

122 TMC-114.tw.

123 TMC114.tw.

124 abacavir.tw,rn.

125 ziagen.tw.

126 Didanosine/

127 didanosine.tw,rn.

128 didanosin$.tw.

129 dideoxyinosine.tw.

130 videx.tw.

131 emtricitabine.tw,rn.

132 emtriva.tw.

133 truvada.tw.

134 coviracil.tw.

135 racivir.tw.

136 Lamivudine/

137 lamivudine.tw,rn.

138 epivir-HBV.tw.

139 epzicom.tw.

140 combivir.tw.

141 heptovir.tw.

142 hepitec.tw.

143 trizivir.tw.

144 epivir.tw.

145 zeffix.tw.

146 Stavudine/

147 stavudin$.tw.

148 stavudine.rn.

149 zerut.tw.

150 estavudina.tw.

151 sanilvudine.tw.

152 zerit.tw.

153 Zidovudine/

154 ZDV.tw.

155 AZT.tw.

156 zidovudin$.tw.

157 zidovudine.rn.

158 azidothymidine.tw.

159 retrovir.tw.

160 tenofovir.tw,rn.

161 apropovir.tw.

162 viread.tw.

163 Delavirdine/

164 delavirdine.tw,rn.

165 DLV.tw.

166 rescriptor.tw.

167 efavirenz.tw,rn.

168 EFV.tw.

169 sustiva.tw.

170 stocrin.tw.

171 Nevirapine/

172 nevirapine.tw,rn.

173 NVP.tw.

174 viramune.tw.

175 etravirine.tw,rn.

176 ETV.tw.

177 intelence.tw.

178 atazanavir.tw,rn.

179 ATV.tw.

180 ATZ.tw.

181 latazanavir.tw.

182 zrivada.tw.

183 reyataz.tw.

184 fosamprenavir.tw,rn.

185 telzir.tw.

186 lexiva.tw.

187 Indinavir/

188 indinavir.tw,rn.

189 IDV.tw.

190 crixivan.tw.

191 Lopinavir/

192 lopinavir.tw,rn.

193 aluvia.tw.

194 aluviran.tw.

195 koletra.tw.

196 kaletra.tw.

197 Nelfinavir/

198 nelfinavir.tw.

199 nelfinavir.rn.

200 Viracept.tw.

201 Ritonavir/

202 ritonavir.tw,rn.

203 RTV.tw.

204 norvir.tw.

205 Saquinavir/

206 saquinavir.tw,rn.

207 fortovase.tw.

208 invirase.tw.

209 tipranavir.tw,rn.

210 TPV.tw.

211 aptivus.tw.

212 enfuvirtide.tw.

213 enfuvirtide.rn.

214 pentafuside.tw.

215 fuzeon.tw.

216 maraviroc.tw,rn.

217 MVC.tw.

218 celsentri.tw.

219 selzentry.tw.

220 raltegravir.tw.

221 raltegravir.rn.

222 isentress.tw.

223 rilpivirine.tw,rn.

224 edurant.tw.

225 or/92-224

226 randomized controlled trial.pt. [ RCT filter - validated ]

227 randomized.mp.

228 placebo.mp.

229 or/226-228

230 Controlled Clinical Trial/ [ quasi-expt-observ study filter ]

231 Observational Study/

232 (descriptive adj3 stud$).tw.

233 (descriptive adj3 design).tw.

234 (descriptive adj3 analys?s).tw.

235 nonrandom$.tw.

236 non-random$.tw.

237 non-experiment$.tw.

238 nonexperiment$.tw.

239 (natural adj experiment?).tw.

240 (observational$ adj3 stud$).tw.

241 (observational$ adj3 design).tw.

242 (observational$ adj3 analys?s).tw.

243 quasirandom$.tw.

244 quasi-random$.tw.

245 quasiexperimental.tw.

246 quasi-experimental.tw.

247 exp Cohort Studies/

248 Registries/

249 Epidemiologic Methods/

250 limit 249 to yr=1971-1988

251 cohort$.tw.

252 (follow-up adj stud$).tw.

253 (followup adj stud$).tw.

254 (follow-up adj design).tw.

255 (followup adj design).tw.

256 (follow-up adj analys?s).tw.

257 (followup adj analys?s).tw.

258 (follow-up and base-line).tw.

259 (followup and baseline).tw.

260 longitudinal.tw.

261 ("long term" adj stud$).tw.

262 (longterm adj stud$).tw.

263 ("long term" adj design).tw.

264 (longterm adj design).tw.

265 ("long term" adj analys?s).tw.

266 (longterm adj analys?s).tw.

267 (population adj stud$).tw.

268 (population adj analys?s).tw.

269 prospective.tw.

270 retrospective.tw.

271 registry.tw.

272 registries.tw.

273 Cross-Sectional Studies/

274 (cross adj sectional).tw.

275 (incidence adj stud$).tw.

276 (prevalence adj stud$).tw.

277 (transversal adj stud$).tw.

278 exp Case-Control Studies/

279 Control Groups/

280 Matched-Pair Analysis/

281 (case$ adj3 control$).tw.

282 (case adj3 comparison$).tw.

283 (case$ and series).tw.

284 case-referent.tw.

285 (control$ adj3 stud$).tw.

286 (control adj group$).tw.

287 before-after.tw.

288 "before and after".tw.

289 (before adj after).tw.

290 (time adj series).tw.

291 Evaluation Studies/

292 Comparative Study/

293 Intervention Studies/

294 Multicenter Study/

295 Pilot Projects/

296 Program Evaluation/

297 Validation Studies/

298 (comparative adj stud$).tw.

299 (comparison adj stud$).tw.

300 (evaluation adj stud$).tw.

301 effectiveness.tw.

302 intervention.tw.

303 (multicenter adj stud$).tw.

304 (multi-center adj stud$).tw.

305 (multicenter adj stud$).tw.

306 (multi-center adj stud$).tw.

307 (multidimensional adj stud$).tw.

308 (multi-dimensional adj stud$).tw.

309 (pre- adj5 post-).tw.

310 (pretest adj5 posttest).tw.

311 (program$ adj6 evaluat$).tw.

312 or/230-248,250-311

313 229 or 312

314 20 and 91 and 225 and 313

315 exp Animals/ not (exp Animals/ and Humans/)

316 314 not 315
