# Supplementary material for: Comparative safety and effectiveness of perinatal antiretroviral therapies for HIV-infected women and their children: Systematic review and network meta-analysis including different study designs
Source: PLoS One. 2018 Jun 18;13(6):e0198447. doi: 10.1371/journal.pone.0198447 (PMC6005568; doi:10.1371/journal.pone.0198447)
Supplement: S6 Appendix — (DOCX) [file pone.0198447.s006.docx]

# S6 Appendix. Study Selection

**Process to identify additional data**

- Authors were contacted
- References of relevant studies were scanned

**Review team agreement:**

| **Total # pilot-tests:** | |
| --- | --- |
| Level 1 –Titles and Abstracts screening  Level 2 – Full text screening  Data Abstraction  Quality Appraisal | 2  2  2  1 |
| **% agreement achieved during pilot tests** | |
| Level 1 –Titles and Abstracts screening  Level 2 – Full text screening  Data Abstraction  Quality Appraisal | 72%  65%  Judgment*  Judgment* |
| **% discrepancies during study selection and data abstraction** | |
| Level 1 –Titles and Abstracts screening  Level 2 – Full text screening  Data Abstraction  Quality Appraisal | 13%  25%  80%†  60%† |

**Notes**: *Agreement between reviewers on main data items was judged to be sufficient.†Approximate proportion of discrepancies in all data items across all included studies.

**Author Contact:**

| Total # of authors contacted: | Contacted 98 authors, of which 25 responded |
| --- | --- |
| # studies with additional data provided: | 9 authors contacted for more information sent us additional information |
| # unpublished study data provided: | 2 included studies (1 CA and 1 letter) and 4 CRs (3 CAs and 1 editorial) provided unpublished data |
| # published studies provided that we did not identify through our search from authors: | 3 included studies and 6 CRs were identified through reference scanning |
| # studies with additional data that was included in our analysis from authors: | 5 studies with additional data included in analysis (4 included studies, 1 CR) |
| Authors’ response rate: | Contacted 98, 25 responded, 9 positive responses with additional information |

**Abbreviations:** CA, conference abstract; CR, companion report.
