# Supplementary material for: Comparative safety and effectiveness of perinatal antiretroviral therapies for HIV-infected women and their children: Systematic review and network meta-analysis including different study designs
Source: PLoS One. 2018 Jun 18;13(6):e0198447. doi: 10.1371/journal.pone.0198447 (PMC6005568; doi:10.1371/journal.pone.0198447)
Supplement: S7 Appendix — (DOCX) [file pone.0198447.s007.docx]

# S7 Appendix. Included studies

After screening 6,468 citations and 1,373 full-text articles, 72 cohorts or registries [1-72], 17 RCTs [73-89], and 1 case control study [90] were included for a total of 90 included studies [1-90], and 90 companion reports were also identified [91-180].

1. Alvarez JR, Bardeguez A, Iffy L, Apuzzio JJ. Preterm premature rupture of membranes in pregnancies complicated by human immunodeficiency virus infection: a single center's five-year experience. The journal of maternal-fetal & neonatal medicine : the official journal of the European Association of Perinatal Medicine, the Federation of Asia and Oceania Perinatal Societies, the International Society of Perinatal Obstet. 2007;20(12):853-7. Epub 2007/10/24. doi: 10.1080/14767050701700766. PubMed PMID: 17952817.

2. Areechokchai D, Bowonwatanuwong C, Phonrat B, Pitisuttithum P, Maek-a-Nantawat W. Pregnancy outcomes among HIV-infected women undergoing antiretroviral therapy. The open AIDS journal. 2009;3:8.

3. Asavapiriyanont S, Kasiwat S. Prevalence of low birthweight infants in HIV-infected women delivered in Rajavithi Hospital. Journal of the Medical Association of Thailand= Chotmaihet thangphaet. 2011;94:S66-70.

4. Bae W, Wester C, Smeaton L, Shapiro R, Lockman S, Onyait K, et al. Hematologic and hepatic toxicities associated with antenatal and postnatal exposure to maternal highly active antiretroviral therapy among infants. AIDS (London, England). 2008;22(13):1633.

5. Bailey H, Townsend CL, Semenenko I, Malyuta R, Cortina-Borja M, Thorne C. Impact of expanded access to combination antiretroviral therapy in pregnancy: results from a cohort study in Ukraine. Bulletin of the World Health Organization. 2013;91(7):491-500. Epub 2013/07/05. doi: 10.2471/blt.12.114405. PubMed PMID: 23825876; PubMed Central PMCID: PMCPmc3699794.

6. Barral MF, Oliveira GRd, Lobato RC, Mendoza-Sassi RA, Martínez AM, Gonçalves CV. Risk factors of HIV-1 vertical transmission (VT) and the influence of antiretroviral therapy (ART) in pregnancy outcome. Revista do Instituto de Medicina Tropical de São Paulo. 2014;56(2):133-8.

7. Bellon Cano JM, Sanchez-Ramon S, Ciria L, Leon JA, Gurbindo D, Fortuny C, et al. The effects on infants of potent antiretroviral therapy during pregnancy: a report from Spain. Medical science monitor : international medical journal of experimental and clinical research. 2004;10(5):Cr179-84. Epub 2004/04/29. PubMed PMID: 15114266.

8. Bera E, McCausland K, Nonkwelo R, Mgudlwa B, Chacko S, Majeke B. Birth defects following exposure to efavirenz-based antiretroviral therapy during pregnancy: a study at a regional South African hospital. AIDS (London, England). 2010;24(2):283-9. Epub 2009/10/30. doi: 10.1097/QAD.0b013e328333af32. PubMed PMID: 19864931.

9. Blood E, Beckwith C, Bazerman L, Cu-Uvin S, Mitty J. Pregnancy among HIV-infected refugees in Rhode Island. AIDS care. 2009;21(2):207-11. Epub 2009/02/21. doi: 10.1080/09540120801932173. PubMed PMID: 19229690.

10. Boer K, Nellen JF, Patel D, Timmermans S, Tempelman C, Wibaut M, et al. The AmRo study: pregnancy outcome in HIV-1-infected women under effective highly active antiretroviral therapy and a policy of vaginal delivery. BJOG : an international journal of obstetrics and gynaecology. 2007;114(2):148-55. Epub 2007/02/20. doi: 10.1111/j.1471-0528.2006.01245.x. PubMed PMID: 17305888.

11. Brogly SB, Abzug MJ, Watts DH, Cunningham CK, Williams PL, Oleske J, et al. Birth defects among children born to human immunodeficiency virus-infected women: pediatric AIDS clinical trials protocols 219 and 219C. The Pediatric infectious disease journal. 2010;29(8):721-7. Epub 2010/06/12. doi: 10.1097/INF.0b013e3181e74a2f. PubMed PMID: 20539252; PubMed Central PMCID: PMCPmc2948952.

12. Bucceri AM, Somigliana E, Matrone R, Ferraris G, Rossi G, Grossi E, et al. Combination antiretroviral therapy in 100 HIV-1-infected pregnant women. Human reproduction (Oxford, England). 2002;17(2):436-41. Epub 2002/02/01. PubMed PMID: 11821291.

13. Chansinghakul D, Soongswang K, Pancharoen C, Thaithumyanon P, Limpongsanurak S, Thisyakorn U. Prevention of mother-to-child HIV transmission: MTCT-PLUS initiative program. Journal of Pediatric Infectious Diseases. 2009;4(3):281-7.

14. Chen JY, Ribaudo HJ, Souda S, Parekh N, Ogwu A, Lockman S, et al. Highly active antiretroviral therapy and adverse birth outcomes among HIV-infected women in Botswana. Journal of Infectious Diseases. 2012;206(11):1695-705.

15. Chmait R, Franklin P, Spector SA, Hull AD. Protease inhibitors and decreased birth weight in HIV-infected pregnant women with impaired glucose tolerance. Journal of perinatology: official journal of the California Perinatal Association. 2002;22(5):370-3.

16. Contu L, Pintus AE, Serri F, Atzeni S, Arras M, Belloni M, et al. Zidovudine therapy from the beginning of gestation until delivery in hiv-1positive women: safety and effectiveness on vertical infection. Medical science research. 1995;23(11):727-31.

17. Cotter AM, Brookfield KF, Duthely LM, Quintero VHG, Potter JE, O'Sullivan MJ. Duration of membrane rupture and risk of perinatal transmission of HIV-1 in the era of combination antiretroviral therapy. American journal of obstetrics and gynecology. 2012;207(6):482. e1-. e5.

18. Darak S, Darak T, Kulkarni S, Kulkarni V, Parchure R, Hutter I, et al. Effect of highly active antiretroviral treatment (HAART) during pregnancy on pregnancy outcomes: experiences from a PMTCT program in western India. AIDS patient care and STDs. 2013;27(3):163-70.

19. Duran AS, Ivalo SA, Hakim A, Masciottra FM, Zlatkes R, Adissi L, et al. Prevention of mother to child HIV transmission. MEDICINA-BUENOS AIRES-. 2006;66(1):24.

20. European Collaborative Study. The mother-to-child HIV transmission epidemic in Europe: evolving in the East and established in the West. AIDS (London, England). 2006;20(10):1419-27. Epub 2006/06/23. doi: 10.1097/01.aids.0000233576.33973.b3. PubMed PMID: 16791017.

21. Ezechi OC, David AN, Gab-Okafor CV, Ohwodo H, Oladele DA, Kalejaiye OO, et al. Incidence of and socio-biologic risk factors for spontaneous preterm birth in HIV positive Nigerian women. BMC pregnancy and childbirth. 2012;12(1):1.

22. Fiore S, Newell ML, Trabattoni D, Thorne C, Gray L, Savasi V, et al. Antiretroviral therapy-associated modulation of Th1 and Th2 immune responses in HIV-infected pregnant women. Journal of reproductive immunology. 2006;70(1-2):143-50. Epub 2006/01/21. doi: 10.1016/j.jri.2005.12.001. PubMed PMID: 16423410.

23. Fitzgerald FC, Bekker L-G, Kaplan R, Myer L, Lawn SD, Wood R. Mother-to-child transmission of HIV in a community-based antiretroviral clinic in South Africa. South African medical journal. 2010;100(12):827-31. PubMed PMID: PMC3954611.

24. Floridia M, Tamburrini E, Ravizza M, Anzidei G, Tibaldi C, Bucceri A, et al. Antiretroviral therapy at conception in pregnant women with HIV in Italy: wide range of variability and frequent exposure to contraindicated drugs. Antiviral therapy. 2006;11(7):941-6. Epub 2007/02/17. PubMed PMID: 17302258.

25. Frenkel LM, Cowles MK, Shapiro DE, Melvin AJ, Watts DH, McLellan C, et al. Analysis of the maternal components of the AIDS clinical trial group 076 zidovudine regimen in the prevention of mother-to-infant transmission of human immunodeficiency virus type 1. The Journal of infectious diseases. 1997;175(4):971-4. Epub 1997/04/01. PubMed PMID: 9086162.

26. Gartland MG, Chintu NT, Li MS, Lembalemba MK, Mulenga SN, Bweupe M, et al. Field effectiveness of combination antiretroviral prophylaxis for the prevention of mother-to-child HIV transmission in rural Zambia. AIDS (London, England). 2013;27(8):1253-62. Epub 2013/01/18. doi: 10.1097/QAD.0b013e32835e3937. PubMed PMID: 23324656; PubMed Central PMCID: PMCPmc3836017.

27. Gibb DM, Kizito H, Russell EC, Chidziva E, Zalwango E, Nalumenya R, et al. Pregnancy and infant outcomes among HIV-infected women taking long-term ART with and without tenofovir in the DART trial. PLoS Med. 2012;9(5):e1001217. Epub 2012/05/23. doi: 10.1371/journal.pmed.1001217. PubMed PMID: 22615543; PubMed Central PMCID: PMCPmc3352861.

28. Goldstein PJ, Smit R, Stevens M, Sever JL. Association between HIV in pregnancy and antiretroviral therapy, including protease inhibitors and low birth weight infants. Infectious diseases in obstetrics and gynecology. 2000;8(2):94-8. Epub 2000/05/11. doi: 10.1155/s1064744900000089. PubMed PMID: 10805364; PubMed Central PMCID: PMCPmc1784673.

29. Grosch-Worner I, Schafer A, Obladen M, Maier RF, Seel K, Feiterna-Sperling C, et al. An effective and safe protocol involving zidovudine and caesarean section to reduce vertical transmission of HIV-1 infection. AIDS (London, England). 2000;14(18):2903-11. Epub 2001/01/12. PubMed PMID: 11153672.

30. Habib N, Daltveit A, Bergsjø P, Shao J, Oneko O, Lie R. Maternal HIV status and pregnancy outcomes in northeastern Tanzania: a registry‐based study. BJOG: An International Journal of Obstetrics & Gynaecology. 2008;115(5):616-24.

31. Hankin C, Lyall H, Willey B, Peckham C, Masters J, Tookey P. In utero exposure to antiretroviral therapy: feasibility of long-term follow-up. AIDS care. 2009;21(7):809-16.

32. Hoffman R, Black V, Technau K, van der Merwe KJ, Currier J, Coovadia A, et al. Effects of highly active antiretroviral therapy duration and regimen on risk for mother-to-child transmission of HIV in Johannesburg, South Africa. Journal of acquired immune deficiency syndromes 2010;54(1):35.

33. Hussain A, Moodley D, Naidoo S, Esterhuizen TM. Pregnant women's access to PMTCT and ART services in South Africa and implications for universal antiretroviral treatment. PloS one. 2011;6(12):e27907. Epub 2011/12/14. doi: 10.1371/journal.pone.0027907. PubMed PMID: 22162993; PubMed Central PMCID: PMCPmc3230616.

34. Joao EC, Calvet GA, Krauss MR, Freimanis Hance L, Ortiz J, Ivalo SA, et al. Maternal antiretroviral use during pregnancy and infant congenital anomalies: the NISDI perinatal study. Journal of acquired immune deficiency syndromes (1999). 2010;53(2):176-85. Epub 2010/01/28. doi: 10.1097/QAI.0b013e3181c5c81f. PubMed PMID: 20104119; PubMed Central PMCID: PMCPmc2901917.

35. John AMS, Kumar A, Cave C. Reduction in perinatal transmission and mortality from human immunodeficiency virus after intervention with zidovudine in Barbados. The Pediatric infectious disease journal. 2003;22(5):422-5.

36. Lemos LM, Rocha TF, Conceicao MV, Silva Ede L, Santos AH, Gurgel RQ. Evaluation of preventive measures for mother-to-child transmission of HIV in Aracaju, State of Sergipe, Brazil. Revista da Sociedade Brasileira de Medicina Tropical. 2012;45(6):682-6. Epub 2013/01/09. PubMed PMID: 23295868.

37. Leroy V, Ekouevi DK, Becquet R, Viho I, Dequae-Merchadou L, Tonwe-Gold B, et al. 18-month effectiveness of short-course antiretroviral regimens combined with alternatives to breastfeeding to prevent HIV mother-to-child transmission. PloS one. 2008;3(2):e1645.

38. Lin HC, Wang SM, Wu CS, Chang FM, Liu CC. Clinical and immunologic characteristics and therapeutic interventions in children born to human immunodeficiency virus-infected mothers in southern Taiwan. Journal of microbiology, immunology, and infection = Wei mian yu gan ran za zhi. 2005;38(2):89-95. Epub 2005/04/22. PubMed PMID: 15843852.

39. Lindegren ML, Rhodes P, Gordon L, Fleming P. Drug safety during pregnancy and in infants. Lack of mortality related to mitochondrial dysfunction among perinatally HIV-exposed children in pediatric HIV surveillance. Annals of the New York Academy of Sciences. 2000;918:222-35. Epub 2000/12/29. PubMed PMID: 11131709.

40. Lopez M, Figueras F, Hernandez S, Lonca M, Garcia R, Palacio M, et al. Association of HIV infection with spontaneous and iatrogenic preterm delivery: effect of HAART. AIDS (London, England). 2012;26(1):37-43.

41. Lussiana C, Clemente SV, Ghelardi A, Lonardi M, Pulido Tarquino IA, Floridia M. Effectiveness of a prevention of mother-to-child HIV transmission programme in an urban hospital in Angola. PloS one. 2012;7(4):e36381. Epub 2012/05/05. doi: 10.1371/journal.pone.0036381. PubMed PMID: 22558455; PubMed Central PMCID: PMCPmc3340343.

42. Mandelbrot L, Landreau-Mascaro A, Rekacewicz C, Berrebi A, Benifla JL, Burgard M, et al. Lamivudine-zidovudine combination for prevention of maternal-infant transmission of HIV-1. Jama. 2001;285(16):2083-93. Epub 2001/05/10. PubMed PMID: 11311097.

43. Mania A, Kemnitz P, Cudnoch K, Mazur-Melewska K, Figlerowicz M, Sluzewski W. Clinical condition and transmission of coinfections with human cytomegalovirus in infants of HIV-1 infected mothers in the era of mother-to-child-transmission prophylaxis. Early human development. 2013;89(2):119-24. Epub 2012/10/02. doi: 10.1016/j.earlhumdev.2012.08.008. PubMed PMID: 23021943.

44. Marazzi MC, Palombi L, Nielsen-Saines K, Haswell J, Zimba I, Magid NA, et al. Extended antenatal use of triple antiretroviral therapy for prevention of mother-to-child transmission of HIV-1 correlates with favorable pregnancy outcomes. AIDS (London, England). 2011;25(13):1611-8. Epub 2011/06/16. doi: 10.1097/QAD.0b013e3283493ed0. PubMed PMID: 21673553.

45. Marczynska M, Szczepanska-Putz M. Vertical transmission of HIV-1 in Poland. Scand J Infect Dis. 2000;32(2):165-7. Epub 2000/05/29. PubMed PMID: 10826902.

46. Matheson PB, Abrams EJ, Thomas PA, Hernan MA, Thea DM, Lambert G, et al. Efficacy of antenatal zidovudine in reducing perinatal transmission of human immunodeficiency virus type 1. The New York City Perinatal HIV Transmission Collaborative Study Group. The Journal of infectious diseases. 1995;172(2):353-8. Epub 1995/08/01. PubMed PMID: 7622877.

47. Mazur-Melewska K, Mania A, Służewski W. Vertical transmission of HIV – the influence of antiretroviral prevention on the infants health. HIV & AIDS Review. 2005;4(3):13-7.

48. McGowan JP, Crane M, Wiznia AA, Blum S. Combination antiretroviral therapy in human immunodeficiency virus-infected pregnant women. Obstetrics and gynecology. 1999;94(5 Pt 1):641-6. Epub 1999/11/05. PubMed PMID: 10546703.

49. Meyer SA, Westreich DJ, Patel E, Ehlinger EP, Kalilani L, Lovingood RV, et al. Postnatal cytomegalovirus exposure in infants of antiretroviral-treated and untreated HIV-infected mothers. Infectious diseases in obstetrics and gynecology. 2014;2014.

50. Money D vSJ, Maan E, Chaworth-Musters T, Tatum S, Espinosa V, Burdge D, Gilgoff S, Alimenti A, Forbes J. Perinatal outcomes in a cohort of ART treated HIV positive pregnant women in British Columbia [abstract]. Can J Infect Dis Med Microbiol 2007;18:(Suppl B):31B.

51. Msellati P, Hingst G, Kaba F, Viho I, Welffens-Ekra C, Dabis F. Operational issues in preventing mother-to-child transmission of HIV-1 in Abidjan, Cote d'Ivoire, 1998-99. Bulletin of the World Health Organization. 2001;79(7):641-7. Epub 2001/08/02. PubMed PMID: 11477967; PubMed Central PMCID: PMCPmc2566468.

52. Mussi-Pinhata MM, Kato CM, Duarte G, Paschoini MC, Bettiol H, Quintana SM. Factors associated with vertical HIV transmission during two different time periods: the impact of zidovudine use on clinical practice at a Brazilian reference centre. International journal of STD & AIDS. 2003;14(12):818-25. Epub 2003/12/18. doi: 10.1258/095646203322556156. PubMed PMID: 14678590.

53. Onakewhor Joseph, Olagbuji Biodun, Ezeanochie Michael. Pregnancy outcome among HIV positive women receiving antenatal HAART versus untreated maternal HIV infection. Journal of the College of Physicians and Surgeons Pakistan. 2011;21(6):356-9.

54. Parker MM, Wade N, Lloyd RM, Jr., Birkhead GS, Gallagher BK, Cheku B, et al. Prevalence of genotypic drug resistance among a cohort of HIV-infected newborns. Journal of acquired immune deficiency syndromes (1999). 2003;32(3):292-7. Epub 2003/03/11. PubMed PMID: 12626889.

55. Phiri K, Hernandez-Diaz S, Dugan KB, Williams PL, Dudley JA, Jules A, et al. First trimester exposure to antiretroviral therapy and risk of birth defects. The Pediatric infectious disease journal. 2014;33(7):741.

56. Read JS, Samuel N, Parameshwari S, Dharmarajan S, Van Hook HM, Jacob SM, et al. Safety of HIV-1 Perinatal Transmission Prophylaxis With Zidovudine and Nevirapine in Rural South India. Journal of the International Association of Physicians in AIDS Care (JIAPAC). 2007;6(2):125-36.

57. Rutstein RM, Volkman KM, Bonda S, Shah SS. Ongoing Maternal-Child Transmission of HIV in an Urban Area, 2003–2012. Hospital pediatrics. 2015;5(2):92-5.

58. Santini-Oliveira M, Friedman RK, Veloso VG, Cunha CB, Pilotto JH, Marins LMS, et al. Incidence of antiretroviral adverse drug reactions in pregnant women in two referral centers for HIV prevention of mother-to-child-transmission care and research in Rio de Janeiro, Brazil. The Brazilian Journal of Infectious Diseases. 2014;18(4):372-8.

59. Schulte J, Dominguez K, Sukalac T, Bohannon B, Fowler MG. Declines in low birth weight and preterm birth among infants who were born to HIV-infected women during an era of increased use of maternal antiretroviral drugs: pediatric spectrum of HIV disease, 1989–2004. Pediatrics. 2007;119(4):e900-e6.

60. Short C, Douglas M, Smith J, Taylor G. Preterm delivery risk in women initiating antiretroviral therapy to prevent HIV mother‐to‐child transmission. HIV medicine. 2014;15(4):233-8.

61. Simonds RJ, Steketee R, Nesheim S, Matheson P, Palumbo P, Alger L, et al. Impact of zidovudine use on risk and risk factors for perinatal transmission of HIV. AIDS (London, England). 1998;12(3):301-8.

62. Sinha G, Choi TJ, Nayak U, Gupta A, Nair S, Gupte N, et al. Clinically significant anemia in HIV-infected pregnant women in India is not a major barrier to zidovudine use for prevention of maternal-to-child transmission. JAIDS Journal of Acquired Immune Deficiency Syndromes. 2007;45(2):210-7.

63. Soler-Palacín P, Martín-Nalda A, Martínez-Gómez X, Melendo S, Riudor E, Arranz JA, et al. Hyperlactatemia and in utero exposure to antiretrovirals: is the control group the clue? AIDS research and human retroviruses. 2012;28(8):752-8.

64. Torpey K, Mandala J, Kasonde P, Bryan-Mofya G, Bweupe M, Mukundu J, et al. Analysis of HIV early infant diagnosis data to estimate rates of perinatal HIV transmission in Zambia. PloS one. 2012;7(8):e42859.

65. Ugochukwu EF, Kalu SO. Early infant diagnosis of HIV infection in southeastern Nigeria: prevalence of HIV infection among HIV-exposed babies. West African journal of medicine. 2010;29(1):3-7. Epub 2010/05/25. PubMed PMID: 20496330.

66. Viani RM, Ruiz-Calderon J, Lopez G, Chacon-Cruz E, Spector SA. Mother-to-child HIV transmission in a cohort of pregnant women diagnosed by rapid HIV testing at Tijuana General Hospital, Baja California, Mexico. Journal of the International Association of Physicians in AIDS Care (Chicago, Ill : 2002). 2010;9(2):82-6. Epub 2010/03/26. doi: 10.1177/1545109710363920. PubMed PMID: 20335496.

67. Watts DH, Li D, Handelsman E, Tilson H, Paul M, Foca M, et al. Assessment of birth defects according to maternal therapy among infants in the Women and Infants Transmission Study. JAIDS Journal of Acquired Immune Deficiency Syndromes. 2007;44(3):299-305.

68. Ziske J, Kunz A, Sewangi J, Lau I, Dugange F, Hauser A, et al. Hematological changes in women and infants exposed to an AZT-containing regimen for prevention of mother-to-child-transmission of HIV in Tanzania. PloS one. 2013;8(2):e55633.

69. Zuccotti G, Ferraris G, Agostoni C, Rancilio L, Ravizza M, Bucceri A, et al. Zidovudine prophylaxis and perinatal HIV-1 transmission. Acta Paediatrica. 1999;88(11):1298-300.

70. Zuk DM, Hughes CA, Foisy MM, Robinson JL, Singh AE, Houston S. Adverse effects of antiretrovirals in HIV-infected pregnant women. The Annals of pharmacotherapy. 2009;43(6):1028-35. Epub 2009/06/06. doi: 10.1345/aph.1L689. PubMed PMID: 19491318.

71. Prieto LM, González-Tomé MI, Muñoz E, Fernández-Ibieta M, Soto B, Álvarez A, et al. Birth defects in a cohort of infants born to HIV-infected women in Spain, 2000-2009. BMC infectious diseases. 2014;14(1):1.

72. Vannappagari V, Albano J, Tilson H, Gee C, Gandhi A, Koram N, et al., editors. Zidovudine Exposure during Pregnancy and Birth Outcomes: Data from the Antiretroviral Pregnancy Registry. Pharmacoepidemiology and Drug Safety; 2013: WILEY-BLACKWELL 111 RIVER ST, HOBOKEN 07030-5774, NJ USA.

73. Chung MH, Kiarie JN, Richardson BA, Lehman DA, Overbaugh J, John-Stewart GC. Breast milk HIV-1 suppression and decreased transmission: a randomized trial comparing HIVNET 012 nevirapine versus short-course zidovudine. AIDS (London, England). 2005;19(13):1415.

74. Dabis F, Msellati P, Meda N, Welffens-Ekra C, You B, Manigart O, et al. 6-month efficacy, tolerance, and acceptability of a short regimen of oral zidovudine to reduce vertical transmission of HIV in breastfed children in Côte d'Ivoire and Burkina Faso: a double-blind placebo-controlled multicentre trial. The Lancet. 1999;353(9155):786-92.

75. Dorenbaum A, Cunningham CK, Gelber RD, Culnane M, Mofenson L, Britto P, et al. Two-dose intrapartum/newborn nevirapine and standard antiretroviral therapy to reduce perinatal HIV transmission: a randomized trial. Jama. 2002;288(2):189-98.

76. Gray G, Violari A, McIntyre J, Jivkov B, Schnittman S, Reynolds L, et al. Antiviral activity of nucleoside analogues during short-course monotherapy or dual therapy: its role in preventing HIV infection in infants. Journal of acquired immune deficiency syndromes (1999). 2006;42(2):169-76. Epub 2006/04/28. doi: 10.1097/01.qai.0000219772.74432.20. PubMed PMID: 16639342.

77. Guay LA, Musoke P, Fleming T, Bagenda D, Allen M, Nakabiito C, et al. Intrapartum and neonatal single-dose nevirapine compared with zidovudine for prevention of mother-to-child transmission of HIV-1 in Kampala, Uganda: HIVNET 012 randomised trial. Lancet. 1999;354(9181):795-802. Epub 1999/09/15. doi: 10.1016/s0140-6736(99)80008-7. PubMed PMID: 10485720.

78. Jackson JB, Musoke P, Fleming T, Guay LA, Bagenda D, Allen M, et al. Intrapartum and neonatal single-dose nevirapine compared with zidovudine for prevention of mother-to-child transmission of HIV-1 in Kampala, Uganda: 18-month follow-up of the HIVNET 012 randomised trial. Lancet. 2003;362(9387):859-68. doi: 10.1016/S0140-6736(03)14341-3. PubMed PMID: 13678973.

79. Kiarie JN, Kreiss JK, Richardson BA, John-Stewart GC. Compliance with antiretroviral regimens to prevent perinatal HIV-1 transmission in Kenya. AIDS (London, England). 2003;17(1):65-71. Epub 2002/12/13. doi: 10.1097/01.aids.0000042938.55529.e1. PubMed PMID: 12478070; PubMed Central PMCID: PMCPmc3387271.

80. Koss CA, Natureeba P, Plenty A, Luwedde F, Mwesigwa J, Ades V, et al. Risk factors for preterm birth among HIV-infected pregnant Ugandan women randomized to lopinavir/ritonavir-or efavirenz-based antiretroviral therapy. Journal of acquired immune deficiency syndromes (1999). 2014;67(2):128-35.

81. Lambert JS, Watts DH, Mofenson L, Stiehm ER, Harris DR, Bethel J, et al. Risk factors for preterm birth, low birth weight, and intrauterine growth retardation in infants born to HIV-infected pregnant women receiving zidovudine. Pediatric AIDS Clinical Trials Group 185 Team. AIDS (London, England). 2000;14(10):1389-99. Epub 2000/08/10. PubMed PMID: 10930154.

82. Limpongsanurak S, Thaithumyanon P, Chaithongwongwatthana S, Thisyakorn U, Ruxrungtham K, Kongsin P, et al. Short course zidovudine maternal treatment in HIV-1 vertical transmission: randomized controlled multicenter trial. Journal of the Medical Association of Thailand = Chotmaihet thangphaet. 2001;84 Suppl 1:S338-45. Epub 2001/09/01. PubMed PMID: 11529355.

83. Shaffer N, Chuachoowong R, Mock PA, Bhadrakom C, Siriwasin W, Young NL, et al. Short-course zidovudine for perinatal HIV-1 transmission in Bangkok, Thailand: a randomised controlled trial. The Lancet. 1999;353(9155):773-80.

84. Shapiro R, Hughes M, Ogwu A, Kitch D, Lockman S, Moffat C, et al. Antiretroviral regimens in pregnancy and breast-feeding in Botswana. New England Journal of Medicine. 2010;362(24):2282-94.

85. Sperling RS, Shapiro DE, McSherry GD, Britto P, Cunningham BE, Culnane M, et al. Safety of the maternal-infant zidovudine regimen utilized in the Pediatric AIDS Clinical Trial Group 076 Study. AIDS (London, England). 1998;12(14):1805-13. Epub 1998/10/29. PubMed PMID: 9792381.

86. The Kesho Bora Study Group. Triple antiretroviral compared with zidovudine and single-dose nevirapine prophylaxis during pregnancy and breastfeeding for prevention of mother-to-child transmission of HIV-1 (Kesho Bora study): a randomised controlled trial. The Lancet infectious diseases. 2011;11(3):171-80.

87. Petra Study Team. Efficacy of three short-course regimens of zidovudine and lamivudine in preventing early and late transmission of HIV-1 from mother to child in Tanzania, South Africa, and Uganda (Petra study): a randomised, double-blind, placebo-controlled trial. The Lancet. 2002;359(9313):1178-86.

88. Tubiana R, Mandelbrot L, Le Chenadec J, Delmas S, Rouzioux C, Hirt D, et al. Lopinavir/ritonavir monotherapy as a nucleoside analogue-sparing strategy to prevent HIV-1 mother-to-child transmission: the ANRS 135 PRIMEVA phase 2/3 randomized trial. Clinical infectious diseases : an official publication of the Infectious Diseases Society of America. 2013;57(6):891-902. Epub 2013/06/15. doi: 10.1093/cid/cit390. PubMed PMID: 23766338.

89. Wiktor SZ, Ekpini E, Karon JM, Nkengasong J, Maurice C, Severin ST, et al. Short-course oral zidovudine for prevention of mother-to-child transmission of HIV-1 in Abidjan, Cote d'Ivoire: a randomised trial. Lancet. 1999;353(9155):781-5. Epub 1999/08/25. doi: 10.1016/s0140-6736(98)10412-9. PubMed PMID: 10459958.

90. Witt KL, Cunningham CK, Patterson KB, Kissling GE, Dertinger SD, Livingston E, et al. Elevated frequencies of micronucleated erythrocytes in infants exposed to zidovudine in utero and postpartum to prevent mother‐to‐child transmission of HIV. Environmental and molecular mutagenesis. 2007;48(3‐4):322-9.

91. Bailey A, Newell M-L, Peckham C, De Rossi A, Ehrnst A, Grosch-Worner I, et al. Maternal viral load and vertical transmission of HIV-1: an important factor but not the only one: the European Collaborative Study. AIDS (London, England). 1999;13:1377-85.

92. Bailey H, Townsend C, Cortina-Borja M, Thorne C. Insufficient antiretroviral therapy in pregnancy: missed opportunities for prevention of mother-to-child transmission of HIV in Europe. Antiviral therapy. 2011;16(6):895-903. Epub 2011/09/09. doi: 10.3851/imp1849. PubMed PMID: 21900722; PubMed Central PMCID: PMCPmc3428867.

93. Beckerman K AJ, Martinez-Tristani M, Seekins D, Storfer S, David N, Vannappagari V, Watts DH, Scheurle A, Tilson H. Preterm Birth (PTB), low birth weight (LBW) and fetal antiretroviral (ARV) exposure: Gestational age (EGA) and birth weight data from 10022 singleton live births (LB) reported to the Antiretroviral Pregnancy Registry (APR) 1989 through 31 January 2009. AIDS 2010 - XVIII International AIDS Conference: Abstract no WEAX01052010.

94. Bollen LJ, Whitehead SJ, Mock PA, Leelawiwat W, Asavapiriyanont S, Chalermchockchareonkit A, et al. Maternal herpes simplex virus type 2 coinfection increases the risk of perinatal HIV transmission: possibility to further decrease transmission? AIDS (London, England). 2008;22(10):1169-76. Epub 2008/06/06. doi: 10.1097/QAD.0b013e3282fec42a. PubMed PMID: 18525263.

95. Boyer PJ, Dillon M, Navaie M, Deveikis A, Keller M, O'Rourke S, et al. Factors predictive of maternal-fetal transmission of HIV-1: preliminary analysis of zidovudine given during pregnancy and/or delivery. Jama. 1994;271(24):1925-30.

96. Briand N, Mandelbrot L, Le Chenadec J, Tubiana R, Teglas JP, Faye A, et al. No relation between in-utero exposure to HAART and intrauterine growth retardation. AIDS (London, England). 2009;23(10):1235-43. Epub 2009/05/09. doi: 10.1097/QAD.0b013e32832be0df. PubMed PMID: 19424054.

97. Briand N, Warszawski J, Mandelbrot L, Dollfus C, Pannier E, Cravello L, et al. Is intrapartum intravenous zidovudine for prevention of mother-to-child HIV-1 transmission still useful in the combination antiretroviral therapy era? Clinical infectious diseases : an official publication of the Infectious Diseases Society of America. 2013;57(6):903-14. Epub 2013/06/04. doi: 10.1093/cid/cit374. PubMed PMID: 23728147.

98. Brogly SB, DiMauro S, Van Dyke RB, Williams PL, Naini A, Libutti DE, et al. Short communication: transplacental nucleoside analogue exposure and mitochondrial parameters in HIV-uninfected children. AIDS research and human retroviruses. 2011;27(7):777-83. Epub 2010/12/15. doi: 10.1089/aid.2010.0204. PubMed PMID: 21142587; PubMed Central PMCID: PMCPmc3159117.

99. Brown Jr Robert GD, Peschell Ken, Zhang Sherry, Fagan Elizabeth. Tenofovir Disoproxil Fumarate-Containing Regimens in Pregnancy: Report From the Antiretroviral Pregnancy Registry. 60th Annual Meeting of the American Association for the Study of Liver Diseases; October 30 - November 3, 2009; Boston, Massachusetts, USA2009.

100. Canada Communicable Disease Report. Reduction of HIV transmission from mother to infant. CMAJ: Canadian Medical Association Journal. 1994;151(5):583-8. PubMed PMID: PMC1337193.

101. Centers for Disease Control and Prevention (CDC). Birth outcomes following zidovudine therapy in pregnant women. MMWR Morbidity and mortality weekly report. 1994;43(22):409, 15-6. Epub 1994/06/10. PubMed PMID: 8003111.

102. Chotpitayasunondh T, Vanprapar N, Simonds R, Chokephaibulkit K, Waranawat N, Mock P, et al. Safety of late in utero exposure to zidovudine in infants born to human immunodeficiency virus-infected mothers: Bangkok. Pediatrics. 2001;107(1):e5-e.

103. Chung MH, Kiarie JN, Richardson BA, Lehman DA, Overbaugh J, Kinuthia J, et al. Highly active antiretroviral therapy versus zidovudine/nevirapine effects on early breast milk HIV type-1 Rna: a phase II randomized clinical trial. Antiviral therapy. 2008;13(6):799-807. Epub 2008/10/09. PubMed PMID: 18839781; PubMed Central PMCID: PMCPMC2859833.

104. Connor EM, Sperling RS, Gelber R, Kiselev P, Scott G, O'Sullivan MJ, et al. Reduction of maternal-infant transmission of human immunodeficiency virus type 1 with zidovudine treatment. Pediatric AIDS Clinical Trials Group Protocol 076 Study Group. The New England journal of medicine. 1994;331(18):1173-80. Epub 1994/11/03. doi: 10.1056/nejm199411033311801. PubMed PMID: 7935654.

105. Cooper ER, Charurat M, Mofenson L, Hanson IC, Pitt J, Diaz C, et al. Combination antiretroviral strategies for the treatment of pregnant HIV-1-infected women and prevention of perinatal HIV-1 transmission. Journal of acquired immune deficiency syndromes (1999). 2002;29(5):484-94.

106. Cooper ER, Nugent RP, Diaz C, Pitt J, Hanson C, Kalish LA, et al. After AIDS clinical trial 076: the changing pattern of zidovudine use during pregnancy, and the subsequent reduction in the vertical transmission of human immunodeficiency virus in a cohort of infected women and their infants. Journal of Infectious Diseases. 1996;174(6):1207-11.

107. Cotter AM, Garcia AG, Duthely ML, Luke B, O’Sullivan MJ. Is antiretroviral therapy during pregnancy associated with an increased risk of preterm delivery, low birth weight, or stillbirth? Journal of Infectious Diseases. 2006;193(9):1195-201.

108. Covington DL, Conner SD, Doi PA, Swinson J, Daniels EM. Risk of birth defects associated with nelfinavir exposure during pregnancy. Obstetrics & Gynecology. 2004;103(6):1181-9.

109. Culnane M, Fowler M, Lee SS, McSherry G, Brady M, O'Donnell K, et al. Lack of long-term effects of in utero exposure to zidovudine among uninfected children born to HIV-infected women. Jama. 1999;281(2):151-7.

110. Dabis F, Bequet L, Ekouevi DK, Viho I, Rouet F, Horo A, et al. Field efficacy of zidovudine, lamivudine and single-dose nevirapine to prevent peripartum HIV transmission. AIDS (London, England). 2005;19(3):309.

111. Dabis F, Elenga N, Meda N, Leroy V, Viho I, Manigart O, et al. 18-Month mortality and perinatal exposure to zidovudine in West Africa. AIDS (London, England). 2001;15(6):771-9. Epub 2001/05/24. PubMed PMID: 11371692.

112. de Souza RS, Gómez-Marín O, Scott GB, Guasti S, O'Sullivan MJ, Oliveira RH, et al. Effect of prenatal zidovudine on disease progression in perinatally HIV-1-infected infants. JAIDS Journal of Acquired Immune Deficiency Syndromes. 2000;24(2):154-61.

113. Dryden-Peterson S, Jayeoba O, Hughes MD, Jibril H, Keapoletswe K, Tlale J, et al. Highly active antiretroviral therapy versus zidovudine for prevention of mother-to-child transmission in a programmatic setting, Botswana. Journal of acquired immune deficiency syndromes (1999). 2011;58(3):353-7. Epub 2011/07/28. doi: 10.1097/QAI.0b013e31822d4063. PubMed PMID: 21792062; PubMed Central PMCID: PMCPmc3196679.

114. Ekouevi D, Coffie P, Becquet R, Tonwe-Gold B, Horo A, Thiebaut R, et al. Antiretroviral therapy in pregnant women with advanced HIV disease and pregnancy outcomes in Abidjan, Cote d'Ivoire. AIDS (London, England). 2008;22(14):1815.

115. European Collaborative Study. HIV-infected pregnant women and vertical transmission in Europe since 1986. European collaborative study. AIDS (London, England). 2001;15(6):761-70. Epub 2001/05/24. PubMed PMID: 11371691.

116. European Collaborative Study. Exposure to antiretroviral therapy in utero or early life: the health of uninfected children born to HIV-infected women. Journal of acquired immune deficiency syndromes (1999). 2003;32(4):380.

117. European Collaborative Study. Mother-to-child transmission of HIV infection in the era of highly active antiretroviral therapy. Clinical infectious diseases : an official publication of the Infectious Diseases Society of America. 2005;40(3):458-65. Epub 2005/01/26. doi: 10.1086/427287. PubMed PMID: 15668871.

118. Feiterna-Sperling C, Weizsaecker K, Buhrer C, Casteleyn S, Loui A, Schmitz T, et al. Hematologic effects of maternal antiretroviral therapy and transmission prophylaxis in HIV-1-exposed uninfected newborn infants. Journal of acquired immune deficiency syndromes (1999). 2007;45(1):43-51. Epub 2007/03/16. doi: 10.1097/QAI.0b013e318042d5e3. PubMed PMID: 17356471.

119. Floridia M, Mastroiacovo P, Tamburrini E, Tibaldi C, Todros T, Crepaldi A, et al. Birth defects in a national cohort of pregnant women with HIV infection in Italy, 2001–2011. BJOG: An International Journal of Obstetrics & Gynaecology. 2013;120(12):1466-76.

120. Floridia M, Ravizza M, Masuelli G, Giacomet V, Martinelli P, Degli Antoni A, et al. Atazanavir and lopinavir profile in pregnant women with HIV: tolerability, activity and pregnancy outcomes in an observational national study. Journal of Antimicrobial Chemotherapy. 2014;69(5):1377-84.

121. French CE, Tookey PA, Cortina-Borja M, de Ruiter A, Townsend CL, Thorne C. Influence of short-course antenatal antiretroviral therapy on viral load and mother-to-child transmission in subsequent pregnancies among HIV-infected women. Antiviral therapy. 2013;18(2):183-92. Epub 2013/03/12. doi: 10.3851/imp2327. PubMed PMID: 23475123.

122. Frenkel LM, Wagner LE, 2nd, Demeter LM, Dewhurst S, Coombs RW, Murante BL, et al. Effects of zidovudine use during pregnancy on resistance and vertical transmission of human immunodeficiency virus type 1. Clinical infectious diseases : an official publication of the Infectious Diseases Society of America. 1995;20(5):1321-6. Epub 1995/05/01. PubMed PMID: 7620018.

123. Garcia PM, Kalish LA, Pitt J, Minkoff H, Quinn TC, Burchett SK, et al. Maternal levels of plasma human immunodeficiency virus type 1 RNA and the risk of perinatal transmission. Women and Infants Transmission Study Group. The New England journal of medicine. 1999;341(6):394-402. Epub 1999/08/05. doi: 10.1056/nejm199908053410602. PubMed PMID: 10432324.

124. Giuliano M, Andreotti M, Liotta G, Jere H, Sagno JB, Maulidi M, et al. Maternal antiretroviral therapy for the prevention of mother-to-child transmission of HIV in Malawi: maternal and infant outcomes two years after delivery. PloS one. 2013;8(7):e68950. Epub 2013/07/31. doi: 10.1371/journal.pone.0068950. PubMed PMID: 23894379; PubMed Central PMCID: PMCPmc3716887.

125. Goetghebuer T, Haelterman E, Marvillet I, Barlow P, Hainaut M, Salameh A, et al. Vertical transmission of HIV in Belgium: a 1986-2002 retrospective analysis. European journal of pediatrics. 2009;168(1):79-85. Epub 2008/04/09. doi: 10.1007/s00431-008-0717-y. PubMed PMID: 18392638.

126. Grosch-Woerner I, Puch K, Maier RF, Niehues T, Notheis G, Patel D, et al. Increased rate of prematurity associated with antenatal antiretroviral therapy in a German/Austrian cohort of HIV-1-infected women. HIV medicine. 2008;9(1):6-13. Epub 2008/01/18. doi: 10.1111/j.1468-1293.2008.00520.x. PubMed PMID: 18199167.

127. Group KBS. Eighteen-Month Follow-Up of HIV-1–Infected Mothers and Their Children Enrolled in the Kesho Bora Study Observational Cohorts. JAIDS Journal of Acquired Immune Deficiency Syndromes. 2010;54(5):533-41.

128. Huntington SE, Bansi LK, Thorne C, Anderson J, Newell ML, Taylor GP, et al. Treatment switches during pregnancy among HIV-positive women on antiretroviral therapy at conception. AIDS (London, England). 2011;25(13):1647-55. Epub 2011/06/16. doi: 10.1097/QAD.0b013e32834982af. PubMed PMID: 21673558; PubMed Central PMCID: PMCPmc3428898.

129. Ikechebelu JI, Ugboaja JO, Kalu SO, Ugochukwu EF. The outcome of prevention of mother to child transmission (PMTCT) of HIV infection programme in Nnewi, southeast Nigeria. Nigerian journal of medicine : journal of the National Association of Resident Doctors of Nigeria. 2011;20(4):421-5. Epub 2012/02/01. PubMed PMID: 22288315.

130. Italian Register for HIV Infection in Children. Rapid disease progression in HIV-1 perinatally infected children born to mothers receiving zidovudine monotherapy during pregnancy. AIDS (London, England). 1999;13(8):927-33.

131. Jamieson DJ, Sibailly TS, Sadek R, Roels TH, Ekpini ER, Boni-Ouattara E, et al. HIV-1 viral load and other risk factors for mother-to-child transmission of HIV-1 in a breast-feeding population in Cote d'Ivoire. Journal of acquired immune deficiency syndromes (1999). 2003;34(4):430-6. Epub 2003/11/15. PubMed PMID: 14615662.

132. Jungmann EM, Mercey D, DeRuiter A, Edwards S, Donoghue S, Booth T, et al. Is first trimester exposure to the combination of antiretroviral therapy and folate antagonists a risk factor for congenital abnormalities? Sexually transmitted infections. 2001;77(6):441-3. Epub 2001/11/21. PubMed PMID: 11714944; PubMed Central PMCID: PMCPmc1744398.

133. Kouanda S, Tougri H, Cisse M, Simpore J, Pietra V, Doulougou B, et al. Impact of maternal HAART on the prevention of mother-to-child transmission of HIV: results of an 18-month follow-up study in Ouagadougou, Burkina Faso. AIDS care. 2010;22(7):843-50. Epub 2010/07/17. doi: 10.1080/09540120903499204. PubMed PMID: 20635248.

134. Kunz A, von Wurmb-Schwark N, Sewangi J, Ziske J, Lau I, Mbezi P, et al. Zidovudine exposure in HIV-1 infected Tanzanian women increases mitochondrial DNA levels in placenta and umbilical cords. PloS one. 2012;7(7):e41637.

135. Landesman SH, Kalish LA, Burns DN, Minkoff H, Fox HE, Zorrilla C, et al. Obstetrical factors and the transmission of human immunodeficiency virus type 1 from mother to child. The Women and Infants Transmission Study. The New England journal of medicine. 1996;334(25):1617-23. Epub 1996/06/20. doi: 10.1056/nejm199606203342501. PubMed PMID: 8628356.

136. Liotta G, Mancinelli S, Nielsen-Saines K, Gennaro E, Scarcella P, Magid NA, et al. Reduction of maternal mortality with highly active antiretroviral therapy in a large cohort of HIV-infected pregnant women in Malawi and Mozambique. PloS one. 2013;8(8):e71653. Epub 2013/08/31. doi: 10.1371/journal.pone.0071653. PubMed PMID: 23990966; PubMed Central PMCID: PMCPmc3747183.

137. Lipshultz SE, Shearer WT, Thompson B, Rich KC, Cheng I, Orav EJ, et al. Cardiac effects of antiretroviral therapy in HIV-negative infants born to HIV-positive mothers: NHLBI CHAART-1 (National Heart, Lung, and Blood Institute Cardiovascular Status of HAART Therapy in HIV-Exposed Infants and Children cohort study). Journal of the American College of Cardiology. 2011;57(1):76-85. Epub 2010/12/28. doi: 10.1016/j.jacc.2010.08.620. PubMed PMID: 21185505; PubMed Central PMCID: PMCPmc3243620.

138. Machado ES, Hofer CB, Costa TT, Nogueira SA, Oliveira RH, Abreu TF, et al. Pregnancy outcome in women infected with HIV-1 receiving combination antiretroviral therapy before versus after conception. Sexually transmitted infections. 2009;85(2):82-7. Epub 2008/11/07. doi: 10.1136/sti.2008.032300. PubMed PMID: 18987014; PubMed Central PMCID: PMCPmc2864649.

139. Mandelbrot L, Le Chenadec J, Berrebi A, Bongain A, Benifla JL, Delfraissy JF, et al. Perinatal HIV-1 transmission: interaction between zidovudine prophylaxis and mode of delivery in the French Perinatal Cohort. Jama. 1998;280(1):55-60. Epub 1998/07/11. PubMed PMID: 9660364.

140. Mandelbrot L, Mazy F, Floch-Tudal C, Meier F, Azria E, Crenn-Hebert C, et al. Atazanavir in pregnancy: impact on neonatal hyperbilirubinemia. European Journal of Obstetrics & Gynecology and Reproductive Biology. 2011;157(1):18-21.

141. Marsit CJ, Brummel SS, Kacanek D, Seage III GR, Spector SA, Armstrong DA, et al. Infant peripheral blood repetitive element hypomethylation associated with antiretroviral therapy in utero. Epigenetics. 2015;10(8):708-16.

142. Martin F, Taylor GP. Increased rates of preterm delivery are associated with the initiation of highly active antiretrovial therapy during pregnancy: a single-center cohort study. The Journal of infectious diseases. 2007;196(4):558-61. Epub 2007/07/13. doi: 10.1086/519848. PubMed PMID: 17624841.

143. Martinelli P, Agangi A, Sansone M, Maruotti GM, Buffolano W, Paladini D, et al. Epidemiological and clinical features of pregnant women with HIV: a 21-year perspective from a highly specialized regional center in southern Italy. HIV clinical trials. 2008;9(1):36-42.

144. McConnell M, Bakaki P, Eure C, Mubiru M, Bagenda D, Downing R, et al. Effectiveness of repeat single-dose nevirapine for prevention of mother-to-child transmission of HIV-1 in repeat pregnancies in Uganda. Journal of acquired immune deficiency syndromes (1999). 2007;46(3):291-6. Epub 2008/01/03. PubMed PMID: 18167645.

145. McSherry GD, Shapiro DE, Coombs RW, McGrath N, Frenkel LM, Britto P, et al. The effects of zidovudine in the subset of infants infected with human immunodeficiency virus type-1 (Pediatric AIDS Clinical Trials Group Protocol 076). The Journal of pediatrics. 1999;134(6):717-24. Epub 1999/06/04. PubMed PMID: 10356140.

146. Owor M, Mwatha A, Donnell D, Musoke P, Mmiro F, Allen M, et al. Long Term Follow-up of Children in the HIVNET 012 Perinatal HIV Prevention Trial: Five-Year Growth and Survival. Journal of acquired immune deficiency syndromes (1999). 2013;64(5):464.

147. Pacheco SE, McIntosh K, Lu M, Mofenson LM, Diaz C, Foca M, et al. Effect of perinatal antiretroviral drug exposure on hematologic values in HIV-uninfected children: An analysis of the women and infants transmission study. Journal of Infectious Diseases. 2006;194(8):1089-97.

148. Peixoto MF, Pilotto JH, Stoszek SK, Kreitchmann R, Mussi-Pinhata MM, Melo VH, et al. Lopinavir/ritonavir dosing during pregnancy in Brazil and maternal/infant laboratory abnormalities. Brazilian Journal of Infectious Diseases. 2011;15(3):253-61.

149. Phiri K, Williams PL, Dugan KB, Fischer MA, Cooper WO, Seage III GR, et al. Antiretroviral Therapy Use During Pregnancy and the Risk of Small for Gestational Age Birth in a Medicaid Population. The Pediatric infectious disease journal. 2015;34(7):e169-e75.

150. Pinnetti C, Baroncelli S, Molinari A, Nardini G, Genovese O, Ricerca BM, et al. Common occurrence of anaemia at the end of pregnancy following exposure to zidovudine-free regimens. Journal of Infection. 2011;63(2):144-50.

151. Powis KM, Kitch D, Ogwu A, Hughes MD, Lockman S, Leidner J, et al. Increased risk of preterm delivery among HIV-infected women randomized to protease versus nucleoside reverse transcriptase inhibitor-based HAART during pregnancy. Journal of Infectious Diseases. 2011;204(4):506-14.

152. Roberts SS, Martinez M, Covington DL, Rode RA, Pasley MV, Woodward WC. Lopinavir/ritonavir in pregnancy. JAIDS Journal of Acquired Immune Deficiency Syndromes. 2009;51(4):456-61.

153. Shapiro DE, Sperling RS, Mandelbrot L, Britto P, Cunningham BE. Risk factors for perinatal human immunodeficiency virus transmission in patients receiving zidovudine prophylaxis. Obstetrics & Gynecology. 1999;94(6):897-908.

154. Shapiro RL, Kitch D, Ogwu A, Hughes MD, Lockman S, Powis K, et al. HIV transmission and 24-month survival in a randomized trial of HAART to prevent MTCT during pregnancy and breastfeeding in Botswana (The Mma Bana Study). AIDS (London, England). 2013;27(12):1911.

155. Shapiro RL, Souda S, Parekh N, Binda K, Kayembe M, Lockman S, et al. High prevalence of hypertension and placental insufficiency, but no in utero HIV transmission, among women on HAART with stillbirths in Botswana. PloS one. 2012;7(2):e31580.

156. Shapiro RL, Thior I, Gilbert PB, Lockman S, Wester C, Smeaton LM, et al. Maternal single-dose nevirapine versus placebo as part of an antiretroviral strategy to prevent mother-to-child HIV transmission in Botswana. AIDS (London, England). 2006;20(9):1281-8.

157. Siberry GK, Williams PL, Mendez H, SEAGE III GR, Jacobson DL, Hazra R, et al. Safety of tenofovir use during pregnancy: early growth outcomes in HIV-exposed uninfected infants. AIDS (London, England). 2012;26(9):1151.

158. Sibiude J, Le Chenadec J, Bonnet D, Tubiana R, Faye A, Dollfus C, et al. In utero exposure to zidovudine and heart anomalies in the ANRS French perinatal cohort and the nested PRIMEVA randomized trial. Clinical Infectious Diseases. 2015;61(2):270-80.

159. Sibiude J, Mandelbrot L, Blanche S, Le Chenadec J, Boullag-Bonnet N, Faye A, et al. Association between prenatal exposure to antiretroviral therapy and birth defects: an analysis of the French perinatal cohort study (ANRS CO1/CO11). PLoS Med. 2014;11(4):e1001635.

160. Sibiude J, Warszawski J, Tubiana R, Dollfus C, Faye A, Rouzioux C, et al. Premature delivery in HIV-infected women starting protease inhibitor therapy during pregnancy: role of the ritonavir boost? Clinical infectious diseases : an official publication of the Infectious Diseases Society of America. 2012;54(9):1348-60. Epub 2012/03/31. doi: 10.1093/cid/cis198. PubMed PMID: 22460969.

161. Simon T, Funke A-M, Hero B, Reiser-Hartwig S, Fuhrmann U. Effektivität und Nebenwirkungen der antiretroviralen Therapie bei HIV infizierten Schwangeren. Zentralblatt für Gynäkologie. 2002;124(8-9):413-7.

162. Sutthent R, Chokephaibulkit K, Piyasujabul D, Vanprapa N, Roogpisuthipong A, Chaisilwatana P. Effect of perinatal short-course zidovudine on the clinical and virological manifestations of HIV-1 subtype E infection in infants. Journal of clinical virology. 2002;25(1):47-56.

163. Suy A, Hernandez S, Thorne C, Lonca M, Lopez M, Coll O. Current guidelines on management of HIV-infected pregnant women: impact on mode of delivery. European Journal of Obstetrics & Gynecology and Reproductive Biology. 2008;139(2):127-32.

164. Suy A, Martínez E, Coll O, Lonca M, Palacio M, de Lazzari E, et al. Increased risk of pre-eclampsia and fetal death in HIV-infected pregnant women receiving highly active antiretroviral therapy. AIDS (London, England). 2006;20(1):59-66.

165. Szyld EG, Warley EM, Freimanis L, Gonin R, Cahn PE, Calvet GA, et al. Maternal antiretroviral drugs during pregnancy and infant low birth weight and preterm birth. AIDS (London, England). 2006;20(18):2345-53.

166. Timmermans S, Tempelman C, Godfried MH, Nellen J, Dieleman J, Sprenger H, et al. Nelfinavir and nevirapine side effects during pregnancy. AIDS (London, England). 2005;19(8):795-9.

167. Tonwe-Gold B, Ekouevi DK, Viho I, Amani-Bosse C, Toure S, Coffie PA, et al. Antiretroviral treatment and prevention of peripartum and postnatal HIV transmission in West Africa: evaluation of a two-tiered approach. PLoS medicine. 2007;4(8):e257.

168. Townsend C, Cortina‐Borja M, Peckham C, Tookey P. Trends in management and outcome of pregnancies in HIV‐infected women in the UK and Ireland, 1990–2006. BJOG: An International Journal of Obstetrics & Gynaecology. 2008;115(9):1078-86.

169. Townsend CL, Cortina-Borja M, Peckham CS, Tookey PA. Antiretroviral therapy and premature delivery in diagnosed HIV-infected women in the United Kingdom and Ireland. AIDS (London, England). 2007;21(8):1019-26.

170. Townsend CL, Tookey PA, Cortina-Borja M, Peckham CS. Antiretroviral therapy and congenital abnormalities in infants born to HIV-1-infected women in the United Kingdom and Ireland, 1990 to 2003. JAIDS Journal of Acquired Immune Deficiency Syndromes. 2006;42(1):91-4.

171. Townsend CL, Willey BA, Cortina-Borja M, Peckham CS, Tookey PA. Antiretroviral therapy and congenital abnormalities in infants born to HIV-infected women in the UK and Ireland, 1990–2007. AIDS (London, England). 2009;23(4):519-24.

172. van der Merwe K, Hoffman R, Black V, Chersich M, Coovadia A, Rees H. Birth outcomes in South African women receiving highly active antiretroviral therapy: a retrospective observational study. Journal of the International AIDS Society. 2011;14:42. Epub 2011/08/17. doi: 10.1186/1758-2652-14-42. PubMed PMID: 21843356; PubMed Central PMCID: PMCPmc3163172.

173. Vannappagari V, Koram N, Albano J, Tilson H, Gee C. Abacavir and lamivudine exposures during pregnancy and non-defect adverse pregnancy outcomes: data from the Antiretroviral Pregnancy Registry. JAIDS Journal of Acquired Immune Deficiency Syndromes. 2015;68(3):359-64.

174. von Linstow ML, Rosenfeldt V, Lebech AM, Storgaard M, Hornstrup T, Katzenstein TL, et al. Prevention of mother-to-child transmission of HIV in Denmark, 1994-2008. HIV medicine. 2010;11(7):448-56. Epub 2010/02/12. doi: 10.1111/j.1468-1293.2009.00811.x. PubMed PMID: 20146735.

175. Watts DH, Balasubramanian R, Maupin RT, Delke I, Dorenbaum A, Fiore S, et al. Maternal toxicity and pregnancy complications in human immunodeficiency virus–infected women receiving antiretroviral therapy: PACTG 316. American journal of obstetrics and gynecology. 2004;190(2):506-16.

176. Watts DH, Covington DL, Beckerman K, Garcia P, Scheuerle A, Dominguez K, et al. Assessing the risk of birth defects associated with antiretroviral exposure during pregnancy. American journal of obstetrics and gynecology. 2004;191(3):985-92.

177. Watts DH, Huang S, Culnane M, Kaiser KA, Scheuerle A, Mofenson L, et al. Birth defects among a cohort of infants born to HIV-infected women on antiretroviral medication. Journal of perinatal medicine. 2011;39(2):163-70. doi: 10.1515/JPM.2010.139. PubMed PMID: 21142844; PubMed Central PMCID: PMC3068472.

178. Watts DH, Williams PL, Kacanek D, Griner R, Rich K, Hazra R, et al. Combination antiretroviral use and preterm birth. Journal of Infectious Diseases. 2013;207(4):612-21.

179. White A, Eldridge R, Andrews E. Birth outcomes following zidovudine exposure in pregnant women: the Antiretroviral Pregnancy Registry. Acta paediatrica (Oslo, Norway : 1992) Supplement. 1997;421:86-8. Epub 1997/06/01. PubMed PMID: 9240865.

180. Williams PL, Crain MJ, Yildirim C, Hazra R, Van Dyke RB, Rich K, et al. Congenital anomalies and in utero antiretroviral exposure in human immunodeficiency virus–exposed uninfected infants. JAMA pediatrics. 2015;169(1):48-55.
