# Supplementary material for: Comparative safety and effectiveness of perinatal antiretroviral therapies for HIV-infected women and their children: Systematic review and network meta-analysis including different study designs
Source: PLoS One. 2018 Jun 18;13(6):e0198447. doi: 10.1371/journal.pone.0198447 (PMC6005568; doi:10.1371/journal.pone.0198447)
Supplement: S8 Appendix — (DOCX) [file pone.0198447.s008.docx]

# S8 Appendix. Study Characteristics

| **Author, Year** | **Country of Conduct** | **Single or multi-center** | **Setting/Registry Name** | **Sample Size used in Analyses** | **Period of Conduct/Data Collection** |
| --- | --- | --- | --- | --- | --- |
| ***Observational studies (n=72)**** | | | | | |
| Alvarez 2007[1] | USA | single center | University Hospital in Newark, New Jersey | 18 | 2000-2005 |
| Areechokchai 2009[2] | Thailand | single center | Chonburi Hospital, Pattaya, Thailand. | 246 | 2001-2006 |
| Asavapiriyanont, 2011[3] | Thailand | single center | Rajavithi Hospital | 200 | 2004-2008 |
| Bae 2008[4] | Botswana | multi-center | Four sites in Botswana for the prevention of MTCT, cohort within Mashi Study | 178 | 2001-2003 |
| Bailey 2013[5] | Ukraine | multi-center | HIV centres | 3535 | 2008-2010 |
| Barral, 2014[6] | Brazil | multi-center | 32 primary care units (PCU), and two general hospitals. Health care for people living with HIV is offered by a specialized unit at the University Hospital Miguel Riet Correa Junior (HU-FURG). | 236 | 2003-2007 |
| Bellón Cano 2004[7] | Spain | multi-center | Gregorio Maraƒón General University Hospital in Madrid. | 126 | 1997-2000 |
| Bera 2010[8] | South Africa | single center | Efavirenz in Pregnancy Registry/Frere Hospital | 217 | 2006-2008 |
| Blood 2009[9] | USA | single center | Miriam Hospital, Alpert Medical School of Brown University, Providence, US | 8 | 2000-2006 |
| Boer 2006[10] | the Netherlands | multi-center | Academic Medical Center in Amsterdam and Erasmus Medical Center in Rotterdam, the Netherlands | 143 | 1997-2003 |
| Brogly 2010[11] | USA | multi-center | Pediatric AIDS Clinical Trials Group 219 and 219C protocols | 2033 | 1993-2000 |
| Bucceri 2002[12] | Italy | single center | Department of Gynecology and Obstetrics, University of Milan | 89 | 1998-2000 |
| Chansinghakul 2009[13] | Thailand | single center | King Chulalongkorn Memorial Hospital | 176 | 2003-2006 |
| Chen 2012[14] | Botswana | multi center | 6 Government facilities: Princess Marina Hospital, Gaborone; Scottish Livingstone Hospital, Molepolole; Deborah Retief Memorial Hospital, Mochudi; Nyangabgwe Hospital, Francistown; Letsholathebe Memorial Hospital, Maun; Gantsi Primary Hospital, Gantsi. | 9149 | 2009-2011 |
| Chmait 2002[15] | USA | single center | UCSD Mother, Child and Adolescent HIV Program | 62 | 1998-2000 |
| Contu 1995[16] | Italy | multi-center | Clinical institutes in Italy | 76 | 1989-1992 |
| Cotter 2012[17] | USA | single center | University of Miami/ Jackson Memorial Medical Center | 717 | 1996-2008 |
| Darak 2013[18] | India | multi-center | 50 hospital antenatal care clinics in 10 districts of Maharashtra, India | 516 | 2008-2012 |
| de Lemos 2012[19] | Brazil | single center | Nossa Senhora de Lourdes Maternity Hospital (MNSL) in Aracaju, State of Sergipe, Brazil | 110 | 1994-2010 |
| Duran 2006[20] | Argentina | single center | HIV Perinatal clinic in Hospital José María Ramos Mejía in Buenos Aires | 291 | 1994-2003 |
| European Collaborative Study 2006[21] | UK | multi-center | Western European centres in Spain, Italy, the United Kingdom, Germany,Belgium,Sweden, the Netherlands, Poland , Denmark, Ukraine | 4712 | 1985-2004 |
| Ezechi 2012[22] | Nigeria | single center | HIV treatment centre, Nigerian Institute of Medical Research, Lagos | 1626 | 2004-2010 |
| Fiore 2006[23] | Italy | multi-center | Maternity centres | 49 | 2001-2002 |
| Fitzgerald 2010[24] | South Africa | single center | Gugulethu Community Health Centre in Nyanga, CapeTown | 752 | 2002-2008 |
| Floridia 2006[25] | Italy | multi-center | The National Program on Surveillance on Antiretroviral Treatment in Pregnancy (an on-going national surveillance study established in Italy in 2001.) | 60 | 2001-2005 |
| Frenkel 1997[26] | USA | multi-center | HIV-1 obstetric centers | 173 | 1988-1994 |
| Gartland 2013[27] | Zambia | multi-center | Primary care clinics | 270 | 2009-2011 |
| Gibb 2012[28] | Uganda/Zimbabwe | multi-center | DART trial | 366 | 2003-2009 |
| Goldstein 2000[29] | USA | single center | Washington Hospital Centre (DC) | 103 | 1995-1998 |
| Grosch-Woerner 2000[30] | Germany | single center | Charite Virchow-Klinikum, Berlin | 177 | 1985-1999 |
| Habib 2008[31] | Tanzania | single center | Kilimanjaro Christian Medical Centre (KCMC) Hospital in Moshi Urban district | 434 | 1999-2006 |
| Hankin 2009[32] | UK | multi-center | 168 hospitals | 699 | 2002-2005 |
| Hoffman 2010[33] | South Africa | multi-center | 28 Integrated antenatal and antiretroviral clinics in Johannesburg city and surrounding suburbs. Charlotte Maxeke Johannesburg Academic Hospital and Rahima Moosa Mother and Child Hospital | 3003 | 2004-2008 |
| Hussain 2011[34] | South Africa | single center | Prince Mshiyeni Memorial Hospital | 415 | 2010 |
| Joao 2010[35] | Argentina, Brazil | multi-center | Argentinian and Brazilian sites (hospitals) participating in the NISDI Perinatal study. | 995 | NA |
| Leroy 2008[36] | Cote d'Ivoire | multi-center | Unspecified community-run facilities in two urban districts of Abidjan, Cote d'Ivoire. | 730 | 2001-2003 |
| Lin 2005[37] | Taiwan | single center | Tertiary care university hospital Dept of pediatrics, National Cheng Kung University Hospital | 8 | 1993-2003 |
| Lindegren 2000[38] | USA | multi-center | 32 States with HIV reporting and a special HIV surveillance project in Los Angeles County and in 22 hospitals in New York City | 6395 | 1993-1998 |
| Lopez 2012[39] | Spain | single center | University referral hospital, Barcelona | 519 | 1986-2010 |
| Lussiana 2012[40] | Angola | single center | Luanda Municipal Hospital Divina Providencia service for PMTCT, Luanda | 107 | 2007-2011 |
| Mandelbrot 2001[41] | France | multi-center | French Perinatal Cohort study, 85 centres throughout France. | 1362 | NA |
| Mania 2013[42] | Poland | single center | Department of Infectious Diseases and Child Neurology, University of Medical Sciences, Poznan | 35 | 2003-2010 |
| Marazzi 2011[43] | Malawi, Mozambique | multi-center | Drug Enhancement Against AIDS and Malnutrition (DREAM) centers. | 3273 | 2005-2009 |
| Marczynska 2000[44] | Poland | single center | Department of Children's Infectious Diseases (a pediatric HIV referral centre) at the Medical University, Warsaw, Poland. | 92 | 1989-1999 |
| Matheson 1995[45] | USA | multi-center | Patients from NYC | 321 | 1986-1993 |
| Mazur-Melewska 2005[46] | Poland | single center | Department of Infectious Diseases and Child Neurology in Poznan, Poland. | 28 | 1992-2004 |
| McGowan 1999[47] | USA | single center | Bronx-Lebanon Hospital (New York) | 26 | 1996-1998 |
| Meyer 2014[48] | Malawi | multi-center | Two rural health clinics outside Blantyre,  Malawi | 69 | 2009-2010 |
| Money 2007[49] | Canada | multi-center | Provincial (British Columbia) HIV perinatal database. | 294 | 1995-2005 |
| Msellati 2001[50] | Cote d'Ivoire | multi-center | 4 Health care centres (Yopougon Teaching Hospital; a public sector health centre; 2 community-based health centres, Abidjan. | 89 | 1998-1999 |
| Mussi-Pinhata 2003[51] | Brazil | single center | University Hospital of the Faculty of Medicine of Ribeirao Preto, University of Sao Paulo (HCFMRP-USP) | 389 | 1988-1999 |
| Onakewhor 2011[52] | Nigeria | single center | Antenatal clinic, University of Benin Teaching Hospital | 249 | 2008-2009 |
| Parker 2003[53] | USA | single center | The Pediatric HIV Testing Service of the New York State Department of Health (NYSDOH). Patients from New York State. | 87 | 1998-1999 |
| Phiri 2014[54] | USA | multi-center | Tennessee Medicaid data linked to vital records | 806 | 1994-2009 |
| Prieto 2014[55] | Spain | multi-center | Madrid Cohort of HIV Infected Mother Infant Pairs; Birth defects (BDs) were registered according to European Surveillance of Congenital Anomalies (EUROCAT). | 820 | 2000-2009 |
| Read 2007[56] | India | multi-center | Two hospitals in Rural south India (Tamil Nadu): Namakkal District Hospital; Rasipuram Government Hospital | 60 | NA |
| Rutstein 2014[57] | USA | single center | Clinic, an urban referral site for  HIV-exposed and -infected children. | 505 | 2003-2012 |
| Santini-Oliveira 2014[58] | Brazil | multi-center | Hospital Federaldos Servidores do Estado (HFSE) and Hospital Geral de NovaIguac¸u (HGNI), both located at Rio de Janeiro State, Brazil | 214 | 2005-2006 |
| Schulte 2007[59] | USA | multi-center | Chart abstractions carried out at 8 US geographic sites participating in the Pediatric Spectrum of Disease study. | 8793 | 1989-2004 |
| Short 2014[60] | UK | single center | St. Mary's Hospital, London | 331 | 1996-2010 |
| Simonds 1998[61] | USA | multi-center | Perinatal AIDS Collaborative Transmission Studies (PACTS). A collaboration of 4 CDC funded institutions (Atlanta, Baltimore, New York City, Newark) | 1032 | 1985-1997 |
| Sinha 2007[62] | India | single center | Sassoon General Hospital, Pune | 398 | 2002-2006 |
| Soler-Palacin 2012[63] | Spain | single center | Pediatric referral hospital in Barcelona (Spain). Pediatric Infectious Diseases and Immunodeficiencies Unit, Vall d'Hebron University Hospital | 19 | 2004-2007 |
| St. John 2003[64] | Barbados | single center | Queen Elizabeth Hospital which is where HIV-infected pregnant women once diagnosed are referred from regional polyclinics for follow-up and care by consultant obstetricians. | 167 | NA |
| Torpey 2012[65] | Zambia | multi-center | The Zambian Prevention Care and Treatment Project (ZPCT) and its successor project, ZPCT II, cover 5 of 9 provinces in Zambia: Central, Copperbelt, Luapula, North-Western, and Northern Provinces. This project supports the continuum of care for PMTCT: from HIV testing of pregnant women to early diagnosis of HIV among children born to HIV-positive mothers. | 5533 | 2007-2010 |
| Vannappagari 2013[66] | USA | multi-center | Antiretroviral Pregnancy Registry | 13537 | 1989-2012 |
| Ugochukwu 2009[67] | Nigeria | single center | Primary and secondary health facilities in Anambra state, Southern Nigeria and beyond, PMTCT sites in mission and private hospitals in Anambra, referrals from in Nnamdi Azikwe University Teaching Hospital (NAUTH) NAUTH labour ward and Special Care Baby Unit and its outstation comprehensive health centres in Umunya, Upko, Neni and Oba, Anambra State. Testing carried out at NAUTH, Nnewi | 96 | NA |
| Viani 2010[68] | Mexico | single center | Tijuana General Hospital, Mexico | 62 | 2003-2005 |
| Watts 2007[69] | USA, Puerto Rico | multi-center | Women and Infants Transmission Study (WITS) carried out at sites in Chicago, Houston, Boston, Worcester, New York City, Brooklyn, San Juan | 2868 | 1990-2004 |
| Witt 2007[70] | USA | multi-center | Duke University Medical Centre (DUMC), University of North Carolina | 10 | NA |
| Ziske 2013[71] | Tanzania | single center | Kyela District Hospital (KDH) in Mbeya Region, Tanzania | 99 | 2008-2009 |
| Zucotti 1999[72] | Italy | multi-center | Department of pediatrics and Deptartment of Obstetrics and Gynecology, San Paolo Hospital, Neonatal Intensive Care Unit, Mangiagalli Hospital, Milan | 236 | 1993-1997 |
| Zuk 2009[73] | Canada | single center | Patients from the Northern Alberta HIV program database. | 144 | 1997-2006 |
| ***Randomised Clinical Trials (n=17)*** | | | | | |
| Chung 2005[74] | Kenya | single center | Mathare North City Council Clinic in Nairobi | 60 | 2003 |
| Dabis 1999[75] | Cote d'Ivoire, Burkina Faso, | multi-center | Public clinics of Abidjan and Bobo Dioulasso | 414 | 1995-1998 |
| Dorenbaum 2002[76] | USA, Brazil, Bahamas | multi-center | Clinical sites providing care for HIV infection | 1248 | 1997-2000 |
| Gray 2006[77] | South Africa | single center | Chris Hani Baragwanath Hospital, Soweto, South Africa | 372 | 1999-2000 |
| Guay 1999[78] | Uganda | single center | General antenatal clinics. Mulago Hospital, Kampala | 650 | NA |
| Jackson 2003[79] | Uganda | single center | Mulago Hospital, Kampala | 647 | 1997-2001 |
| Kiarie 2003[80] | Kenya | single center | Tertiary hospital antenatal clinic, Nairobi. | 128 | NA |
| Koss 2014[81] | Uganda | single center | Secondary analysis of the PROMOTE-Pregnant Women and Infants Study (NCT00993031) | 389 | 2009-2012 |
| Lambert 2000[82] | USA, Puerto Rico | multi-center | 53 Clinical sites in Mainland USA and Puerto Rico | 497 | NA |
| Limpongsanurak 2001[83] | Thailand | multi-center | 3 Tertiary care government hospitals: King Chulalongkorn Memorial hospital, Vajira Hospital and Taksin Hospital, Bangkok | 182 | 1995-1996 |
| Shaffer 1999[84] | Thailand | multi-center | Siriraj and Rajavithi Hospitals, Bangkok, Thailand. | 395 | 1996-1997 |
| Shapiro 2010[85] | Botswana | multi-center | Study locations in Gaborone, Lobatse, Molepole, and Mochudi, Southern, Botswana | 709 | NA |
| Sperling 1998[86] | USA, France | multi-centre | Fifty-five centers in the United States and France | 417 | 1991-1994 |
| The Kesho Bora Study Group 2011[87] | Burkina Faso, Kenya, South Africa | multi-center | 5 Study sites: Centre Muraz, Bobo-Dioulasso, International Centre for Reproductive Health, Mombasa, Kenyatta National Hospital, Nairobi, University of Kwa-Zulu-Natal, Somkhele. | 813 | NA |
| The Petra Study Team 2002[88] | South Africa, Tanzania, Uganda | multi-center | South Africa: 2 large public hospitals (Chris Hani Baragwanath Hospital Johannesburg & King Edward VII Hospital, Durban); Tanzania: 1 Large public hospital (Muhimbili General Hospital, Dar es Salaam); Uganda 1 large public hospital (Mulago Hospital, Kampala) | 1501 | NA |
| Tubiana 2013[89] | France | multi-center | 19 Sites in mainland France | 105 | 2007-2010 |
| Wiktor 1999[90] | Ivory Coast, Multi-country | single center | Koumassi clinic, Abidjan. A large public, mother-child health centre | 273 | 1996-1998 |
| **Notes:** *Includes 72 cohorts and 1 case control study (Witt 2007)  **Abbreviations:** NA,; Not applicable | | | | | |
