# Supplementary material for: Comparative safety and effectiveness of perinatal antiretroviral therapies for HIV-infected women and their children: Systematic review and network meta-analysis including different study designs
Source: PLoS One. 2018 Jun 18;13(6):e0198447. doi: 10.1371/journal.pone.0198447 (PMC6005568; doi:10.1371/journal.pone.0198447)
Supplement: S9 Appendix — (DOCX) [file pone.0198447.s009.docx]

# S9 Appendix. Patient Characteristics

| **Author, Year*** | **Study Design** | **HIV-1 stage** | **Overall # of women (ITT)** | **Mean or Median age (SD or range)†** | **Potential Risk Factors‡** | **LMIC** | **% of patients with antenatal care** | **MP Outcomes; CR Outcomes (included in analyses)** |
| --- | --- | --- | --- | --- | --- | --- | --- | --- |
| Alvarez 2007[1] | cohort | NR | 18 | NR (NR) | 11.11 % of patients with low CD4+ count (<200) | No | 56 | MTCT |
| Areechokchai 2009[2] | cohort | CDC: A, B, C | 246 | Median: 27 (16-41) | 13.3 % of patients with low CD4+ count (<200) | No | 100 | Preterm |
| Asavapiriyanont 2011[3] | cohort | NR | 416 | NR (NR) | NR | No | NR | LBW and SGA |
| Bae 2008[4] | cohort | NR | 178 | NR (NR) | 100 % of patients with low CD4+ count (<200) | Yes | 100 | CM, Preterm |
| Bailey 2013[6] | cohort | WHO: 3, 4 (14%) | 3535 | NR (NR) | 15 % of patients using illicit drugs, 11 % of patients with low CD4+ count (<200) | Yes | NR | Preterm |
| Barral 2014[7] | cohort | NR | 262 | 27 (6.24) | NR | No | ≥83.5 | LBW and SGA, Preterm |
| Bellón Cano 2004[8] | cohort | NR | 124 | NR (NR) | 43.54 % of patients using illicit drugs, 27 % of patients with low CD4+ count (<200) | No | NR | LBW and SGA, Preterm |
| Blood 2009[9] | cohort | NR | 14 | Median: 31 (16-46) | NR | No | 100 | Preterm |
| Boer 2006[10]; CR:Timmermans 2005[11] | cohort | NR | 143 | Median: 29 (16-42) | 5 % of patients using illicit drugs, 2 % of patients using alcohol, 20% of patients who smoke, 34 % of patients with low CD4+ count (<200) | No | 100 | Preterm |
| Brogly 2010[12]; CR: Culnane 1999[13] | cohort | NR | 2202 | NR (NR) | NR | No | 100 | CM; LBW and SGA, Preterm |
| Bucceri 2002[14] | cohort | NR | 100 | Median: 30 (18-40) | 54 % of patients using illicit drugs, 14 % of patients with low CD4+ count (<200) | No | NR | Still Birth |
| Chansinghakul 2009[15] | cohort | NR | 176 | NR (NR) | 25 % of patients with low CD4+ count (<200) | No | 100 | LBW and SGA, Preterm, Still Birth |
| Chen 2012[16]; CR: Dryden-Peterson 2011[17] | cohort | WHO: 3, 4 | 9504 | Median: 28.44 (NR) | 5.29 % of patients using alcohol, 1.76% of patients who smoke, 12.31 % of patients with low CD4+ count (<200) | Yes | 95.54 | Preterm, Still Birth; Infant and Child, LBW and SGA |
| Chmait 2002[18] | cohort | NR | 64 | NR (NR) | NR | No | 100 | LBW and SGA |
| Contu L 1995[19] | cohort | NR | 76 | NR (NR) | 14.2 % of patients with low CD4+ count (<200) | No | NR | MTCT, Infant and Child, CM, LBW and SGA, Preterm |
| Cotter 2012[20]; CR: Cotter 2006[21] | cohort | NR | 717 | NR (NR) | 10.85 % of patients using illicit drugs, 1.7 % of patients using alcohol, 5.6% of patients who smoke, 13.4 % of patients with low CD4+ count (<200) | No | 100 | MTCT; LBW and SGA, Preterm, Still Birth |
| Darak 2013[22] | cohort | NR | 516 | 25 (4.5) | 11 % of patients with low CD4+ count (<200) | Yes | 100 | LBW and SGA, Preterm, Still Birth |
| de Lemos 2012[23] | cohort | NR | 110 | NR (17-45) - | NR | No | 91.8 | Still Birth |
| Duran 2006[24] | cohort | NR | 351 | 26.8 (17-43) | 17.8 % of patients using illicit drugs, 0.28% of patients with TB co-infection | No | NR | MTCT, CM |
| European Collaborative Study 2006[25]; CR: European Collaborative Study 2003[26], Jungmann 2001[27] | cohort | NR | 5967 | Median: 28.5 (10-47) | 20.27 % of patients using illicit drugs, 14 % of patients with low CD4+ count (<200) | No | 100 | MTCT; CM, LBW and SGA, Preterm |
| Ezechi 2012[28] | cohort | NR | 1626 | NR (NR) | 19 % of patients with low CD4+ count (<200) | Yes | 100 | Preterm |
| Fiore 2006[29] | cohort | NR | 57 | 28.47 (NR) | NR | No | 100 | Preterm |
| Fitzgerald 2010[30] | cohort | WHO: 1, 2, 3, 4 | 367 | Median: 27.5 (15-44) | 9% of patients with TB co-infection | No | 100 | Still Birth |
| Floridia 2006[31]; CR: Martinelli 2008[32] | cohort | NR | 334 | 33.6 (17 - 46) | 23.9 % of patients using illicit drugs | No | NR | Still Birth, CM |
| Frenkel 1997[33]; CR: Frenkel 1995[34] | cohort | NR | 188 | 28.1 (5.5) | 20 % of patients using illicit drugs, 21 % of patients with low CD4+ count (<200) | No | 100 | MTCT; Infant and Child |
| Gartland 2013[35] | cohort | WHO: 3, 4 | 284 | Median: 27.1 (23.8-32.5) | NR | Yes | 100 | Preterm, Still Birth |
| Gibb 2012[36] | cohort | NR | 302 | NR (NR) | 26 % of patients with low CD4+ count (<200) | Yes | 100 | Infant and Child, LBW and SGA, Preterm, Still Birth |
| Goldstein 2000[37] | cohort | NR | 107 | NR (NR) | 18.06 % of patients with low CD4+ count (<200) | No | 100 | LBW and SGA |
| Grosch-Woerner 2000[38]; CR: Grosch-Woerner 2008[39], Simon 2002[40] | cohort | NR | 179 | Median: 27.83 (NR) | 50 % of patients using illicit drugs, 7.6 % of patients with low CD4+ count (<200) | No | 100 | MTCT, CM; LBW and SGA, Preterm |
| Hankin 2009[41]; CR: Townsend 2007[42] | cohort | NR | 704 | 30.5 (NR) | 2.7 % of patients using illicit drugs | No | NR | CM; Infant and Child, Preterm, Still Birth |
| Hoffman 2010[43]; CR: Van der Merwe 2011[44] | cohort | WHO: 4 | 1142 | 30.2 (5) | 3.5 % of patients using alcohol, 3.5% of patients who smoke, 76 % of patients with low CD4+ count (<200) | No | NR | MTCT; LBW and SGA, Preterm |
| Hussain 2011[45] | cohort | NR | 1609 | Median: 24 (SD 6.1) | 48 % of patients with low CD4+ count (<200) | No | 100 | LBW and SGA, Preterm, Still Birth |
| Joao 2010[46]  CR: Szyld 2006[47] | cohort | CDC A, B & C | 995 | NR (NR) | 4.2 % of patients using illicit drugs, 9 % of patients using alcohol, 26.23% of patients who smoke, 12.56 % of patients with low CD4+ count (<200) | No | 100 | CM, Still Birth; LBW and SGA, Preterm |
| Leroy 2008 [49]; CR:[50] | cohort | NR | 808 | Median: 26.04 (22-31) | 16.2 % of patients with low CD4+ count (<200) | Yes | 100 | LBW and SGA, Still Birth |
| Lin 2005[51] | cohort | NR | 6 | NR (NR) | NR | No | NR | Preterm |
| Lopez 2012 [52]; CR: Suy 2006[53] | cohort | NR | 519 | 30.3 (5.8) | 1.9 % of patients using illicit drugs, 54.9% of patients who smoke, 11.2 % of patients with low CD4+ count (<200) | No | 100 | Preterm; Stillbirth |
| Lussiana 2012 [54] | cohort | WHO: 1, 2, 3, 4 | 104 | 29.2 (25-33) | 8.7% of patients with TB co-infection | No | NR | Infant and Child |
| Mandelbrot 2001[55]; CR: Mandelbrot 1998[56], Briand 2013[57] | cohort | NR | 1344 | Median: 29.33 (NR) | 14 % of patients using illicit drugs | No | 100 | CM, Still Birth; LBW and SGA, Preterm, Short length |
| Mania 2013 [58] | cohort | NR | 35 | NR (NR) | NR | No | 77.14 | LBW and SGA, Preterm, Small head |
| Marazzi 2011[59]; CR: Giuliano 2013[60] | cohort | NR | 3273 | Median: 26.4 (23.1-30.4) | NR | Yes | 100 | Still Birth, Preterm; LBW and SGA |
| Marczynska 2000[61] | cohort | NR | 91 | NR (NR) | NR | No | NR | Preterm |
| Matheson 1995[62] | cohort | NR | 432 | NR (NR) | 42 % of patients using illicit drugs, 11.5 % of patients with low CD4+ count (<200) | No | 84.42 | Preterm |
| Mazur-Melewska 2005[63] | cohort | NR | 28 | NR (NR) | NR | No | NR | CM, LBW and SGA, Preterm |
| McGowan 1999[64] | cohort | NR | 30 | Median: 29 (22-40) | 37 % of patients using illicit drugs, 43.33 % of patients with low CD4+ count (<200) | No | 100 | LBW and SGA, Preterm, Still Birth |
| Meyer 2014[65] | cohort | NR | 69 | Median: 27.6 (23-36) | NR | No | NR | LBW and SGA, Preterm |
| Money 2007[66] | cohort | NR | 283 | NR (NR) | 6.6 % of patients with low CD4+ count (<200) | No | NR | LBW and SGA, Preterm |
| Msellati 2001[67] | cohort | NR | 80 | NR (NR) | NR | Yes | NR | Still Birth |
| Mussi-Pinhata 2003[68] | cohort | NR | 386 | NR (NR) | 10.1 % of patients using illicit drugs, 17.74 % of patients using alcohol, 33.43% of patients who smoke | No | 28.4 | Infant and Child, Preterm |
| Onakewhor 2011[69] | cohort | NR | 249 | NR (NR) | NR | Yes | 82.3 | Infant and Child, LBW and SGA, Preterm, Still Birth |
| Parker 2003[70] | cohort | NR | 120 | NR (NR) | NR | No | NR | LBW and SGA |
| Prieto 2014[71] | cohort | NR | 872 | 31.36 (NR) | 9.87 % of patients using illicit drugs, 4.89 % of patients using alcohol, 30.10% of patients who smoke, 11.91% of patients with low CD4+ count (<200) | No | NR | CM |
| Read 2007[72] | cohort | NR | 60 | NR (NR) | NR | Yes | 100 | Still Birth |
| Rutstein 2014[73] | cohort | NR | 516 | NR (NR) | NR | No | 94 | MTCT |
| Santini-Oliveira 2014[74] | cohort | NR | 214 | Median: 28.12 (23.3-34.8) | 15 % of patients with low CD4+ count (<200) | No | 100 | CM, LBW and SGA, Preterm |
| Schulte 2007[75] | cohort | NR | 11297 | NR (NR) | 33 % of patients using illicit drugs | No | 1989: 48%, 1995: 78%, 2001: 90%, 2004 84% | LBW and SGA, Preterm |
| Short 2014[76] | cohort | NR | 331 | Median: 32.2 (NR) | 0.9 % of patients using illicit drugs, 13% of patients who smoke | No | NR | Still Birth, Preterm |
| Simonds 1998[78] | cohort | CDC: A, B, C | 1366 | Median: 29 (NR) | 16.7 % of patients using illicit drugs, 11 % of patients with low CD4+ count (<200) | No | 70 | LBW and SGA, Preterm |
| Sinha 2007[79] | cohort | NR | 467 | 23.3 (23.1 - 23.5) | 7.6 % of patients with low CD4+ count (<200) | Yes | 100 | LBW and SGA, Preterm, Still Birth |
| Soler-Palacin 2012[80] | cohort | NR | 39 | Median: 30.5 (25.8-35) | 28.2 % of patients using illicit drugs, 7.7 % of patients with low CD4+ count (<200) | No | NR | LBW and SGA, Preterm |
| St. John 2003[81] | cohort | NR | 167 | NR (NR) | NR | No | NR | Still Birth |
| Torpey 2012[82] | cohort | NR | 28330 | NR (NR) | NR | Yes | NR | MTCT |
| Ugochukwu 2009[83] | cohort | NR | 304 | NR (NR) | NR | Yes | NR | MTCT |
| Viani, R, 2010[85] | cohort | WHO: 1, 2, 3 | 62 | 26.3 (NR) | 46.77 % of patients using illicit drugs, 8.06% of patients with TB co-infection | No | 100 | Preterm, Still Birth |
| Watts 2007[86]; CR: Pacheco 2006[87], Cooper 2002[88], Watts 2013[89] | cohort | NR | 2353 | 28.03 (NR) | 29.01 % of patients using illicit drugs, 34.34 % of patients using alcohol, 39.92% of patients who smoke, 10.08 % of patients with low CD4+ count (<200) | No | 73 | Still Birth, CM; Preterm, LBW and SGA |
| Ziske 2013[90] | cohort | NR | 144 | Median: 26.71 (NR) | NR | No | NR | Preterm |
| Zucotti 1999[92]; CR: de Martino 1999[93] | cohort | NR | 238 | NR (NR) | NR | No | NR | MTCT; Preterm |
| Zuk 2009[94] | cohort | NR | 126 | 26 (17-41) | 42 % of patients using illicit drugs, 46 % of patients using alcohol, 65% of patients who smoke | No | NR | LBW and SGA, Preterm |
| Chung 2005[95]; CR: Chung 2008[96] | RCT | NR | 66 | NR (NR) | 10.3 % of patients with low CD4+ count (<200) | Yes | 100 | Infant and Child, LBW and SGA; Stillbirth |
| Dabis 1999[97]; CR: Dabis 2001[98] | RCT | NR | 431 | Median: 24.5 (18-43) | 8.08 % of patients with low CD4+ count (<200) | Yes | 100 | CM, LBW and SGA, Preterm, Still Birth; Infant and Child |
| Dorenbaum 2002[99]; CR: Watts 2004[100], Machado 2009[101] | RCT | NR | 1270 | Median: 27.9 (23.9-32.3) | 12 % of patients with low CD4+ count (<200) | No | 100 | MTCT; Still Birth, LBW and SGA, Preterm |
| Gray 2006[102] | RCT | NR | 373 | 28.3 (5.8) | 1.34% of patients with TB co-infection | No | 100 | CM, Still Birth |
| Guay 1999[103] | RCT | NR | 645 | Medians: 24.5 (21-28) | 16 % of patients with low CD4+ count (<200) | Yes | 100 | LBW and SGA, Still Birth |
| Jackson 2003[104] | RCT | NR | 645 | Median: 24.5 (NR) | 16.2 % of patients with low CD4+ count (<200) | No | NR | LBW and SGA, Still Birth |
| Kiarie 2003[106] | RCT | NR | 139 | Median: 25 (NR) | NR | Yes | 100 | LBW and SGA, Preterm, Still Birth |
| Koss 2014[107] | RCT | WHO: 1 (96%) | 356 | Median: 29.5 (25-33) | 14.04% of patients with low CD4+ count (<200) | Yes | 100 | Preterm Births, Stillbirths |
| Lambert 2000[108] | RCT | NR | 501 | Median: 26 (NR) | 18 % of patients using illicit drugs, 18 % of patients using alcohol, 31% of patients who smoke | No | 100 | LBW and SGA, Preterm |
| Limpongsanurak 2001[109] | RCT | NR | 182 | 24.6 (17-39) | NR | No | 100 | MTCT, Still Birth |
| Shaffer 1999[110]; CR: Chotpitayasunondh 2001[111] | RCT | NR | 397 | Median: 24 (NR) | <1 % of patients using illicit drugs, 11 % of patients with low CD4+ count (<200) | No | 100 | MTCT, LBW and SGA; Infant and Child, CM |
| Shapiro 2010[112]; CR: Shapiro 2013[113] | RCT | NR | 730 | Median: 26.5 (NR) | 23.3 % of patients with low CD4+ count (<200) | No | 100 | CM; Infant and Child, LBW and SGA, Preterm, Still Birth |
| Sperling 1998[114]; CR: Boyer 1994[115]; Connor 1994[116] | RCT | NR | 477 | NR (NR) | NR | No | NR | Infant and Child, CM, Preterm, Still Birth; MTCT, LBW and SGA |
| The Kesho Bora Study Group 2011[117] | RCT | WHO: 1, 2, 3 | 824 | Median: 27 (NR) | NR | Yes | 100 | Infant and Child, CM, LBW and SGA, Preterm, Still Birth |
| The Petra Study Team 2002[119] | RCT | WHO: 3, 4 (3.5%) | 1797 | Median: 26 (NR) | NR | Yes | 100 | Infant and Child, CM, Still Birth |
| Tubiana 2013[120] | RCT | NR | 107 | Median: 29.66 (NR) | 2.85% of patients who smoke | No | 100 | CM, Preterm |
| Wiktor 1999[121]; CR: Jamieson 2003[122], Tonwe-Gold 2007[123] | RCT | NR | 280 | Median: 25.5 (NR) | 6 % of patients with low CD4+ count (<200) | Yes | 100 | Infant and Child, CM, LBW and SGA, Still Birth; Preterm, MTCT |
| Bera 2010[124] | registry | WHO: 4 | 851 | NR (NR) | 11.6% of patients with TB co-infection | No | NR | CM |
| Vannappagari 2013[125] | registry | NR | 13537 | NR | NR | No | NR | LBW and SGA, Preterm, Still Birth |
| Habib 2008 [133] | registry | NR | 434 | NR (NR) | NR | Yes | 100 | Infant and Child, LBW and SGA, Preterm |
| Lindegren 2000[134] | registry | NR | 11636 | NR (NR) | NR | No | NR | Infant and Child |
| Phiri 2014[135]; CR:[136] | Registry | NR | 806 | Median: 25.7 (14-43) | 13 % of patients using illicit drugs, 7 % of patients using alcohol, 27% of patients who smoke | No | 93 | CM, LBW and SGA, Preterm |
| Witt 2007[137] | case-control | NR | 16 | Median: 29 (14-39) | NR | No | 100 | LBW and SGA, Preterm |
| **Abbreviations:** CDC, Centers for Disease Control and Prevention; CM, Congenital Malformation; CR, companion report; NR, Not reported; LBW and SGA, Low Birth weight and Small for Gestational Age; LMIC, Low to Middle Income Country; MTCT, Mother-to-Child Transmission of HIV; Main Paper, MP; NR, not reported; RCT, Randomised Control Trial; TB, tuberculosis; WHO, World Health Organization; **Note**:*Only companion reports with data included in analyses reported; †Median and range reported where Mean and Standard deviation not available; ‡NR could also include 0% | | | | | | | | |
