# Supplementary material for: Comparative safety and effectiveness of perinatal antiretroviral therapies for HIV-infected women and their children: Systematic review and network meta-analysis including different study designs
Source: PLoS One. 2018 Jun 18;13(6):e0198447. doi: 10.1371/journal.pone.0198447 (PMC6005568; doi:10.1371/journal.pone.0198447)
Supplement: S10 Appendix — (DOCX) [file pone.0198447.s010.docx]

# S10 Appendix. Interventions compared in the analyses

| **Author, Year** | **Analysis by Category** | **Analysis by Specific Drug** |
| --- | --- | --- |
| **Congenital Malformations** | | |
| Bae WH, 2008[21] | ART-mono,  HAART | ZDV,  ZDV+3TC+NVP |
| Bera E, 2010[25] |  | NVP,  EFV |
| Brogly, 2010[28] | ART-unspecified,  No treatment |  |
| Chotpitayasunondh T, 2001†[119] | ART-mono,  No treatment | ZDV,  No treatment |
| Contu L, 1995[34] | ART-mono,  No treatment | ZDV,  No treatment |
| Dabis F, 1999[91] | ART-mono,  No treatment | ZDV,  No treatment |
| Duran AS, 2006[37] | ART-mono,  ART-unspecified,  No treatment | ZDV,  No treatment |
| European Collaborative Study, 2003†[133] | ART-unspecified,  No treatment |  |
| Floridia M, 2006[42] | ART-mono,  ART-dual | EFV,  ddI+d4T |
| Gray G, 2006[93] | ART-mono,  ART-dual | d4T,  ZDV,  ddI,  ddI+d4T |
| Grosch-Woerner I, 2000[47] | ART-mono,  No treatment | ZDV,  No treatment |
| Hankin C, 2009[49] | ART-mono,  ART-dual,  HAART,  No treatment |  |
| Joao EC, 2010[52] | ART-unspecified,  No treatment |  |
| Jungmann EM, 2001†[148] |  | ZDV,  3TC+d4T,  ZDV+ddI+NVP,  ddI+d4T+NVP |
| Mandelbrot L, 2001[60] | ART-mono,  ART-dual | ZDV,  ZDV+3TC |
| Martinelli P, 2008†[158] | ART-unspecified,  No treatment |  |
| Mazur-Melewska K, 2005[65] | ART-mono,  HAART,  No treatment | ZDV,  ZDV+3TC+NLF,  ZDV+3TC+NVP,  No treatment |
| Phiri K, 2014[73] | ART-unspecified,  No treatment |  |
| Prieto L, 2014[89] | ART-mono,  ART-dual,  HAART,  No treatment | ZDV,  No treatment |
| Santini-Oliveira M, 2014[76] | ART-unspecified,  No treatment |  |
| Shapiro RL, 2010[101] |  | ZDV+3TC+NVP,  ZDV+3TC+LOP+RIT,  ZDV+3TC+ABC |
| Simon T, 2002†[174] | ART-mono,  ART-dual,  HAART,  No treatment | ZDV,  ZDV+3TC,  No treatment |
| Sperling RS, 1998[102] | ART-mono,  No treatment | ZDV,  No treatment |
| The Kesho Bora Study Group, 2011[103] | ART-mono,  HAART | ZDV,  ZDV+3TC+LOP+RIT |
| The Petra Study Team, 2002[104] | ART-dual,  No treatment | ZDV+3TC,  No treatment |
| Tubiana R, 2013[105] | ART-dual,  HAART | LOP+RIT,  ZDV+3TC+LOP+RIT |
| Watts D, 2007[85] | ART-unspecified,  No treatment |  |
| Wiktor SZ, 1999[106] | ART-mono,  No treatment | ZDV,  No treatment |
| **Mother-to-Child Transmission of HIV*** | | |
| Alvarez, 2007[18] | ART-unspecified + ART-mono,  No treatment + ART-dual |  |
| Boyer PJ, 1994†[112] | ART-mono + ART-mono,  No treatment + No treatment | [ZDV] + [ZDV],  [No treatment] + [No treatment] |
| Contu L, 1995[34] | ART-mono + ART-mono,  No treatment + No treatment |  |
| Cotter AM, 2012[35] | No treatment + ART-mono,  ART-mono + ART-mono,  ART-unspecified + ART-mono, | [No treatment] + [ZDV],  [ZDV] + [ZDV] |
| Dorenbaum A, 2002[92] | ART-unspecified + ART-mono,  ART-unspecified + placebo |  |
| Duran AS, 2006[37] | No treatment + No treatment,  ART-mono + ART-mono,  HAART + No treatment, |  |
| European Collaborative Study, 2006[38] | ART-mono + No treatment,  ART-dual + No treatment,  HAART + No treatment,  No treatment + No treatment, | [ZDV] + [No treatment],  [No treatment] + [No treatment] |
| Frenkel LM, 1997[43] | No treatment + ART-mono,  ART-mono + No treatment,  ART-mono + ART-mono, | [No treatment] + [ZDV],  [ZDV] + [No treatment],  [ZDV] + [ZDV] |
| Grosch-Woerner, I, 2000[47] | ART-mono + ART-mono,  No treatment + No treatment | [ZDV] + [ZDV],  [No treatment] + [No treatment] |
| Hoffman R, 2010[50] | No treatment + ART-mono,  HAART + No treatment,  No treatment + No treatment, | [No treatment] + [NVP],  [No treatment] + [No treatment] |
| Jamieson DJ, 2003†[147] | ART-mono + ART-mono,  Placebo + Placebo, | [ZDV] + [ZDV],  [Placebo] + [Placebo], |
| Limpongsanurak S, 2001[99] | ART-mono + ART-mono,  Placebo + Placebo | [ZDV] + [ZDV],  [Placebo] + [Placebo] |
| Rutstein RM, 2014[75] | ART-unspecified + No treatment,  No treatment + No treatment |  |
| Shaffer, N, 1999[100] | ART-mono + ART-mono,  placebo + placebo | [ZDV] + [ZDV],  [placebo] + [placebo] |
| Torpey K, 2012[82] | No treatment + ART-mono,  ART-mono + ART-mono,  HAART + No treatment,  No treatment + No treatment | [No treatment] + [NVP],  [ZDV] + [NVP],  [No treatment] + [No treatment] |
| Ugochukwu EF, 2009[83] | ART-unspecified + ART-mono,  No treatment + No treatment |  |
| Zucotti GV, 1999[87] | ART-mono + ART-mono,  ART-mono + No treatment,  No treatment + No treatment | [ZDV] + [ZDV],  [ZDV] + [No treatment],  [No Treatment] + [No treatment] |
| **Infant and Child Deaths** | | |
| Chotpitayasunondh T, 2001†[119] | ART-mono,  placebo | ZDV,  Placebo |
| Chung MH, 2005[90] |  | NVP,  ZDV |
| Chung MH, 2008†[120] | ART-dual,  HAART |  |
| Contu L, 1995[34] | ART-mono,  No treatment | ZDV,  No treatment |
| Dabis F, 2001†[128] | ART-mono,  placebo | ZDV,  placebo |
| Dryden-Peterson S, 2011†[130] | ART-mono,  HAART |  |
| Frenkel LM, 1995†[138] | ART-mono,  No treatment | ZDV,  No treatment |
| Gibb, 2012[45] | ART-mono,  No treatment | TDF,  No treatment |
| Habib N, 2008[48] | ART-unspecified,  no treatment |  |
| Lindegren, 2000[57] | ART-mono,  ART-dual,  ART-unspecified,  No treatment | ZDV,  ZDV+3TC,  No treatment |
| Lussiana C, 2012[59] | HAART,  No treatment |  |
| Mussi-Pinhata M, 2003[70] | ART-mono,  No treatment | ZDV,  No treatment |
| Onakewhor J, 2011[71] | HAART,  No treatment |  |
| Shapiro RL, 2013†[169] |  | ZDV+3TC+ABC,  ZDV+3TC+LOP+RIT,  ZDV+3TC+NVP |
| Sperling RS, 1998[102] | ART-mono,  placebo | ZDV,  placebo |
| The Kesho Bora Study Group, 2011[103] | ART-mono,  HAART | ZDV,  ZDV+3TC+LOP+RIT |
| The Petra Study Team, 2002[104] | ART-dual,  placebo | ZDV+3TC,  placebo |
| Townsend CL, 2007†[182] | HAART,  ART-unspecified |  |
| Wiktor SZ, 1999[106] | ART-mono,  placebo | placebo,  ZDV |
| **Preterm Births** | | |
| Areechokchai, 2009[19] | HAART,  ART-unspecified,  No treatment |  |
| Bae WH, 2008[21] | ART-mono,  HAART | ZDV,  ZDV+3TC+NVP |
| Bailey H, 2013[22] | ART-mono,  HAART,  No treatment | ZDV,  No treatment |
| Barral M, 2014[23] | HAART,  ART-unspecified,  No treatment | ZDV+3TC+NLF,  ZDV+3TC+LOP+RIT,  No treatment |
| Bellón Cano JM, 2004[24] | HAART,  ART-unspecified |  |
| Blood E, 2009[26] |  | 3TC+ddI+NLF,  ZDV+3TC+LOP+RIT,  ZDV+3TC+NLF,  ZDV+3TC+ABC |
| Boer, 2007[27] |  | ZDV+3TC+NVP,  ZDV+3TC+NLF |
| Briand N, 2013†[114] |  | ZDV,  No treatment |
| Chansinghakul D, 2009[31] | ART-dual,  HAART |  |
| Chen JY, 2012[32] | ART-mono,  HAART,  No treatment | ZDV,  No treatment |
| Contu L, 1995[34] | ART-mono,  No treatment | ZDV,  No treatment |
| Cotter AM, 2006†[124] | ART-mono,  ART-unspecified,  No treatment | ZDV,  No treatment |
| Culnane M, 1999†[126] | ART-mono,  placebo | ZDV,  Placebo |
| Dabis F, 1999[91] | ART-mono,  placebo | ZDV,  Placebo |
| Darak S, 2013[36] | ART-mono,  HAART |  |
| de Martino M, 1999†[146] | ART-mono,  No treatment | ZDV,  No treatment |
| European Collaborative Study, 2003†[133] | ART-mono,  ART-unspecified,  No treatment |  |
| Ezechi OC, 2012[39] | HAART,  ART-unspecified |  |
| Fiore S, 2006[40] | HAART,  ART-unspecified |  |
| Gartland MG, 2013[44] | ART-mono,  HAART |  |
| Gibb, 2012[45] | ART-mono,  No treatment | TDF,  No treatment |
| Grosch-Woerner I, 2008†[142] | ART-mono,  ART-dual,  HAART | ZDV,  ZDV+3TC |
| Habib N, 2008[48] | ART-unspecified,  No treatment |  |
| Hussain A, 2011[51] | ART-dual,  HAART,  No treatment | ZDV+NVP,  No treatment |
| Kiarie JN, 2003[96] | ART-mono,  No treatment | ZDV,  No treatment |
| Koss CA, 2014[97] |  | ZDV+3TC+EFV,  ZDV+3TC+LOP+RIT |
| Lambert JS, 2000[98] | ART-mono,  ART-unspecified |  |
| Lin HC, 2005[56] | ART-unspecified,  No treatment |  |
| Lopez M, 2012[58] | HAART,  ART-unspecified |  |
| Machado E, 2014†[154] | ART-mono,  ART-dual,  HAART |  |
| Mandelbrot L, 1998†[155] | ART-mono,  No treatment | ZDV,  No treatment |
| Mania A, 2013[61] | HAART,  ART-unspecified |  |
| Marazzi MC, 2011[62] | HAART,  ART-unspecified |  |
| Marczynska M, 2000[63] | ART-mono,  No treatment | ZDV,  No treatment |
| Matheson PB, 1995[64] | ART-mono,  No treatment | ZDV,  No treatment |
| Mazur-Melewska K, 2005[65] | ART-mono,  HAART,  No treatment | ZDV,  ZDV+3TC+NLF,  ZDV+3TC+NVP,  No treatment |
| McGowan J, 1999[66] | ART-dual,  HAART | ZDV+3TC,  ZDV+3TC+NLF,  ZDV+3TC+IND,  3TC+d4T+IND |
| Meyer S, 2014[67] | HAART,  No treatment | 3TC+d4T+NVP,  No treatment |
| Money D, 2007[68] | HAART,  ART-unspecified |  |
| Mussi-Pinhata M, 2003[70] | ART-mono,  No treatment | ZDV,  No treatment |
| Onakewhor J, 2011[71] | HAART,  No treatment |  |
| Pacheco SE, 2006†[162] | No treatment,  ART-unspecified |  |
| Phiri K, 2014[73] | ART-mono,  ART-dual,  ART-unspecified |  |
| Santini-Oliveira M, 2014[76] | ART-unspecified,  No treatment |  |
| Schulte J, 2007[77] | ART-mono,  ART-dual,  HAART,  No treatment |  |
| Shapiro RL, 2013†[169] |  | ZDV+3TC+ABC,  ZDV+3TC+LOP+RIT,  ZDV+3TC+NVP |
| Short CES, 2014[78] | ART-mono,  ART-dual,  HAART,  ART-unspecified,  No treatment | ZDV,  ZDV+3TC+ABC,  No treatment |
| Simonds RJ, 1998[79] | ART-mono,  No treatment | ZDV,  No treatment |
| Sinha G, 2007[80] | ART-mono,  No treatment | ZDV,  No treatment |
| Soler-Palacin P, 2012[81] | ART-mono,  HAART | ZDV,  ZDV+3TC+NVP  ZDV+3TC+LOP+RIT+TDF |
| Sperling RS, 1998[102] | ART-mono,  placebo | ZDV,  Placebo |
| Szyld EG, 2006†[178] | HAART,  ART-unspecified |  |
| The Kesho Bora Study Group, 2011[103] | ART-mono,  HAART | ZDV,  ZDV+3TC+LOP+RIT |
| Tonwe-Gold B, 2007†[180] | ART-dual,  HAART |  |
| Townsend CL, 2007†[182] | HAART,  ART-unspecified |  |
| Tubiana R, 2013[105] | ART-dual,  HAART | LOP+RIT,  ZDV+3TC+LOP+RIT |
| Van der Merwe K, 2011†[185] | HAART,  ART-unspecified | 3TC+d4T+EFV,  3TC+d4T+NVP,  3TC+d4T+LOP+RIT |
| Vannappagari V, 2013†[186] | ART-mono,  ART-unspecified |  |
| Viani R, 2010[84] | HAART,  no treatment |  |
| Watts DH, 2013†[191] | HAART,  ART-unspecified,  No treatment |  |
| Witt KL, 2007[107] |  | ZDV+3TC+LOP+RIT,  ZDV+3TC+NLF |
| Ziske J, 2013[86] | ART-mono,  No treatment | ZDV,  No treatment |
| Zuk DM, 2009[88] | ART-unspecified,  No treatment |  |
| **Stillbirths** |  |  |
| Bucceri AM, 2002[30] | ART-dual,  HAART | 3TC+d4T,  ZDV+3TC,  ZDV+3TC+NVP,  3TC+d4T+NVP,  ZDV+3TC+IND,  ZDV+3TC+NLF,  ZDV+3TC+SAQ,  3TC+d4T+SAQ |
| Chansinghakul D, 2009[31] | ART-dual,  HAART |  |
| Chen JY, 2012[32] | ART-mono,  HAART,  No treatment | ZDV,  No treatment |
| Chung MH, 2008†[120] | ART-dual,  HAART |  |
| Connor EM, 1994†[121] | ART-mono,  placebo | ZDV,  Placebo |
| Cotter AM, 2006†[124] | ART-mono,  ART-unspecified,  No treatment | ZDV,  No treatment |
| Dabis F, 1999[91] | ART-mono,  placebo | ZDV,  Placebo |
| Darak S, 2013[36] | ART-mono,  HAART |  |
| de Lemos L, 2012[54] | ART-unspecified,  No treatment |  |
| Fitzgerald FC, 2010[41] | HAART,  ART-unspecified,  No treatment |  |
| Floridia M, 2006[42] | ART-mono,  ART-dual | EFV,  ddI+d4T |
| Gartland MG, 2013[44] | ART-mono,  HAART |  |
| Gibb, 2012[45] | ART-mono,  No treatment | TDF,  No treatment |
| Gray G, 2006[93] | ART-mono,  ART-dual | ZDV,  d4T,  ddI,  d4T+ddI |
| Guay LA, 1999[94] | ART-mono,  placebo | ZDV,  NVP,  placebo |
| Hussain A, 2011[51] | ART-dual,  HAART,  No treatment | ZDV+NVP,  No treatment |
| Jackson JB, 2003[95] | ART-mono,  placebo | NVP,  ZDV,  Placebo |
| Joao EC, 2010[52] | ART-unspecified,  No treatment |  |
| Kiarie JN, 2003[96] | ART-mono,  No treatment | ZDV,  No treatment |
| Koss CA, 2014[97] |  | ZDV+3TC+LOP+RIT,  ZDV+3TC+EFV |
| Leroy V, 2008[55] | ART-mono,  ART-dual | ZDV,  ZDV+3TC |
| Limpongsanurak S, 2001[99] | ART-mono,  placebo | ZDV,  Placebo |
| Mandelbrot L, 2001[60] | ART-mono,  ART-dual | ZDV,  ZDV+3TC |
| Marazzi MC, 2011[62] | HAART,  No treatment |  |
| McGowan J, 1999[66] | ART-dual,  HAART | ZDV+3TC,  ZDV+3TC+NLF,  ZDV+3TC+IND,  3TC+d4T+IND |
| Msellati P, 2001[69] | ART-mono,  No treatment | ZDV,  No treatment |
| Onakewhor J, 2011[71] | HAART,  No treatment |  |
| Read J, 2007b[74] | ART-mono,  No treatment | ZDV,  No treatment |
| Shapiro RL, 2013†[169] |  | ZDV+3TC+ABC,  ZDV+3TC+LOP+RIT,  ZDV+3TC+NVP |
| Short CES, 2014[78] | ART-mono,  ART-dual,  HAART,  ART-unspecified,  No treatment | ZDV,  ZDV+3TC+ABC,  No treatment |
| Sinha G, 2007[80] | ART-mono,  No treatment | ZDV,  No treatment |
| Sperling RS, 1998[102] | ART-mono,  placebo | ZDV,  Placebo |
| St. John A, 2003[53] | ART-mono,  No treatment | ZDV,  No treatment |
| Suy A, 2006†[177] | HAART,  ART-unspecified,  No treatment |  |
| The Kesho Bora Study Group, 2011[103] | ART-mono,  HAART | ZDV,  ZDV+3TC+LOP+RIT |
| The Petra Study Team, 2002[104] | ART-dual,  placebo | ZDV+3TC,  Placebo |
| Tonwe-Gold B, 2007†[180] | ART-dual,  HAART |  |
| Townsend CL, 2007†[182] | ART-unspecified,  HAART |  |
| Vannappagari V, 2013†[186] | ART-mono,  ART-unspecified |  |
| Viani R, 2010[84] | HAART,  No treatment |  |
| Watts D, 2004†[188] | ART-mono,  ART-unspecified |  |
| Watts D, 2007[85] | ART-unspecified,  No treatment |  |
| Wiktor SZ, 1999[106] | ART-mono,  placebo | ZDV,  Placebo |
| **Low Birth Weight** | | |
| Asavapiriyanont S, 2011[20] | ART-mono,  ART-dual,  HAART,  No treatment |  |
| Barral M, 2014[23] | HAART,  ART-unspecified,  No treatment | ZDV+3TC+NLF,  ZDV+3TC+LOP+RIT,  No treatment |
| Bellón Cano JM, 2004[24] | HAART,  ART-unspecified |  |
| Briand N, 2013†[114] | ART-mono,  No treatment | ZDV,  No treatment |
| Chansinghakul D, 2009[31] | ART-dual,  HAART |  |
| Chmait, 2002[33] | ART-mono,  HAART |  |
| Chung MH, 2005[90] |  | NVP,  ZDV |
| Chung MH, 2008†[120] | ART-dual,  HAART |  |
| Connor EM, 1994†[121] | ART-mono,  placebo | ZDV,  placebo |
| Contu L, 1995[34] | ART-mono,  No treatment | ZDV,  No treatment |
| Cooper ER, 2002†[122] | ART-mono,  HAART,  ART-unspecified,  No treatment | ZDV,  No treatment |
| Cotter AM, 2006†[124] | ART-mono,  ART-unspecified,  No treatment | ZDV,  No treatment |
| Culnane M, 1999†[126] | ART-mono,  No treatment | ZDV,  placebo |
| Dabis F, 1999[91] | ART-mono,  placebo | ZDV,  placebo |
| Darak S, 2013[36] | ART-mono,  HAART |  |
| Dryden-Peterson S, 2011†[130] | ART-mono,  HAART |  |
| European Collaborative Study, 2003†[133] | ART-unspecified,  No treatment |  |
| Gibb, 2012[45] | ART-mono,  No treatment | TDF,  No treatment |
| Giuliano M, 2013†[140] |  | ZDV+3TC+NVP,  3TC+d4t+NVP |
| Goldstein PJ, 2000[46] | ART-mono,  ART-dual,  HAART,  No treatment | ZDV,  ZDV+3TC,  ZDV+3TC+NVP,  No treatment |
| Grosch-Woerner I, 2008†[142] | ART-mono,  ART-dual,  HAART | ZDV,  ZDV+3TC |
| Guay LA, 1999[94] |  | NVP,  ZDV |
| Habib N, 2008[48] | ART-unspecified,  No treatment |  |
| Hussain A, 2011[51] | ART-dual,  HAART,  No treatment | ZDV+NVP,  No treatment |
| Jackson JB, 2003[95] |  | NVP,  ZDV |
| Kiarie JN, 2003[96] | ART-mono,  No treatment | ZDV,  No treatment |
| Lambert JS, 2000[98] | ART-mono,  ART-unspecified |  |
| Leroy V, 2008[55] | ART-mono,  ART-dual | ZDV,  ZDV+3TC |
| Machado E, 2009†[154] | ART-mono,  ART-dual,  HAART |  |
| Mania A, 2013[61] | HAART,  ART-unspecified |  |
| Mazur-Melewska K, 2005[65] | ART-mono,  HAART,  No treatment | ZDV,  ZDV+3TC+NLF,  ZDV+3TC+NVP,  No treatment |
| McGowan J, 1999[66] | ART-dual,  HAART | ZDV+3TC,  ZDV+3TC+NLF,  ZDV+3TC+IND,  3TC+d4T+IND |
| Meyer S, 2014[67] | HAART,  No treatment | 3TC+d4T+NVP,  No treatment |
| Money D, 2007[68] | ART-mono,  ART-dual,  HAART |  |
| Onakewhor J, 2011[71] | HAART,  No treatment |  |
| Parker MM, 2003[72] | ART-unspecified,  No treatment |  |
| Phiri K, 2014[73] | ART-mono,  ART-dual,  ART-unspecified |  |
| Santini-Oliveira M, 2014[76] | ART-unspecified,  No treatment |  |
| Schulte J, 2007[77] | ART-mono,  ART-dual,  HAART,  No treatment |  |
| Shaffer N, 1999[100] | ART-mono,  placebo | ZDV,  placebo |
| Shapiro RL, 2013†[169] |  | ZDV+3TC+ABC,  ZDV+3TC+LOP+RIT,  ZDV+3TC+NVP |
| Simonds RJ, 1998[79] | ART-mono,  No treatment | ZDV,  No treatment |
| Sinha G, 2007[80] | ART-mono,  No treatment | ZDV,  No treatment |
| Soler-Palacin P, 2012[81] | ART-mono,  HAART | ZDV,  ZDV+3TC+NVP, ZDV+3TC+LOP+RIT+TDF |
| Szyld EG, 2006†[178] | HAART,  ART-unspecified |  |
| The Kesho Bora Study Group, 2011 | ART-mono,  HAART | ZDV,  ZDV+3TC+LOP+RIT |
| Tonwe-Gold B, 2007†[103] | ART-dual,  HAART |  |
| Van der Merwe K, 2011†[185] | HAART,  ART-unspecified | 3TC+d4T+EFV,  3TC+d4T+NVP,  3TC+d4T+LOP+RIT |
| Vannappagari V, 2013[186] | ART-mono,  ART-unspecified |  |
| Wiktor SZ, 1999[106] | ART-mono,  placebo | ZDV,  placebo |
| Witt KL, 2007[107] |  | ZDV+3TC+LOP+RIT,  ZDV+3TC+NLF |
| Zuk DM, 2009[88] | ART-unspecified,  No treatment |  |
| **Short length** |  |  |
| Briand N, 2013†[114] | ART-mono,  No treatment |  |
| **Small Head** |  |  |
| Mania A, 2013[61] | ART-unspecified,  HAART |  |
| **Notes:** *Treatment administered during two time points: during pregnancy and labour  †Companion report to an included main article  **Abbreviations:** ART, Antiretroviral Therapy; HAART, Highly Active Anti-Retroviral; ABC, Abacavir; ddI, Didanosine; 3TC, Lamivudine; d4T, Stavudine; AZT (ZDV), Zidovudine; EFV, Sustiva; NVP, Nevirapine; IND, Indinavir; LPV (LOP), Lopinavir; NFV (NLF) Nelfinavir; r (RIT), Ritonavir; SAQ, Saquinavir; NoT, No Treatment; Plc, Placebo | | |
