# Supplementary material for: Comparative safety and effectiveness of perinatal antiretroviral therapies for HIV-infected women and their children: Systematic review and network meta-analysis including different study designs
Source: PLoS One. 2018 Jun 18;13(6):e0198447. doi: 10.1371/journal.pone.0198447 (PMC6005568; doi:10.1371/journal.pone.0198447)
Supplement: S11 Appendix — (DOCX) [file pone.0198447.s011.docx]

# S11 Appendix. Cochrane EPOC Risk-of-bias appraisal results (n=17 RCTs)

| **Author, Year** | **1** | **2** | **3** | **4** | **5** | **6** | **7** | **8** | **9** |
| --- | --- | --- | --- | --- | --- | --- | --- | --- | --- |
| Chung MH, 2005 | Low | Unclear | NA | Low | High | Low | Low | Unclear | Low |
| Dabis F, 1999 | Low | Low | NA | Low | Low | Low | Unclear | Unclear | Low |
| Dorenbaum A, 2002 | Unclear | Unclear | NA | Low | Low | Low | Unclear | Unclear | High |
| Gray G, 2006 | Unclear | Unclear | NA | Low | Low | Low | Unclear | Unclear | Unclear |
| Guay LA, 1999 | Low | Low | NA | Low | Low | Low | Unclear | Unclear | Unclear |
| Jackson, JB, 2003 | Low | Low | NA | Low | Low | Low | Unclear | Low | Unclear |
| Kiarie JN, 2003 | Low | Unclear | NA | Low | Unclear | Low | Unclear | Unclear | Low |
| Koss CA, 2014 | Low | Unclear | NA | Low | Low | Low | Unclear | Low | Unclear |
| Lambert JS, 2000 | Unclear | Unclear | NA | Low | Low | Low | Unclear | Unclear | Low |
| Limpongsanurak S, 2001 | Unclear | Unclear | NA | Unclear | Low | Low | Unclear | Unclear | Low |
| Shaffer, N, 1999 | Low | Low | NA | Low | Low | Low | Unclear | Unclear | High |
| Shapiro RL, 2010 | Unclear | Unclear | NA | Low | Low | Low | Unclear | Low | Unclear |
| Sperling RS, 1998 | Unclear | Unclear | NA | Low | Low | Low | Unclear | Unclear | Unclear |
| The Kesho Bora Study Group, 2011 | Low | High | NA | Low | Low | Low | Unclear | Low | Low |
| The Petra Study Team, 2002 | Low | Low | NA | Low | High | Low | Unclear | Unclear | High |
| Tubiana R, 2013 | Unclear | Unclear | NA | Low | Low | Low | Unclear | Low | Low |
| Wiktor SZ, 1999 | Low | Low | NA | Low | Low | Low | Unclear | Unclear | Unclear |

**Abbreviations:** EPOC, Effective Practice and Organization of Care; RCTs, Randomised Clinical Trials; High, High risk of bias; Low, Low risk of bias; Unclear, Unclear risk of bias; NA, Not applicable.

**Items:**

1. Random sequence generation

2. Allocation concealment

3. Similar baseline outcome measures

4. Similar baseline characteristics

5. Incomplete outcome data

6. Blinding

7. Contamination

8. Selective outcome reporting

9. Other bias
