# Supplementary material for: Comparative safety and effectiveness of perinatal antiretroviral therapies for HIV-infected women and their children: Systematic review and network meta-analysis including different study designs
Source: PLoS One. 2018 Jun 18;13(6):e0198447. doi: 10.1371/journal.pone.0198447 (PMC6005568; doi:10.1371/journal.pone.0198447)
Supplement: S12 Appendix — (DOCX) [file pone.0198447.s012.docx]

# S12 Appendix. Newcastle-Ottawa Scale appraisal results

**n=73 observational studies (72 cohorts and 1 case control study*)**

| **Author, Year** | **1** | **2** | **3** | **4** | **5** | **6** | **7** | **8** |
| --- | --- | --- | --- | --- | --- | --- | --- | --- |
| Alvarez 2007 | B | A | A | A | D | A | A | A |
| Areechokchai 2009 | B | A | A | A | D | A | A | D |
| Asavapiriyanont 2011 | B | A | A | A | D | A | A | B |
| Bae 2008 | B | A | A | A | A | A | A | A |
| Bailey 2013 | A | A | A | A | C | A | A | B |
| Barral 2014 | B | A | A | A | D | A | A | D |
| Bellón Cano 2004 | A | A | A | A | D | A | A | A |
| Bera 2010 | B | A | A | A | D | A | A | B |
| Blood 2009 | B | A | A | A | D | A | A | B |
| Boer 2006 | B | A | A | A | A | A | A | D |
| Brogly 2010 | B | A | A | A | C | A | A | D |
| Bucceri 2002 | B | A | A | A | D | A | A | A |
| Chansinghakul 2009 | B | A | A | A | D | A | A | B |
| Chen 2012 | B | A | A | A | C | A | A | B |
| Chmait 2002 | B | C | A | A | D | A | A | B |
| Contu 1995 | B | A | A | A | D | A | A | B |
| Cotter 2012 | B | A | A | A | C | A | A | A |
| Darak 2013 | B | A | A | A | A | A | A | C |
| de Lemos 2012 | B | A | A | A | D | A | A | C |
| Duran 2006 | B | A | A | A | C | A | A | C |
| European Collaborative Study 2006 | A | A | A | A | C | A | A | D |
| Ezechi 2012 | B | A | A | A | A | A | A | D |
| Fiore 2006 | B | A | A | A | C | A | A | C |
| Fitzgerald 2010 | B | A | A | A | D | A | A | C |
| Floridia 2006 | A | A | A | A | D | A | A | D |
| Frenkel 1997 | B | A | A | A | C | A | A | B |
| Gartland 2013 | B | B | A | A | A | A | A | C |
| Gibb 2012 | B | A | A | A | D | A | A | B |
| Goldstein 2000 | B | A | A | A | D | A | A | D |
| Grosch-Woerner 2000 | A | A | A | A | C | A | A | A |
| Habib 2008 | B | A | A | A | C | A | A | C |
| Hankin 2009 | A | A | A | A | D | A | A | C |
| Hoffman 2010 | B | A | A | A | C | A | A | C |
| Hussain 2011 | B | A | A | A | D | A | A | C |
| Joao 2010 | A | A | A | A | D | A | A | B |
| Leroy 2008 | B | C | A | A | C | A | A | C |
| Lin 2005 | B | A | A | A | D | A | A | D |
| Lindegren 2000 | A | A | A | A | D | A | A | D |
| Lopez 2012 | B | A | D | A | D | A | A | D |
| Lussiana 2012 | B | A | A | A | C | A | A | C |
| Mandelbrot 2001 | A | A | A | A | C | A | A | B |
| Mania 2013 | B | A | A | A | D | A | A | D |
| Marazzi 2011 | B | A | A | A | C | A | A | D |
| Marczynska 2000 | B | A | A | A | D | A | A | B |
| Matheson 1995 | B | A | A | A | C | A | A | C |
| Mazur-Melewska 2005 | B | A | A | A | D | A | A | D |
| McGowan 1999 | B | A | A | A | D | A | A | A |
| Meyer 2014 | B | A | A | A | D | A | A | A |
| Money 2007 | A | A | A | A | D | A | A | D |
| Msellati 2001 | B | A | A | A | D | A | A | C |
| Mussi-Pinhata 2003 | B | C | A | A | C | A | A | D |
| Onakewhor 2011 | B | A | A | A | D | A | A | A |
| Parker 2003 | D | A | A | A | D | A | A | C |
| Phiri 2014 | B | A | A | A | A | A | A | B |
| Prieto 2014 | B | A | A | A | A | A | A | D |
| Read 2007b | B | A | A | A | D | A | A | B |
| Rutstein 2014 | B | A | A | A | D | A | A | A |
| Santini-Oliveira 2014 | B | A | A | A | D | A | A | A |
| Schulte 2007 | A | A | A | A | C | A | A | D |
| Short CES 2014 | B | A | A | A | A | A | A | A |
| Simonds 1998 | B | A | A | A | C | A | A | C |
| Sinha 2007 | B | A | A | A | C | A | A | D |
| Soler-Palacin 2012 | B | A | A | A | D | A | A | B |
| St. John 2003 | B | C | A | A | D | A | A | D |
| Torpey 2012 | A | A | A | A | A | A | A | D |
| Ugochukwu 2009 | B | A | A | A | D | A | A | D |
| Vannappagari 2013 | A | A | A | A | D | A | A | A |
| Viani 2010 | B | A | A | A | D | A | A | C |
| Watts 2007 | B | A | A | A | C | A | A | D |
| Witt 2007 * | B | A | A | A | D | A | A | D |
| Ziske 2013 | B | A | B | A | D | A | A | C |
| Zucotti 1999 | B | A | A | A | D | A | A | D |
| Zuk 2009 | A | A | A | A | D | A | A | D |

**Appraisal Items and abbreviations:**

1. Representativeness of the exposed cohort: A, Truly representative of the average HIV-infected pregnant woman taking ART in the community; B, somewhat representative of the average HIV-infected pregnant woman taking ART in the community; D, no description of the derivation of the cohort
2. Selection of the non-exposed cohort: A, drawn from the same community as the exposed cohort; B, drawn from a different source; C, no description of the derivation of the non-exposed cohort
3. Ascertainment of exposure: A, Secure record (e.g., medical records, surgical records); B, Structured interview; D, No description
4. Demonstration that outcome of interest was not present at start of study: A, Yes; D, No description
5. Comparability of cohorts on the basis of the design or analysis: A, study controls for age and one other important factor; C, study controls for any other important factor; D, study does not control for any important factor or it is not described
6. Assessment of outcome: A, Independent OR blind assessment; D, No description
7. Was follow-up long enough for outcomes to occur: A, yes; D, No description

Adequacy of follow-up of cohorts: A, complete follow-up - all subjects accounted for; B, subjects lost to follow-up unlikely to introduce bias - small number lost, or description provided of those lost; C, follow-up rate is inadequate and no description of those lost; D, no statement
