# Supplementary material for: Comparative safety and effectiveness of perinatal antiretroviral therapies for HIV-infected women and their children: Systematic review and network meta-analysis including different study designs
Source: PLoS One. 2018 Jun 18;13(6):e0198447. doi: 10.1371/journal.pone.0198447 (PMC6005568; doi:10.1371/journal.pone.0198447)
Supplement: S13 Appendix — (DOCX) [file pone.0198447.s013.docx]

# S13 Appendix. Funnel Plots including Antiretroviral Drug Categories by Outcomes reported


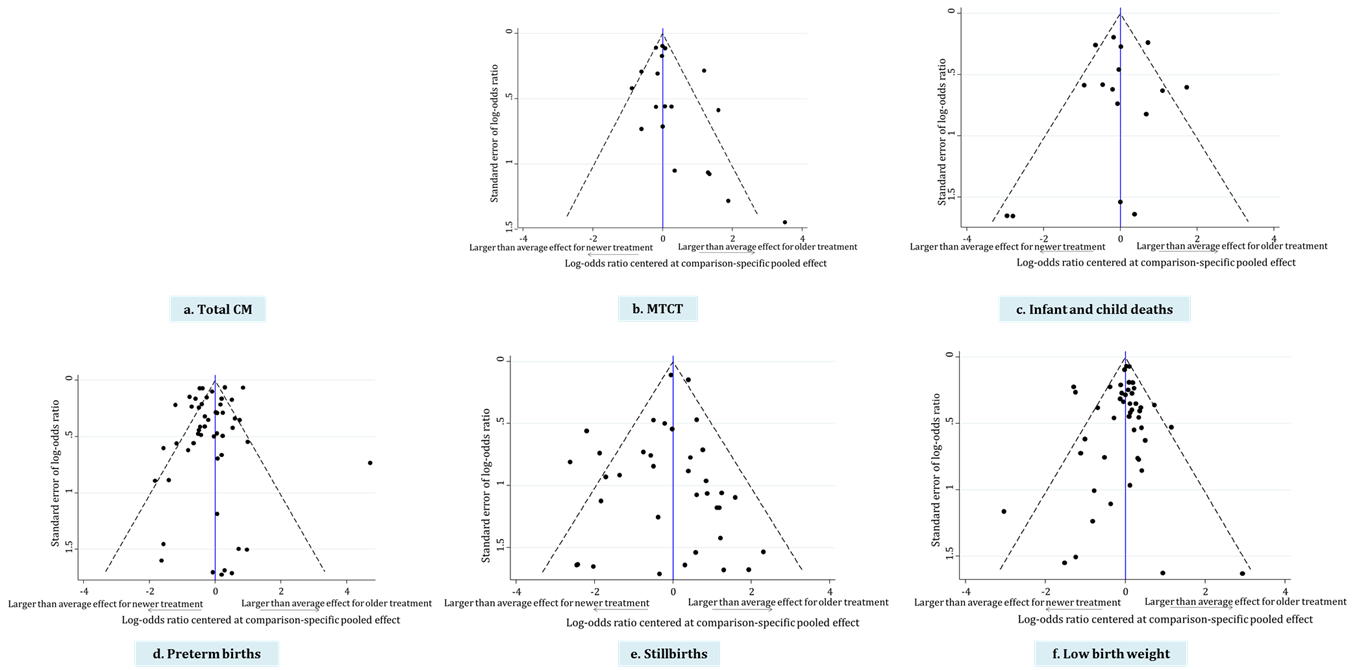

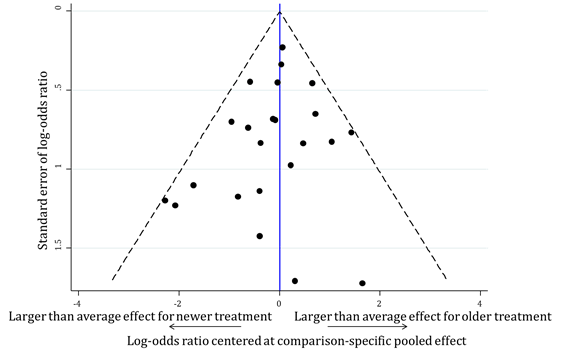


**Funnel Plots including Antiretroviral Drug Categories by Outcomes reported.**

**Legend: A) Total Congenital Malformations**– #17 studies, #7833 patients, # 4 treatments **B) Mother-to-Child Transmission of HIV**– #12 studies, #14967 patients, #6 treatments **C) Infant and child deaths**– 15 studies, #11451 patients, # 4 treatments **D) Preterm births** – 40 studies, # 36727 patients, # 4 treatments **E) Stillbirths** – 33 studies, # 21545 patients, # 4 treatments **F) Low birth weight** – 35 studies, # 31319 patients, # 4 treatments
