# Supplementary material for: Comparative safety and effectiveness of perinatal antiretroviral therapies for HIV-infected women and their children: Systematic review and network meta-analysis including different study designs
Source: PLoS One. 2018 Jun 18;13(6):e0198447. doi: 10.1371/journal.pone.0198447 (PMC6005568; doi:10.1371/journal.pone.0198447)
Supplement: S14 Appendix — (DOCX) [file pone.0198447.s014.docx]

# S14 Appendix. Network Diagrams for Anti-Retroviral Therapy Drug Categories


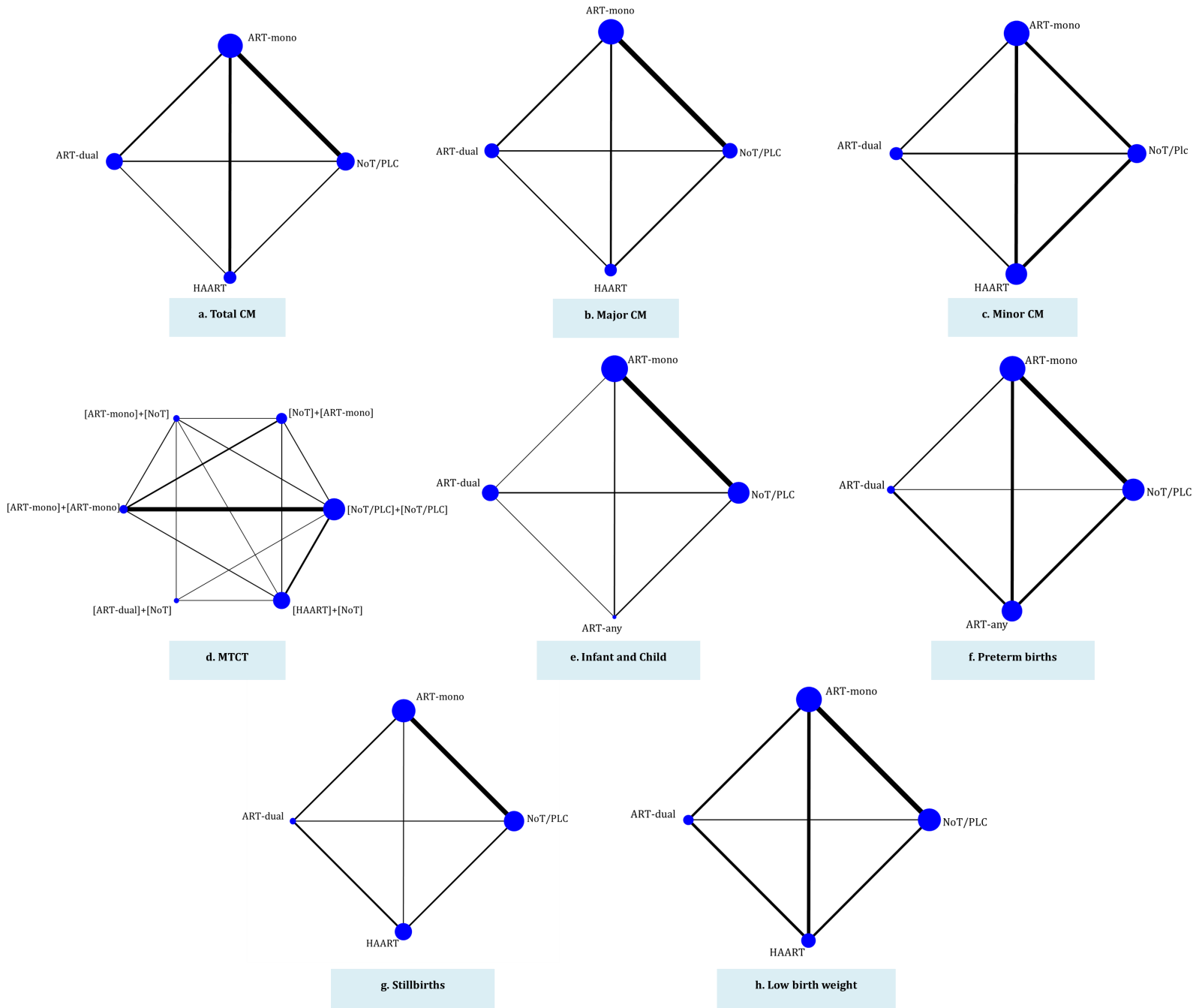


**Network Diagrams for Anti-Retroviral Therapy Drug Categories.**

**Legend:** Network diagrams of antiretroviral medications for each outcome. Each node represents an antiretroviral medication and each line represents a direct comparison between medications. The nodes are weighted according to the number of patients in each medication, and the lines are weighted according to the number of studies included in the direct comparison. **A) Total Congenital Malformations** –# 17 studies, # 7833 patients, # 4 treatments **B) Major Congenital Malformations -** #9 studies, # 3475 patients, # 4 treatments **C) Minor Congenital Malformations** - 2 studies, # 87 patients, # 4 treatments **D)** **Mother to Child Transmission of HIV**– #12 studies, # 14967 patients, # 6 treatments **E) Infant and child deaths–** # 15 studies, #11451 patients, # 4 treatments **F) Preterm births**– # 40 studies, # 36727 patients, # 4 treatments **G) Stillbirths**– # 33 studies, # 21545 patients, # 4 treatments **H) Low birth weight–** 35 studies, # 31319 patients, # 4 treatments **Abbreviations:** ABC, Abacavir; ddI, Didanosine; CM, Congenital Malformations; IND, Indinavir; 3TC, Lamivudine; LOP, Lopinavir; MTCT, Mother-to-Child Transmission of HIV; NVP, Nevirapine; NLF Nelfinavir; NoT, No Treatment; Plc, Placebo; SAQ, Saquinavir; d4T Stavudine; EFV, Sustiva; RIT, Ritonavir; ZDV, Zidovudine.
