# Supplementary material for: Comparative safety and effectiveness of perinatal antiretroviral therapies for HIV-infected women and their children: Systematic review and network meta-analysis including different study designs
Source: PLoS One. 2018 Jun 18;13(6):e0198447. doi: 10.1371/journal.pone.0198447 (PMC6005568; doi:10.1371/journal.pone.0198447)
Supplement: S15 Appendix — (DOCX) [file pone.0198447.s015.docx]

# S15 Appendix. Transitivity Assessment Results

| **Treatment comparison** | ***CD4+ Count*** | ***Antenatal Care*** | ***Illicit Drugs*** | ***Maternal Age*** | ***Baseline Risk*** | ***LMIC*** | ***Study Designs*** |
| --- | --- | --- | --- | --- | --- | --- | --- |
| ***Mother-to-child transmission of HIV [treatment drug]*** | | | | | | | |
| [ZDV]+[NoT] vs [NoT/PLC]+[NoT/PLC] | 14.00 | 100.00 | 20.27 | 28.50 | 0.15 | No | Cohorts |
| [ZDV]+[ZDV] vs [NoT/PLC]+[NoT/PLC] | 12.60 | 100.00 | 13.00 | 25.20 | 0.23 | No | RCTs/Cohorts |
| [NoT]+[NVP] vs [NoT/PLC]+[NoT/PLC] | 76.00 | NR | NR | 30.20 | 0.25 | No | Cohorts |
| [ZDV]+[NVP] vs [NoT/PLC]+[NoT/PLC] | NR | NR | NR | NR | 0.33 | No | Cohorts |
| [ZDV]+[NoT] vs [NoT]+[ZDV] | 21.00 | 100.00 | 20.00 | 28.10 | NA | No | Cohorts |
| [ZDV]+[ZDV] vs [NoT]+[ZDV] | 17.20 | 100.00 | 15.43 | 28.10 | NA | No | Cohorts |
| [ZDV]+[ZDV] vs [ZDV]+[NoT] | 21.00 | 100.00 | 20.00 | 28.10 | NA | No | Cohorts |
| [ZDV]+[NVP] vs [NoT]+[NVP] | NR | NR | NR | NR | NA | No | Cohorts |
| ***Mother-to-child transmission of HIV [treatment category]*** | | | | | | | |
| [NoT]+[ART-mono] vs [NoT/PLC]+[NoT/PLC] | 76.00 | NR | NR | 30.20 | 0.25 | No | Cohorts |
| [ART-mono]+[NoT] vs [NoT/PLC]+[NoT/PLC] | 14.00 | 100.00 | 20.27 | 28.50 | 0.15 | No | Cohorts |
| [ART-mono]+[ART-mono] vs [NoT/PLC]+[NoT/PLC] | 12.60 | 100.00 | 14.60 | 25.60 | 0.26 | No | RCTs/Cohorts |
| [ART-dual]+[NoT] vs [NoT/PLC]+[NoT/PLC] | 14.00 | 100.00 | 20.27 | 28.50 | 0.13 | No | Cohorts |
| [HAART]+[NoT] vs [NoT/PLC]+[NoT/PLC] | 45.00 | 100.00 | 19.04 | 28.50 | 0.25 | No | Cohorts |
| [ART-mono]+[NoT] vs [NoT]+[ART-mono] | 21.00 | 100.00 | 20.00 | 28.10 | NA | No | Cohorts |
| [ART-mono]+[ART-mono] vs [NoT]+[ART-mono] | 17.20 | 100.00 | 15.43 | 28.10 | NA | No | Cohorts |
| [HAART]+[NoT] vs [NoT]+[ART-mono] | 76.00 | NR | NR | 30.20 | NA | No | Cohorts |
| [ART-mono]+[ART-mono] vs [ART-mono]+[NoT] | 21.00 | 100.00 | 20.00 | 28.10 | NA | No | Cohorts |
| [ART-dual]+[NoT] vs [ART-mono]+[NoT] | 14.00 | 100.00 | 20.27 | 28.50 | NA | No | Cohorts |
| [HAART]+[NoT] vs [ART-mono]+[NoT] | 14.00 | 100.00 | 20.27 | 28.50 | NA | No | Cohorts |
| [HAART]+[NoT] vs [ART-mono]+[ART-mono] | NR | NR | 17.80 | 26.80 | NA | No | Cohorts |
| [HAART]+[NoT] vs [ART-dual]+[NoT] | 14.00 | 100.00 | 20.27 | 28.50 | NA | No | Cohorts |
| ***Total Congenital Malformations [treatment drug]*** | | | | | | | |
| ZDV+3TC+NVP vs ZDV+3TC+ABC | NR | NR | NR | NR | NA | No | Cohorts |
| ZDV+3TC+LOP+RIT vs ZDV+3TC+ABC | NR | NR | NR | 29.00 | NA | No | Cohorts |
| ZDV+3TC+NVP vs ZDV+3TC+NFV | 23.30 | 100.00 | NR | 26.50 | NA | No | RCTs |
| ddI+d4T+NVP vs ZDV+ddI+NVP | 0.00 | 100.00 | NR | 29.66 | NA | No | RCTs |
| ZDV+3TC+LOP+RIT vs ZDV+3TC+NVP | NR | NR | NR | NR | NA | No | Cohorts |
| ZDV+3TC+LOP+RIT vs LOP+RIT | NR | NR | NR | NR | NA | No | Cohorts |
| ZDV+3TC vs NoT/PLC | 7.97 | 100.00 | 25.89 | 26.86 | 0.08 | No | RCTs/Cohorts |
| ZDV+3TC+NFV vs NoT/PLC | NR | 100.00 | NR | 26.00 | 0.08 | No | RCTs/Cohorts |
| ZDV+3TC+NVP vs NoT/PLC | NR | NR | NR | NR | 0.09 | No | Cohorts |
| ZDV vs NoT/PLC | NR | NR | NR | 26.75 | 0.12 | No | Cohorts |
| ZDV+3TC vs ZDV | NR | NR | NR | 29.00 | NA | No | Cohorts |
| ZDV+3TC+NFV vs ZDV | 100.00 | 100.00 | NR | NR | NA | No | Cohorts |
| ZDV+ddI+NVP vs ZDV | 0.00 | 100.00 | NR | 27.00 | NA | No | RCTs |
| ddI+d4T+NVP vs ZDV | NR | 100.00 | NR | 28.30 | NA | No | RCTs |
| ZDV+3TC+NVP vs ZDV | NR | 100.00 | NR | 28.30 | NA | No | RCTs |
| ZDV+3TC+LOP+RIT vs ZDV | NR | 100.00 | NR | 28.30 | NA | No | RCTs |
| d4T vs ZDV | NR | NR | NR | 29.00 | NA | No | Cohorts |
| ddl vs ZDV | NR | 100.00 | 14.00 | 29.33 | NA | No | Cohorts |
| d4T+ddI vs ZDV | NR | 100.00 | NR | 28.30 | NA | No | RCTs |
| 3TC+d4T vs ZDV | NR | 100.00 | NR | 28.30 | NA | No | RCTs |
| ddl vs d4T | NR | 100.00 | NR | 28.30 | NA | No | RCTs |
| d4T+ddI vs d4T | NR | NR | 23.90 | 33.60 | NA | No | Cohorts |
| d4T+ddI vs ddl | NR | NR | NR | NR | NA | No | Cohorts |
| EFV vs d4T+ddI | NR | NR | NR | 29.00 | NA | No | Cohorts |
| NVP vs EFV | NR | NR | NR | 29.00 | NA | No | Cohorts |
| ZDV+ddI+NVP vs 3TC+d4T | 23.30 | 100.00 | NR | 26.50 | NA | No | RCTs |
| ddI+d4T+NVP vs 3TC+d4T | 23.30 | 100.00 | NR | 26.50 | NA | No | RCTs |
| ***Total Congenital Malformations [treatment category]*** | | | | | | | |
| ART-mono vs Not/PLC | 7.97 | 100.00 | 20.66 | 25.99 | 0.09 | No | RCTs/Cohorts |
| ART-dual vs Not/PLC | 11.91 | 100.00 | 5.98 | 29.17 | 0.11 | No | RCTs/Cohorts |
| HAART vs Not/PLC | 11.91 | 100.00 | 5.98 | 30.75 | 0.11 | No | Cohorts |
| ART-dual vs ART-mono | 11.91 | 100.00 | 8.66 | 29.78 | NA | No | RCTs/Cohorts |
| HAART vs ART-mono | 27.98 | 100.00 | 5.98 | 29.5 | NA | No | RCTs/Cohorts |
| HAART vs ART-dual | 11.91 | 100.00 | 5.98 | 30.38 | NA | No | RCTs/Cohorts |
| ***Major Congenital Malformations [treatment drug]*** | | | | | | | |
| ZDV+d4T+3TC+NVP vs NoT/Plc | 8.03 | 100.00 | 9.87 | 26.63 | 0.11 | No | RCTs/Cohorts |
| ZDV vs NoT/Plc | NR | 100.00 | NR | 24.50 | 0.17 | No | Cohorts |
| ZDV+d4T+3TC vs NoT/Plc | NR | NR | NR | NR | 0.08 | No | Cohorts |
| ZDV+d4T+3TC+NFV vs NoT/Plc | NR | NR | NR | NR | 0.08 | No | Cohorts |
| ZDV+d4T+3TC+NVP vs ZDV | NR | NR | NR | 29.00 | NA | No | Cohorts |
| d4T+3TC+d4T vs ZDV | NR | 100.00 | 14.00 | 29.33 | NA | No | Cohorts |
| ZDV+d4T+3TC vs ZDV | NR | NR | NR | NR | NA | No | Cohorts |
| ZDV+d4T+3TC+NFV vs ZDV | NR | NR | NR | 29.00 | NA | No | Cohorts |
| ZDV+ddI+NVP vs ZDV | NR | NR | NR | 29.00 | NA | No | Cohorts |
| ddI+d4T+NVP vs ZDV | NR | NR | NR | NR | NA | No | Cohorts |
| ZDV+ddI+NVP vs d4T+3TC+d4T | NR | NR | NR | 29.00 | NA | No | Cohorts |
| ddI+d4T+NVP vs d4T+3TC+d4T | NR | NR | NR | 29.00 | NA | No | Cohorts |
| ZDV+d4T+3TC+NVP vs ZDV+d4T+3TC+NFV | NR | NR | NR | NR | NA | No | Cohorts |
| ddI+d4T+NVP vs ZDV+ddI+NVP | NR | NR | NR | 29.00 | NA | No | Cohorts |
| ***Major Congenital Malformations [treatment category]*** | | | | | | | |
| ART-mono vs Not/PLC | 6.73 | 100.00 | 2.10 | 25.50 | 0.12 | No | RCTs/Cohorts |
| ART-dual vs Not/PLC | NR | 100.00 | 2.10 | 30.50 | 0.15 | No | Cohorts |
| HAART vs Not/PLC | NR | 100.00 | 2.10 | 30.50 | 0.13 | No | Cohorts |
| ART-dual vs ART-mono | NR | 100.00 | 8.05 | 29.92 | NA | No | Cohorts |
| HAART vs ART-mono | NR | 100.00 | 2.10 | 30.50 | NA | No | Cohorts |
| HAART vs ART-dual | NR | 100.00 | 2.10 | 30.50 | NA | No | Cohorts |
| ***Minor Congenital Malformations [treatment drug]*** | | | | | | | |
| ZDV vs NoT/PLC | NR | 100.00 | NR | 24.50 | 0.00 | No | Cohorts |
| ZDV+3TC vs NoT/PLC | NR | 100.00 | NR | NR | 0.00 | No | Cohorts |
| ZDV+3TC+NFV vs NoT/PLC | NR | NR | NR | NR | 0.00 | No | Cohorts |
| ZDV+3TC+NVP vs NoT/PLC | NR | NR | NR | NR | 0.00 | No | Cohorts |
| ZDV+3TC vs ZDV | NR | 100.00 | NR | NR | NA | No | Cohorts |
| ZDV+3TC+NFV vs ZDV | NR | NR | NR | NR | NA | No | Cohorts |
| ZDV+3TC+NVP vs ZDV | NR | NR | NR | NR | NA | No | Cohorts |
| ZDV+3TC+NVP vs ZDV+3TC+NFV | NR | NR | NR | NR | NA | No | Cohorts |
| ***Minor Congenital Malformations [treatment category]*** | | | | | | | |
| ART-mono vs NoT/PLC | NR | 100.00 | NR | 24.50 | 0.00 | No | Cohorts |
| ART-dual vs NoT/PLC | NR | 100.00 | NR | NR | 0.00 | No | Cohorts |
| HAART vs NoT/PLC | NR | 100.00 | NR | NR | 0.00 | No | Cohorts |
| ART-dual vs ART-mono | NR | 100.00 | NR | NR | NA | No | Cohorts |
| HAART vs ART-mono | NR | 100.00 | NR | NR | NA | No | Cohorts |
| HAART vs ART-dual | NR | 100.00 | NR | NR | NA | No | Cohorts |
| ***Infant Deaths [treatment drug]*** | | | | | | | |
| ZDV vs NoT/PLC | 7.25 | 82.10 | 10.10 | 25.17 | 0.06 | No | RCTs/Cohorts |
| TDF vs NoT/PLC | 26.00 | 100.00 | NR | NR | 0.10 | Yes | RCTs/Cohorts |
| ZDV+3TC vs NoT/PLC | NR | 100.00 | NR | 26.00 | 0.09 | Yes | RCTs/Cohorts |
| ZDV+3TC vs ZDV | NR | NR | NR | NR | NA | No | Cohorts |
| NVP vs ZDV | 10.30 | 100.00 | NR | NR | NA | Yes | RCTs |
| ZDV+3TC+LOP+RIT vs ZDV | 0.00 | 100.00 | NR | 27.00 | NA | Yes | RCTs |
| ZDV+3TC+NVP vs ZDV+3TC+ABC | 23.30 | 100.00 | NR | 26.32 | NA | Yes | RCTs |
| ZDV+3TC+LOP+RIT vs ZDV+3TC+ABC | 23.30 | 100.00 | NR | 26.32 | NA | Yes | RCTs |
| ZDV+3TC+LOP+RIT vs ZDV+3TC+NVP | 23.30 | 100.00 | NR | 26.32 | NA | Yes | RCTs |
| ***Infant Deaths [treatment category]*** | | | | | | | |
| ART-mono vs Not/PLC | 11.00 | 85.68 | 10.10 | 25.17 | 0.07 | No | RCTs/Cohorts |
| ART-dual vs Not/PLC | NR | 100.00 | NR | 26.00 | 0.09 | Yes | RCTs/Cohorts |
| HAART vs Not/PLC | NR | 82.30 | NR | 29.20 | 0.04 | Yes | Cohorts |
| ART-dual vs ART-mono | NR | NR | NR | NR | NA | No | Cohorts |
| HAART vs ART-mono | 0.00 | 100.00 | NR | 28.60 | NA | Yes | RCTs/Cohorts |
| ART-any vs ART-dual | NR | NR | NR | NR | NA | No | Cohorts |
| HAART vs ART-dual | NR | 100.00 | NR | 25.00 | NA | Yes | RCTs |
| HAART vs ART-any | 10.81 | 100.00 | NR | 29.70 | NA | No | Cohorts |
| ***Low Birth Weight [treatment drug]*** | | | | | | | |
| ZDV+3TC+NVP vs 3TC+d4T+NVP | NR | 100.00 | NR | 27.00 | NA | Yes | Cohorts |
| 3TC+d4T+LOP+RIT vs 3TC+d4T+NVP | 74.00 | NR | NR | 30.10 | NA | No | Cohorts |
| ZDV+3TC+LOP+RIT vs ZDV+3TC+NVP | 23.30 | 100.00 | NR | 26.32 | NA | No | Cohorts |
| ZDV+3TC+LOP+RIT+TDF vs ZDV+3TC+NVP | 7.70 | NR | 28.20 | 30.50 | NA | No | Cohorts |
| ZDV+NVP vs NoT/PLC | 48.00 | 100.00 | NR | 24.00 | 0.03 | No | Cohorts |
| 3TC+d4T+NVP vs NoT/PLC | NR | NR | NR | 27.60 | 0.02 | Yes | Cohorts |
| ZDV+3TC+NVP vs NoT/PLC | 18.06 | 100.00 | NR | NR | 0.31 | No | Cohorts |
| ZDV+3TC+LOP+RIT vs NoT/PLC | NR | 83.50 | NR | 27.00 | 0.17 | No | Cohorts |
| ZDV vs NoT/PLC | 10.27 | 97.27 | 14.40 | 25.04 | 0.18 | No | RCTs |
| TDF vs NoT/PLC | 26.00 | 100.00 | NR | NR | 0.19 | Yes | Cohorts |
| ZDV+3TC vs NoT/PLC | 18.06 | 100.00 | NR | NR | 0.45 | No | Cohorts |
| ZDV+3TC+NLF vs NoT/PLC | NR | 83.50 | NR | 27.00 | 0.17 | No | Cohorts |
| NVP vs ZDV | 14.17 | 100.00 | 0.00 | 24.50 | NA | Yes | RCTs |
| ZDV+3TC+NVP vs ZDV | 12.88 | 100.00 | 28.20 | 30.50 | NA | No | RCTs |
| ZDV+3TC+LOP+RIT vs ZDV | 0.00 | 100.00 | NR | 27.00 | NA | Yes | Cohorts |
| ZDV+3TC+LOP+RIT+TDF vs ZDV | 7.70 | NR | 28.20 | 30.50 | NA | No | Cohorts |
| ZDV+3TC vs ZDV | 13.24 | 100.00 | 18.58 | 27.00 | NA | No | Cohorts |
| ZDV+3TC+NLF vs ZDV | NR | NR | NR | NR | NA | No | Cohorts |
| ZDV+3TC vs ZDV+3TC+IND | 43.33 | 100.00 | 37.00 | 29.00 | NA | No | Cohorts |
| 3TC+d4T+IND vs ZDV+3TC+IND | 43.33 | 100.00 | 37.00 | 29.00 | NA | No | Cohorts |
| ZDV+3TC+NLF vs ZDV+3TC+IND | 43.33 | 100.00 | 37.00 | 29.00 | NA | No | Cohorts |
| ZDV+3TC+NVP vs ZDV+3TC | 18.06 | 100.00 | NR | NR | NA | No | Cohorts |
| 3TC+d4T+IND vs ZDV+3TC | 43.33 | 100.00 | 37.00 | 29.00 | NA | No | Cohorts |
| ZDV+3TC+NLF vs ZDV+3TC | 43.33 | 100.00 | 37.00 | 29.00 | NA | No | Cohorts |
| ZDV+3TC+NLF vs 3TC+d4T+IND | 43.33 | 100.00 | 37.00 | 29.00 | NA | No | Cohorts |
| ZDV+3TC+NVP vs ZDV+3TC+ABC | 23.30 | 100.00 | NR | 26.32 | NA | Yes | RCTs |
| ZDV+3TC+LOP+RIT vs ZDV+3TC+ABC | 23.30 | 100.00 | NR | 26.32 | NA | Yes | RCTs |
| 3TC+d4T+NVP vs 3TC+d4T+EFV | 74.00 | NR | NR | 30.10 | NA | Yes | Cohorts |
| 3TC+d4T+LOP+RIT vs 3TC+d4T+EFV | 74.00 | NR | NR | 30.10 | NA | No | Cohorts |
| ZDV+3TC+NVP vs ZDV+3TC+NLF | NR | NR | NR | NR | NA | No | Cohorts |
| ZDV+3TC+LOP+RIT vs ZDV+3TC+NLF | NR | 91.75 | NR | 28.00 | NA | No | Case-Control/Cohorts |
| ***Low Birth Weight [treatment category]*** | | | | | | | |
| ART-mono vs Not/PLC | 11.70 | 93.69 | 16.72 | 25.04 | 0.18 | No | RCTs/Cohorts |
| ART-dual vs Not/PLC | 33.03 | 82.67 | 33.00 | 24.00 | 0.22 | No | Cohorts |
| HAART vs Not/PLC | 33.03 | 85.63 | 32.00 | 26.60 | 0.18 | No | Cohorts |
| ART-dual vs ART-mono | 12.16 | 90.17 | 20.20 | 26.36 | NA | No | Cohorts |
| HAART vs ART-mono | 9.04 | 94.22 | 23.96 | 27.91 | NA | No | RCTs/Cohorts |
| HAART vs ART-dual | 23.02 | 94.22 | 24.40 | 26.65 | NA | No | RCTs/Cohorts |
| ***Preterm Birth [treatment drug]*** | | | | | | | |
| 3TC+d4T+LOP+RIT vs 3TC+d4T+NVP | 74.00 | NR | NR | 30.10 | NA | No | Cohorts |
| ZDV+3TC+LOP+RIT vs ZDV+3TC+NVP | 23.30 | 100.00 | NR | 26.32 | NA | Yes | RCTs |
| ZDV+3TC+EFV vs ZDV+3TC+NVP | 7.70 | NR | 28.20 | 30.50 | NA | No | Cohorts |
| ZDV+3TC+LOP+RIT vs LOP+RIT | 0.00 | 100.00 | NR | 29.66 | NA | No | RCTs |
| ZDV+3TC+EFV vs ZDV+3TC+LOP+RIT | 14.00 | 100.00 | NR | 30.00 | NA | Yes | RCTs |
| ZDV+NVP vs NoT/PLC | 48.00 | 100.00 | NR | 24.00 | 0.65 | No | Cohorts |
| 3TC+d4T+NVP vs NoT/PLC | NR | NR | NR | 27.60 | 0.23 | No | Cohorts |
| ZDV+3TC+NVP vs NoT/PLC | NR | NR | NR | NR | 0.08 | No | Cohorts |
| ZDV+3TC+LOP+RIT vs NoT/PLC | NR | 83.50 | NR | 27.00 | 0.24 | No | Cohorts |
| ZDV vs NoT/PLC | 9.62 | 86.48 | 16.83 | 25.74 | 0.17 | No | RCTs/Cohorts |
| TDF vs NoT/PLC | 26.00 | 100.00 | NR | NR | 0.11 | Yes | RCTs |
| ZDV+3TC+ABC vs NoT/PLC | NR | NR | 0.90 | 32.20 | 0.13 | No | Cohorts |
| ZDV+3TC+NLF vs NoT/PLC | NR | 83.50 | NR | 27.00 | 0.16 | No | Cohorts |
| ZDV+3TC+NVP vs ZDV | 53.85 | 100.00 | 28.20 | 30.50 | NA | No | Cohorts |
| ZDV+3TC+LOP+RIT vs ZDV | 0.00 | 100.00 | NR | 27.00 | NA | Yes | RCTs |
| ZDV+3TC+EFV vs ZDV | 7.70 | NR | 28.20 | 30.50 | NA | No | Cohorts |
| ZDV+3TC vs ZDV | 5.46 | 100.00 | 18.58 | 27.83 | NA | No | Cohorts |
| ZDV+3TC+ABC vs ZDV | NR | NR | 0.90 | 32.20 | NA | No | Cohorts |
| ZDV+3TC+NLF vs ZDV | NR | NR | NR | NR | NA | No | Cohorts |
| ZDV+3TC vs ZDV+3TC+IND | 43.33 | 100.00 | 37.00 | 29.00 | NA | No | Cohorts |
| 3TC+d4T+IND vs ZDV+3TC+IND | 43.33 | 100.00 | 37.00 | 29.00 | NA | No | Cohorts |
| ZDV+3TC+NLF vs ZDV+3TC+IND | 43.33 | 100.00 | 37.00 | 29.00 | NA | No | Cohorts |
| 3TC+d4T+IND vs ZDV+3TC | 43.33 | 100.00 | 37.00 | 29.00 | NA | No | Cohorts |
| ZDV+3TC+NLF vs ZDV+3TC | 43.33 | 100.00 | 37.00 | 29.00 | NA | No | Cohorts |
| ZDV+3TC+NLF vs 3TC+d4T+IND | 43.33 | 100.00 | 37.00 | 29.00 | NA | No | Cohorts |
| ZDV+3TC+NVP vs ZDV+3TC+ABC | 23.30 | 100.00 | NR | 26.32 | NA | Yes | RCTs |
| ZDV+3TC+LOP+RIT vs ZDV+3TC+ABC | 23.30 | 100.00 | 0.00 | 28.66 | NA | Yes | RCTs |
| ZDV+3TC+NLF vs ZDV+3TC+ABC | NR | 100.00 | 0.00 | 31.00 | NA | No | Cohorts |
| 3TC+d4T+NVP vs 3TC+d4T+EFV | 74.00 | NR | NR | 30.10 | NA | No | Cohorts |
| 3TC+d4T+LOP+RIT vs 3TC+d4T+EFV | 74.00 | NR | NR | 30.10 | NA | No | Cohorts |
| ZDV+3TC+NVP vs ZDV+3TC+NLF | 34.00 | 100.00 | 5.00 | 29.00 | NA | No | Cohorts |
| ZDV+3TC+LOP+RIT vs ZDV+3TC+NLF | NR | 94.50 | 0.00 | 29.00 | NA | No | Case-control/Cohorts |
| ***Preterm Birth [treatment category]*** | | | | | | | |
| ART-mono vs Not/PLC | 11.29 | 84.21 | 18.59 | 25.79 | 0.17 | No | RCTs/Cohorts |
| ART-dual vs Not/PLC | 48.00 | 74.00 | 16.95 | 28.10 | 0.33 | No | Cohorts |
| HAART vs Not/PLC | 19.52 | 88.67 | 20.73 | 27.44 | 0.25 | No | Cohorts |
| ART-dual vs ART-mono | 9.97 | 85.25 | 15.38 | 27.37 | NA | No | Cohorts |
| HAART vs ART-mono | 20.24 | 92.94 | 17.45 | 28.13 | NA | NR | RCTs/Cohorts |
| HAART vs ART-dual | 22.78 | 93.50 | 19.70 | 28.11 | NA | No | RCTs/Cohorts |
| ***Stillbirth [treatment drug]*** | | | | | | | |
| ZDV+3TC+NLF vs 3TC+d4T+IND | 43.33 | 100.00 | 37.00 | 29.00 | NA | No | Cohorts |
| ZDV+3TC+NVP vs ZDV+3TC+ABC | 23.30 | 100.00 | NR | 26.32 | NA | Yes | RCTs |
| ZDV+3TC+LOP+RIT vs ZDV+3TC+ABC | 23.30 | 100.00 | NR | 26.32 | NA | Yes | RCTs |
| ZDV+3TC+SAQ vs ZDV+3TC+NLF | 14.00 | NR | 54.00 | 30.00 | NA | No | Cohorts |
| 3TC+d4T+SAQ vs ZDV+3TC+NLF | 14.00 | NR | 54.00 | 30.00 | NA | No | Cohorts |
| 3TC+d4T+NVP vs ZDV+3TC+NLF | 14.00 | NR | 54.00 | 30.00 | NA | No | Cohorts |
| ZDV+3TC+NVP vs ZDV+3TC+NLF | 14.00 | NR | 54.00 | 30.00 | NA | No | Cohorts |
| 3TC+d4T+SAQ vs ZDV+3TC+SAQ | 14.00 | NR | 54.00 | 30.00 | NA | No | Cohorts |
| 3TC+d4T+NVP vs ZDV+3TC+SAQ | 14.00 | NR | 54.00 | 30.00 | NA | No | Cohorts |
| ZDV+3TC+NVP vs ZDV+3TC+SAQ | 14.00 | NR | 54.00 | 30.00 | NA | No | Cohorts |
| 3TC+d4T+NVP vs 3TC+d4T+SAQ | 14.00 | NR | 54.00 | 30.00 | NA | No | Cohorts |
| ZDV+3TC+NVP vs 3TC+d4T+SAQ | 14.00 | NR | 54.00 | 30.00 | NA | No | Cohorts |
| ZDV+3TC+NVP vs 3TC+d4T+NVP | 14.00 | NR | 54.00 | 30.00 | NA | No | Cohorts |
| ZDV+3TC+LOP+RIT vs ZDV+3TC+NVP | 23.30 | 100.00 | NR | 26.32 | NA | Yes | RCTs |
| ZDV+3TC+EFV vs ZDV+3TC+LOP+RIT | 14.00 | 100.00 | NR | 30.00 | NA | Yes | RCTs |
| ZDV+3TC+ABC vs NoT/PLC | NR | NR | 0.90 | 32.20 | 0.13 | No | Cohorts |
| NoT/PLC vs NVP | 16.10 | 100.00 | 0.00 | 24.50 | 0.05 | No | RCTs |
| NoT/PLC vs ZDV+NVP | 48.00 | 100.00 | NR | 24.00 | 0.06 | No | Cohorts |
| ZDV vs NoT/PLC | 9.37 | 99.59 | 8.55 | 25.15 | 0.06 | Yes | RCTs/Cohorts |
| TDF vs NoT/PLC | 26.00 | 100.00 | NR | NR | 0.03 | Yes | Cohorts |
| ZDV+3TC vs NoT/PLC | NR | 100.00 | NR | 26.00 | 0.01 | Yes | RCTs |
| ZDV+3TC+ABC vs ZDV | NR | NR | 0.90 | 32.20 | NA | No | Cohorts |
| NVP vs ZDV | 16.10 | 100.00 | 0.00 | 24.50 | NA | No | RCTs |
| ZDV+3TC+LOP+RIT vs ZDV | 0.00 | 100.00 | NR | 27.00 | NA | Yes | RCTs |
| d4T vs ZDV | NR | 100.00 | NR | 28.30 | NA | No | RCTs |
| ddl vs ZDV | NR | 100.00 | NR | 28.30 | NA | No | RCTs |
| d4T+ddI vs ZDV | NR | 100.00 | NR | 28.30 | NA | No | RCTs |
| ZDV+3TC vs ZDV | 16.20 | 100.00 | 14.00 | 27.75 | NA | No | RCTs/Cohorts |
| ddl vs d4T | NR | 100.00 | NR | 28.30 | NA | No | RCTs |
| d4T+ddI vs d4T | NR | 100.00 | NR | 28.30 | NA | No | RCTs |
| d4T+ddI vs ddl | NR | 100.00 | NR | 28.30 | NA | No | RCTs |
| ZDV+3TC+IND vs 3TC+d4T+IND | 43.33 | 100.00 | 37.00 | 29.00 | NA | No | Cohorts |
| ZDV+3TC+NLF vs ZDV+3TC+IND | 28.67 | 100.00 | 45.50 | 29.50 | NA | No | Cohorts |
| ZDV+3TC+SAQ vs ZDV+3TC+IND | 14.00 | NR | 54.00 | 30.00 | NA | No | Cohorts |
| 3TC+d4T+SAQ vs ZDV+3TC+IND | 14.00 | NR | 54.00 | 30.00 | NA | No | Cohorts |
| 3TC+d4T+NVP vs ZDV+3TC+IND | 14.00 | NR | 54.00 | 30.00 | NA | No | Cohorts |
| ZDV+3TC+NVP vs ZDV+3TC+IND | 14.00 | NR | 54.00 | 30.00 | NA | No | Cohorts |
| 3TC+d4T vs ZDV+3TC+IND | 14.00 | NR | 54.00 | 30.00 | NA | No | Cohorts |
| ZDV+3TC vs ZDV+3TC+IND | 28.67 | 100.00 | 45.50 | 29.50 | NA | No | Cohorts |
| ZDV+3TC+NLF vs 3TC+d4T | 14.00 | NR | 54.00 | 30.00 | NA | No | Cohorts |
| ZDV+3TC+SAQ vs 3TC+d4T | 14.00 | NR | 54.00 | 30.00 | NA | No | Cohorts |
| 3TC+d4T+SAQ vs 3TC+d4T | 14.00 | NR | 54.00 | 30.00 | NA | No | Cohorts |
| 3TC+d4T+NVP vs 3TC+d4T | 14.00 | NR | 54.00 | 30.00 | NA | No | Cohorts |
| ZDV+3TC+NVP vs 3TC+d4T | 14.00 | NR | 54.00 | 30.00 | NA | No | Cohorts |
| ZDV+3TC vs 3TC+d4T | 14.00 | NR | 54.00 | 30.00 | NA | No | Cohorts |
| 3TC+d4T+IND vs ZDV+3TC | 43.33 | 100.00 | 37.00 | 29.00 | NA | No | Cohorts |
| ZDV+3TC+NLF vs ZDV+3TC | 28.67 | 100.00 | 45.50 | 29.50 | NA | No | Cohorts |
| ZDV+3TC+SAQ vs ZDV+3TC | 14.00 | NR | 54.00 | 30.00 | NA | No | Cohorts |
| 3TC+d4T+SAQ vs ZDV+3TC | 14.00 | NR | 54.00 | 30.00 | NA | No | Cohorts |
| 3TC+d4T+NVP vs ZDV+3TC | 14.00 | NR | 54.00 | 30.00 | NA | No | Cohorts |
| ZDV+3TC+NVP vs ZDV+3TC | 14.00 | NR | 54.00 | 30.00 | NA | No | Cohorts |
| ***Stillbirth [treatment category]*** | | | | | | | |
| ART-mono vs Not/PLC | 11.03 | 99.63 | 8.55 | 25.15 | 0.06 | Yes | RCTs/Cohorts |
| ART-dual vs Not/PLC | 48.00 | 100.00 | 0.90 | 27.40 | 0.07 | No | RCTs/Cohorts |
| HAART vs Not/PLC | 53.44 | 95.57 | 46.77 | 26.56 | 0.08 | No | Cohorts |
| ART-dual vs ART-mono | 16.20 | 100.00 | 7.45 | 29.92 | NA | No | RCTs/Cohorts |
| HAART vs ART-mono | 7.77 | 98.89 | NR | 26.86 | NA | Yes | RCTs/Cohorts |
| HAART vs ART-dual | 30.71 | 100.00 | 45.50 | 27.00 | NA | No | RCTs/Cohorts |
| **Abbreviations:** ABC, Abacavir; ART, Antiretroviral Therapy; d4T, Stavudine; ddI, Didanosine; dual, duo- therapy; EFV, Sustiva; HAART, Highly Active Anti-Retroviral; IND, Indinavir; LMIC, Low Middle Income Country; LOP, Lopinavir; mono, monotherapy; NA, Not applicable; NFV (NLF), Nelfinavir; NoT, No Treatment; NR, Not reported; NVP, Nevirapine; Plc, Placebo; RCTs, Randomised Clinical Trials; RIT, Ritonavir; SAQ, Saquinavir; 3TC, Lamivudine; ZDV, Zidovudine. | | | | | | | |
