# Supplementary material for: Comparative safety and effectiveness of perinatal antiretroviral therapies for HIV-infected women and their children: Systematic review and network meta-analysis including different study designs
Source: PLoS One. 2018 Jun 18;13(6):e0198447. doi: 10.1371/journal.pone.0198447 (PMC6005568; doi:10.1371/journal.pone.0198447)
Supplement: S16 Appendix — (DOCX) [file pone.0198447.s016.docx]

# S16 Appendix. Network Meta-analysis and Meta-analysis results for Specific Antiretroviral Drugs by Outcomes

| ***Treatment Comparison*** | ***Network Meta-Analysis Odds Ratio Estimate (CrI)*** | ***Predictive interval*** | ***Pairwise Meta-Analysis Odds Ratio Estimate (CrI)*** | ***No. of Studies (No. of Patients)*** | ***Study design*** |
| --- | --- | --- | --- | --- | --- |
| ***Outcome: Total Congenital Malformations - # 20 studies (11 Cohorts + 9 RCTs, # 7503 patients, # 16 treatments)*** | | | | | |
| ZDV vs NoT/Plc | 1.11 (0.70 to 2.12) | 0.38 to 4.18 | 1.29 (0.71 to 2.64) | 10 (2051) | RCTs/Cohorts |
| d4T vs NoT/Plc | 4.39 (0.39 to 130.60) | 0.32 to 157.80 | - | - | - |
| d4T vs ZDV | 3.90 (0.35 to 115.70) | 0.27 to 132.40 | 4.64 (0.46 to 140.00) | 1 (184) | RCT |
| ddl vs NoT/Plc | 1.08 (0.02 to 38.77) | 0.02 to 43.28 | - | - | - |
| ddl vs ZDV | 0.95 (0.02 to 32.92) | 0.02 to 38.71 | 1.04 (0.02 to 56.57) | 1 (187) | RCT |
| ddl vs d4T | 0.24 (0.01 to 2.89) | 0.00 to 3.35 | 0.24 (0.01 to 2.58) | 1 (185) | RCT |
| d4T+ddI vs NoT/Plc | 5.99 (0.60 to 161.90) | 0.50 to 194.10 | - | - | - |
| d4T+ddI vs ZDV | 5.29 (0.53 to 137.00) | 0.43 to 165.30 | 5.43 (0.63 to 180.30) | 1 (187) | RCT |
| d4T+ddI vs d4T | 1.36 (0.21 to 9.25) | 0.15 to 12.58 | **1.40 (0.27 to 6.96)** | 1 (185) | RCT |
| d4T+ddI vs ddl | 5.64 (0.55 to 228.20) | 0.43 to 262.30 | **6.07 (0.74 to 165.70)** | 1 (188) | RCT |
| EFV vs NoT/Plc | 25.56 (0.31 to 21350.00) | 0.26 to 23700.00 | - | - | - |
| EFV vs ZDV | 22.07 (0.27 to 17940.00) | 0.25 to 21040.00 | - | - | - |
| EFV vs d4T | 5.32 (0.08 to 2262.00) | 0.07 to 2617.00 | - | - | - |
| EFV vs ddl | 24.96 (0.28 to 24270.00) | 0.24 to 25320.00 | - | - | - |
| EFV vs d4T+ddI | 3.71 (0.10 to 1446.00) | 0.08 to 1744.00 | 4.04 (0.13 to 4194.00) | 1 (51) | Cohort |
| 3TC+d4T vs NoT/Plc | 0.49 (0.02 to 5.18) | 0.01 to 6.54 | - | - | - |
| 3TC+d4T vs ZDV | 0.43 (0.01 to 4.05) | 0.01 to 5.17 | 0.42 (0.02 to 3.12) | 1 (114) | Cohort |
| 3TC+d4T vs d4T | 0.10 (0.00 to 3.08) | 0.00 to 3.51 | - | - | - |
| 3TC+d4T vs ddl | 0.41 (0.00 to 37.01) | 0.00 to 42.48 | - | - | - |
| 3TC+d4T vs d4T+ddI | 0.07 (0.00 to 2.07) | 0.00 to 2.40 | - | - | - |
| 3TC+d4T vs EFV | 0.02 (0.00 to 2.89) | 0.00 to 3.34 | - | - | - |
| ZDV+3TC vs NoT/Plc | 1.21 (0.52 to 2.63) | 0.30 to 4.57 | 0.91 (0.20 to 2.79) | 2 (1515) | RCTs/Cohorts |
| ZDV+3TC vs ZDV | 1.09 (0.40 to 2.20) | 0.24 to 3.53 | 1.24 (0.25 to 3.69) | 2 (1397) | Cohorts |
| ZDV+3TC vs d4T | 0.27 (0.01 to 3.37) | 0.01 to 4.21 | - | - | - |
| ZDV+3TC vs ddl | 1.13 (0.03 to 57.14) | 0.03 to 65.29 | - | - | - |
| ZDV+3TC vs d4T+ddI | 0.20 (0.01 to 2.16) | 0.01 to 2.60 | - | - | - |
| ZDV+3TC vs EFV | 0.05 (0.00 to 4.15) | 0.00 to 4.55 | - | - | - |
| ZDV+3TC vs 3TC+d4T | 2.53 (0.20 to 77.89) | 0.16 to 88.71 | - | - | - |
| ZDV+3TC+ABC vs NoT/Plc | 0.60 (0.10 to 3.88) | 0.08 to 5.06 | - | - | - |
| ZDV+3TC+ABC vs ZDV | 0.54 (0.09 to 2.94) | 0.07 to 3.78 | - | - | - |
| ZDV+3TC+ABC vs d4T | 0.13 (0.00 to 2.73) | 0.00 to 3.03 | - | - | - |
| ZDV+3TC+ABC vs ddl | 0.57 (0.01 to 38.84) | 0.01 to 44.21 | - | - | - |
| ZDV+3TC+ABC vs d4T+ddI | 0.10 (0.00 to 1.80) | 0.00 to 2.10 | - | - | - |
| ZDV+3TC+ABC vs EFV | 0.02 (0.00 to 2.74) | 0.00 to 3.01 | - | - | - |
| ZDV+3TC+ABC vs 3TC+d4T | 1.29 (0.07 to 56.41) | 0.06 to 60.62 | - | - | - |
| ZDV+3TC+ABC vs ZDV+3TC | 0.49 (0.08 to 3.71) | 0.06 to 4.78 | - | - | - |
| ZDV+3TC+NFV vs NoT/Plc | 0.27 (0.00 to 6.10) | 0.00 to 7.31 | 0.76 (0.00 to 44.21) | 1 (15) | Cohort |
| ZDV+3TC+NFV vs ZDV | 0.24 (0.00 to 5.34) | 0.00 to 6.19 | 0.11 (0.00 to 2.32) | 1 (13) | Cohort |
| ZDV+3TC+NFV vs d4T | 0.05 (0.00 to 3.14) | 0.00 to 3.55 | - | - | - |
| ZDV+3TC+NFV vs ddl | 0.21 (0.00 to 39.51) | 0.00 to 43.31 | - | - | - |
| ZDV+3TC+NFV vs d4T+ddI | 0.04 (0.00 to 2.32) | 0.00 to 2.70 | - | - | - |
| ZDV+3TC+NFV vs EFV | 0.01 (0.00 to 2.54) | 0.00 to 2.70 | - | - | - |
| ZDV+3TC+NFV vs 3TC+d4T | 0.57 (0.00 to 50.27) | 0.00 to 56.65 | - | - | - |
| ZDV+3TC+NFV vs ZDV+3TC | 0.22 (0.00 to 5.68) | 0.00 to 6.77 | - | - | - |
| ZDV+3TC+NFV vs ZDV+3TC+ABC | 0.43 (0.00 to 15.38) | 0.00 to 17.85 | - | - | - |
| NVP vs NoT/Plc | 16.22 (0.07 to 20770.00) | 0.06 to 21740.00 | - | - | - |
| NVP vs ZDV | 14.48 (0.06 to 17510.00) | 0.05 to 18990.00 | - | - | - |
| NVP vs d4T | 3.33 (0.02 to 2174.00) | 0.02 to 2295.00 | - | - | - |
| NVP vs ddl | 15.86 (0.06 to 19920.00) | 0.06 to 21100.00 | - | - | - |
| NVP vs d4T+ddI | 2.45 (0.02 to 1365.00) | 0.02 to 1508.00 | - | - | - |
| NVP vs EFV | 0.66 (0.02 to 6.58) | 0.02 to 8.16 | 0.70 (0.03 to 4.96) | 1 (217) | Cohort |
| NVP vs 3TC+d4T | 38.56 (0.09 to 82120.00) | 0.09 to 88680.00 | - | - | - |
| NVP vs ZDV+3TC | 13.58 (0.06 to 17920.00) | 0.05 to 19100.00 | - | - | - |
| NVP vs ZDV+3TC+ABC | 26.92 (0.09 to 43100.00) | 0.08 to 47520.00 | - | - | - |
| NVP vs ZDV+3TC+NFV | 79.75 (0.12 to 870600.00) | 0.11 to 945800.00 | - | - | - |
| ZDV+ddI+NVP vs NoT/Plc | 2.53 (0.22 to 20.79) | 0.18 to 27.40 | - | - | - |
| ZDV+ddI+NVP vs ZDV | 2.25 (0.21 to 16.45) | 0.16 to 20.88 | 2.39 (0.26 to 12.78) | 1 (102) | Cohort |
| ZDV+ddI+NVP vs d4T | 0.53 (0.01 to 12.89) | 0.01 to 14.73 | - | - | - |
| ZDV+ddI+NVP vs ddl | 2.29 (0.03 to 179.30) | 0.03 to 204.80 | - | - | - |
| ZDV+ddI+NVP vs d4T+ddI | 0.39 (0.01 to 8.97) | 0.01 to 10.28 | - | - | - |
| ZDV+ddI+NVP vs EFV | 0.09 (0.00 to 12.43) | 0.00 to 14.12 | - | - | - |
| ZDV+ddI+NVP vs 3TC+d4T | 5.32 (0.31 to 202.60) | 0.25 to 242.10 | 5.23 (0.29 to 174.00) | 1 (38) | Cohort |
| ZDV+ddI+NVP vs ZDV+3TC | 2.06 (0.17 to 19.43) | 0.15 to 25.26 | - | - | - |
| ZDV+ddI+NVP vs ZDV+3TC+ABC | 4.12 (0.22 to 63.10) | 0.18 to 74.42 | - | - | - |
| ZDV+ddI+NVP vs ZDV+3TC+NFV | 9.99 (0.17 to 6308.00) | 0.15 to 7016.00 | - | - | - |
| ZDV+ddI+NVP vs NVP | 0.15 (0.00 to 46.09) | 0.00 to 51.20 | - | - | - |
| ddI+d4T+NVP vs NoT/Plc | 2.55 (0.24 to 21.31) | 0.20 to 28.40 | - | - | - |
| ddI+d4T+NVP vs ZDV | 2.26 (0.22 to 17.10) | 0.18 to 21.31 | 2.43 (0.28 to 13.16) | 1 (102) | Cohort |
| ddI+d4T+NVP vs d4T | 0.55 (0.01 to 13.73) | 0.01 to 16.61 | - | - | - |
| ddI+d4T+NVP vs ddl | 2.39 (0.04 to 188.60) | 0.03 to 214.00 | - | - | - |
| ddI+d4T+NVP vs d4T+ddI | 0.40 (0.01 to 9.26) | 0.01 to 10.57 | - | - | - |
| ddI+d4T+NVP vs EFV | 0.10 (0.00 to 13.12) | 0.00 to 15.20 | - | - | - |
| ddI+d4T+NVP vs 3TC+d4T | 5.42 (0.30 to 180.70) | 0.26 to 204.00 | 5.08 (0.41 to 138.60) | 1 (38) | Cohort |
| ddI+d4T+NVP vs ZDV+3TC | 2.10 (0.19 to 20.92) | 0.15 to 26.34 | - | - | - |
| ddI+d4T+NVP vs ZDV+3TC+ABC | 4.19 (0.24 to 64.37) | 0.21 to 77.81 | - | - | - |
| ddI+d4T+NVP vs ZDV+3TC+NFV | 10.42 (0.20 to 5715.00) | 0.18 to 6048.00 | - | - | - |
| ddI+d4T+NVP vs NVP | 0.16 (0.00 to 58.92) | 0.00 to 63.97 | - | - | - |
| ddI+d4T+NVP vs ZDV+ddI+NVP | 1.03 (0.07 to 14.75) | 0.06 to 17.76 | 1.01 (0.09 to 12.48) | 1 (26) | Cohort |
| ZDV+3TC+NVP vs NoT/Plc | 1.08 (0.29 to 4.10) | 0.21 to 5.93 | 0.64 (0.00 to 22.32) | 1 (15) | Cohort |
| ZDV+3TC+NVP vs ZDV | 0.97 (0.27 to 3.10) | 0.18 to 4.52 | 0.76 (0.13 to 3.50) | 2 (191) | Cohorts |
| ZDV+3TC+NVP vs d4T | 0.25 (0.01 to 3.58) | 0.01 to 4.22 | - | - | - |
| ZDV+3TC+NVP vs ddl | 1.02 (0.02 to 58.89) | 0.02 to 70.47 | - | - | - |
| ZDV+3TC+NVP vs d4T+ddI | 0.18 (0.01 to 2.34) | 0.00 to 2.83 | - | - | - |
| ZDV+3TC+NVP vs EFV | 0.04 (0.00 to 3.93) | 0.00 to 4.34 | - | - | - |
| ZDV+3TC+NVP vs 3TC+d4T | 2.29 (0.17 to 85.90) | 0.14 to 91.74 | - | - | - |
| ZDV+3TC+NVP vs ZDV+3TC | 0.89 (0.22 to 4.09) | 0.16 to 5.69 | - | - | - |
| ZDV+3TC+NVP vs ZDV+3TC+ABC | 1.80 (0.38 to 8.68) | 0.28 to 12.19 | 1.87 (0.48 to 7.20) | 1 (439) | RCT |
| ZDV+3TC+NVP vs ZDV+3TC+NFV | 4.21 (0.15 to 1931.00) | 0.13 to 2169.00 | 1.03 (0.00 to 1761.00) | 1 (6) | Cohort |
| ZDV+3TC+NVP vs NVP | 0.07 (0.00 to 17.69) | 0.00 to 20.34 | - | - | - |
| ZDV+3TC+NVP vs ZDV+ddI+NVP | 0.44 (0.04 to 5.89) | 0.03 to 7.10 | - | - | - |
| ZDV+3TC+NVP vs ddI+d4T+NVP | 0.43 (0.04 to 5.49) | 0.03 to 6.77 | - | - | - |
| LOP+RIT vs NoT/Plc | 0.30 (0.05 to 2.14) | 0.04 to 2.99 | - | - | - |
| LOP+RIT vs ZDV | 0.27 (0.04 to 1.60) | 0.03 to 2.20 | - | - | - |
| LOP+RIT vs d4T | 0.07 (0.00 to 1.48) | 0.00 to 1.78 | - | - | - |
| LOP+RIT vs ddl | 0.29 (0.01 to 21.08) | 0.01 to 23.51 | - | - | - |
| LOP+RIT vs d4T+ddI | 0.05 (0.00 to 1.01) | 0.00 to 1.17 | - | - | - |
| LOP+RIT vs EFV | 0.01 (0.00 to 1.42) | 0.00 to 1.55 | - | - | - |
| LOP+RIT vs 3TC+d4T | 0.66 (0.04 to 29.35) | 0.03 to 34.19 | - | - | - |
| LOP+RIT vs ZDV+3TC | 0.25 (0.04 to 2.02) | 0.03 to 2.62 | - | - | - |
| LOP+RIT vs ZDV+3TC+ABC | 0.51 (0.07 to 4.29) | 0.05 to 5.25 | - | - | - |
| LOP+RIT vs ZDV+3TC+NFV | 1.21 (0.03 to 723.80) | 0.03 to 723.30 | - | - | - |
| LOP+RIT vs NVP | 0.02 (0.00 to 5.42) | 0.00 to 5.77 | - | - | - |
| LOP+RIT vs ZDV+ddI+NVP | 0.12 (0.01 to 2.55) | 0.01 to 2.99 | - | - | - |
| LOP+RIT vs ddI+d4T+NVP | 0.12 (0.01 to 2.18) | 0.01 to 2.60 | - | - | - |
| LOP+RIT vs ZDV+3TC+NVP | 0.28 (0.05 to 1.95) | 0.03 to 2.54 | - | - | - |
| ZDV+3TC+LOP+RIT vs NoT/Plc | 0.65 (0.19 to 2.47) | 0.14 to 3.61 | - | - | - |
| ZDV+3TC+LOP+RIT vs ZDV | 0.58 (0.17 to 1.83) | 0.11 to 2.76 | 0.61 (0.24 to 1.46) | 1 (805) | RCT |
| ZDV+3TC+LOP+RIT vs d4T | 0.14 (0.00 to 2.21) | 0.00 to 2.54 | - | - | - |
| ZDV+3TC+LOP+RIT vs ddl | 0.61 (0.01 to 36.03) | 0.01 to 42.07 | - | - | - |
| ZDV+3TC+LOP+RIT vs d4T+ddI | 0.11 (0.00 to 1.47) | 0.00 to 1.73 | - | - | - |
| ZDV+3TC+LOP+RIT vs EFV | 0.03 (0.00 to 2.57) | 0.00 to 2.93 | - | - | - |
| ZDV+3TC+LOP+RIT vs 3TC+d4T | 1.37 (0.11 to 47.53) | 0.08 to 56.84 | - | - | - |
| ZDV+3TC+LOP+RIT vs ZDV+3TC | 0.53 (0.14 to 2.51) | 0.10 to 3.56 | - | - | - |
| ZDV+3TC+LOP+RIT vs ZDV+3TC+ABC | 1.07 (0.24 to 5.52) | 0.17 to 7.47 | 1.06 (0.28 to 3.83) | 1 (553) | RCT |
| ZDV+3TC+LOP+RIT vs ZDV+3TC+NFV | 2.46 (0.09 to 1225.00) | 0.08 to 1317.00 | - | - | - |
| ZDV+3TC+LOP+RIT vs NVP | 0.04 (0.00 to 10.53) | 0.00 to 11.29 | - | - | - |
| ZDV+3TC+LOP+RIT vs ZDV+ddI+NVP | 0.26 (0.02 to 3.54) | 0.02 to 4.44 | - | - | - |
| ZDV+3TC+LOP+RIT vs ddI+d4T+NVP | 0.26 (0.02 to 3.33) | 0.02 to 4.19 | - | - | - |
| ZDV+3TC+LOP+RIT vs ZDV+3TC+NVP | 0.59 (0.17 to 2.24) | 0.13 to 3.36 | 0.56 (0.15 to 2.10) | 1 (426) | RCT |
| ZDV+3TC+LOP+RIT vs LOP+RIT | 2.13 (0.55 to 8.08) | 0.38 to 11.52 | 2.14 (0.92 to 5.10) | 1 (105) | RCT |
| *Common within-network between-study variance* | 0.15 (0.00 to 1.14) | - | 0.29 (0.00 to 1.75) | 20 (7503) | RCTs/Cohorts |
| *Design-by-treatment interaction model for inconsistency χ² (d.f., P-value, between-study variance)* | 7.93 (6, 0.24, 0.00) | |  |  | |
| *Model fit measures and diagnostics* Residual deviance = 49.45 Data points = 48 Effective number of parameters = 40.55 DIC = 90 | | | | | |
| ***Outcome: Major Congenital Malformations - # 9 studies (6 Cohorts + 3RCTs ; (6 2-arm, 1 3-arm, 2 4-arm) , # 2808 patients, # 8 treatments*** | | | | | |
| ZDV vs NoT/PLC | 0.86 (0.44 to 1.67) | 0.22 to 3.29 | 0.88 (0.45 to 1.74) | 7 (1304) | RCTs/Cohorts |
| 3TC+d4T vs NoT/PLC | 0.34 (0.01 to 3.83) | 0.01 to 4.96 | - | - | - |
| 3TC+d4T vs ZDV | 0.41 (0.01 to 4.13) | 0.01 to 5.38 | 0.41 (0.02 to 3.10) | 1 (114) | Cohort |
| ZDV+3TCvs NoT/PLC | 1.27 (0.27 to 3.90) | 0.17 to 5.82 | 0.13 (0.00 to 4.28) | 1 (14) | Cohort |
| ZDV+3TCvs ZDV | 1.49 (0.37 to 3.85) | 0.23 to 5.92 | 1.51 (0.38 to 3.89) | 2 (1397) | Cohorts |
| ZDV+3TCvs 3TC+d4T | 3.55 (0.23 to 135.30) | 0.18 to 165.40 | - | - | - |
| ZDV+3TC+NFV vs NoT/PLC | 0.38 (0.00 to 10.52) | 0.00 to 13.11 | 0.73 (0.00 to 24.05) | 1 (15) | Cohort |
| ZDV+3TC+NFV vs ZDV | 0.44 (0.00 to 12.46) | 0.00 to 15.50 | 0.30 (0.00 to 7.26) | 1 (13) | Cohort |
| ZDV+3TC+NFV vs 3TC+d4T | 1.15 (0.00 to 128.80) | 0.00 to 156.10 | - | - | - |
| ZDV+3TC+NFV vsZDV+3TC | 0.31 (0.00 to 11.58) | 0.00 to 14.26 | - | - | - |
| ZDV+ddI+NVP vs NoT/PLC | 2.00 (0.17 to 17.00) | 0.13 to 22.63 | - | - | - |
| ZDV+ddI+NVP vs ZDV | 2.34 (0.22 to 17.86) | 0.17 to 23.13 | 2.32 (0.27 to 12.20) | 1 (102) | Cohort |
| ZDV+ddI+NVP vs 3TC+d4T | 5.87 (0.34 to 226.30) | 0.28 to 281.80 | 5.69 (0.41 to 169.60) | 1 (38) | Cohort |
| ZDV+ddI+NVP vsZDV+3TC | 1.60 (0.13 to 18.52) | 0.11 to 24.92 | - | - | - |
| ZDV+ddI+NVP vs ZDV+3TC+NFV | 5.21 (0.09 to 4996.00) | 0.08 to 5613.00 | - | - | - |
| ddI+d4T+NVP vs NoT/PLC | 2.00 (0.16 to 15.95) | 0.11 to 21.46 | - | - | - |
| ddI+d4T+NVP vs ZDV | 2.34 (0.19 to 16.98) | 0.15 to 22.14 | 2.33 (0.26 to 12.65) | 1 (102) | Cohort |
| ddI+d4T+NVP vs 3TC+d4T | 5.74 (0.31 to 237.40) | 0.24 to 271.70 | 5.49 (0.38 to 178.10) | 1 (38) | Cohort |
| ddI+d4T+NVP vsZDV+3TC | 1.59 (0.12 to 17.92) | 0.10 to 24.35 | - | - | - |
| ddI+d4T+NVP vs ZDV+3TC+NFV | 5.27 (0.08 to 3456.00) | 0.07 to 4076.00 | - | - | - |
| ddI+d4T+NVP vs ZDV+ddI+NVP | 1.00 (0.06 to 12.52) | 0.05 to 15.49 | 1.00 (0.08 to 11.36) | 1 (26) | Cohort |
| ZDV+3TC+NVP vs NoT/PLC | 0.40 (0.00 to 9.85) | 0.00 to 12.39 | 0.73 (0.00 to 27.44) | 1 (15) | Cohort |
| ZDV+3TC+NVP vs ZDV | 0.47 (0.00 to 11.54) | 0.00 to 13.58 | 0.27 (0.00 to 7.20) | 1 (13) | Cohort |
| ZDV+3TC+NVP vs 3TC+d4T | 1.16 (0.00 to 137.40) | 0.00 to 154.20 | - | - | - |
| ZDV+3TC+NVP vsZDV+3TC | 0.32 (0.00 to 10.90) | 0.00 to 12.87 | - | - | - |
| ZDV+3TC+NVP vs ZDV+3TC+NFV | 1.03 (0.00 to 976.50) | 0.00 to 1093.00 | 0.92 (0.00 to 902.30) | 1 (6) | Cohort |
| ZDV+3TC+NVP vs ZDV+ddI+NVP | 0.19 (0.00 to 12.10) | 0.00 to 13.80 | - | - | - |
| ZDV+3TC+NVP vs ddI+d4T+NVP | 0.19 (0.00 to 12.46) | 0.00 to 14.18 | - | - | - |
| *Common within-network between-study variance* | 0.13 (0.00 to 1.66) | - | 0.12 (0.00 to 1.58) | 9 (2808) | RCTs/Cohorts |
| *Design-by-treatment interaction model for inconsistency χ² (d.f., P-value, between-study variance)* | 2.62 (3,0.45, 0.00) | |  |  | |
| *Model fit measures and diagnostics* Residual deviance = 22.85 Data points = 23 Effective number of parameters = 18.78 DIC = 41.63 | | | | | |
| ***Outcome: Minor Congenital Malformations - # 2 studies (Cohorts) # 69 patients, # 5 treatments*** | | | | | |
| ZDV vs NoT/PLC | 6.00 (0.46 to 298.30) | 0.25 to 461.90 | 5.91 (0.46 to 211.00) | 2 (55) | Cohort |
| ZDV+3TC vs NoT/PLC | 1.05 (0.00 to 151.70) | 0.00 to 220.40 | 0.78 (0.00 to 460.10) | 1 (14) | Cohort |
| ZDV+3TC vs ZDV | 0.18 (0.00 to 5.59) | 0.00 to 9.64 | 0.20 (0.00 to 4.02) | 1 (35) | Cohort |
| ZDV+3TC+NFV vs NoT/PLC | 1.86 (0.00 to 214.60) | 0.00 to 330.10 | 4.55 (0.01 to 3911.00) | 1 (15) | Cohort |
| ZDV+3TC+NFV vs ZDV | 0.30 (0.00 to 13.42) | 0.00 to 22.12 | 0.26 (0.00 to 7.82) | 1 (13) | Cohort |
| ZDV+3TC+NFV vs ZDV+3TC | 1.70 (0.00 to 2203.00) | 0.00 to 2865.00 | - | - | - |
| ZDV+3TC+NVP vs NoT/PLC | 1.90 (0.00 to 236.00) | 0.00 to 349.80 | 4.20 (0.01 to 3014.00) | 1 (15) | Cohort |
| ZDV+3TC+NVP vs ZDV | 0.30 (0.00 to 14.19) | 0.00 to 22.65 | 0.26 (0.00 to 7.36) | 1 (13) | Cohort |
| ZDV+3TC+NVP vs ZDV+3TC | 1.69 (0.00 to 2913.00) | 0.00 to 3819.00 | 0.00 (0.00 to 0.00) | - | - |
| ZDV+3TC+NVP vs ZDV+3TC+NFV | 1.02 (0.00 to 864.80) | 0.00 to 1064.00 | 0.97 (0.00 to 850.10) | 1 (6) | Cohort |
| *Common within-network between-study variance* | 0.40 (0.00 to 4.48) | - | 0.39 (0.00 to 4.51) | 2 (69) | Cohort |
| *Design-by-treatment interaction model for inconsistency χ² (d.f., P-value, between-study variance)* | 0.37 (1, 0.54, NA†) | |  |  | |
| *Model fit measures and diagnostics* Residual deviance = 7.56 Data points = 7 Effective number of parameters = 7.39 DIC = 14.94 | | | | | |
| ***Outcome: MTCT - #11 studies (8 Cohorts + 3 RCTs; 9 2-arm, 3 3-arm) , # 10786 patients, # 6 treatments*** | | | | | |
| [NoT]+[ZDV] vs [NoT/PLC]+[NoT/PLC] | 0.95 (0.22 to 3.86) | 0.16 to 5.53 | - | - | - |
| [ZDV]+[NoT] vs [NoT/PLC]+[NoT/PLC] | **0.39 (0.19 to 0.83)** | **0.11 to 1.45** | **0.36 (0.14 to 0.80)** | 2 (3395) | Cohorts |
| [ZDV]+[NoT] vs [NoT]+[ZDV] | 0.42 (0.10 to 1.84) | 0.07 to 2.46 | 0.88 (0.21 to 4.51) | 1 (102) | Cohort |
| [ZDV]+[ZDV] vs [NoT/PLC]+[NoT/PLC] | **0.43 (0.21 to 0.68)** | **0.11 to 1.23** | **0.46 (0.20 to 0.74)** | 6 (1128) | RCTs/Cohorts |
| [ZDV]+[ZDV] vs [NoT]+[ZDV] | 0.45 (0.11 to 1.77) | 0.07 to 2.41 | 0.37 (0.08 to 1.58) | 2 (169) | Cohorts |
| [ZDV]+[ZDV] vs [ZDV]+[NoT] | 1.10 (0.41 to 2.17) | 0.24 to 3.56 | 0.89 (0.25 to 2.93) | 2 (243) | Cohorts |
| [NoT]+[NVP] vs [NoT/PLC]+[NoT/PLC] | 0.43 (0.18 to 1.04) | 0.11 to 1.72 | 0.43 (0.18 to 1.02) | 2 (5453) | Cohorts |
| [NoT]+[NVP] vs [NoT]+[ZDV] | 0.46 (0.09 to 2.49) | 0.07 to 3.40 | - | - | - |
| [NoT]+[NVP] vs [ZDV]+[NoT] | 1.10 (0.35 to 3.44) | 0.24 to 5.35 | - | - | - |
| [NoT]+[NVP] vs [ZDV]+[ZDV] | 0.99 (0.41 to 3.42) | 0.28 to 5.35 | - | - | - |
| [ZDV]+[NVP] vs [NoT/PLC]+[NoT/PLC] | 0.35 (0.13 to 1.00) | 0.08 to 1.55 | 0.35 (0.28 to 0.44) | 1 (3717) | Cohort |
| [ZDV]+[NVP] vs [NoT]+[ZDV] | 0.38 (0.07 to 2.28) | 0.05 to 3.06 | - | - | - |
| [ZDV]+[NVP] vs [ZDV]+[NoT] | 0.90 (0.26 to 3.21) | 0.18 to 4.84 | - | - | - |
| [ZDV]+[NVP] vs [ZDV]+[ZDV] | 0.81 (0.30 to 3.22) | 0.20 to 5.03 | - | - | - |
| [ZDV]+[NVP] vs [NoT]+[NVP] | 0.82 (0.29 to 2.31) | 0.18 to 3.57 | 0.82 (0.62 to 1.07) | 1 (1589) | Cohort |
| *Common within-network between-study variance* | 0.12 (0.00 to 1.22) | - | 0.11 (0.00 to 1.24) | 11 (10786) | RCTs/Cohorts |
| *Design-by-treatment interaction model for inconsistency χ² (d.f., P-value, between-study variance)* | 4.08 (5, 0.54, 0.06) | |  |  | |
| *Model fit measures and diagnostics*  Residual deviance= 27.5 Data points= 25 Effective number of parameters= 19.5 DIC= 47 | | | | | |
| ***Outcome: Infant and child Deaths - # 13 studies (5 Cohorts + 8 RCTs; 11 2-arm, 2 3-arm), # 11385 patients, # 8 treatments*** | | | | | |
| ZDV vs NoT/PLC | 0.49 (0.21 to 1.36) | 0.05 to 5.43 | 0.46 (0.19 to 1.37) | 8 (7458) | RCTs/Cohorts |
| TDF vs NoT/PLC | 0.64 (0.06 to 7.00) | 0.03 to 15.40 | 0.63 (0.19 to 2.11) | 1 (173) | Cohort |
| TDF vs ZDV | 1.29 (0.09 to 15.44) | 0.04 to 35.26 | - | - | - |
| ZDV+3TC vs NoT/PLC | 0.35 (0.07 to 1.51) | 0.02 to 4.39 | 0.31 (0.05 to 1.52) | 2 (3107) | RCTs/Cohorts |
| ZDV+3TC vs ZDV | 0.73 (0.11 to 3.16) | 0.04 to 8.68 | 0.50 (0.11 to 1.44) | 1 (5191) | Cohort |
| ZDV+3TC vs TDF | 0.56 (0.03 to 8.86) | 0.02 to 19.25 | - | - | - |
| ZDV+3TC+ABC vs NoT/PLC | 0.24 (0.01 to 7.13) | 0.01 to 12.83 | - | - | - |
| ZDV+3TC+ABC vs ZDV | 0.50 (0.02 to 11.37) | 0.01 to 19.78 | - | - | - |
| ZDV+3TC+ABC vs TDF | 0.38 (0.01 to 25.16) | 0.00 to 39.36 | - | - | - |
| ZDV+3TC+ABC vs ZDV+3TC | 0.69 (0.02 to 28.66) | 0.01 to 50.54 | - | - | - |
| NVP vs NoT/PLC | 0.04 (0.00 to 2.09) | 0.00 to 3.68 | - | - | - |
| NVP vs ZDV | 0.09 (0.00 to 3.68) | 0.00 to 6.79 | 0.10 (0.00 to 1.97) | 1 (60) | RCT |
| NVP vs TDF | 0.07 (0.00 to 6.75) | 0.00 to 10.36 | - | - | - |
| NVP vs ZDV+3TC | 0.12 (0.00 to 8.35) | 0.00 to 13.56 | - | - | - |
| NVP vs ZDV+3TC+ABC | 0.17 (0.00 to 21.97) | 0.00 to 32.65 | - | - | - |
| ZDV+3TC+NVP vs NoT/PLC | 0.30 (0.01 to 9.30) | 0.01 to 17.03 | - | - | - |
| ZDV+3TC+NVP vs ZDV | 0.62 (0.03 to 14.99) | 0.01 to 26.34 | - | - | - |
| ZDV+3TC+NVP vs TDF | 0.47 (0.01 to 33.19) | 0.01 to 57.13 | - | - | - |
| ZDV+3TC+NVP vs ZDV+3TC | 0.86 (0.03 to 38.97) | 0.02 to 70.10 | - | - | - |
| ZDV+3TC+NVP vs ZDV+3TC+ABC | 1.25 (0.13 to 12.52) | 0.06 to 28.68 | 1.26 (0.50 to 3.01) | 1 (439) | RCT |
| ZDV+3TC+NVP vs NVP | 7.39 (0.05 to 7067.00) | 0.03 to 9286.00 | - | - | - |
| ZDV+3TC+LOP+RIT vs NoT/PLC | 0.29 (0.03 to 3.55) | 0.01 to 7.94 | - | - | - |
| ZDV+3TC+LOP+RIT vs ZDV | 0.61 (0.07 to 5.43) | 0.03 to 12.53 | 0.61 (0.35 to 1.03) | 1 (805) | RCT |
| ZDV+3TC+LOP+RIT vs TDF | 0.46 (0.02 to 15.32) | 0.01 to 27.56 | - | - | - |
| ZDV+3TC+LOP+RIT vs ZDV+3TC | 0.84 (0.07 to 15.89) | 0.03 to 34.24 | - | - | - |
| ZDV+3TC+LOP+RIT vs ZDV+3TC+ABC | 1.23 (0.13 to 11.32) | 0.06 to 25.61 | 1.23 (0.57 to 2.68) | 1 (553) | RCT |
| ZDV+3TC+LOP+RIT vs NVP | 7.03 (0.09 to 4740.00) | 0.06 to 6418.00 | - | - | - |
| ZDV+3TC+LOP+RIT vs ZDV+3TC+NVP | 0.98 (0.10 to 9.50) | 0.04 to 22.14 | 0.98 (0.42 to 2.41) | 1 (426) | RCT |
| *Common within-network between-study variance* | 0.83 (0.18 to 3.41) | - | 0.94 (0.22 to 3.65) | 13 (11385) | RCTs/ Cohorts |
| *Design-by-treatment interaction model for inconsistency χ² (d.f., P-value, between-study variance)* | 3.47 (2, 0.18, 0.48) | |  |  |  |
| *Model fit measures and diagnostics*  Residual deviance= 31.98 Data points= 28 Effective number of parameters= 26.16 DIC= 58.14 | | | | | |
| ***Outcome: Preterm Births - # 35 studies (26 Cohorts + 1 case-control + 8 RCTs), # 20576 patients, # 17 treatments*** | | | | | |
| ZDV vs NoT/PLC | **0.64 (0.47 to 0.91)** | **0.19 to 2.19** | **0.64 (0.45 to 0.94)** | 19 (16425) | RCTs/Cohorts |
| TDF vs NoT/PLC | 0.84 (0.19 to 3.88) | 0.13 to 5.56 | 0.83 (0.33 to 2.22) | 1 (212) | RCT |
| TDF vs ZDV | 1.30 (0.28 to 6.18) | 0.19 to 9.33 | - | - | - |
| ZDV+3TC+IND vs NoT/PLC | **69.59 (2.49 to 4982.00)** | **1.99 to 5648.00** | - | - | - |
| ZDV+3TC+IND vs ZDV | **108.20 (3.81 to 7553.00)** | **3.06 to 8579.00** | - | - | - |
| ZDV+3TC+IND vs TDF | **85.14 (2.05 to 7532.00)** | **1.73 to 8697.00** | - | - | - |
| ZDV+3TC vs NoT/PLC | 1.24 (0.30 to 5.41) | 0.20 to 8.08 | - | - | - |
| ZDV+3TC vs ZDV | 1.91 (0.48 to 7.98) | 0.31 to 12.04 | 2.20 (0.91 to 5.23) | 1 (108) | Cohort |
| ZDV+3TC vs TDF | 1.47 (0.18 to 11.82) | 0.13 to 16.57 | - | - | - |
| ZDV+3TC vs ZDV+3TC+IND | **0.02 (0.00 to 0.43)** | **0.00 to 0.54** | **0.01 (0.00 to 0.25)** | 1 (17) | Cohort |
| 3TC+d4T+IND vs NoT/PLC | 2.46 (0.00 to 239.80) | 0.00 to 273.00 | - | - | - |
| 3TC+d4T+IND vs ZDV | 3.82 (0.01 to 375.20) | 0.01 to 446.40 | - | - | - |
| 3TC+d4T+IND vs TDF | 2.89 (0.00 to 361.00) | 0.00 to 405.60 | - | - | - |
| 3TC+d4T+IND vs ZDV+3TC+IND | 0.03 (0.00 to 1.77) | 0.00 to 2.07 | 0.03 (0.00 to 1.34) | 1 (6) | Cohort |
| 3TC+d4T+IND vs ZDV+3TC | 2.01 (0.00 to 169.40) | 0.00 to 196.90 | 4.51 (0.01 to 3242.00) | 1 (17) | Cohort |
| ZDV+3TC+ABC vs NoT/PLC | 0.57 (0.17 to 1.89) | 0.11 to 3.06 | 0.27 (0.00 to 9.80) | 1 (13) | Cohort |
| ZDV+3TC+ABC vs ZDV | 0.89 (0.26 to 2.85) | 0.16 to 4.70 | 0.67 (0.00 to 11.90) | 1 (70) | Cohort |
| ZDV+3TC+ABC vs TDF | 0.68 (0.10 to 4.65) | 0.07 to 6.36 | - | - | - |
| ZDV+3TC+ABC vs ZDV+3TC+IND | **0.01 (0.00 to 0.26)** | **0.00 to 0.34** | - | - | - |
| ZDV+3TC+ABC vs ZDV+3TC | 0.46 (0.07 to 2.82) | 0.05 to 3.98 | - | - | - |
| ZDV+3TC+ABC vs 3TC+d4T+IND | 0.23 (0.00 to 146.00) | 0.00 to 152.20 | - | - | - |
| 3TC+d4T+EFV vs NoT/PLC | 3.10 (0.38 to 25.05) | 0.28 to 32.85 | - | - | - |
| 3TC+d4T+EFV vs ZDV | 4.82 (0.56 to 39.55) | 0.44 to 52.49 | - | - | - |
| 3TC+d4T+EFV vs TDF | 3.68 (0.29 to 48.51) | 0.23 to 63.09 | - | - | - |
| 3TC+d4T+EFV vs ZDV+3TC+IND | 0.04 (0.00 to 2.47) | 0.00 to 2.89 | - | - | - |
| 3TC+d4T+EFV vs ZDV+3TC | 2.51 (0.20 to 31.74) | 0.16 to 41.04 | - | - | - |
| 3TC+d4T+EFV vs 3TC+d4T+IND | 1.29 (0.01 to 963.90) | 0.01 to 1046.00 | - | - | - |
| 3TC+d4T+EFV vs ZDV+3TC+ABC | 5.52 (0.47 to 62.96) | 0.37 to 81.35 | - | - | - |
| ZDV+3TC+NLF vs NoT/PLC | 0.62 (0.22 to 1.75) | 0.13 to 3.05 | 0.55 (0.13 to 2.39) | 2 (150) | Cohorts |
| ZDV+3TC+NLF vs ZDV | 0.97 (0.35 to 2.70) | 0.20 to 4.68 | 3.33 (0.00 to 2118.00) | 1 (13) | Cohort |
| ZDV+3TC+NLF vs TDF | 0.74 (0.12 to 4.59) | 0.09 to 6.51 | - | - | - |
| ZDV+3TC+NLF vs ZDV+3TC+IND | **0.01 (0.00 to 0.26)** | **0.00 to 0.34** | **0.02 (0.00 to 0.64)** | 1 (9) | Cohort |
| ZDV+3TC+NLF vs ZDV+3TC | 0.50 (0.09 to 2.76) | 0.06 to 4.01 | **2.47 (0.00 to 1801.00)** | 1 (20) | Cohort |
| ZDV+3TC+NLF vs 3TC+d4T+IND | 0.25 (0.00 to 147.80) | 0.00 to 173.60 | **0.53 (0.00 to 352.90)** | 1 (9) | Cohort |
| ZDV+3TC+NLF vs ZDV+3TC+ABC | 1.10 (0.30 to 4.11) | 0.20 to 6.33 | 0.48 (0.00 to 257.50) | 1 (6) | Cohort |
| ZDV+3TC+NLF vs 3TC+d4T+EFV | 0.20 (0.02 to 2.14) | 0.02 to 2.69 | - | - | - |
| ZDV+NVP vs NoT/PLC | **0.00 (0.00 to 0.02)** | **0.00 to 0.03** | 0.00 (0.00 to 0.01) | 1 (357) | Cohort |
| ZDV+NVP vs ZDV | **0.01 (0.00 to 0.04)** | **0.00 to 0.05** | - | - | - |
| ZDV+NVP vs TDF | **0.00 (0.00 to 0.05)** | **0.00 to 0.07** | - | - | - |
| ZDV+NVP vs ZDV+3TC+IND | **0.00 (0.00 to 0.00)** | **0.00 to 0.00** | - | - | - |
| ZDV+NVP vs ZDV+3TC | **0.00 (0.00 to 0.03)** | **0.00 to 0.04** | - | - | - |
| ZDV+NVP vs 3TC+d4T+IND | 0.00 (0.00 to 1.04) | 0.00 to 1.10 | - | - | - |
| ZDV+NVP vs ZDV+3TC+ABC | **0.01 (0.00 to 0.06)** | **0.00 to 0.08** | - | - | - |
| ZDV+NVP vs 3TC+d4T+EFV | **0.00 (0.00 to 0.02)** | **0.00 to 0.03** | - | - | - |
| ZDV+NVP vs ZDV+3TC+NLF | **0.01 (0.00 to 0.05)** | **0.00 to 0.07** | - | - | - |
| 3TC+d4T+NVP vs NoT/PLC | 2.69 (0.49 to 14.20) | 0.36 to 20.52 | 2.67 (0.79 to 8.99) | 1 (69) | Cohort |
| 3TC+d4T+NVP vs ZDV | 4.18 (0.75 to 22.29) | 0.51 to 32.21 | - | - | - |
| 3TC+d4T+NVP vs TDF | 3.17 (0.34 to 30.00) | 0.25 to 39.29 | - | - | - |
| 3TC+d4T+NVP vs ZDV+3TC+IND | 0.04 (0.00 to 1.69) | 0.00 to 1.96 | - | - | - |
| 3TC+d4T+NVP vs ZDV+3TC | 2.18 (0.24 to 19.63) | 0.18 to 26.87 | - | - | - |
| 3TC+d4T+NVP vs 3TC+d4T+IND | 1.10 (0.01 to 711.20) | 0.01 to 887.20 | - | - | - |
| 3TC+d4T+NVP vs ZDV+3TC+ABC | 4.76 (0.57 to 36.71) | 0.44 to 50.16 | - | - | - |
| 3TC+d4T+NVP vs 3TC+d4T+EFV | 0.86 (0.24 to 3.14) | 0.15 to 4.96 | **0.85 (0.50 to 1.51)** | 1 (395) | Cohort |
| 3TC+d4T+NVP vs ZDV+3TC+NLF | 4.29 (0.59 to 29.77) | 0.43 to 41.40 | - | - | - |
| 3TC+d4T+NVP vs ZDV+NVP | **659.30 (57.44 to 10400.00)** | **44.21 to 12930.00** | - | - | - |
| ZDV+3TC+NVP vs NoT/PLC | **0.38 (0.15 to 0.99)** | **0.08 to 1.77** | 0.73 (0.00 to 28.96) | 1 (15) | Cohort |
| ZDV+3TC+NVP vs ZDV | 0.60 (0.23 to 1.50) | 0.14 to 2.64 | 0.66 (0.16 to 2.61) | 3 (208) | Cohorts |
| ZDV+3TC+NVP vs TDF | 0.45 (0.08 to 2.71) | 0.05 to 3.89 | - | - | - |
| ZDV+3TC+NVP vs ZDV+3TC+IND | **0.01 (0.00 to 0.17)** | **0.00 to 0.21** | - | - | - |
| ZDV+3TC+NVP vs ZDV+3TC | 0.31 (0.06 to 1.65) | 0.04 to 2.47 | - | - | - |
| ZDV+3TC+NVP vs 3TC+d4T+IND | 0.16 (0.00 to 89.56) | 0.00 to 107.20 | - | - | - |
| ZDV+3TC+NVP vs ZDV+3TC+ABC | 0.67 (0.22 to 2.15) | 0.13 to 3.53 | 0.65 (0.34 to 1.18) | 1 (439) | RCT |
| ZDV+3TC+NVP vs 3TC+d4T+EFV | 0.12 (0.01 to 1.27) | 0.01 to 1.69 | - | - | - |
| ZDV+3TC+NVP vs ZDV+3TC+NLF | 0.61 (0.22 to 1.72) | 0.13 to 2.86 | 0.62 (0.13 to 2.91) | 2 (149) | Cohorts |
| ZDV+3TC+NVP vs ZDV+NVP | **93.02 (12.73 to 1040.00)** | **8.99 to 1331.00** | - | - | - |
| ZDV+3TC+NVP vs 3TC+d4T+NVP | 0.14 (0.02 to 1.01) | 0.02 to 1.36 | - | - | - |
| LOP+RIT vs NoT/PLC | 0.90 (0.14 to 6.99) | 0.10 to 9.34 | - | - | - |
| LOP+RIT vs ZDV | 1.39 (0.21 to 10.55) | 0.15 to 14.30 | - | - | - |
| LOP+RIT vs TDF | 1.07 (0.09 to 13.41) | 0.07 to 17.25 | - | - | - |
| LOP+RIT vs ZDV+3TC+IND | **0.01 (0.00 to 0.64)** | **0.00 to 0.74** | - | - | - |
| LOP+RIT vs ZDV+3TC | 0.73 (0.07 to 8.12) | 0.05 to 10.61 | - | - | - |
| LOP+RIT vs 3TC+d4T+IND | 0.38 (0.00 to 252.50) | 0.00 to 302.00 | - | - | - |
| LOP+RIT vs ZDV+3TC+ABC | 1.57 (0.21 to 13.50) | 0.15 to 17.85 | - | - | - |
| LOP+RIT vs 3TC+d4T+EFV | 0.29 (0.02 to 5.56) | 0.01 to 7.16 | - | - | - |
| LOP+RIT vs ZDV+3TC+NLF | 1.44 (0.20 to 11.24) | 0.14 to 15.37 | - | - | - |
| LOP+RIT vs ZDV+NVP | **221.50 (16.24 to 4203.00)** | **12.37 to 5409.00** | - | - | - |
| LOP+RIT vs 3TC+d4T+NVP | 0.33 (0.03 to 4.83) | 0.02 to 6.17 | - | - | - |
| LOP+RIT vs ZDV+3TC+NVP | 2.33 (0.32 to 18.89) | 0.23 to 23.50 | - | - | - |
| ZDV+3TC+LOP+RIT vs NoT/PLC | 0.96 (0.42 to 2.23) | 0.23 to 4.07 | 1.11 (0.48 to 2.73) | 1 (125) | Cohort |
| ZDV+3TC+LOP+RIT vs ZDV | 1.49 (0.66 to 3.36) | 0.35 to 6.19 | 1.22 (0.79 to 1.87) | 1 (805) | RCT |
| ZDV+3TC+LOP+RIT vs TDF | 1.14 (0.20 to 6.44) | 0.15 to 9.20 | - | - | - |
| ZDV+3TC+LOP+RIT vs ZDV+3TC+IND | **0.01 (0.00 to 0.42)** | **0.00 to 0.51** | - | - | - |
| ZDV+3TC+LOP+RIT vs ZDV+3TC | 0.78 (0.15 to 3.82) | 0.11 to 5.51 | - | - | - |
| ZDV+3TC+LOP+RIT vs 3TC+d4T+IND | 0.39 (0.00 to 228.30) | 0.00 to 250.80 | - | - | - |
| ZDV+3TC+LOP+RIT vs ZDV+3TC+ABC | 1.69 (0.59 to 4.99) | 0.35 to 8.21 | 1.92 (0.56 to 7.02) | 2 (557) | RCTs |
| ZDV+3TC+LOP+RIT vs 3TC+d4T+EFV | 0.31 (0.03 to 2.99) | 0.03 to 4.11 | - | - | - |
| ZDV+3TC+LOP+RIT vs ZDV+3TC+NLF | 1.55 (0.58 to 3.94) | 0.34 to 6.84 | 1.82 (0.51 to 6.37) | 3 (194) | Case-control/Cohorts |
| ZDV+3TC+LOP+RIT vs ZDV+NVP | **234.30 (33.52 to 2554.00)** | **24.49 to 3202.00** | - | - | - |
| ZDV+3TC+LOP+RIT vs 3TC+d4T+NVP | 0.36 (0.06 to 2.39) | 0.04 to 3.37 | - | - | - |
| ZDV+3TC+LOP+RIT vs ZDV+3TC+NVP | **2.51 (1.00 to 6.39)** | **0.58 to 11.18** | **2.61 (1.47 to 4.81)** | 1 (426) | RCT |
| ZDV+3TC+LOP+RIT vs LOP+RIT | 1.08 (0.17 to 6.03) | 0.12 to 8.47 | 1.07 (0.26 to 3.97) | 1 (105) | RCT |
| 3TC+d4T+LOP+RIT vs NoT/PLC | 0.85 (0.11 to 6.74) | 0.07 to 9.05 | - | - | - |
| 3TC+d4T+LOP+RIT vs ZDV | 1.33 (0.16 to 10.85) | 0.12 to 14.08 | - | - | - |
| 3TC+d4T+LOP+RIT vs TDF | 1.02 (0.08 to 12.96) | 0.06 to 15.97 | - | - | - |
| 3TC+d4T+LOP+RIT vs ZDV+3TC+IND | **0.01 (0.00 to 0.67)** | **0.00 to 0.77** | - | - | - |
| 3TC+d4T+LOP+RIT vs ZDV+3TC | 0.70 (0.05 to 8.58) | 0.04 to 10.94 | - | - | - |
| 3TC+d4T+LOP+RIT vs 3TC+d4T+IND | 0.36 (0.00 to 259.30) | 0.00 to 279.40 | - | - | - |
| 3TC+d4T+LOP+RIT vs ZDV+3TC+ABC | 1.51 (0.13 to 16.52) | 0.10 to 20.71 | - | - | - |
| 3TC+d4T+LOP+RIT vs 3TC+d4T+EFV | 0.28 (0.07 to 1.02) | 0.05 to 1.64 | **0.27 (0.15 to 0.50)** | 1 (533) | Cohort |
| 3TC+d4T+LOP+RIT vs ZDV+3TC+NLF | 1.38 (0.13 to 13.58) | 0.10 to 18.26 | - | - | - |
| 3TC+d4T+LOP+RIT vs ZDV+NVP | **215.60 (13.58 to 4401.00)** | **10.98 to 5309.00** | - | - | - |
| 3TC+d4T+LOP+RIT vs 3TC+d4T+NVP | 0.32 (0.09 to 1.15) | 0.06 to 1.83 | **0.32 (0.20 to 0.52)** | 1 (704) | Cohort |
| 3TC+d4T+LOP+RIT vs ZDV+3TC+NVP | 2.25 (0.22 to 22.13) | 0.16 to 28.94 | - | - | - |
| 3TC+d4T+LOP+RIT vs LOP+RIT | 0.97 (0.05 to 15.93) | 0.04 to 20.34 | - | - | - |
| 3TC+d4T+LOP+RIT vs ZDV+3TC+LOP+RIT | 0.89 (0.09 to 8.24) | 0.07 to 10.78 | - | - | - |
| ZDV+3TC+LOP+RIT+TDF vs NoT/PLC | 0.05 (0.00 to 1.51) | 0.00 to 1.91 | - | - | - |
| ZDV+3TC+LOP+RIT+TDF vs ZDV | 0.08 (0.00 to 2.30) | 0.00 to 2.83 | 0.09 (0.00 to 5.45) | 1 (4) | Cohort |
| ZDV+3TC+LOP+RIT+TDF vs TDF | 0.06 (0.00 to 2.52) | 0.00 to 3.10 | - | - | - |
| ZDV+3TC+LOP+RIT+TDF vs ZDV+3TC+IND | **0.00 (0.00 to 0.09)** | **0.00 to 0.10** | - | - | - |
| ZDV+3TC+LOP+RIT+TDF vs ZDV+3TC | 0.04 (0.00 to 1.67) | 0.00 to 2.10 | - | - | - |
| ZDV+3TC+LOP+RIT+TDF vs 3TC+d4T+IND | 0.02 (0.00 to 22.78) | 0.00 to 23.80 | - | - | - |
| ZDV+3TC+LOP+RIT+TDF vs ZDV+3TC+ABC | 0.09 (0.00 to 2.99) | 0.00 to 3.62 | - | - | - |
| ZDV+3TC+LOP+RIT+TDF vs 3TC+d4T+EFV | **0.01 (0.00 to 0.98)** | **0.00 to 1.14** | - | - | - |
| ZDV+3TC+LOP+RIT+TDF vs ZDV+3TC+NLF | 0.08 (0.00 to 2.53) | 0.00 to 3.08 | - | - | - |
| ZDV+3TC+LOP+RIT+TDF vs ZDV+NVP | 11.99 (0.02 to 696.20) | 0.02 to 866.00 | - | - | - |
| ZDV+3TC+LOP+RIT+TDF vs 3TC+d4T+NVP | **0.02 (0.00 to 0.89)** | **0.00 to 1.08** | - | - | - |
| ZDV+3TC+LOP+RIT+TDF vs ZDV+3TC+NVP | 0.13 (0.00 to 3.66) | 0.00 to 4.60 | 0.12 (0.00 to 2.76) | 1 (17) | Cohort |
| ZDV+3TC+LOP+RIT+TDF vs LOP+RIT | 0.05 (0.00 to 2.70) | 0.00 to 3.37 | - | - | - |
| ZDV+3TC+LOP+RIT+TDF vs ZDV+3TC+LOP+RIT | 0.05 (0.00 to 1.60) | 0.00 to 2.02 | - | - | - |
| ZDV+3TC+LOP+RIT+TDF vs 3TC+d4T+LOP+RIT | 0.05 (0.00 to 3.40) | 0.00 to 4.05 | - | - | - |
| ZDV+3TC+EFV vs NoT/PLC | 0.85 (0.18 to 4.01) | 0.13 to 6.00 | - | - | - |
| ZDV+3TC+EFV vs ZDV | 1.33 (0.29 to 6.13) | 0.20 to 8.93 | - | - | - |
| ZDV+3TC+EFV vs TDF | 1.02 (0.12 to 8.68) | 0.09 to 11.74 | - | - | - |
| ZDV+3TC+EFV vs ZDV+3TC+IND | **0.01 (0.00 to 0.46)** | **0.00 to 0.55** | - | - | - |
| ZDV+3TC+EFV vs ZDV+3TC | 0.69 (0.08 to 5.43) | 0.06 to 7.37 | - | - | - |
| ZDV+3TC+EFV vs 3TC+d4T+IND | 0.35 (0.00 to 221.90) | 0.00 to 252.90 | - | - | - |
| ZDV+3TC+EFV vs ZDV+3TC+ABC | 1.50 (0.28 to 8.03) | 0.19 to 11.62 | - | - | - |
| ZDV+3TC+EFV vs 3TC+d4T+EFV | 0.28 (0.02 to 3.73) | 0.02 to 4.87 | - | - | - |
| ZDV+3TC+EFV vs ZDV+3TC+NLF | 1.37 (0.27 to 6.68) | 0.19 to 9.85 | - | - | - |
| ZDV+3TC+EFV vs ZDV+NVP | **208.80 (19.86 to 3000.00)** | **15.21 to 4005.00** | - | - | - |
| ZDV+3TC+EFV vs 3TC+d4T+NVP | 0.32 (0.03 to 3.26) | 0.03 to 4.23 | - | - | - |
| ZDV+3TC+EFV vs ZDV+3TC+NVP | 2.23 (0.46 to 10.94) | 0.33 to 16.19 | - | - | - |
| ZDV+3TC+EFV vs LOP+RIT | 0.96 (0.10 to 8.32) | 0.08 to 11.30 | - | - | - |
| ZDV+3TC+EFV vs ZDV+3TC+LOP+RIT | 0.88 (0.24 to 3.24) | 0.16 to 5.20 | 0.89 (0.50 to 1.60) | 1 (356) | RCT |
| ZDV+3TC+EFV vs 3TC+d4T+LOP+RIT | 1.01 (0.07 to 13.44) | 0.06 to 16.75 | - | - | - |
| ZDV+3TC+EFV vs ZDV+3TC+LOP+RIT+TDF | 18.10 (0.41 to 8737.00) | 0.36 to 9224.00 | - | - | - |
| *Common within-network between-study variance* | 0.31 (0.15 to 0.73) | - | 0.37 (0.16 to 0.89) | 35 (20576) | RCTs/Cohorts/Case-Control |
| *Design-by-treatment interaction model for inconsistency χ² (d.f., P-value, between-study variance)* | 4.3 (13, 0.99, 0.37) | |  |  | |
| *Model fit measures and diagnostics* Residual deviance= 75.27 Data points= 80 Effective number of parameters= 66.93 DIC= 142.20 | | | | | |
| ***Outcome: Stillbirths - # 26 studies (13 Cohorts + 13 RCTs; 19 2-arm, 4 3-arm, 2 4-arm, 1 8-arm), # 17507 patients, # 20 treatments*** | | | | | |
| ZDV vs NoT/PLC | **0.46 (0.24 to 0.93)** | **0.06 to 3.65** | 0.48 (0.23 to 1.02) | 15 (10137) | RCTs/Cohorts |
| d4T vs NoT/PLC | 0.09 (0.00 to 4.99) | 0.00 to 7.45 | - | - | - |
| d4T vs ZDV | 0.19 (0.00 to 9.76) | 0.00 to 14.49 | 0.20 (0.00 to 6.21) | 1 (186) | RCT |
| ddl vs NoT/PLC | 0.09 (0.00 to 4.73) | 0.00 to 6.77 | - | - | - |
| ddl vs ZDV | 0.19 (0.00 to 9.44) | 0.00 to 15.71 | 0.19 (0.00 to 5.88) | 1 (189) | RCT |
| ddl vs d4T | 0.99 (0.00 to 723.10) | 0.00 to 844.80 | 1.02 (0.00 to 650.00) | 1 (187) | RCT |
| d4T+ddI vs NoT/PLC | 1.73 (0.12 to 41.38) | 0.06 to 66.13 | - | - | - |
| d4T+ddI vs ZDV | 3.67 (0.27 to 85.04) | 0.15 to 145.50 | 3.54 (0.59 to 43.05) | 1 (189) | RCT |
| d4T+ddI vs d4T | 19.40 (0.61 to 9094.00) | 0.39 to 11680.00 | 19.43 (1.26 to 8634.00) | 1 (187) | RCT |
| d4T+ddI vs ddl | 19.88 (0.67 to 11030.00) | 0.40 to 13320.00 | 18.58 (1.33 to 6924.00) | 1 (190) | RCT |
| TDF vs NoT/PLC | 2.64 (0.29 to 26.17) | 0.15 to 52.35 | 2.58 (0.92 to 9.46) | 1 (366) | Cohort |
| TDF vs ZDV | 5.70 (0.57 to 62.58) | 0.28 to 123.70 | - | - | - |
| TDF vs d4T | 31.17 (0.31 to 23800.00) | 0.22 to 31990.00 | - | - | - |
| TDF vs ddl | 32.26 (0.32 to 24170.00) | 0.21 to 33650.00 | - | - | - |
| TDF vs d4T+ddI | 1.50 (0.03 to 53.20) | 0.02 to 85.23 | - | - | - |
| ZDV+3TC+IND vs NoT/PLC | 1.44 (0.03 to 34.02) | 0.02 to 56.02 | - | - | - |
| ZDV+3TC+IND vs ZDV | 3.13 (0.06 to 68.15) | 0.04 to 113.90 | - | - | - |
| ZDV+3TC+IND vs d4T | 16.69 (0.06 to 16690.00) | 0.05 to 20510.00 | - | - | - |
| ZDV+3TC+IND vs ddl | 17.21 (0.07 to 18000.00) | 0.05 to 21350.00 | - | - | - |
| ZDV+3TC+IND vs d4T+ddI | 0.76 (0.01 to 49.85) | 0.00 to 72.55 | - | - | - |
| ZDV+3TC+IND vs TDF | 0.54 (0.01 to 24.16) | 0.00 to 39.36 | - | - | - |
| 3TC+d4T vs NoT/PLC | 0.49 (0.00 to 25.68) | 0.00 to 40.12 | - | - | - |
| 3TC+d4T vs ZDV | 1.07 (0.00 to 52.29) | 0.00 to 80.55 | - | - | - |
| 3TC+d4T vs d4T | 5.67 (0.00 to 7833.00) | 0.00 to 9260.00 | - | - | - |
| 3TC+d4T vs ddl | 5.87 (0.00 to 6715.00) | 0.00 to 8162.00 | - | - | - |
| 3TC+d4T vs d4T+ddI | 0.25 (0.00 to 32.45) | 0.00 to 48.47 | - | - | - |
| 3TC+d4T vs TDF | 0.18 (0.00 to 16.59) | 0.00 to 24.53 | - | - | - |
| 3TC+d4T vs ZDV+3TC+IND | 0.35 (0.00 to 42.60) | 0.00 to 62.74 | 0.44 (0.00 to 341.20) | 1 (18) | Cohort |
| ZDV+3TC vs NoT/PLC | 0.51 (0.12 to 1.98) | 0.04 to 5.22 | 0.36 (0.05 to 1.39) | 1 (1501) | RCT |
| ZDV+3TC vs ZDV | 1.09 (0.26 to 4.00) | 0.10 to 10.56 | 1.21 (0.20 to 6.47) | 2 (2092) | RCT/Cohort |
| ZDV+3TC vs d4T | 5.83 (0.09 to 3322.00) | 0.06 to 4460.00 | - | - | - |
| ZDV+3TC vs ddl | 5.90 (0.09 to 3593.00) | 0.06 to 4839.00 | - | - | - |
| ZDV+3TC vs d4T+ddI | 0.29 (0.01 to 5.31) | 0.01 to 9.45 | - | - | - |
| ZDV+3TC vs TDF | 0.19 (0.01 to 2.53) | 0.01 to 4.62 | - | - | - |
| ZDV+3TC vs ZDV+3TC+IND | 0.35 (0.02 to 14.20) | 0.01 to 22.82 | 0.40 (0.02 to 18.20) | 2 (83) | Cohorts |
| ZDV+3TC vs 3TC+d4T | 0.99 (0.02 to 495.80) | 0.01 to 572.50 | 0.19 (0.00 to 132.20) | 1 (70) | Cohort |
| 3TC+d4T+IND vs NoT/PLC | 1.07 (0.00 to 80.11) | 0.00 to 114.90 | - | - | - |
| 3TC+d4T+IND vs ZDV | 2.31 (0.00 to 170.30) | 0.00 to 255.50 | - | - | - |
| 3TC+d4T+IND vs d4T | 12.36 (0.01 to 23750.00) | 0.01 to 29180.00 | - | - | - |
| 3TC+d4T+IND vs ddl | 12.60 (0.01 to 23170.00) | 0.01 to 31740.00 | - | - | - |
| 3TC+d4T+IND vs d4T+ddI | 0.56 (0.00 to 100.20) | 0.00 to 144.20 | - | - | - |
| 3TC+d4T+IND vs TDF | 0.38 (0.00 to 51.13) | 0.00 to 70.81 | - | - | - |
| 3TC+d4T+IND vs ZDV+3TC+IND | 0.79 (0.00 to 94.93) | 0.00 to 135.70 | 0.98 (0.00 to 686.50) | 1 (8) | Cohort |
| 3TC+d4T+IND vs 3TC+d4T | 2.18 (0.00 to 2211.00) | 0.00 to 3136.00 | - | - | - |
| 3TC+d4T+IND vs ZDV+3TC | 2.14 (0.00 to 133.40) | 0.00 to 212.90 | 0.79 (0.00 to 31.87) | 1 (19) | Cohort |
| ZDV+3TC+ABC vs NoT/PLC | 0.86 (0.07 to 8.54) | 0.04 to 16.90 | 0.26 (0.00 to 9.32) | 1 (15) | Cohort |
| ZDV+3TC+ABC vs ZDV | 1.85 (0.15 to 17.63) | 0.08 to 32.90 | 13.18 (0.02 to 7022.00) | 1 (72) | Cohort |
| ZDV+3TC+ABC vs d4T | 10.41 (0.09 to 7303.00) | 0.06 to 8705.00 | - | - | - |
| ZDV+3TC+ABC vs ddl | 10.13 (0.09 to 7516.00) | 0.06 to 9555.00 | - | - | - |
| ZDV+3TC+ABC vs d4T+ddI | 0.49 (0.01 to 16.07) | 0.01 to 22.90 | - | - | - |
| ZDV+3TC+ABC vs TDF | 0.33 (0.01 to 7.72) | 0.01 to 12.95 | - | - | - |
| ZDV+3TC+ABC vs ZDV+3TC+IND | 0.59 (0.01 to 42.32) | 0.01 to 66.48 | - | - | - |
| ZDV+3TC+ABC vs 3TC+d4T | 1.79 (0.02 to 935.20) | 0.01 to 1221.00 | - | - | - |
| ZDV+3TC+ABC vs ZDV+3TC | 1.69 (0.12 to 20.85) | 0.07 to 38.90 | - | - | - |
| ZDV+3TC+ABC vs 3TC+d4T+IND | 0.83 (0.01 to 609.20) | 0.00 to 830.40 | - | - | - |
| ZDV+3TC+NLF vs NoT/PLC | 3.19 (0.16 to 56.85) | 0.09 to 98.94 | - | - | - |
| ZDV+3TC+NLF vs ZDV | 6.91 (0.35 to 119.70) | 0.20 to 199.50 | - | - | - |
| ZDV+3TC+NLF vs d4T | 39.25 (0.25 to 34090.00) | 0.18 to 43940.00 | - | - | - |
| ZDV+3TC+NLF vs ddl | 39.84 (0.28 to 34330.00) | 0.19 to 44120.00 | - | - | - |
| ZDV+3TC+NLF vs d4T+ddI | 1.79 (0.03 to 90.01) | 0.02 to 136.60 | - | - | - |
| ZDV+3TC+NLF vs TDF | 1.21 (0.03 to 44.54) | 0.02 to 72.55 | - | - | - |
| ZDV+3TC+NLF vs ZDV+3TC+IND | 2.20 (0.10 to 101.90) | 0.06 to 150.40 | 2.61 (0.10 to 131.50) | 2 (22) | Cohorts |
| ZDV+3TC+NLF vs 3TC+d4T | 6.47 (0.11 to 3417.00) | 0.08 to 4322.00 | 19.32 (0.47 to 9991.00) | 1 (17) | Cohort |
| ZDV+3TC+NLF vs ZDV+3TC | 6.28 (0.43 to 89.40) | 0.25 to 163.60 | 6.22 (0.35 to 104.60) | 2 (85) | Cohorts |
| ZDV+3TC+NLF vs 3TC+d4T+IND | 2.88 (0.05 to 1550.00) | 0.03 to 2039.00 | 0.47 (0.00 to 230.30) | 1 (11) | Cohort |
| ZDV+3TC+NLF vs ZDV+3TC+ABC | 3.79 (0.11 to 116.00) | 0.07 to 193.10 | - | - | - |
| ZDV+3TC+SAQ vs NoT/PLC | 2.57 (0.00 to 165.30) | 0.00 to 246.40 | - | - | - |
| ZDV+3TC+SAQ vs ZDV | 5.58 (0.01 to 348.20) | 0.01 to 512.10 | - | - | - |
| ZDV+3TC+SAQ vs d4T | 29.94 (0.01 to 41580.00) | 0.01 to 52490.00 | - | - | - |
| ZDV+3TC+SAQ vs ddl | 31.45 (0.02 to 47230.00) | 0.01 to 54970.00 | - | - | - |
| ZDV+3TC+SAQ vs d4T+ddI | 1.34 (0.00 to 194.90) | 0.00 to 294.10 | - | - | - |
| ZDV+3TC+SAQ vs TDF | 0.93 (0.00 to 109.00) | 0.00 to 164.40 | - | - | - |
| ZDV+3TC+SAQ vs ZDV+3TC+IND | 1.89 (0.00 to 247.20) | 0.00 to 346.40 | 2.40 (0.00 to 1721.00) | 1 (9) | Cohort |
| ZDV+3TC+SAQ vs 3TC+d4T | 5.30 (0.00 to 4287.00) | 0.00 to 5924.00 | 5.58 (0.01 to 3373.00) | 1 (15) | Cohort |
| ZDV+3TC+SAQ vs ZDV+3TC | 5.34 (0.01 to 280.60) | 0.01 to 441.60 | 28.34 (0.03 to 17450.00) | 1 (61) | Cohort |
| ZDV+3TC+SAQ vs 3TC+d4T+IND | 2.43 (0.00 to 3261.00) | 0.00 to 3739.00 | - | - | - |
| ZDV+3TC+SAQ vs ZDV+3TC+ABC | 3.01 (0.00 to 272.90) | 0.00 to 395.40 | - | - | - |
| ZDV+3TC+SAQ vs ZDV+3TC+NLF | 0.82 (0.00 to 60.67) | 0.00 to 92.61 | 0.27 (0.00 to 14.11) | 1 (8) | Cohort |
| 3TC+d4T+SAQ vs NoT/PLC | 1.82 (0.00 to 105.40) | 0.00 to 154.50 | - | - | - |
| 3TC+d4T+SAQ vs ZDV | 3.92 (0.00 to 221.00) | 0.00 to 318.10 | - | - | - |
| 3TC+d4T+SAQ vs d4T | 20.68 (0.01 to 29910.00) | 0.01 to 39440.00 | - | - | - |
| 3TC+d4T+SAQ vs ddl | 21.34 (0.01 to 29010.00) | 0.01 to 38450.00 | - | - | - |
| 3TC+d4T+SAQ vs d4T+ddI | 0.93 (0.00 to 141.10) | 0.00 to 189.60 | - | - | - |
| 3TC+d4T+SAQ vs TDF | 0.67 (0.00 to 72.08) | 0.00 to 99.96 | - | - | - |
| 3TC+d4T+SAQ vs ZDV+3TC+IND | 1.26 (0.00 to 157.40) | 0.00 to 217.60 | 1.76 (0.00 to 1694.00) | 1 (10) | Cohort |
| 3TC+d4T+SAQ vs 3TC+d4T | 3.58 (0.00 to 3252.00) | 0.00 to 4163.00 | 3.44 (0.01 to 2393.00) | 1 (16) | Cohort |
| 3TC+d4T+SAQ vs ZDV+3TC | 3.61 (0.00 to 181.60) | 0.00 to 276.20 | 19.23 (0.03 to 14920.00) | 1 (62) | Cohort |
| 3TC+d4T+SAQ vs 3TC+d4T+IND | 1.63 (0.00 to 2345.00) | 0.00 to 2643.00 | - | - | - |
| 3TC+d4T+SAQ vs ZDV+3TC+ABC | 2.02 (0.00 to 182.40) | 0.00 to 275.60 | - | - | - |
| 3TC+d4T+SAQ vs ZDV+3TC+NLF | 0.56 (0.00 to 36.30) | 0.00 to 56.77 | 0.19 (0.00 to 8.94) | 1 (9) | Cohort |
| 3TC+d4T+SAQ vs ZDV+3TC+SAQ | 0.69 (0.00 to 780.30) | 0.00 to 966.10 | 0.67 (0.00 to 396.30) | 1 (7) | Cohort |
| NVP vs NoT/PLC | 0.13 (0.01 to 1.02) | 0.01 to 2.06 | **0.05 (0.00 to 0.83)** | 2 (675) | RCTs |
| NVP vs ZDV | 0.27 (0.02 to 2.02) | 0.01 to 4.15 | 0.41 (0.03 to 4.16) | 2 (1260) | RCTs |
| NVP vs d4T | 1.46 (0.01 to 1003.00) | 0.01 to 1299.00 | - | - | - |
| NVP vs ddl | 1.52 (0.01 to 1040.00) | 0.01 to 1441.00 | - | - | - |
| NVP vs d4T+ddI | 0.07 (0.00 to 2.01) | 0.00 to 3.25 | - | - | - |
| NVP vs TDF | **0.05 (0.00 to 1.00)** | **0.00 to 1.62** | - | - | - |
| NVP vs ZDV+3TC+IND | 0.09 (0.00 to 7.11) | 0.00 to 10.43 | - | - | - |
| NVP vs 3TC+d4T | 0.26 (0.00 to 167.20) | 0.00 to 206.90 | - | - | - |
| NVP vs ZDV+3TC | 0.25 (0.02 to 2.82) | 0.01 to 5.35 | - | - | - |
| NVP vs 3TC+d4T+IND | 0.12 (0.00 to 97.97) | 0.00 to 124.10 | - | - | - |
| NVP vs ZDV+3TC+ABC | 0.15 (0.01 to 3.53) | 0.00 to 5.76 | - | - | - |
| NVP vs ZDV+3TC+NLF | 0.04 (0.00 to 1.42) | 0.00 to 2.15 | - | - | - |
| NVP vs ZDV+3TC+SAQ | 0.05 (0.00 to 44.77) | 0.00 to 55.19 | - | - | - |
| NVP vs 3TC+d4T+SAQ | 0.07 (0.00 to 60.55) | 0.00 to 82.52 | - | - | - |
| ZDV+NVP vs NoT/PLC | **0.04 (0.00 to 0.72)** | **0.00 to 1.27** | **0.05 (0.00 to 0.32)** | 1 (357) | Cohort |
| ZDV+NVP vs ZDV | 0.10 (0.00 to 1.67) | 0.00 to 2.98 | - | - | - |
| ZDV+NVP vs d4T | 0.50 (0.00 to 532.50) | 0.00 to 695.20 | - | - | - |
| ZDV+NVP vs ddl | 0.52 (0.00 to 445.20) | 0.00 to 588.70 | - | - | - |
| ZDV+NVP vs d4T+ddI | 0.02 (0.00 to 1.33) | 0.00 to 1.91 | - | - | - |
| ZDV+NVP vs TDF | **0.02 (0.00 to 0.60)** | **0.00 to 0.97** | - | - | - |
| ZDV+NVP vs ZDV+3TC+IND | 0.03 (0.00 to 3.74) | 0.00 to 5.67 | - | - | - |
| ZDV+NVP vs 3TC+d4T | 0.09 (0.00 to 71.27) | 0.00 to 84.80 | - | - | - |
| ZDV+NVP vs ZDV+3TC | 0.09 (0.00 to 2.09) | 0.00 to 3.44 | - | - | - |
| ZDV+NVP vs 3TC+d4T+IND | 0.04 (0.00 to 41.06) | 0.00 to 53.77 | - | - | - |
| ZDV+NVP vs ZDV+3TC+ABC | 0.05 (0.00 to 2.12) | 0.00 to 3.19 | - | - | - |
| ZDV+NVP vs ZDV+3TC+NLF | **0.01 (0.00 to 0.84)** | **0.00 to 1.20** | - | - | - |
| ZDV+NVP vs ZDV+3TC+SAQ | 0.02 (0.00 to 18.27) | 0.00 to 20.10 | - | - | - |
| ZDV+NVP vs 3TC+d4T+SAQ | 0.02 (0.00 to 28.61) | 0.00 to 33.44 | - | - | - |
| ZDV+NVP vs NVP | 0.34 (0.00 to 13.67) | 0.00 to 22.02 | - | - | - |
| 3TC+d4T+NVP vs NoT/PLC | 2.66 (0.00 to 165.80) | 0.00 to 241.00 | - | - | - |
| 3TC+d4T+NVP vs ZDV | 5.75 (0.01 to 337.70) | 0.01 to 547.80 | - | - | - |
| 3TC+d4T+NVP vs d4T | 28.63 (0.02 to 56420.00) | 0.02 to 65260.00 | - | - | - |
| 3TC+d4T+NVP vs ddl | 31.64 (0.02 to 49500.00) | 0.02 to 60970.00 | - | - | - |
| 3TC+d4T+NVP vs d4T+ddI | 1.39 (0.00 to 208.00) | 0.00 to 280.50 | - | - | - |
| 3TC+d4T+NVP vs TDF | 0.97 (0.00 to 107.50) | 0.00 to 150.30 | - | - | - |
| 3TC+d4T+NVP vs ZDV+3TC+IND | 1.92 (0.00 to 265.00) | 0.00 to 402.20 | 2.39 (0.00 to 2404.00) | 1 (9) | Cohort |
| 3TC+d4T+NVP vs 3TC+d4T | 5.55 (0.01 to 4566.00) | 0.00 to 5226.00 | 5.22 (0.01 to 3163.00) | 1 (15) | Cohort |
| 3TC+d4T+NVP vs ZDV+3TC | 5.39 (0.01 to 288.60) | 0.01 to 458.10 | 27.88 (0.04 to 24540.00) | 1 (61) | Cohort |
| 3TC+d4T+NVP vs 3TC+d4T+IND | 2.51 (0.00 to 3841.00) | 0.00 to 4402.00 | - | - | - |
| 3TC+d4T+NVP vs ZDV+3TC+ABC | 3.08 (0.00 to 299.00) | 0.00 to 439.30 | - | - | - |
| 3TC+d4T+NVP vs ZDV+3TC+NLF | 0.84 (0.00 to 59.43) | 0.00 to 93.55 | 0.27 (0.00 to 14.02) | 1 (8) | Cohort |
| 3TC+d4T+NVP vs ZDV+3TC+SAQ | 1.01 (0.00 to 1270.00) | 0.00 to 1634.00 | 0.99 (0.00 to 851.10) | 1 (6) | Cohort |
| 3TC+d4T+NVP vs 3TC+d4T+SAQ | 1.49 (0.00 to 1350.00) | 0.00 to 1923.00 | 1.42 (0.00 to 934.30) | 1 (7) | Cohort |
| 3TC+d4T+NVP vs NVP | 20.84 (0.03 to 2590.00) | 0.02 to 3666.00 | - | - | - |
| 3TC+d4T+NVP vs ZDV+NVP | 59.74 (0.06 to 13930.00) | 0.05 to 20340.00 | - | - | - |
| ZDV+3TC+NVP vs NoT/PLC | 1.74 (0.14 to 17.67) | 0.07 to 34.87 | - | - | - |
| ZDV+3TC+NVP vs ZDV | 3.72 (0.32 to 36.04) | 0.16 to 71.78 | - | - | - |
| ZDV+3TC+NVP vs d4T | 20.88 (0.17 to 15670.00) | 0.12 to 21660.00 | - | - | - |
| ZDV+3TC+NVP vs ddl | 21.02 (0.17 to 15860.00) | 0.12 to 20340.00 | - | - | - |
| ZDV+3TC+NVP vs d4T+ddI | 1.00 (0.02 to 32.72) | 0.01 to 52.61 | - | - | - |
| ZDV+3TC+NVP vs TDF | 0.65 (0.02 to 15.76) | 0.01 to 25.05 | - | - | - |
| ZDV+3TC+NVP vs ZDV+3TC+IND | 1.24 (0.03 to 76.08) | 0.02 to 114.10 | 0.99 (0.00 to 617.80) | 1 (12) | Cohort |
| ZDV+3TC+NVP vs 3TC+d4T | 3.57 (0.05 to 1800.00) | 0.03 to 2080.00 | 2.23 (0.00 to 1683.00) | 1 (18) | Cohort |
| ZDV+3TC+NVP vs ZDV+3TC | 3.49 (0.27 to 38.77) | 0.14 to 73.66 | 11.71 (0.02 to 7610.00) | 1 (64) | Cohort |
| ZDV+3TC+NVP vs 3TC+d4T+IND | 1.67 (0.02 to 1201.00) | 0.01 to 1489.00 | - | - | - |
| ZDV+3TC+NVP vs ZDV+3TC+ABC | 2.06 (0.27 to 14.88) | 0.12 to 32.02 | 2.21 (0.84 to 5.92) | 1 (455) | RCT |
| ZDV+3TC+NVP vs ZDV+3TC+NLF | 0.55 (0.02 to 14.47) | 0.01 to 24.27 | 0.11 (0.00 to 4.82) | 1 (11) | Cohort |
| ZDV+3TC+NVP vs ZDV+3TC+SAQ | 0.67 (0.01 to 546.60) | 0.00 to 645.60 | 0.39 (0.00 to 290.60) | 1 (9) | Cohort |
| ZDV+3TC+NVP vs 3TC+d4T+SAQ | 0.99 (0.01 to 725.80) | 0.01 to 888.70 | 0.57 (0.00 to 409.20) | 1 (10) | Cohort |
| ZDV+3TC+NVP vs NVP | 14.13 (0.58 to 404.60) | 0.35 to 623.30 | - | - | - |
| ZDV+3TC+NVP vs ZDV+NVP | 40.20 (0.91 to 3112.00) | 0.59 to 4352.00 | - | - | - |
| ZDV+3TC+NVP vs 3TC+d4T+NVP | 0.66 (0.01 to 450.70) | 0.01 to 539.50 | 0.40 (0.00 to 300.60) | 1 (9) | Cohort |
| ZDV+3TC+LOP+RIT vs NoT/PLC | 0.49 (0.06 to 3.81) | 0.03 to 7.87 | - | - | - |
| ZDV+3TC+LOP+RIT vs ZDV | 1.05 (0.13 to 7.37) | 0.06 to 16.52 | 1.02 (0.23 to 4.40) | 1 (813) | RCT |
| ZDV+3TC+LOP+RIT vs d4T | 5.81 (0.06 to 3937.00) | 0.04 to 4836.00 | - | - | - |
| ZDV+3TC+LOP+RIT vs ddl | 5.79 (0.06 to 3820.00) | 0.04 to 4840.00 | - | - | - |
| ZDV+3TC+LOP+RIT vs d4T+ddI | 0.28 (0.01 to 7.18) | 0.00 to 11.52 | - | - | - |
| ZDV+3TC+LOP+RIT vs TDF | 0.18 (0.01 to 3.70) | 0.00 to 6.16 | - | - | - |
| ZDV+3TC+LOP+RIT vs ZDV+3TC+IND | 0.35 (0.01 to 21.88) | 0.01 to 32.49 | - | - | - |
| ZDV+3TC+LOP+RIT vs 3TC+d4T | 1.02 (0.02 to 556.80) | 0.01 to 698.00 | - | - | - |
| ZDV+3TC+LOP+RIT vs ZDV+3TC | 0.97 (0.09 to 9.35) | 0.05 to 19.47 | - | - | - |
| ZDV+3TC+LOP+RIT vs 3TC+d4T+IND | 0.47 (0.00 to 323.10) | 0.00 to 436.80 | - | - | - |
| ZDV+3TC+LOP+RIT vs ZDV+3TC+ABC | 0.58 (0.08 to 4.47) | 0.04 to 9.56 | 0.63 (0.18 to 1.93) | 1 (560) | RCT |
| ZDV+3TC+LOP+RIT vs ZDV+3TC+NLF | 0.15 (0.01 to 4.16) | 0.00 to 7.33 | - | - | - |
| ZDV+3TC+LOP+RIT vs ZDV+3TC+SAQ | 0.19 (0.00 to 148.50) | 0.00 to 187.80 | - | - | - |
| ZDV+3TC+LOP+RIT vs 3TC+d4T+SAQ | 0.28 (0.00 to 228.60) | 0.00 to 271.70 | - | - | - |
| ZDV+3TC+LOP+RIT vs NVP | 3.92 (0.22 to 82.06) | 0.12 to 148.90 | - | - | - |
| ZDV+3TC+LOP+RIT vs ZDV+NVP | 11.00 (0.33 to 766.50) | 0.19 to 1054.00 | - | - | - |
| ZDV+3TC+LOP+RIT vs 3TC+d4T+NVP | 0.19 (0.00 to 128.30) | 0.00 to 160.70 | - | - | - |
| ZDV+3TC+LOP+RIT vs ZDV+3TC+NVP | 0.28 (0.04 to 2.16) | 0.02 to 4.50 | **0.29 (0.09 to 0.85)** | 1 (445) | RCT |
| ZDV+3TC+EFV vs NoT/PLC | 0.59 (0.03 to 13.13) | 0.02 to 22.22 | - | - | - |
| ZDV+3TC+EFV vs ZDV | 1.28 (0.06 to 26.01) | 0.03 to 43.71 | - | - | - |
| ZDV+3TC+EFV vs d4T | 7.46 (0.05 to 6402.00) | 0.03 to 7617.00 | - | - | - |
| ZDV+3TC+EFV vs ddl | 7.47 (0.04 to 6965.00) | 0.03 to 9018.00 | - | - | - |
| ZDV+3TC+EFV vs d4T+ddI | 0.34 (0.00 to 18.04) | 0.00 to 30.44 | - | - | - |
| ZDV+3TC+EFV vs TDF | 0.22 (0.00 to 9.76) | 0.00 to 15.47 | - | - | - |
| ZDV+3TC+EFV vs ZDV+3TC+IND | 0.43 (0.01 to 48.93) | 0.00 to 67.52 | - | - | - |
| ZDV+3TC+EFV vs 3TC+d4T | 1.30 (0.01 to 900.80) | 0.01 to 1246.00 | - | - | - |
| ZDV+3TC+EFV vs ZDV+3TC | 1.20 (0.05 to 29.50) | 0.03 to 49.12 | - | - | - |
| ZDV+3TC+EFV vs 3TC+d4T+IND | 0.60 (0.00 to 577.20) | 0.00 to 713.40 | - | - | - |
| ZDV+3TC+EFV vs ZDV+3TC+ABC | 0.71 (0.04 to 15.65) | 0.02 to 26.08 | - | - | - |
| ZDV+3TC+EFV vs ZDV+3TC+NLF | 0.19 (0.00 to 10.05) | 0.00 to 16.39 | - | - | - |
| ZDV+3TC+EFV vs ZDV+3TC+SAQ | 0.24 (0.00 to 293.30) | 0.00 to 347.80 | - | - | - |
| ZDV+3TC+EFV vs 3TC+d4T+SAQ | 0.36 (0.00 to 387.20) | 0.00 to 476.20 | - | - | - |
| ZDV+3TC+EFV vs NVP | 4.80 (0.12 to 216.50) | 0.08 to 358.90 | - | - | - |
| ZDV+3TC+EFV vs ZDV+NVP | 14.16 (0.22 to 1694.00) | 0.14 to 2293.00 | - | - | - |
| ZDV+3TC+EFV vs 3TC+d4T+NVP | 0.24 (0.00 to 220.40) | 0.00 to 268.40 | - | - | - |
| ZDV+3TC+EFV vs ZDV+3TC+NVP | 0.35 (0.02 to 7.23) | 0.01 to 13.37 | - | - | - |
| ZDV+3TC+EFV vs ZDV+3TC+LOP+RIT | 1.24 (0.12 to 11.81) | 0.06 to 24.04 | 1.22 (0.35 to 4.39) | 1 (356) | RCT |
| *Common within-network between-study variance* | 0.77 (0.19 to 2.32) | - | 0.91 (0.23 to 2.70) | 26 (17507) | RCTs/Cohorts |
| *Design-by-treatment interaction model for inconsistency χ² (d.f., P-value, between-study variance)* | 6.62 (7, 0.47, 0.72) | |  |  | |
| *Model fit measures and diagnostics* Residual deviance= 68.74 Data points= 66 Effective number of parameters= 58.22 DIC= 126.96 | | | | | |
| ***Outcome: Low Birth Weight - # 30 studies (19 Cohorts, 1 case-control, 10 RCTs; 23 2-arm, 4 3-arm, 3 4-arm), # 21848 patients, # 16 treatments*** | | | | | |
| ZDV vs NoT/PLC | 0.85 (0.60 to 1.18) | 0.27 to 2.66 | 0.86 (0.62 to 1.16) | 14 (16620) | RCT |
| TDF vs NoT/PLC | 0.75 (0.20 to 2.83) | 0.13 to 4.14 | 0.74 (0.34 to 1.66) | 1 (199) | Cohort |
| TDF vs ZDV | 0.87 (0.22 to 3.49) | 0.15 to 5.05 | - | - | - |
| ZDV+3TC+IND vs NoT/PLC | 0.26 (0.00 to 7.06) | 0.00 to 8.76 | - | - | - |
| ZDV+3TC+IND vs ZDV | 0.30 (0.00 to 8.18) | 0.00 to 10.32 | - | - | - |
| ZDV+3TC+IND vs TDF | 0.34 (0.00 to 12.65) | 0.00 to 14.55 | - | - | - |
| ZDV+3TC vs NoT/PLC | 0.89 (0.35 to 1.96) | 0.21 to 3.25 | 0.55 (0.06 to 3.73) | 1 (28) | Cohort |
| ZDV+3TC vs ZDV | 1.04 (0.44 to 2.23) | 0.26 to 3.82 | 1.52 (0.71 to 3.24) | 3 (872) | Cohorts |
| ZDV+3TC vs TDF | 1.21 (0.23 to 5.36) | 0.17 to 7.49 | - | - | - |
| ZDV+3TC vs ZDV+3TC+IND | 3.39 (0.13 to 2017.00) | 0.10 to 2304.00 | 0.23 (0.00 to 122.60) | 1 (17) | Cohort |
| 3TC+d4T+IND vs NoT/PLC | 0.25 (0.00 to 7.13) | 0.00 to 8.71 | - | - | - |
| 3TC+d4T+IND vs ZDV | 0.29 (0.00 to 8.24) | 0.00 to 9.77 | - | - | - |
| 3TC+d4T+IND vs TDF | 0.32 (0.00 to 12.46) | 0.00 to 14.90 | - | - | - |
| 3TC+d4T+IND vs ZDV+3TC+IND | 0.97 (0.00 to 866.30) | 0.00 to 906.00 | 1.01 (0.00 to 817.20) | 1 (6) | Cohort |
| 3TC+d4T+IND vs ZDV+3TC | 0.29 (0.00 to 8.07) | 0.00 to 9.72 | 4.96 (0.01 to 5164.00) | 1 (17) | Cohort |
| ZDV+3TC+ABC vs NoT/PLC | 1.22 (0.32 to 4.27) | 0.22 to 6.33 | - | - | - |
| ZDV+3TC+ABC vs ZDV | 1.43 (0.40 to 4.96) | 0.27 to 7.62 | - | - | - |
| ZDV+3TC+ABC vs TDF | 1.66 (0.25 to 10.18) | 0.18 to 13.35 | - | - | - |
| ZDV+3TC+ABC vs ZDV+3TC+IND | 4.74 (0.14 to 3078.00) | 0.12 to 3491.00 | - | - | - |
| ZDV+3TC+ABC vs ZDV+3TC | 1.36 (0.32 to 6.17) | 0.23 to 9.05 | - | - | - |
| ZDV+3TC+ABC vs 3TC+d4T+IND | 4.99 (0.14 to 1609.00) | 0.12 to 1798.00 | - | - | - |
| 3TC+d4T+EFV vs NoT/PLC | **6.12 (1.04 to 39.13)** | **0.78 to 49.77** | - | - | - |
| 3TC+d4T+EFV vs ZDV | **7.15 (1.21 to 45.84)** | **0.90 to 63.98** | - | - | - |
| 3TC+d4T+EFV vs TDF | 8.30 (0.93 to 81.50) | 0.70 to 106.20 | - | - | - |
| 3TC+d4T+EFV vs ZDV+3TC+IND | 25.40 (0.54 to 18020.00) | 0.47 to 19160.00 | - | - | - |
| 3TC+d4T+EFV vs ZDV+3TC | **6.89 (1.03 to 54.82)** | **0.80 to 73.23** | - | - | - |
| 3TC+d4T+EFV vs 3TC+d4T+IND | 26.49 (0.56 to 9087.00) | 0.50 to 9824.00 | - | - | - |
| 3TC+d4T+EFV vs ZDV+3TC+ABC | 5.09 (0.74 to 38.84) | 0.54 to 51.51 | - | - | - |
| ZDV+3TC+NLF vs NoT/PLC | 1.47 (0.52 to 4.39) | 0.34 to 6.91 | 0.72 (0.20 to 2.60) | 2 (151) | Cohorts |
| ZDV+3TC+NLF vs ZDV | 1.72 (0.62 to 5.15) | 0.39 to 8.31 | 0.28 (0.00 to 7.43) | 1 (13) | Cohort |
| ZDV+3TC+NLF vs TDF | 1.97 (0.37 to 11.43) | 0.27 to 15.52 | - | - | - |
| ZDV+3TC+NLF vs ZDV+3TC+IND | 5.60 (0.21 to 3413.00) | 0.18 to 3947.00 | 31.37 (1.15 to 15580.00) | 1 (9) | Cohort |
| ZDV+3TC+NLF vs ZDV+3TC | 1.65 (0.51 to 6.11) | 0.36 to 9.61 | 139.00 (6.05 to 68650.00) | 1 (20) | Cohort |
| ZDV+3TC+NLF vs 3TC+d4T+IND | 5.94 (0.21 to 1809.00) | 0.17 to 2054.00 | 28.66 (1.02 to 12600.00) | 1 (9) | Cohort |
| ZDV+3TC+NLF vs ZDV+3TC+ABC | 1.20 (0.29 to 5.64) | 0.21 to 8.29 | - | - | - |
| ZDV+3TC+NLF vs 3TC+d4T+EFV | 0.24 (0.03 to 1.74) | 0.02 to 2.30 | - | - | - |
| NVP vs NoT/PLC | 1.39 (0.56 to 3.40) | 0.34 to 5.69 | - | - | - |
| NVP vs ZDV | 1.63 (0.72 to 3.79) | 0.42 to 6.40 | 1.61 (0.75 to 3.47) | 3 (1282) | RCTs |
| NVP vs TDF | 1.89 (0.37 to 9.05) | 0.26 to 12.88 | - | - | - |
| NVP vs ZDV+3TC+IND | 5.47 (0.17 to 3524.00) | 0.15 to 3898.00 | - | - | - |
| NVP vs ZDV+3TC | 1.57 (0.52 to 5.22) | 0.34 to 8.23 | - | - | - |
| NVP vs 3TC+d4T+IND | 5.63 (0.18 to 2005.00) | 0.15 to 2035.00 | - | - | - |
| NVP vs ZDV+3TC+ABC | 1.16 (0.26 to 5.34) | 0.18 to 7.69 | - | - | - |
| NVP vs 3TC+d4T+EFV | 0.23 (0.03 to 1.63) | 0.02 to 2.17 | - | - | - |
| NVP vs ZDV+3TC+NLF | 0.95 (0.24 to 3.53) | 0.16 to 5.18 | - | - | - |
| ZDV+NVP vs NoT/PLC | 0.46 (0.06 to 3.16) | 0.05 to 4.18 | 0.47 (0.08 to 2.31) | 1 (357) | Cohort |
| ZDV+NVP vs ZDV | 0.54 (0.07 to 3.83) | 0.06 to 5.04 | - | - | - |
| ZDV+NVP vs TDF | 0.62 (0.06 to 6.33) | 0.05 to 7.79 | - | - | - |
| ZDV+NVP vs ZDV+3TC+IND | 1.90 (0.04 to 1194.00) | 0.03 to 1417.00 | - | - | - |
| ZDV+NVP vs ZDV+3TC | 0.52 (0.06 to 4.45) | 0.05 to 5.72 | - | - | - |
| ZDV+NVP vs 3TC+d4T+IND | 1.93 (0.04 to 765.10) | 0.03 to 851.30 | - | - | - |
| ZDV+NVP vs ZDV+3TC+ABC | 0.38 (0.04 to 3.93) | 0.03 to 4.84 | - | - | - |
| ZDV+NVP vs 3TC+d4T+EFV | 0.08 (0.00 to 1.03) | 0.00 to 1.19 | - | - | - |
| ZDV+NVP vs ZDV+3TC+NLF | 0.31 (0.03 to 2.78) | 0.02 to 3.43 | - | - | - |
| ZDV+NVP vs NVP | 0.33 (0.04 to 2.72) | 0.03 to 3.76 | - | - | - |
| 3TC+d4T+NVP vs NoT/PLC | 2.89 (0.76 to 11.54) | 0.54 to 16.78 | **23.70 (2.65 to 802.60)** | 1 (69) | Cohort |
| 3TC+d4T+NVP vs ZDV | 3.39 (0.90 to 13.99) | 0.61 to 20.90 | - | - | - |
| 3TC+d4T+NVP vs TDF | 3.91 (0.61 to 26.23) | 0.47 to 35.35 | - | - | - |
| 3TC+d4T+NVP vs ZDV+3TC+IND | 11.55 (0.31 to 7755.00) | 0.26 to 8313.00 | - | - | - |
| 3TC+d4T+NVP vs ZDV+3TC | 3.26 (0.71 to 17.19) | 0.51 to 24.43 | - | - | - |
| 3TC+d4T+NVP vs 3TC+d4T+IND | 12.21 (0.32 to 3769.00) | 0.28 to 4267.00 | - | - | - |
| 3TC+d4T+NVP vs ZDV+3TC+ABC | 2.38 (0.51 to 12.55) | 0.36 to 18.20 | - | - | - |
| 3TC+d4T+NVP vs 3TC+d4T+EFV | 0.47 (0.14 to 1.57) | 0.09 to 2.40 | **0.47 (0.29 to 0.77)** | 1 (376) | Cohort |
| 3TC+d4T+NVP vs ZDV+3TC+NLF | 1.98 (0.39 to 9.91) | 0.28 to 14.08 | - | - | - |
| 3TC+d4T+NVP vs NVP | 2.09 (0.43 to 10.73) | 0.32 to 15.47 | - | - | - |
| 3TC+d4T+NVP vs ZDV+NVP | 6.27 (0.60 to 72.61) | 0.49 to 92.42 | - | - | - |
| ZDV+3TC+NVP vs NoT/PLC | 1.37 (0.46 to 3.70) | 0.28 to 5.77 | 0.74 (0.05 to 7.74) | 2 (39) | Cohorts |
| ZDV+3TC+NVP vs ZDV | 1.61 (0.55 to 4.33) | 0.34 to 6.91 | 0.42 (0.04 to 2.72) | 3 (102) | RCTs |
| ZDV+3TC+NVP vs TDF | 1.85 (0.32 to 9.68) | 0.24 to 13.60 | - | - | - |
| ZDV+3TC+NVP vs ZDV+3TC+IND | 5.29 (0.17 to 3159.00) | 0.14 to 3713.00 | - | - | - |
| ZDV+3TC+NVP vs ZDV+3TC | 1.54 (0.44 to 5.51) | 0.29 to 8.33 | 2.21 (0.04 to 140.30) | 1 (8) | Cohort |
| ZDV+3TC+NVP vs 3TC+d4T+IND | 5.54 (0.17 to 1731.00) | 0.14 to 1866.00 | - | - | - |
| ZDV+3TC+NVP vs ZDV+3TC+ABC | 1.13 (0.35 to 3.61) | 0.23 to 5.31 | 1.15 (0.64 to 2.01) | 1 (439) | RCT |
| ZDV+3TC+NVP vs 3TC+d4T+EFV | 0.22 (0.04 to 1.11) | 0.03 to 1.47 | - | - | - |
| ZDV+3TC+NVP vs ZDV+3TC+NLF | 0.93 (0.24 to 3.32) | 0.16 to 4.75 | 1.01 (0.00 to 941.00) | 1 (6) | Cohort |
| ZDV+3TC+NVP vs NVP | 0.99 (0.26 to 3.58) | 0.17 to 5.20 | - | - | - |
| ZDV+3TC+NVP vs ZDV+NVP | 2.97 (0.32 to 27.81) | 0.25 to 35.30 | - | - | - |
| ZDV+3TC+NVP vs 3TC+d4T+NVP | 0.47 (0.13 to 1.43) | 0.09 to 2.21 | 0.83 (0.39 to 1.71) | 1 (276) | Cohort |
| ZDV+3TC+LOP+RIT vs NoT/PLC | 1.64 (0.71 to 3.72) | 0.42 to 6.27 | 1.61 (0.64 to 4.48) | 1 (126) | Cohort |
| ZDV+3TC+LOP+RIT vs ZDV | 1.93 (0.87 to 4.27) | 0.51 to 7.38 | 1.55 (0.96 to 2.53) | 1 (763) | Cohort |
| ZDV+3TC+LOP+RIT vs TDF | 2.22 (0.46 to 10.27) | 0.33 to 14.70 | - | - | - |
| ZDV+3TC+LOP+RIT vs ZDV+3TC+IND | 6.29 (0.23 to 3982.00) | 0.20 to 4061.00 | - | - | - |
| ZDV+3TC+LOP+RIT vs ZDV+3TC | 1.86 (0.64 to 5.80) | 0.41 to 9.10 | - | - | - |
| ZDV+3TC+LOP+RIT vs 3TC+d4T+IND | 6.59 (0.23 to 2062.00) | 0.20 to 2415.00 | - | - | - |
| ZDV+3TC+LOP+RIT vs ZDV+3TC+ABC | 1.36 (0.45 to 4.28) | 0.29 to 6.63 | 1.33 (0.83 to 2.14) | 1 (553) | RCT |
| ZDV+3TC+LOP+RIT vs 3TC+d4T+EFV | 0.27 (0.04 to 1.61) | 0.03 to 2.02 | - | - | - |
| ZDV+3TC+LOP+RIT vs ZDV+3TC+NLF | 1.13 (0.38 to 3.05) | 0.24 to 4.80 | 1.75 (0.56 to 5.37) | 2 (188) | Case-Control/Cohort |
| ZDV+3TC+LOP+RIT vs NVP | 1.18 (0.37 to 3.75) | 0.25 to 5.53 | - | - | - |
| ZDV+3TC+LOP+RIT vs ZDV+NVP | 3.55 (0.44 to 30.96) | 0.33 to 40.28 | - | - | - |
| ZDV+3TC+LOP+RIT vs 3TC+d4T+NVP | 0.57 (0.14 to 2.20) | 0.09 to 3.19 | - | - | - |
| ZDV+3TC+LOP+RIT vs ZDV+3TC+NVP | 1.20 (0.47 to 3.29) | 0.30 to 5.30 | **1.17 (0.69 to 2.04)** | 1 (426) | Cohort |
| 3TC+d4T+LOP+RIT vs NoT/PLC | 2.27 (0.39 to 13.62) | 0.29 to 18.59 | - | - | - |
| 3TC+d4T+LOP+RIT vs ZDV | 2.65 (0.45 to 16.65) | 0.34 to 22.73 | - | - | - |
| 3TC+d4T+LOP+RIT vs TDF | 3.02 (0.35 to 28.64) | 0.28 to 36.82 | - | - | - |
| 3TC+d4T+LOP+RIT vs ZDV+3TC+IND | 9.29 (0.20 to 6150.00) | 0.17 to 6690.00 | - | - | - |
| 3TC+d4T+LOP+RIT vs ZDV+3TC | 2.56 (0.39 to 19.68) | 0.29 to 26.70 | - | - | - |
| 3TC+d4T+LOP+RIT vs 3TC+d4T+IND | 9.68 (0.22 to 3169.00) | 0.17 to 3457.00 | - | - | - |
| 3TC+d4T+LOP+RIT vs ZDV+3TC+ABC | 1.86 (0.28 to 13.98) | 0.21 to 18.91 | - | - | - |
| 3TC+d4T+LOP+RIT vs 3TC+d4T+EFV | 0.37 (0.11 to 1.18) | 0.07 to 1.80 | **0.37 (0.23 to 0.59)** | 1 (534) | Cohort |
| 3TC+d4T+LOP+RIT vs ZDV+3TC+NLF | 1.55 (0.21 to 11.05) | 0.17 to 14.27 | - | - | - |
| 3TC+d4T+LOP+RIT vs NVP | 1.62 (0.23 to 12.24) | 0.18 to 16.30 | - | - | - |
| 3TC+d4T+LOP+RIT vs ZDV+NVP | 4.89 (0.38 to 75.96) | 0.31 to 90.25 | - | - | - |
| 3TC+d4T+LOP+RIT vs 3TC+d4T+NVP | 0.78 (0.25 to 2.47) | 0.16 to 3.78 | 0.78 (0.53 to 1.16) | 1 (680) | Cohort |
| 3TC+d4T+LOP+RIT vs ZDV+3TC+NVP | 1.65 (0.34 to 9.37) | 0.24 to 13.19 | - | - | - |
| 3TC+d4T+LOP+RIT vs ZDV+3TC+LOP+RIT | 1.37 (0.24 to 8.69) | 0.18 to 11.58 | - | - | - |
| ZDV+3TC+LOP+RIT+TDF vs NoT/PLC | 0.16 (0.00 to 4.59) | 0.00 to 5.53 | - | - | - |
| ZDV+3TC+LOP+RIT+TDF vs ZDV | 0.18 (0.00 to 5.30) | 0.00 to 6.35 | **0.01 (0.00 to 0.80)** | 1 (4) | Cohort |
| ZDV+3TC+LOP+RIT+TDF vs TDF | 0.21 (0.00 to 8.18) | 0.00 to 9.25 | - | - | - |
| ZDV+3TC+LOP+RIT+TDF vs ZDV+3TC+IND | 0.63 (0.00 to 751.80) | 0.00 to 788.30 | - | - | - |
| ZDV+3TC+LOP+RIT+TDF vs ZDV+3TC | 0.18 (0.00 to 5.69) | 0.00 to 6.87 | - | - | - |
| ZDV+3TC+LOP+RIT+TDF vs 3TC+d4T+IND | 0.64 (0.00 to 435.40) | 0.00 to 469.30 | - | - | - |
| ZDV+3TC+LOP+RIT+TDF vs ZDV+3TC+ABC | 0.13 (0.00 to 4.03) | 0.00 to 4.88 | - | - | - |
| ZDV+3TC+LOP+RIT+TDF vs 3TC+d4T+EFV | 0.02 (0.00 to 1.02) | 0.00 to 1.13 | - | - | - |
| ZDV+3TC+LOP+RIT+TDF vs ZDV+3TC+NLF | 0.10 (0.00 to 3.49) | 0.00 to 4.13 | - | - | - |
| ZDV+3TC+LOP+RIT+TDF vs NVP | 0.11 (0.00 to 3.75) | 0.00 to 4.49 | - | - | - |
| ZDV+3TC+LOP+RIT+TDF vs ZDV+NVP | 0.32 (0.00 to 16.86) | 0.00 to 19.20 | - | - | - |
| ZDV+3TC+LOP+RIT+TDF vs 3TC+d4T+NVP | 0.05 (0.00 to 1.79) | 0.00 to 2.12 | - | - | - |
| ZDV+3TC+LOP+RIT+TDF vs ZDV+3TC+NVP | 0.12 (0.00 to 3.20) | 0.00 to 3.73 | 0.21 (0.00 to 4.99) | 1 (17) | Cohort |
| ZDV+3TC+LOP+RIT+TDF vs ZDV+3TC+LOP+RIT | 0.10 (0.00 to 2.81) | 0.00 to 3.38 | - | - | - |
| ZDV+3TC+LOP+RIT+TDF vs 3TC+d4T+LOP+RIT | 0.07 (0.00 to 2.77) | 0.00 to 3.12 | - | - | - |
| *Common within-network between-study variance* | 0.25 (0.08 to 0.75) | - | 0.19 (0.06 to 0.55) | 30 (21848) | RCTs/Cohorts/Case-Control |
| *Design-by-treatment interaction model for inconsistency χ² (d.f., P-value, between-study variance)* | 16.61 (11, 0.12, 0.15) | |  |  | |
| *Model fit measures and diagnostics*  Residual deviance= 74.35 Data points= 70 Effective number of parameters= 58.87 DIC= 133.22 | | | | | |
| ***Outcome: Short-Length - # 1 study (1 RCT; 1 2-arm), # 1088 patients, # 2 treatments*** | | | | | |
| ZDV vs NoT (ART-mon vs NoT) | N/A | - | 166.67 (111.11 to 250.00) | 1 (1088) | RCT |
| **Notes:** Statistically significant results are **bolded**. **^†^** A fixed-effect model was used due to insufficient number of studies per design.  **Abbreviations:** ABC, Abacavir; CrI, Credible Interval; d.f., Degrees of freedom; ddI, Didanosine; IND; DIC, Deviance Information Criterion; Indinavir; 3TC, Lamivudine; LOP, Lopinavir; NVP, Nevirapine; NLF Nelfinavir; No., Number; NoT, No Treatment; N/A, Not Applicable; Plc, Placebo; SAQ, Saquinavir; d4T Stavudine; EFV, Sustiva; RIT, Ritonavir; ZDV, Zidovudine. | | | | | |
