# Supplementary material for: Comparative safety and effectiveness of perinatal antiretroviral therapies for HIV-infected women and their children: Systematic review and network meta-analysis including different study designs
Source: PLoS One. 2018 Jun 18;13(6):e0198447. doi: 10.1371/journal.pone.0198447 (PMC6005568; doi:10.1371/journal.pone.0198447)
Supplement: S17 Appendix — (DOCX) [file pone.0198447.s017.docx]

# S17 Appendix. Network Meta-analysis and Meta-analysis results for Antiretroviral Drug Categories by Outcomes

| ***Treatment Comparison*** | ***Network Meta-Analysis Odds Ratio Estimate (CrI)*** | ***Predictive interval*** | ***Pairwise Meta-Analysis Odds Ratio Estimate (CrI)*** | ***No. of Studies (No. of Patients)*** | | ***Study design*** |
| --- | --- | --- | --- | --- | --- | --- |
| ***Outcome: Total Congenital Malformations - # 17 studies (9 Cohorts + 8 RCTs, # 7833 patients, # 4 treatments)*** | | | | | | |
| ART-mono vs Not/PLC | 1.06 (0.63 to 2.00) | 0.26 to 5.10 | 1.06 (0.58 to 2.11) | 11 (2228) | | RCTs/Cohort |
| ART-dual vs Not/PLC | 1.27 (0.62 to 2.74) | 0.28 to 6.03 | 1.13 (0.41 to 3.00) | 4 (1656) | | RCTs/Cohort |
| ART-any vs Not/PLC | N/A | - | 0.92 (0.42 to 1.68) | 8 (9297) | | Cohort |
| HAART vs Not/PLC | 1.23 (0.61 to 2.71) | 0.29 to 6.14 | 0.97 (0.34 to 2.99) | 4 (1357) | | Cohort |
| ART-dual vs ART-mono | 1.21 (0.57 to 2.35) | 0.25 to 5.30 | 1.69 (0.64 to 4.02) | 5 (1973) | | RCTs/Cohort |
| ART-any vs ART-mono | N/A | - | **0.00 (0.00 to 0.00)** | 2 (35510) | | Cohort |
| HAART vs ART-mono | 1.16 (0.59 to 2.23) | 0.25 to 5.04 | 1.11 (0.48 to 2.37) | 7 (2422) | | RCTs/Cohort |
| HAART vs ART-dual | 0.97 (0.45 to 2.11) | 0.21 to 4.64 | 1.46 (0.55 to 4.04) | 3 (1381) | | Cohort |
| *Common between-study variance across treatment comparisons* | 0.33 (0.01 to 1.35) | - | 0.36 (0.00 to 1.32) | 17 (7833) | | RCTs/Cohorts |
| *Design-by-treatment interaction model for inconsistency χ² (d.f., P-value, between-study variance)* | 15.69 (7, 0.03, 0.00) | |  |  | | |
| *Model fit measures and diagnostics*  Residual deviance= 48.85 Data points= 41 Effective number of parameters= 32.9 DIC= 81.76 | | | | | | |
| ***Outcome: Major Congenital Malformations - # 9 studies (6 Cohorts + 3 RCTs ; (6 2-arm, 1 3-arm, 2 4-arm) , # 3475 patients, # 4 treatments*** | | | | | | |
| ART-mono vs Not/PLC | 0.79 (0.45 to 1.32) | 0.29 to 2.10 | 0.80 (0.46 to 1.33) | 8 (1481) | | RCTs/Cohorts |
| ART-dual vs Not/PLC | 1.51 (0.60 to 3.25) | 0.42 to 4.41 | 1.44 (0.40 to 4.84) | 3 (155) | | Cohorts |
| ART-any vs Not/PLC | N/A | - | 1.14 (0.70 to 2.03) | 3 (5366) | | Cohorts |
| HAART vs Not/PLC | 0.82 (0.39 to 1.67) | 0.26 to 2.35 | 0.70 (0.32 to 1.70) | 4 (1357) | | Cohorts |
| ART-dual vs ART-mono | 1.91 (0.87 to 3.82) | 0.58 to 5.47 | 1.82 (0.82 to 3.79) | 4 (1601) | | Cohorts |
| ART-any vs ART-mono | N/A | - | **0.00 (0.00 to 0.01)** | 1 (239) | | Cohorts |
| HAART vs ART-mono | 1.04 (0.53 to 2.12) | 0.34 to 3.03 | 1.25 (0.52 to 3.00) | 4 (1439) | | Cohorts |
| HAART vs ART-dual | 0.55 (0.24 to 1.34) | 0.18 to 1.83 | 0.58 (0.22 to 1.80) | 3 (1276) | | Cohorts |
| *Common between-study variance across treatment comparisons* | 0.06 (0.00 to 0.87) | - | 0.05 (0.00 to 0.63) | 12 (8104) | | RCTs/Cohorts |
| *Design-by-treatment interaction model for inconsistency χ² (d.f., P-value, between-study variance)* | 2.04 (4, 0.73, 0.00) | |  | | | |
| *Model fit measures and diagnostics* Residual deviance = 26.21 Data points = 25 Effective number of parameters = 17.00 DIC = 43.21 | | | | | | |
| ***Outcome: Minor Congenital Malformations - # 2 studies (Cohorts) # 87 patients, # 4 treatments*** | | | | | | |
| ART-mono vs NoT/PLC | 5.00 (0.37 to 226.00) | 0.19 to 407.00 | 6.17 (0.42 to 268.90) | 2 (55) | | Cohort |
| ART-dual vs NoT/PLC | 0.58 (0.00 to 67.07) | 0.00 to 103.20 | 0.74 (0.00 to 632.70) | 1 (14) | | Cohort |
| ART-any vs NoT/PLC | N/A | - | 0.32 (0.02 to 5.18) | 1 (2527) | | Cohort |
| HAART vs NoT/PLC | 9.66 (0.59 to 390.70) | 0.28 to 694.70 | 7.53 (0.45 to 319.90) | 2 (42) | | Cohort |
| ART-dual vs ART-mono | 0.11 (0.00 to 3.77) | 0.00 to 6.91 | 0.20 (0.00 to 3.95) | 1 (35) | | Cohort |
| HAART vs ART-mono | 2.01 (0.18 to 13.29) | 0.06 to 32.10 | 2.04 (0.19 to 12.76) | 2 (61) | | Cohort |
| HAART vs ART-dual | 16.81 (0.47 to 9645.00) | 0.23 to 12130.00 | **23.37 (1.47 to 13690.00)** | 1 (26) | | Cohort |
| *Common between-study variance across treatment comparisons* | 0.60 (0.00 to 5.01) | - | 0.57 (0.00 to 4.70) | 2 (87) | | Cohorts |
| *Design-by-treatment interaction model for inconsistency χ² (d.f., P-value, between-study variance)* | N/A – all closed loops are correlated | |  |  | | |
| *Model fit measures and diagnostics* Residual deviance = 7.61 Data points = 7 Effective number of parameters = 6.16 DIC = 13.77 | | | | | | |
| ***Outcome: MTCT - #12 studies (9 Cohorts +34 RCTs) , # 14967 patients, # 6 treatments*** | | | | | | |
| [NoT]+[ART-any] vs [NoT/PLC]+[NoT/PLC] | 0.37 (0.14 to 1.05) | 0.05 to 2.94 | 0.64 (0.07 to 22.00) | 1 (60) | | Cohort |
| [NoT]+[ART-mono] vs [NoT/PLC]+[NoT/PLC] | N/A | - | 0.43 (0.12 to 1.58) | 2 (5453) | | Cohort |
| [ART-mono]+[NoT] vs [NoT/PLC]+[NoT/PLC] | 0.37 (0.12 to 1.06) | 0.04 to 2.95 | 0.33 (0.09 to 1.19) | 2 (3395) | | Cohort |
| [ART-mono]+[ART-mono] vs [NoT/PLC]+[NoT/PLC] | **0.28 (0.13 to 0.53)** | 0.04 to 1.82 | **0.31 (0.14 to 0.60)** | 8 (4993) | | RCT/Cohort |
| [ART-dual]+[NoT] vs [NoT/PLC]+[NoT/PLC] | **0.04 (0.00 to 0.33)** | 0.00 to 0.64 | **0.04 (0.01 to 0.13)** | 1 (2722) | | Cohort |
| [HAART]+[NoT] vs [NoT/PLC]+[NoT/PLC] | **0.12 (0.04 to 0.30)** | 0.02 to 0.85 | **0.11 (0.04 to 0.27)** | 4 (9278) | | Cohort |
| [ART-any]+[ART-mono] vs [NoT/PLC]+[NoT/PLC] | N/A | - | **0.07 (0.01 to 0.28)** | 1 (96) | | Cohort |
| [ART-any]+[NoT/PLC] vs [NoT/PLC]+[NoT/PLC] | N/A | - | 0.04 (0.00 to 1.57) | 1 (451) | | Cohort |
| [ART-mono]+[NoT] vs [NoT]+[ART-mono] | 1.01 (0.25 to 3.48) | 0.10 to 8.49 | 0.84 (0.22 to 4.32) | 1 (102) | | Cohort |
| [ART-mono]+[ART-mono] vs [NoT]+[ART-mono] | 0.76 (0.25 to 2.03) | 0.08 to 5.41 | 0.54 (0.15 to 1.68) | 3 (1758) | | Cohort |
| [HAART]+[NoT] vs [NoT]+[ART-mono] | **0.34 (0.10 to 0.90)** | 0.04 to 2.36 | 0.66 (0.20 to 2.22) | 2 (4796) | | Cohort |
| [ART-any]+[ART-mono] vs [NoT]+[ART-mono] | N/A | - | **0.04 (0.01 to 0.36)** | 1 (649) | | Cohort |
| [ART-mono]+[ART-mono] vs [ART-mono]+[NoT] | 0.76 (0.24 to 2.34) | 0.09 to 6.31 | 0.87 (0.17 to 4.11) | 2 (243) | | Cohort |
| [ART-dual]+[NoT] vs [ART-mono]+[NoT] | **0.12 (0.01 to 0.98)** | 0.01 to 1.82 | **0.11 (0.02 to 0.38)** | 1 (1022) | | Cohort |
| [HAART]+[NoT] vs [ART-mono]+[NoT] | N/A | - | **0.18 (0.09 to 0.33)** | 1 (1990) | | Cohort |
| [HAART]+[NoT] vs [ART-mono]+[ART-mono] | 0.44 (0.16 to 1.18) | 0.06 to 3.33 | 0.46 (0.09 to 1.71) | 2 (1854) | | Cohort |
| [ART-any]+[ART-mono] vs [ART-mono]+[ART-mono] | N/A | - | 0.86 (0.12 to 23.56) | 1 (707) | | Cohort |
| [ART-any]+[ART-mono] vs [NoT]+[ART-dual] | N/A | - | 16.63 (0.72 to 10450.00) | 1 (18) | | Cohort |
| [HAART]+[NoT] vs [ART-dual]+[NoT] | 2.71 (0.33 to 33.72) | 0.17 to 57.43 | 1.73 (0.45 to 11.70) | 1 (1534) | | Cohort |
| [ART-any]+[NoT/PLC] vs [ART-any]+[ART-mono] | N/A | - | 1.15 (0.46 to 2.88) | 1 (1248) | | RCT |
| [NoT]+[ART-any] vs [ART-any]+[NoT/PLC] | N/A | - | **15.84 (3.58 to 85.47)** | 1 (499) | | Cohort |
| [ART-dual]+[NoT] vs [NoT]+[ART-mono] | 0.12 (0.01 to 1.04) | 0.00 to 1.91 | N/A | - | | - |
| [ART-dual]+[NoT] vs [ART-mono]+[ART-mono] | 0.16 (0.01 to 1.26) | 0.01 to 2.43 | N/A | - | | - |
| [HAART]+[NoT] vs [ART-mono]+[NoT] | 0.33 (0.09 to 1.11) | 0.04 to 2.85 | N/A | - | | - |
| *Common between-study variance across treatment comparisons* | 0.66 (0.20 to 2.05) | - | 0.59 (0.18 to 1.70) | 12 (14967) | | RCT/Cohorts |
| *Design-by-treatment interaction model for inconsistency χ² (d.f., P-value, between-study variance)* | 27.22 (11, 0.00, 0.06) | |  | | | |
| *Model fit measures and diagnostics*  Residual deviance= 35.32 Data points= 32 Effective number of parameters= 29.52 DIC= 64.84 | | | | | | |
| ***Outcome: Infant and child Deaths - # 15 studies (8 Cohorts + 7 RCTs; 15 2-arm, 1 3-arm), # 11451 patients, # 4 treatments*** | | | | | | |
| ART-mono vs Not/PLC | 0.52 (0.27 to 1.13) | 0.08 to 3.78 | 0.46 (0.23 to 1.03) | 9 (7631) | | RCTs/Cohorts |
| ART-dual vs Not/PLC | 0.51 (0.16 to 1.89) | 0.06 to 4.93 | 0.33 (0.08 to 1.13) | 2 (3107) | | RCTs/Cohorts |
| ART-any vs Not/PLC | N/A | - | 0.26 (0.06 to 1.10) | 2 (1638) | | Cohorts |
| HAART vs Not/PLC | 0.36 (0.12 to 1.18) | 0.04 to 3.20 | 1.32 (0.18 to 13.32) | 2 (356) | | Cohorts |
| ART-dual vs ART-mono | 0.98 (0.27 to 3.62) | 0.11 to 8.91 | 0.49 (0.11 to 1.42) | 1 (5191) | | Cohorts |
| ART-any vs ART-mono | N/A | - | 0.76 (0.11 to 2.70) | 1 (4789) | | Cohorts |
| HAART vs ART-mono | 0.69 (0.22 to 2.01) | 0.08 to 5.67 | 0.62 (0.16 to 2.40) | 2 (1233) | | RCTs/Cohorts |
| ART-any vs ART-dual | N/A | - | 1.54 (0.18 to 10.48) | 1 (956) | | Cohorts |
| HAART vs ART-dual | 0.71 (0.14 to 3.10) | 0.06 to 6.88 | **0.00 (0.00 to 0.00)** | 1 (51) | | RCTs |
| HAART vs ART-any | N/A | - | 2.58 (0.68 to 18.91) | 1 (4396) | | Cohorts |
| *Common between-study variance across treatment comparisons* | 0.61 (0.13 to 2.43) | - | 0.61 (0.16 to 2.21) | 15 (11451) | | RCTs/Cohorts |
| *Design-by-treatment interaction model for inconsistency χ² (d.f., P-value, between-study variance)* | 2.41 (2, 0.30, 0.00) | |  | | | |
| *Model fit measures and diagnostics* Residual deviance= 36.66 Data points= 31 Effective number of parameters= 25.32 DIC= 61.98 | | | | | | |
| ***Outcome: Preterm Births - # 40 studies (35 Cohorts + 5 RCTs; 32 2-arm, 6 3-arm, 2 4-arm), # 36727 patients, # 4 treatments*** | | | | | | |
| ART-mono vs Not/PLC | **0.60 (0.41 to 0.89)** | 0.11 to 3.41 | **0.68 (0.47 to 0.99)** | 21 (22418) | | RCTs/Cohorts |
| ART-dual vs Not/PLC | **0.30 (0.15 to 0.60)** | 0.05 to 1.86 | **0.10 (0.03 to 0.29)** | 3 (3981) | | Cohorts |
| ART-any vs Not/PLC | N/A | - | 0.79 (0.46 to 1.34) | 10 (6383) | | Cohorts |
| HAART vs Not/PLC | 0.72 (0.44 to 1.15) | 0.13 to 4.08 | 0.62 (0.37 to 1.04) | 12 (14676) | | Cohorts |
| ART-dual vs ART-mono | **0.51 (0.25 to 0.97)** | 0.08 to 3.04 | 1.19 (0.55 to 2.53) | 5 (4408) | | Cohorts |
| ART-any vs ART-mono | N/A | - | 1.55 (0.83 to 2.94) | 6 (15955) | | RCTs/Cohorts |
| HAART vs ART-mono | 1.20 (0.75 to 1.89) | 0.20 to 7.00 | 1.35 (0.82 to 2.23) | 12 (17564) | | RCTs/Cohorts |
| ART-any vs ART-dual | N/A | - | 1.36 (0.36 to 5.61) | 2 (662) | | Cohorts |
| HAART vs ART-dual | **2.39 (1.27 to 4.66)** | 0.40 to 15.32 | **2.58 (1.38 to 4.94)** | 9 (5019) | | RCTs/Cohorts |
| HAART vs ART-any | N/A | - | 1.40 (0.88 to 2.22) | 14 (14129) | | Cohorts |
| *Common between-study variance across treatment comparisons* | 0.70 (0.39 to 1.27) | - | 0.53 (0.34 to 0.83) | 40 (36727) | | RCTs/Cohorts |
| *Design-by-treatment interaction model for inconsistency χ² (d.f., P-value, between-study variance)* | 45.56 (9, 0.00, 0.14 ) | |  |  | | |
| *Model fit measures and diagnostics* Residual deviance= 93.95 Data points= 90 Effective number of parameters= 79.11 DIC= 173.06 | | | | | | |
| ***Outcome: Stillbirths - # 33 studies (21 Cohorts + 12 RCTs; 36 2-arm, 4 3-arm, 1 4-arm), # 21542 patients, # 4 treatments*** | | | | | | |
| ART-mono vs Not/PLC | **0.43 (0.23 to 0.83)** | 0.05 to 3.56 | 0.56 (0.29 to 1.07) | 16 (11141) | | RCTs/Cohorts |
| ART-dual vs Not/PLC | 0.40 (0.14 to 1.05) | 0.04 to 3.66 | **0.16 (0.03 to 0.77)** | 3 (1875) | | RCTs/Cohorts |
| ART-any vs Not/PLC | N/A | - | **0.37 (0.16 to 0.87)** | 8 (8984) | | Cohorts |
| HAART vs Not/PLC | 0.59 (0.24 to 1.32) | 0.06 to 5.24 | 0.41 (0.14 to 1.11) | 5 (6165) | | Cohorts |
| ART-dual vs ART-mono | 0.94 (0.35 to 2.29) | 0.09 to 7.86 | 1.69 (0.51 to 5.41) | 5 (2600) | | RCTs/Cohorts |
| ART-any vs ART-mono | N/A | - | 3.01 (0.86 to 10.91) | 4 (16017) | | RCTs/Cohorts |
| HAART vs ART-mono | 1.36 (0.57 to 2.98) | 0.15 to 11.24 | 2.40 (0.84 to 6.63) | 4 (8551) | | RCTs/Cohorts |
| ART-any vs ART-dual | N/A | - | 1.33 (0.00 to 1111.00) | 1 (14) | | Cohorts |
| HAART vs ART-dual | 1.46 (0.55 to 3.99) | 0.16 to 13.93 | 1.54 (0.45 to 5.45) | 6 (877) | | RCTs/Cohorts |
| HAART vs ART-any | N/A | - | 1.68 (0.43 to 6.42) | 2 (5139) | | Cohorts |
| *Common between-study variance across treatment comparisons* | 0.92 (0.33 to 2.28) | - | 0.71 (0.29 to 1.56) | 33 (21545) | | RCTs/Cohorts |
| *Design-by-treatment interaction model for inconsistency χ² (d.f., P-value, between-study variance)* | 10.45 (9, 0.32, 0.46) | |  |  | | |
| *Model fit measures and diagnostics* Residual deviance= 74.69 Data points= 69 Effective number of parameters= 55.41 DIC= 130.10 | | | | | | |
| ***Outcome: Low Birth Weight - # 35 studies (28 Cohorts, 7 RCTs; 26 2-arm, 6 3-arm, 3 4-arm), # 31319 patients, # 4 treatments*** | | | | | | |
| ART-mono vs Not/PLC | 0.78 (0.58 to 1.03) | 0.27 to 2.18 | 0.84 (0.63 to 1.10) | 17 (22065) | | RCTs/Cohorts |
| ART-dual vs Not/PLC | 0.79 (0.51 to 1.19) | 0.27 to 2.33 | 0.58 (0.30 to 1.13) | 4 (4153) | | Cohorts |
| ART-any vs Not/PLC | N/A | - | 1.26 (0.83 to 1.97) | 8 (4703) | | Cohorts |
| HAART vs Not/PLC | 1.30 (0.92 to 1.87) | 0.46 to 3.71 | 1.29 (0.82 to 2.07) | 9 (6619) | | Cohorts |
| ART-dual vs ART-mono | 1.02 (0.69 to 1.47) | 0.35 to 2.85 | 1.46 (0.96 to 2.25) | 8 (5381) | | Cohorts |
| ART-any vs ART-mono | N/A | - | 1.04 (0.66 to 1.65) | 5 (15656) | | RCTs/Cohorts |
| HAART vs ART-mono | **1.67 (1.23 to 2.34)** | 0.59 to 4.90 | 1.39 (0.98 to 1.96) | 13 (8923) | | RCTs/Cohorts |
| ART-any vs ART-dual | N/A | - | 0.59 (0.34 to 1.03) | 1 (409) | | Cohorts |
| HAART vs ART-dual | **1.64 (1.13 to 2.48)** | 0.57 to 4.89 | **1.95 (1.29 to 3.03)** | 11 (5342) | | RCTs/Cohorts |
| HAART vs ART-any | N/A | - | 1.11 (0.68 to 1.87) | 6 (2491) | | Cohorts |
| *Common between-study variance across treatment comparisons* | 0.23 (0.10 to 0.50) | - | 0.20 (0.11 to 0.36) | 46 (49592) | | RCTs/Cohorts |
| *Design-by-treatment interaction model for inconsistency χ² (d.f., P-value, between-study variance)* | 51.19 (11, 0, 0.03) | |  |  | | |
| *Model fit measures and diagnostics* Residual deviance= 94.08 Data points= 82 Effective number of parameters= 67.94 DIC= 162.03 | | | | | | |
| ***Outcome: Short-Length - # 1 study (1 RCT; 1 2-arm), # 1088 patients, # 2 treatments*** | | | | | | |
| ZDV vs NoT (ART-mon vs NoT) | N/A | - | 166.67 (111.11 to 250.00) | | 1 (1088) | RCT |
| ***Outcome: Small Head - # 1 study (1 RCT; 1 2-arm), # 62 patients, # 2 treatments*** | | | | | | |
| ART-any vs HAART | N/A | - | 0.63 (0.06 to 6.33) | | 1 (62) | RCT |
| **Notes:** Statistically significant results are **bolded**. **^†^** A fixed-effect model was used due to insufficient number of studies per design.  **Abbreviations:** ABC, Abacavir; CrI, Credible Interval; d.f., Degrees of freedom; ddI, Didanosine; IND; DIC, Deviance Information Criterion; Indinavir; 3TC, Lamivudine; LOP, Lopinavir; NVP, Nevirapine; NLF Nelfinavir; No., Number; NoT, No Treatment; N/A, Not Applicable; Plc, Placebo; SAQ, Saquinavir; d4T Stavudine; EFV, Sustiva; RIT, Ritonavir; ZDV, Zidovudine. | | | | | | |
