# Supplementary material for: Comparative safety and effectiveness of perinatal antiretroviral therapies for HIV-infected women and their children: Systematic review and network meta-analysis including different study designs
Source: PLoS One. 2018 Jun 18;13(6):e0198447. doi: 10.1371/journal.pone.0198447 (PMC6005568; doi:10.1371/journal.pone.0198447)
Supplement: S18 Appendix — (DOCX) [file pone.0198447.s018.docx]

# S18 Appendix. Plots from Inconsistency Assessment using the Loop-Specific Approach


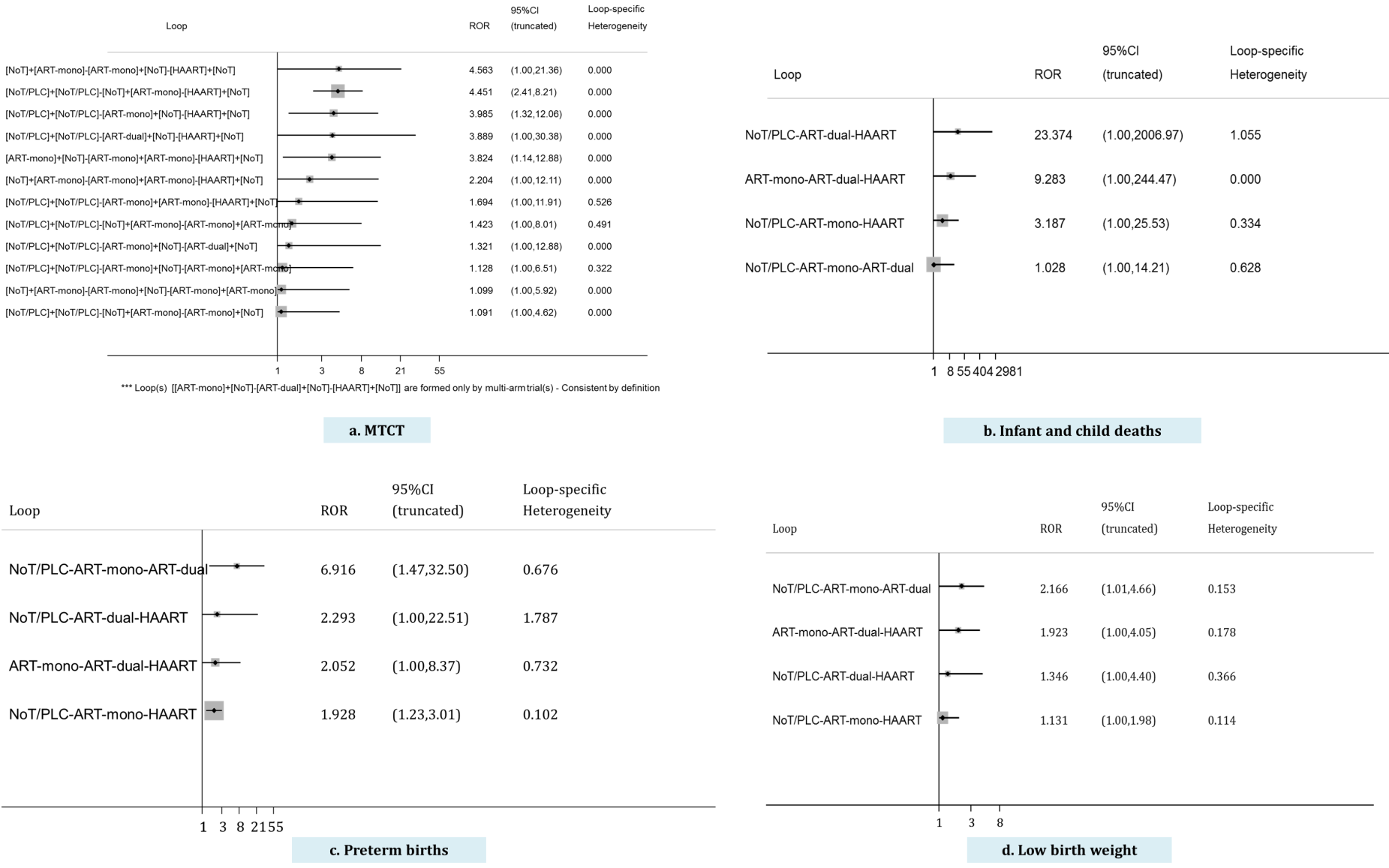


**Forest Plots from I****nconsistency Assessment using the Loop-Spe****cific Approach.**

**Legend: A) Mother-to-Child Transmission of HIV B) Infant and child deaths C) Preterm births D) Low birth weight.** The black horizontal lines represent the credible intervals for the summary odds ratios for each specific drug comparison and the red horizontal lines represent the predictive intervals. The blue vertical line is the line of no effect. The 95% CI is truncated at zero since the direction of the inconsistency factor is irrelevant to the inconsistency assessment. The loop-specific beween-study variance is presented in each plot, estimated with the restricted maximum likelihood method. **Abbreviations:** ART, Antiretroviral Therapy; CI, Confidence Interval; dual, duo- therapy; HAART, Highly Active Anti-Retroviral; mono, monotherapy; NoT, No Treatment; PLC, Placebo; ROR, Ratio of Odds Ratios.
