# Supplementary material for: Comparative safety and effectiveness of perinatal antiretroviral therapies for HIV-infected women and their children: Systematic review and network meta-analysis including different study designs
Source: PLoS One. 2018 Jun 18;13(6):e0198447. doi: 10.1371/journal.pone.0198447 (PMC6005568; doi:10.1371/journal.pone.0198447)
Supplement: S19 Appendix — (DOCX) [file pone.0198447.s019.docx]

# S19 Appendix. Forests Plots for Antiretroviral Therapy Drug Categories versus No treatment/placebo for each outcome


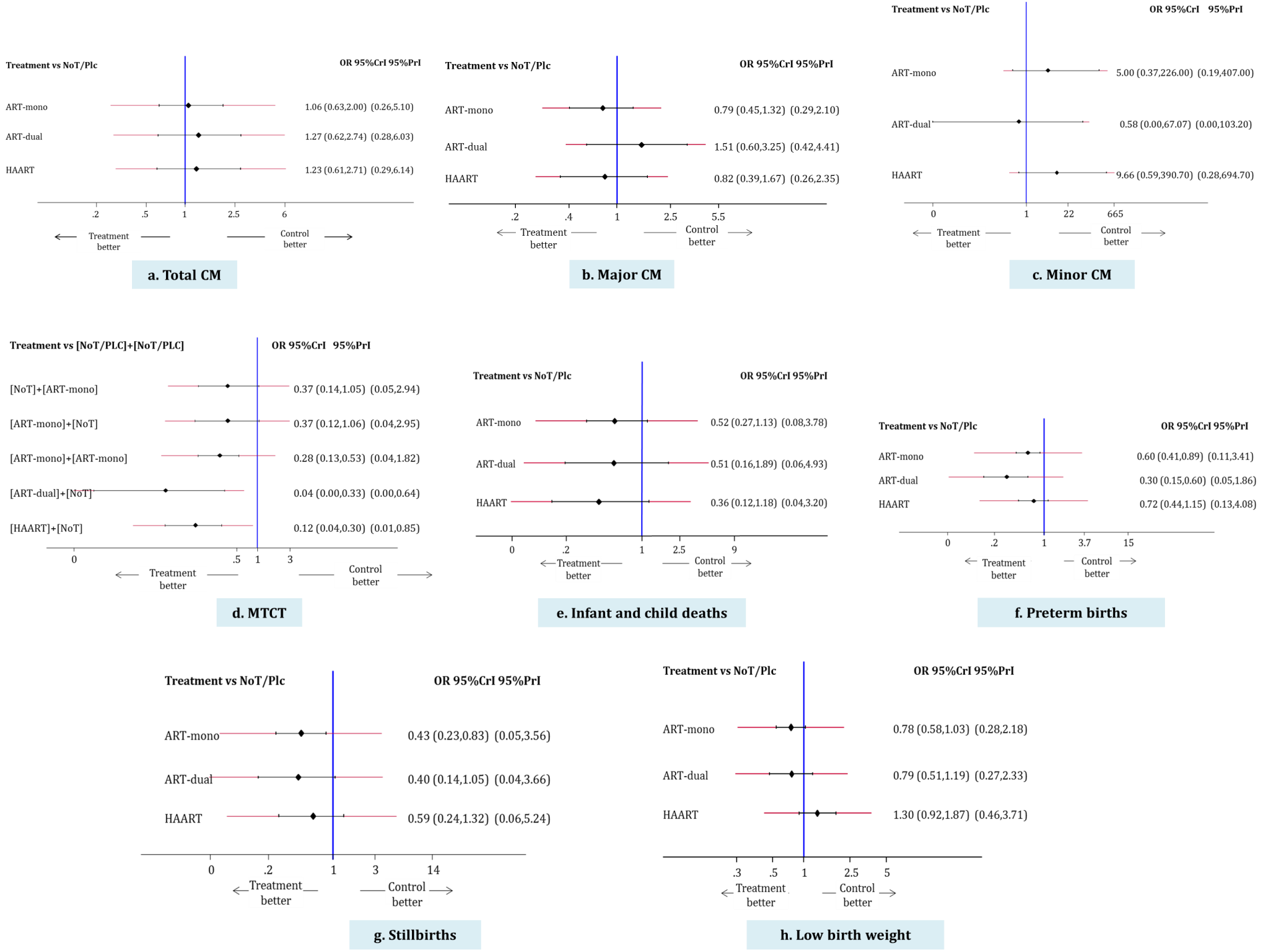


**Forests Plot for each outcome using Antiretroviral Therapy Drug Categories.**

**Legend:** (𝜏^2^= Between-Study Variance) **A) Total Congenital Malformation** (𝜏^2^= 0.33, 95% CrI 0.01-1.35) *Model fit measures and diagnostics* Residual deviance= 48.85 Data points= 41, Effective number of parameters= 32.9 DIC= 81.76 **B) Major Congential Malformation** (𝜏^2^=0.06, 95% CrI 0.00-0.87) **C) Minor Congential Malformation** (𝜏^2^=0.60, 95% CrI 0.00-5.01) **D) Mother-to-Child Transmission of HIV** (𝜏^2^=0.66, 95% CrI 0.20-2.05) *Model fit measures and diagnostics* Residual deviance= 35.32 Data points= 32, Effective number of parameters= 29.52 DIC= 64.84 **E) Infant and child deaths** (𝜏^2^=0.62, 95% CrI 0.13-2.43) **F) Preterm births** (𝜏^2^=0.70, 95% CrI 0.39-1.27) **G) Stillbirths** (𝜏^2^=0.92, 95% CrI 0.33-2.28) **H) Low birth weight.** (𝜏^2^=0.23, 95% CrI 0.10-0.50) The black horizontal lines represent the credible intervals for the summary odds ratios for each drug category comparison and the red horizontal lines represent the predictive intervals. The blue vertical line is the line of no effect. **Abbreviations:** ART, Antiretroviral Therapy; dual, duo- therapy; CrI, Credible Intervals; CM, Congenital Malformations; DIC, Deviance Information Criterion; HAART, Highly Active Anti-Retroviral; mono, monotherapy; MTCT, Mother- to -Child Transmission of HIV; NoT, No Treatment; OR, Odds Ratio; Plc, Placebo. **Notes:** Inconsistency was found for the following network meta-analyses when considering antiretroviral categories. Results are reported in the following format: *Design-by-treatment interaction model for inconsistency* χ² (d.f., P-value, between-study variance); Mother- to -Child Transmission of HIV 27.22 (11, 0.00, 0.06), Infant and Child Deaths 2.41 (2, 0.30, 0.00), Low Birth Weight 51.19 (11, 0, 0.03), Preterm Births 45.56 (9, 0, 0.14).
