# Supplementary material for: Comparative safety and effectiveness of perinatal antiretroviral therapies for HIV-infected women and their children: Systematic review and network meta-analysis including different study designs
Source: PLoS One. 2018 Jun 18;13(6):e0198447. doi: 10.1371/journal.pone.0198447 (PMC6005568; doi:10.1371/journal.pone.0198447)
Supplement: S20 Appendix — (DOCX) [file pone.0198447.s020.docx]

# S20 Appendix. Characteristics of the treatment nodes per outcome along with their surface under the cumulative ranking curve values

| **Treatment** | **Frequency of Treatment in Network** | **Number of Events/Sample Size*** | **Treatment Group Risk Median (IQR)** | **SUCRA Median (95% CrI)** |
| --- | --- | --- | --- | --- |
| ***Mother-to-child transmission of HIV using specific Antiretroviral Therapy Drugs*** | | | | |
| NoT/Plc + NoT/Plc | 10 | 1455/6230 | 0.19 (0.17 to 0.29) | 0.00 (0.00 to 0.40) |
| NoT/Plc + [ZDV] | 2 | 5/30 | 0.18 (0.15 to 0.20) | 0.20 (0.00 to 1.00) |
| [ZDV] + NoT/Plc | 3 | 54/892 | 0.06 (0.05 to 0.12) | 0.80 (0.20 to 1.00) |
| [ZDV] + [ZDV] | 9 | 71/691 | 0.07 (0.04 to 0.13) | 0.60 (0.20 to 1.00) |
| NoT/Plc + [NVP] | 2 | 274/2418 | 0.13 (0.08 to 0.17) | 0.60 (0.20 to 1.00) |
| [ZDV] + [NVP] | 1 | 103/705 | 0.15 (0.15 to 0.15) | 0.80 (0.20 to 1.00) |
| ***Mother-to-child transmission of HIV using Antiretroviral Therapy Drug Categories*** | | | | |
| NoT/Plc + NoT/Plc | 13 | 1514/6361 | 0.24 (0.17 to 0.31) | 0.00 (0.00 to 0.20) |
| NoT + ART-mono | 4 | 279/2448 | 0.16 (0.11 to 0.19) | 0.40 (0.00 to 0.60) |
| ART-mono + No treatment | 3 | 54/892 | 0.06 (0.05 to 0.12) | 0.40 (0.00 to 0.80) |
| ART-mono + ART-mono | 11 | 178/1490 | 0.07 (0.04 to 0.15) | 0.60 (0.20 to 0.80) |
| ART-dual + No treatment | 1 | 2/283 | 0.01 (0.01 to 0.01) | 1.00 (0.40 to 1.00) |
| HAART + No treatment | 4 | 203/3752 | 0.03 (0.01 to 0.09) | 0.80 (0.60 to 1.00) |
| ***Total Congenital malformations using specific Antiretroviral Therapy Drugs*** | | | | |
| NoT/Plc | 11 | 163/2169 | 0.07 (0.02 to 0.17) | 0.60 (0.27 to 0.87) |
| ZDV | 15 | 128/2606 | 0.05 (0.02 to 0.22) | 0.53 (0.27 to 0.80) |
| d4T | 1 | 3/91 | 0.03 (0.03 to 0.03) | 0.20 (0.00 to 0.80) |
| ddl | 1 | 1/94 | 0.01 (0.01 to 0.01) | 0.53 (0.13 to 1.00) |
| d4T+ddI | 2 | 4/115 | 0.02 (0.00 to 0.04) | 0.13 (0.00 to 0.67) |
| EFV | 2 | 7/214 | 0.03 (0.03 to 0.03) | 0.07 (0.00 to 0.80) |
| 3TC+d4T | 1 | 1/25 | 0.04 (0.04 to 0.04) | 0.80 (0.20 to 1.00) |
| ZDV+3TC | 3 | 46/840 | 0.04 (0.00 to 0.07) | 0.47 (0.20 to 0.87) |
| ZDV+3TC+ABC | 1 | 5/283 | 0.02 (0.02 to 0.02) | 0.73 (0.20 to 1.00) |
| ZDV+3TC+NFV | 1 | 0/3 | 0.00 (0.00 to 0.00) | 0.93 (0.13 to 1.00) |
| NVP | 1 | 1/33 | 0.03 (0.03 to 0.03) | 0.07 (0.00 to 1.00) |
| ZDV+ddI+NVP | 1 | 2/13 | 0.15 (0.15 to 0.15) | 0.33 (0.00 to 0.93) |
| ddI+d4T+NVP | 1 | 2/13 | 0.15 (0.15 to 0.15) | 0.33 (0.00 to 0.93) |
| ZDV+3TC+NVP | 3 | 9/228 | 0.03 (0.00 to 0.06) | 0.53 (0.13 to 0.87) |
| LOP+RIT | 1 | 16/69 | 0.23 (0.23 to 0.23) | 0.87 (0.40 to 1.00) |
| ZDV+3TC+LOP+RIT | 3 | 27/707 | 0.02 (0.02 to 0.39) | 0.73 (0.33 to 0.93) |
| ***Total Congenital malformations using Antiretroviral Therapy Drug Categories*** | | | | |
| NoT/Plc | 11 | 163/2169 | 0.07 (0.03 to 0.15) | 0.67 (0.00 to 1.00) |
| ART-mono | 15 | 127/2858 | 0.03 (0.02 to 0.22) | 0.67 (0.00 to 1.00) |
| ART-dual | 7 | 72/1041 | 0.07 (0.04 to 0.19) | 0.33 (0.00 to 1.00) |
| HAART | 7 | 110/1742 | 0.06 (0.02 to 0.39) | 0.33 (0.00 to 1.00) |
| ***Major Congenital malformations using specific Antiretroviral Therapy Drugs*** | | | | |
| NoT/Plc | **7** | 77/694 | 0.08 (0.04 to 0.17) | 0.43 (0.14 to 0.86) |
| ZDV | 9 | 88/1607 | 0.09 (0.03 to 0.19) | 0.57 (0.14 to 0.86) |
| 3TC+d4T | 1 | 1/25 | 0.04 (0.04 to 0.04) | 0.86 (0.14 to 1.00) |
| ZDV+3TC | 2 | 18/460 | 0.02 (0.00 to 0.04) | 0.29 (0.00 to 0.86) |
| ZDV+3TC+NFV | 1 | 0/3 | 0.00 (0.00 to 0.00) | 0.86 (0.00 to 1.00) |
| ZDV+ddI+NVP | 1 | 2/13 | 0.15 (0.15 to 0.15) | 0.14 (0.00 to 0.86) |
| ddI+d4T+NVP | 1 | 2/13 | 0.15 (0.15 to 0.15) | 0.14 (0.00 to 0.86) |
| ZDV+3TC+NVP | 1 | 0/3 | 0.00 (0.00 to 0.00) | 0.86 (0.00 to 1.00) |
| ***Major Congenital malformations using Antiretroviral Therapy Drug Categories*** | | | | |
| NoT/Plc | 11 | 168/2739 | 0.11 (0.06 to 0.16) | 0.33 (0.00 to 1.00) |
| ART-mono | 10 | 93/1792 | 0.09 (0.03 to 0.19) | 0.67 (0.33 to 1.00) |
| ART-dual | 4 | 24/498 | 0.08 (0.02 to 0.15) | 0.00 (0.00 to 0.67) |
| HAART | 4 | 77/1236 | 0.06 (0.03 to 0.14) | 0.67 (0.00 to 1.00) |
| ***Minor Congenital malformations using specific Antiretroviral Therapy Drugs*** | | | | |
| NoT/Plc | 2 | 0/18 | 0.00 (0.00 to 0.00) | 0.75 (0.00 to 1.00) |
| ZDV | 2 | 5/37 | 0.16 (0.11 to 0.20) | 0.25 (0.00 to 0.75) |
| ZDV+3TC | 1 | 0/8 | 0.00 (0.00 to 0.00) | 0.75 (0.00 to 1.00) |
| ZDV+3TC+NFV | 1 | 0/3 | 0.00 (0.00 to 0.00) | 0.50 (0.00 to 1.00) |
| ZDV+3TC+NVP | 1 | 0/3 | 0.00 (0.00 to 0.00) | 0.50 (0.00 to 1.00) |
| ***Minor Congenital malformations using Antiretroviral Therapy Drug Categories*** | | | | |
| NoT/Plc | 3 | 1/635 | 0.00 (0.00 to 0.00) | 0.67 (0.00 to 1.00) |
| ART-mono | 2 | 5/37 | 0.16 (0.11 to 0.20) | 0.33 (0.00 to 0.67) |
| ART-dual | 1 | 0/8 | 0.00 (0.00 to 0.00) | 1.00 (0.00 to 1.00) |
| HAART | 2 | 7/24 | 0.19 (0.00 to 0.39) | 0.00 (0.00 to 0.67) |
| ***Infant/Child Deaths using specific Antiretroviral Therapy Drugs*** | | | | |
| NoT/Plc | 10 | 271/3105 | 0.04 (0.03 to 0.13) | 0.14 (0.00 to 0.57) |
| ZDV | 10 | 126/5970 | 0.02 (0.04 to 0.07) | 0.43 (0.14 to 0.86) |
| TDF | 1 | 7/111 | 0.06 (0.06 to 0.06) | 0.29 (0.00 to 1.00) |
| ZDV+3TC | 2 | 40/1059 | 0.05 (0.00 to 0.10) | 0.57 (0.00 to 1.00) |
| ZDV+3TC+ABC | 1 | 13/283 | 0.05 (0.05 to 0.05) | 0.71 (0.00 to 1.00) |
| NVP | 1 | 0/30 | 0.00 (0.00 to 0.00) | 1.00 (0.00 to 1.00) |
| ZDV+3TC+NVP | 1 | 9/156 | 0.06 (0.06 to 0.06) | 0.57 (0.00 to 1.00) |
| ZDV+3TC+LOP+RIT | 2 | 39/671 | 0.06 (0.06 to 0.06) | 0.57 (0.00 to 1.00) |
| ***Infant/Child Deaths using Antiretroviral Therapy Drug Categories*** | | | | |
| NoT/Plc | 13 | 283/3322 | 0.04 (0.02 to 0.11) | 0.00 (0.00 to 0.67) |
| ART-mono | 11 | 141/6221 | 0.05 (0.02 to 0.06) | 0.67 (0.00 to 1.00) |
| ART-dual | 3 | 43/1084 | 0.10 (0.00 to 0.12) | 0.67 (0.00 to 1.00) |
| HAART | 6 | 55/4302 | 0.04 (0.00 to 0.06) | 1.00 (0.00 to 1.00) |
| ***Preterms using specific Antiretroviral Therapy Drugs*** | | | | |
| NoT/Plc | 23 | 1854/7380 | 0.19 (0.09 to 0.23) | 0.44 (0.19 to 0.63) |
| ZDV | 23 | 1172/9943 | 0.11 (0.06 to 0.22) | 0.63 (0.38 to 0.81) |
| TDF | 1 | 13/140 | 0.09 (0.09 to 0.09) | 0.50 (0.06 to 0.88) |
| ZDV+3TC+IND | 1 | 2/3 | 0.67 (0.67 to 0.67) | 0.00 (0.00 to 0.19) |
| ZDV+3TC | 2 | 14/46 | 0.22 (0.00 to 0.44) | 0.31 (0.06 to 0.88) |
| 3TC+d4T+IND | 1 | 0/3 | 0.00 (0.00 to 0.00) | 0.19 (0.00 to 0.94) |
| ZDV+3TC+ABC | 3 | 42/290 | 0.00 (0.00 to 0.15) | 0.69 (0.19 to 0.88) |
| 3TC+d4T+EFV | 1 | 23/112 | 0.21 (0.21 to 0.21) | 0.13 (0.00 to 0.75) |
| ZDV+3TC+NLF | 6 | 32/204 | 0.07 (0.00 to 0.18) | 0.63 (0.25 to 0.88) |
| ZDV+NVP | 1 | 2/216 | 0.01 (0.01 to 0.01) | 1.00 (0.94 to 1.00) |
| 3TC+d4T+NVP | 2 | 58/299 | 0.31 (0.18 to 0.44) | 0.19 (0.06 to 0.69) |
| ZDV+3TC+NVP | 5 | 34/293 | 0.10 (0.07 to 0.12) | 0.81 (0.50 to 0.94) |
| LOP+RIT | 1 | 7/69 | 0.10 (0.10 to 0.10) | 0.44 (0.06 to 0.94) |
| ZDV+3TC+LOP+RIT | 7 | 171/978 | 0.17 (0.13 to 0.26) | 0.44 (0.19 to 0.69) |
| 3TC+d4T+LOP+RIT | 1 | 28/421 | 0.07 (0.07 to 0.07) | 0.50 (0.13 to 0.94) |
| ZDV+3TC+LOP+RIT+TDF | 1 | 0/2 | 0.00 (0.00 to 0.00) | 0.94 (0.31 to 1.00) |
| ZDV+3TC+EFV | 1 | 26/177 | 0.15 (0.15 to 0.15) | 0.50 (0.06 to 0.88) |
| ***Preterm using Antiretroviral Therapy Drug Categories*** | | | | |
| NoT/Plc | 32 | 2753/12166 | 0.20 (0.11 to 0.25) | 0.00 (0.00 to 0.33) |
| ART-mono | 31 | 3468/24671 | 0.13 (0.07 to 0.17) | 0.67 (0.33 to 0.67) |
| ART-dual | 10 | 216/1812 | 0.08 (0.00 to 0.14) | 1.00 (1.00 to 1.00) |
| HAART | 33 | 3412/19887 | 0.17 (0.10 to 0.25) | 0.33 (0.00 to 0.67) |
| ***Stillbirths using specific Antiretroviral Therapy Drugs*** | | | | |
| NoT/Plc | 18 | 250/5182 | 0.03 (0.01 to 0.06) | 0.42 (0.16 to 0.68) |
| ZDV | 19 | 114/8116 | 0.01 (0.01 to 0.02) | 0.63 (0.32 to 0.84) |
| d4T | 1 | 0/91 | 0.00 (0.00 to 0.00) | 0.84 (0.16 to 1.00) |
| ddl | 1 | 0/94 | 0.00 (0.00 to 0.00) | 0.84 (0.16 to 1.00) |
| d4T+ddI | 1 | 4/94 | 0.04 (0.04 to 0.04) | 0.32 (0.00 to 0.79) |
| TDF | 1 | 20/251 | 0.08 (0.08 to 0.08) | 0.21 (0.00 to 0.74) |
| ZDV+3TC+IND | 2 | 0/8 | 0.00 (0.00 to 0.00) | 0.32 (0.00 to 0.95) |
| 3TC+d4T | 1 | 0/11 | 0.00 (0.00 to 0.00) | 0.58 (0.05 to 1.00) |
| ZDV+3TC | 5 | 17/1252 | 0.01 (0.00 to 0.04) | 0.58 (0.26 to 0.84) |
| 3TC+d4T+IND | 1 | 0/3 | 0.00 (0.00 to 0.00) | 0.42 (0.00 to 1.00) |
| ZDV+3TC+ABC | 2 | 8/290 | 0.01 (0.00 to 0.03) | 0.47 (0.05 to 0.89) |
| ZDV+3TC+NLF | 2 | 1/10 | 0.13 (0.00 to 0.25) | 0.16 (0.00 to 0.74) |
| ZDV+3TC+SAQ | 1 | 0/2 | 0.00 (0.00 to 0.00) | 0.21 (0.00 to 1.00) |
| 3TC+d4T+SAQ | 1 | 0/3 | 0.00 (0.00 to 0.00) | 0.26 (0.00 to 1.00) |
| NVP | 2 | 2/638 | 0.00 (0.00 to 0.00) | 0.84 (0.37 to 1.00) |
| ZDV+NVP | 1 | 1/216 | 0.00 (0.00 to 0.00) | 0.89 (0.47 to 1.00) |
| 3TC+d4T+NVP | 1 | 0/2 | 0.00 (0.00 to 0.00) | 0.21 (0.00 to 1.00) |
| ZDV+3TC+NVP | 2 | 10/175 | 0.03 (0.00 to 0.06) | 0.26 (0.00 to 0.74) |
| ZDV+3TC+LOP+RIT | 3 | 14/874 | 0.02 (0.01 to 0.03) | 0.58 (0.21 to 0.89) |
| ZDV+3TC+EFV | 1 | 6/195 | 0.03 (0.03 to 0.03) | 0.53 (0.05 to 0.95) |
| ***Stillbirths using Antiretroviral Therapy Drug Categories*** | | | | |
| NoT/Plc | 26 | 297/6490 | 0.03 (0.01 -0.07) | 0.00 (0.00 to 0.33) |
| ART-mono | 25 | 624/22046 | 0.01(0.00-0.04) | 0.67 (0.33 to 1.00) |
| ART-dual | 12 | 31/1825 | 0.01 (0.00-0.04) | 1.00 (0.33 to 1.00) |
| HAART | 14 | 277/8088 | 0.03 (0.01-0.06) | 0.33 (0.00 to 1.00) |
| ***Low birth weight outcomes using specific Antiretroviral Therapy Drugs*** | | | | |
| NoT/Plc | 18 | 582/3625 | 0.16 (0.07 to 0.19) | 0.53 (0.27 to 0.80) |
| ZDV | 21 | 988/14758 | 0.14 (0.07 to 0.19) | 0.67 (0.40 to 0.87) |
| TDF | 1 | 19/130 | 0.15 (0.15 to 0.15) | 0.73 (0.07 to 0.93) |
| ZDV+3TC+IND | 1 | 0/3 | 0.00 (0.00 to 0.00) | 0.87 (0.00 to 1.00) |
| ZDV+3TC | 4 | 59/376 | 0.24 (0.07 to 0.35) | 0.67 (0.20 to 0.87) |
| 3TC+d4T+IND | 1 | 0/3 | 0.00 (0.00 to 0.00) | 0.87 (0.00 to 1.00) |
| ZDV+3TC+ABC | 1 | 37/283 | 0.13 (0.13 to 0.13) | 0.47 (0.07 to 0.87) |
| 3TC+d4T+EFV | 1 | 41/115 | 0.36 (0.36 to 0.36) | 0.00 (0.00 to 0.40) |
| ZDV+3TC+NLF | 4 | 18/107 | 0.31 (0.06 to 0.58) | 0.33 (0.00 to 0.80) |
| NVP | 3 | 46/645 | 0.07 (0.07 to 0.13) | 0.40 (0.07 to 0.80) |
| ZDV+NVP | 1 | 3/216 | 0.01 (0.01 to 0.01) | 0.80 (0.07 to 1.00) |
| 3TC+d4T+NVP | 3 | 79/432 | 0.21 (0.14 to 0.25) | 0.13 (0.00 to 0.60) |
| ZDV+3TC+NVP | 5 | 43/297 | 0.15 (0.12 to 0.33) | 0.40 (0.13 to 0.80) |
| ZDV+3TC+LOP+RIT | 4 | 112/744 | 0.20 (0.14 to 0.29) | 0.33 (0.07 to 0.67) |
| 3TC+d4T+LOP+RIT | 1 | 71/419 | 0.17 (0.17 to 0.17) | 0.20 (0.00 to 0.87) |
| ZDV+3TC+LOP+RIT+TDF | 1 | 0/2 | 0.00 (0.00 to 0.00) | 0.93 (0.07 to 1.00) |
| ***Low Birth Weight outcomes using Antiretroviral Therapy Drug Categories*** | | | | |
| NoT/Plc | 26 | 1521/8126 | 0.17 (0.10 to 0.23) | 0.33 (0.00 to 0.67) |
| ART-mono | 29 | 3575/29758 | 0.14 (0.09 to 0.20) | 1.00 (0.67 to 1.00) |
| ART-dual | 13 | 328/2296 | 0.12 (0.04 to 0.17) | 0.67 (0.33 to 1.00) |
| HAART | 25 | 1178/6462 | 0.19 (0.15 to 0.29) | 0.00 (0.00 to 0.33) |
| **Notes:** Inconsistency was found for the following network meta-analyses with specific antiretroviral drug categories: Mother- to -Child Transmission of HIV, Infant and child deaths, Low Birth Weight, Preterm Births. *:For drug categories within each outcome, the total number of events and sample sizes was used. In particular, we used the entire dataset, even if some studies were included in the meta-analysis and not in the NMA (e.g., studies comparing ART-any). **Abbreviations:** ART, Antiretroviral Therapy; HAART, Highly Active Anti-Retroviral; ABC, Abacavir; ddI, Didanosine; CM, Congenital Malformations; IND, Indinavir; IQR, interquantile range; 3TC, Lamivudine; LOP, Lopinavir; MTCT, Mother-to-Child Transmission of HIV; NVP, Nevirapine; NLF Nelfinavir; NoT, No Treatment; Plc, Placebo; SAQ, Saquinavir; d4T Stavudine; EFV, Sustiva; SUCRA, Surface Under the Cumulative Ranking curve; RIT, Ritonavir; ZDV, Zidovudine. | | | | |
