# Supplementary material for: Comparative safety and effectiveness of perinatal antiretroviral therapies for HIV-infected women and their children: Systematic review and network meta-analysis including different study designs
Source: PLoS One. 2018 Jun 18;13(6):e0198447. doi: 10.1371/journal.pone.0198447 (PMC6005568; doi:10.1371/journal.pone.0198447)
Supplement: S21 Appendix — (DOCX) [file pone.0198447.s021.docx]

# S21 Appendix. Additional Analyses: Specific Drugs

**Subgroup, Sensitivity, Meta-regression, and Schmitz Network Meta-analysis results (All outcomes) Including Specific Antiretroviral Therapy Drugs**

| ***Treatment Comparison*** | ***NMA Odds Ratio Estimate (Credible Interval)*** | ***Predictive Intervals*** |
| --- | --- | --- |
| ***Subgroup Analysis: Large Studies Only (>300 patients per study)*** | | |
| **Total Congenital Malformations** | | |
| ZDV vs NoT/Plc | 1.06 (0.47 to 4.00) | 0.19 to 10.92 |
| d4T vs NoT/Plc | 4.59 (0.28 to 229.10) | 0.19 to 317.70 |
| d4T vs ZDV | 4.05 (0.27 to 167.90) | 0.18 to 234.60 |
| ddl vs NoT/Plc | 1.17 (0.02 to 68.80) | 0.02 to 99.34 |
| ddl vs ZDV | 1.04 (0.02 to 50.50) | 0.02 to 68.51 |
| ddl vs d4T | 0.25 (0.01 to 3.90) | 0.00 to 6.08 |
| d4T+ddI vs NoT/Plc | 6.29 (0.38 to 307.10) | 0.27 to 410.10 |
| d4T+ddI vs ZDV | 5.66 (0.38 to 222.50) | 0.25 to 336.80 |
| d4T+ddI vs d4T | 1.36 (0.14 to 14.05) | 0.09 to 21.87 |
| d4T+ddI vs ddl | 5.49 (0.38 to 198.40) | 0.24 to 293.30 |
| ZDV+3TC vs NoT/Plc | 1.42 (0.46 to 6.47) | 0.20 to 14.85 |
| ZDV+3TC vs ZDV | 1.35 (0.32 to 4.62) | 0.14 to 10.03 |
| ZDV+3TC vs d4T | 0.32 (0.01 to 6.15) | 0.00 to 9.24 |
| ZDV+3TC vs ddl | 1.27 (0.02 to 78.71) | 0.02 to 102.90 |
| ZDV+3TC vs d4T+ddI | 0.23 (0.00 to 4.34) | 0.00 to 6.69 |
| ZDV+3TC+ABC vs NoT/Plc | 0.61 (0.04 to 13.23) | 0.03 to 21.33 |
| ZDV+3TC+ABC vs ZDV | 0.57 (0.04 to 8.13) | 0.02 to 12.59 |
| ZDV+3TC+ABC vs d4T | 0.13 (0.00 to 6.34) | 0.00 to 8.36 |
| ZDV+3TC+ABC vs ddl | 0.56 (0.00 to 63.23) | 0.00 to 82.03 |
| ZDV+3TC+ABC vs d4T+ddI | 0.10 (0.00 to 4.39) | 0.00 to 5.46 |
| ZDV+3TC+ABC vs ZDV+3TC | 0.42 (0.02 to 9.13) | 0.01 to 13.88 |
| ZDV+3TC+NVP vs NoT/Plc | 1.13 (0.07 to 27.01) | 0.05 to 40.60 |
| ZDV+3TC+NVP vs ZDV | 1.04 (0.06 to 15.79) | 0.04 to 25.26 |
| ZDV+3TC+NVP vs d4T | 0.24 (0.00 to 11.43) | 0.00 to 16.06 |
| ZDV+3TC+NVP vs ddl | 1.04 (0.01 to 112.90) | 0.01 to 144.80 |
| ZDV+3TC+NVP vs d4T+ddI | 0.18 (0.00 to 8.32) | 0.00 to 11.51 |
| ZDV+3TC+NVP vs ZDV+3TC | 0.78 (0.04 to 17.34) | 0.03 to 25.60 |
| ZDV+3TC+NVP vs ZDV+3TC+ABC | 1.83 (0.24 to 14.20) | 0.14 to 27.68 |
| ZDV+3TC+LOP+RIT vs NoT/Plc | 0.65 (0.10 to 6.80) | 0.06 to 11.85 |
| ZDV+3TC+LOP+RIT vs ZDV | 0.60 (0.09 to 3.78) | 0.05 to 7.24 |
| ZDV+3TC+LOP+RIT vs d4T | 0.14 (0.00 to 3.83) | 0.00 to 5.55 |
| ZDV+3TC+LOP+RIT vs ddl | 0.60 (0.01 to 43.88) | 0.01 to 58.46 |
| ZDV+3TC+LOP+RIT vs d4T+ddI | 0.11 (0.00 to 2.68) | 0.00 to 3.91 |
| ZDV+3TC+LOP+RIT vs ZDV+3TC | 0.45 (0.05 to 4.82) | 0.03 to 8.05 |
| ZDV+3TC+LOP+RIT vs ZDV+3TC+ABC | 1.07 (0.13 to 8.19) | 0.08 to 14.91 |
| ZDV+3TC+LOP+RIT vs ZDV+3TC+NVP | 0.57 (0.08 to 4.70) | 0.04 to 7.97 |
| *Common within-network between-study variance* | 0.29 (0.00 to 2.99) |  |
| *Design-by-treatment interaction model for inconsistency χ² (d.f., P-value, between-study variance)* | 0.85 (1, 0.36, 0.23) | |
| **Major Congenital Malformations** | | |
| ZDV vs NoT/PLC | 1.01 (0.30 to 4.95) | 0.13 to 11.77 |
| ZDV+3TCvs NoT/PLC | 2.11 (0.23 to 25.69) | 0.12 to 47.59 |
| ZDV+3TCvs ZDV | 2.06 (0.30 to 13.57) | 0.14 to 30.18 |
| *Common within-network between-study variance* | 0.28 (0.00 to 3.73) | 0.00 to 0.00 |
| *Design-by-treatment interaction model for inconsistency χ² (d.f., P-value, between-study variance)* | Not Applicable - No closed loops | |
| **Mother-to-child transmission of HIV** | | |
| [ZDV]+[NoT] vs [NoT/PLC]+[NoT/PLC] | 0.38 (0.08 to 1.83) | 0.04 to 3.29 |
| [ZDV]+[ZDV] vs [NoT/PLC]+[NoT/PLC] | 0.43 (0.08 to 2.12) | 0.05 to 3.84 |
| [ZDV]+[ZDV] vs [ZDV]+[NoT] | 1.14 (0.11 to 10.84) | 0.07 to 15.72 |
| [NoT]+[NVP] vs [NoT/PLC]+[NoT/PLC] | 0.43 (0.13 to 1.43) | 0.06 to 3.00 |
| [NoT]+[NVP] vs [ZDV]+[NoT] | 1.13 (0.15 to 8.27) | 0.09 to 14.16 |
| [NoT]+[NVP] vs [ZDV]+[ZDV] | 0.99 (0.13 to 7.60) | 0.08 to 13.67 |
| [ZDV]+[NVP] vs [NoT/PLC]+[NoT/PLC] | 0.35 (0.08 to 1.56) | 0.04 to 2.91 |
| [ZDV]+[NVP] vs [ZDV]+[NoT] | 0.93 (0.11 to 8.09) | 0.07 to 13.57 |
| [ZDV]+[NVP] vs [ZDV]+[ZDV] | 0.81 (0.10 to 7.84) | 0.06 to 12.69 |
| [ZDV]+[NVP] vs [NoT]+[NVP] | 0.82 (0.20 to 3.65) | 0.10 to 7.24 |
| *Common within-network between-study variance* | 0.16 (0.00 to 3.15) |  |
| *Design-by-treatment interaction model for inconsistency χ² (d.f., P-value, between-study variance)* | Not Applicable - No independent closed loops | |
| **Preterm Births** | | |
| ZDV vs NoT/PLC | **0.54 (0.33 to 0.86)** | - |
| ZDV+3TC+ABC vs NoT/PLC | 0.38 (0.04 to 3.15) | - |
| ZDV+3TC+ABC vs ZDV | 0.72 (0.08 to 5.55) | - |
| 3TC+d4T+EFV vs NoT/PLC | 88.01 (0.00 to 1.95E + 20) | - |
| 3TC+d4T+EFV vs ZDV | 162.70 (0.00 to 4.74E+20) | - |
| 3TC+d4T+EFV vs ZDV+3TC+ABC | 212.90 (0.00 to 6.79E+20) | - |
| ZDV+NVP vs NoT/PLC | **0.00 (0.00 to 0.03)** | - |
| ZDV+NVP vs ZDV | **0.01 (0.00 to 0.05)** | - |
| ZDV+NVP vs ZDV+3TC+ABC | **0.01 (0.00 to 0.19)** | - |
| ZDV+NVP vs 3TC+d4T+EFV | 0.00 (0.00 to 9.71E+25) | - |
| 3TC+d4T+NVP vs NoT/PLC | 72.87 (0.00 to 1.97E+20) | - |
| 3TC+d4T+NVP vs ZDV | 126.20 (0.00 to 4.01E+20) | - |
| 3TC+d4T+NVP vs ZDV+3TC+ABC | 160.50 (0.00 to 7.37E+20) | - |
| 3TC+d4T+NVP vs 3TC+d4T+EFV | 0.87 (0.19 to 4.07) | - |
| 3TC+d4T+NVP vs ZDV+NVP | 14670.00 (0.00 to 5.58E+22) | - |
| ZDV+3TC+NVP vs NoT/PLC | 0.24 (0.03 to 2.19) | - |
| ZDV+3TC+NVP vs ZDV | 0.45 (0.05 to 3.77) | - |
| ZDV+3TC+NVP vs ZDV+3TC+ABC | 0.65 (0.13 to 2.84) | - |
| ZDV+3TC+NVP vs 3TC+d4T+EFV | 0.00 (0.00 to 9.14E+27) | - |
| ZDV+3TC+NVP vs ZDV+NVP | **61.07 (3.12 to 1443.00)** | - |
| ZDV+3TC+NVP vs 3TC+d4T+NVP | 0.00 (0.00 to 1.06E+28) | - |
| ZDV+3TC+LOP+RIT vs NoT/PLC | 0.66 (0.14 to 2.76) | - |
| ZDV+3TC+LOP+RIT vs ZDV | 1.21 (0.28 to 4.94) | - |
| ZDV+3TC+LOP+RIT vs ZDV+3TC+ABC | 1.71 (0.38 to 7.36) | - |
| ZDV+3TC+LOP+RIT vs 3TC+d4T+EFV | 0.01 (0.00 to 2.29E+28) | - |
| ZDV+3TC+LOP+RIT vs ZDV+NVP | **164.10 (14.07 to 2475.00)** | - |
| ZDV+3TC+LOP+RIT vs 3TC+d4T+NVP | 0.01 (0.00 to 3.38E+28) | - |
| ZDV+3TC+LOP+RIT vs ZDV+3TC+NVP | 2.67 (0.56 to 13.07) | - |
| 3TC+d4T+LOP+RIT vs NoT/PLC | 22.55 (0.00 to 5.30E+19) | - |
| 3TC+d4T+LOP+RIT vs ZDV | 42.31 (0.00 to 1.04E+20) | - |
| 3TC+d4T+LOP+RIT vs ZDV+3TC+ABC | 51.59 (0.00 to 2.61E+20) | - |
| 3TC+d4T+LOP+RIT vs 3TC+d4T+EFV | 0.28 (0.06 to 1.33) | - |
| 3TC+d4T+LOP+RIT vs ZDV+NVP | 5403.00 (0.00 to 1.33E+22) | - |
| 3TC+d4T+LOP+RIT vs 3TC+d4T+NVP | 0.32 (0.07 to 1.39) | - |
| 3TC+d4T+LOP+RIT vs ZDV+3TC+NVP | 101.00 (0.00 to 4.14E+20) | - |
| 3TC+d4T+LOP+RIT vs ZDV+3TC+LOP+RIT | 29.93 (0.00 to 1.21E+20) | - |
| ZDV+3TC+EFV vs NoT/PLC | 0.57 (0.07 to 5.19) | - |
| ZDV+3TC+EFV vs ZDV | 1.05 (0.13 to 9.39) | - |
| ZDV+3TC+EFV vs ZDV+3TC+ABC | 1.48 (0.17 to 12.85) | - |
| ZDV+3TC+EFV vs 3TC+d4T+EFV | 0.01 (0.00 to 1.61E+28) | - |
| ZDV+3TC+EFV vs ZDV+NVP | **143.20 (7.66 to 3302.00)** | - |
| ZDV+3TC+EFV vs 3TC+d4T+NVP | 0.01 (0.00 to 2.14E+28) | - |
| ZDV+3TC+EFV vs ZDV+3TC+NVP | 2.37 (0.27 to 21.43) | - |
| ZDV+3TC+EFV vs ZDV+3TC+LOP+RIT | 0.88 (0.19 to 4.16) | - |
| ZDV+3TC+EFV vs 3TC+d4T+LOP+RIT | 0.02 (0.00 to 6.00E+28) | - |
| *Common within-network between-study variance* | 0.44 (0.17 to 1.31) |  |
| *Design-by-treatment interaction model for inconsistency χ² (d.f., P-value, between-study variance)* | N/A | |
| ***Subgroup Analysis: Infants only*** | | |
| **Infant and Child Deaths** | | |
| ZDV vs NoT/PLC | 0.68 (0.27 to 2.30) | 0.07 to 9.43 |
| TDF vs NoT/PLC | 0.62 (0.06 to 7.29) | 0.03 to 15.49 |
| TDF vs ZDV | 0.92 (0.06 to 11.35) | 0.03 to 24.25 |
| ZDV+3TC vs NoT/PLC | 0.71 (0.08 to 6.30) | 0.03 to 14.56 |
| ZDV+3TC vs ZDV | 1.06 (0.08 to 9.77) | 0.03 to 22.51 |
| ZDV+3TC vs TDF | 1.12 (0.04 to 27.49) | 0.02 to 52.71 |
| NVP vs NoT/PLC | 0.06 (0.00 to 3.60) | 0.00 to 5.77 |
| NVP vs ZDV | 0.09 (0.00 to 4.10) | 0.00 to 7.36 |
| NVP vs TDF | 0.10 (0.00 to 11.08) | 0.00 to 16.47 |
| NVP vs ZDV+3TC | 0.09 (0.00 to 8.36) | 0.00 to 14.56 |
| ZDV+3TC+LOP+RIT vs NoT/PLC | 0.41 (0.04 to 5.46) | 0.02 to 13.41 |
| ZDV+3TC+LOP+RIT vs ZDV | 0.60 (0.06 to 5.64) | 0.03 to 13.81 |
| ZDV+3TC+LOP+RIT vs TDF | 0.65 (0.02 to 24.02) | 0.01 to 43.60 |
| ZDV+3TC+LOP+RIT vs ZDV+3TC | 0.57 (0.03 to 18.26) | 0.01 to 33.03 |
| ZDV+3TC+LOP+RIT vs NVP | 6.69 (0.09 to 5717.00) | 0.06 to 7969.00 |
| *Common within-network between-study variance* | 0.73 (0.01 to 4.02) |  |
| *Design-by-treatment interaction model for inconsistency χ² (d.f., P-value, between-study variance)* | N/A | |
| ***Meta-regression Analysis: Baseline Risk*** | | |
| **Total Congenital Malformations** | | |
| ZDV vs NoT/Plc | 0.67 (0.19 to 2.38) | 0.12 to 3.63 |
| d4T vs NoT/Plc | 2.83 (0.16 to 117.20) | 0.13 to 133.70 |
| d4T vs ZDV | 4.19 (0.31 to 146.00) | 0.25 to 171.70 |
| ddl vs NoT/Plc | 0.66 (0.01 to 33.61) | 0.01 to 38.71 |
| ddl vs ZDV | 0.98 (0.02 to 45.19) | 0.02 to 52.27 |
| ddl vs d4T | 0.24 (0.01 to 3.10) | 0.01 to 3.93 |
| d4T+ddI vs NoT/Plc | 3.83 (0.25 to 135.40) | 0.20 to 163.20 |
| d4T+ddI vs ZDV | 5.74 (0.51 to 181.30) | 0.38 to 214.60 |
| d4T+ddI vs d4T | 1.35 (0.19 to 10.42) | 0.14 to 13.31 |
| d4T+ddI vs ddl | 5.61 (0.53 to 230.20) | 0.42 to 270.50 |
| EFV vs NoT/Plc | 17.25 (0.14 to 20800.00) | 0.12 to 22880.00 |
| EFV vs ZDV | 24.97 (0.26 to 27270.00) | 0.23 to 27240.00 |
| EFV vs d4T | 5.37 (0.08 to 3773.00) | 0.07 to 3713.00 |
| EFV vs ddl | 24.33 (0.30 to 29800.00) | 0.26 to 32500.00 |
| EFV vs d4T+ddI | 3.72 (0.10 to 2253.00) | 0.08 to 2398.00 |
| 3TC+d4T vs NoT/Plc | 0.27 (0.01 to 4.06) | 0.01 to 4.97 |
| 3TC+d4T vs ZDV | 0.41 (0.01 to 4.22) | 0.01 to 5.53 |
| 3TC+d4T vs d4T | 0.08 (0.00 to 3.47) | 0.00 to 4.24 |
| 3TC+d4T vs ddl | 0.38 (0.00 to 43.17) | 0.00 to 47.24 |
| 3TC+d4T vs d4T+ddI | 0.06 (0.00 to 2.17) | 0.00 to 2.58 |
| 3TC+d4T vs EFV | 0.01 (0.00 to 2.81) | 0.00 to 3.18 |
| ZDV+3TC vs NoT/Plc | 0.69 (0.13 to 2.78) | 0.09 to 3.94 |
| ZDV+3TC vs ZDV | 1.03 (0.36 to 2.20) | 0.20 to 3.87 |
| ZDV+3TC vs d4T | 0.24 (0.01 to 3.69) | 0.01 to 4.48 |
| ZDV+3TC vs ddl | 1.02 (0.02 to 50.53) | 0.02 to 57.96 |
| ZDV+3TC vs d4T+ddI | 0.17 (0.00 to 2.24) | 0.00 to 2.82 |
| ZDV+3TC vs EFV | 0.04 (0.00 to 4.13) | 0.00 to 4.61 |
| ZDV+3TC vs 3TC+d4T | 2.47 (0.18 to 95.15) | 0.14 to 107.10 |
| ZDV+3TC+ABC vs NoT/Plc | 0.38 (0.04 to 3.31) | 0.03 to 4.06 |
| ZDV+3TC+ABC vs ZDV | 0.57 (0.08 to 3.40) | 0.06 to 4.66 |
| ZDV+3TC+ABC vs d4T | 0.13 (0.00 to 3.14) | 0.00 to 3.81 |
| ZDV+3TC+ABC vs ddl | 0.59 (0.01 to 40.63) | 0.01 to 47.28 |
| ZDV+3TC+ABC vs d4T+ddI | 0.10 (0.00 to 2.07) | 0.00 to 2.63 |
| ZDV+3TC+ABC vs EFV | 0.02 (0.00 to 2.78) | 0.00 to 3.24 |
| ZDV+3TC+ABC vs 3TC+d4T | 1.44 (0.07 to 73.97) | 0.05 to 81.95 |
| ZDV+3TC+ABC vs ZDV+3TC | 0.55 (0.08 to 4.67) | 0.06 to 6.44 |
| ZDV+3TC+NFV vs NoT/Plc | 0.11 (0.00 to 4.16) | 0.00 to 4.92 |
| ZDV+3TC+NFV vs ZDV | 0.16 (0.00 to 3.80) | 0.00 to 4.44 |
| ZDV+3TC+NFV vs d4T | 0.03 (0.00 to 2.58) | 0.00 to 2.97 |
| ZDV+3TC+NFV vs ddl | 0.14 (0.00 to 22.27) | 0.00 to 24.58 |
| ZDV+3TC+NFV vs d4T+ddI | 0.02 (0.00 to 1.67) | 0.00 to 1.87 |
| ZDV+3TC+NFV vs EFV | 0.00 (0.00 to 1.67) | 0.00 to 1.81 |
| ZDV+3TC+NFV vs 3TC+d4T | 0.39 (0.00 to 41.34) | 0.00 to 47.94 |
| ZDV+3TC+NFV vs ZDV+3TC | 0.16 (0.00 to 4.27) | 0.00 to 5.14 |
| ZDV+3TC+NFV vs ZDV+3TC+ABC | 0.28 (0.00 to 10.94) | 0.00 to 12.48 |
| NVP vs NoT/Plc | 10.31 (0.03 to 18870.00) | 0.03 to 21400.00 |
| NVP vs ZDV | 15.41 (0.06 to 24100.00) | 0.06 to 26510.00 |
| NVP vs d4T | 3.37 (0.02 to 3527.00) | 0.02 to 3452.00 |
| NVP vs ddl | 14.61 (0.06 to 26100.00) | 0.05 to 29890.00 |
| NVP vs d4T+ddI | 2.37 (0.02 to 2162.00) | 0.02 to 2257.00 |
| NVP vs EFV | 0.68 (0.02 to 6.81) | 0.02 to 8.83 |
| NVP vs 3TC+d4T | 45.49 (0.09 to 97310.00) | 0.08 to 101200.00 |
| NVP vs ZDV+3TC | 15.26 (0.06 to 25370.00) | 0.05 to 26940.00 |
| NVP vs ZDV+3TC+ABC | 27.47 (0.08 to 52040.00) | 0.07 to 54220.00 |
| NVP vs ZDV+3TC+NFV | 133.70 (0.16 to 1100000.00) | 0.14 to 1208000.00 |
| ZDV+ddI+NVP vs NoT/Plc | 1.51 (0.10 to 16.43) | 0.08 to 20.64 |
| ZDV+ddI+NVP vs ZDV | 2.28 (0.22 to 17.42) | 0.17 to 22.62 |
| ZDV+ddI+NVP vs d4T | 0.50 (0.01 to 14.87) | 0.01 to 18.32 |
| ZDV+ddI+NVP vs ddl | 2.23 (0.03 to 196.30) | 0.03 to 229.70 |
| ZDV+ddI+NVP vs d4T+ddI | 0.38 (0.01 to 9.88) | 0.01 to 11.68 |
| ZDV+ddI+NVP vs EFV | 0.09 (0.00 to 13.55) | 0.00 to 15.87 |
| ZDV+ddI+NVP vs 3TC+d4T | 5.59 (0.32 to 239.60) | 0.26 to 301.40 |
| ZDV+ddI+NVP vs ZDV+3TC | 2.25 (0.20 to 22.81) | 0.16 to 28.82 |
| ZDV+ddI+NVP vs ZDV+3TC+ABC | 3.96 (0.21 to 65.18) | 0.16 to 82.34 |
| ZDV+ddI+NVP vs ZDV+3TC+NFV | 14.95 (0.26 to 16370.00) | 0.24 to 18410.00 |
| ZDV+ddI+NVP vs NVP | 0.14 (0.00 to 56.50) | 0.00 to 60.08 |
| ddI+d4T+NVP vs NoT/Plc | 1.51 (0.11 to 16.35) | 0.09 to 20.20 |
| ddI+d4T+NVP vs ZDV | 2.28 (0.22 to 17.31) | 0.17 to 23.39 |
| ddI+d4T+NVP vs d4T | 0.50 (0.01 to 14.83) | 0.01 to 16.78 |
| ddI+d4T+NVP vs ddl | 2.22 (0.03 to 189.80) | 0.03 to 220.10 |
| ddI+d4T+NVP vs d4T+ddI | 0.37 (0.01 to 9.80) | 0.01 to 11.48 |
| ddI+d4T+NVP vs EFV | 0.09 (0.00 to 12.82) | 0.00 to 15.26 |
| ddI+d4T+NVP vs 3TC+d4T | 5.66 (0.29 to 225.70) | 0.24 to 279.30 |
| ddI+d4T+NVP vs ZDV+3TC | 2.22 (0.19 to 22.34) | 0.15 to 30.54 |
| ddI+d4T+NVP vs ZDV+3TC+ABC | 4.00 (0.21 to 63.02) | 0.16 to 79.09 |
| ddI+d4T+NVP vs ZDV+3TC+NFV | 14.76 (0.25 to 15360.00) | 0.22 to 16350.00 |
| ddI+d4T+NVP vs NVP | 0.14 (0.00 to 50.27) | 0.00 to 55.15 |
| ddI+d4T+NVP vs ZDV+ddI+NVP | 0.99 (0.07 to 13.55) | 0.06 to 17.27 |
| ZDV+3TC+NVP vs NoT/Plc | 0.70 (0.12 to 3.47) | 0.08 to 4.75 |
| ZDV+3TC+NVP vs ZDV | 1.04 (0.28 to 3.61) | 0.18 to 5.25 |
| ZDV+3TC+NVP vs d4T | 0.24 (0.01 to 4.42) | 0.01 to 5.45 |
| ZDV+3TC+NVP vs ddl | 1.06 (0.02 to 60.61) | 0.02 to 69.40 |
| ZDV+3TC+NVP vs d4T+ddI | 0.18 (0.00 to 2.86) | 0.00 to 3.41 |
| ZDV+3TC+NVP vs EFV | 0.04 (0.00 to 4.54) | 0.00 to 5.12 |
| ZDV+3TC+NVP vs 3TC+d4T | 2.58 (0.17 to 111.90) | 0.14 to 135.70 |
| ZDV+3TC+NVP vs ZDV+3TC | 1.02 (0.23 to 5.20) | 0.16 to 7.53 |
| ZDV+3TC+NVP vs ZDV+3TC+ABC | 1.83 (0.37 to 9.88) | 0.25 to 13.61 |
| ZDV+3TC+NVP vs ZDV+3TC+NFV | 6.49 (0.21 to 6083.00) | 0.19 to 6699.00 |
| ZDV+3TC+NVP vs NVP | 0.07 (0.00 to 18.30) | 0.00 to 21.34 |
| ZDV+3TC+NVP vs ZDV+ddI+NVP | 0.46 (0.04 to 6.45) | 0.03 to 7.97 |
| ZDV+3TC+NVP vs ddI+d4T+NVP | 0.45 (0.04 to 6.49) | 0.03 to 8.05 |
| LOP+RIT vs NoT/Plc | 0.19 (0.02 to 1.74) | 0.01 to 2.31 |
| LOP+RIT vs ZDV | 0.29 (0.04 to 1.82) | 0.03 to 2.51 |
| LOP+RIT vs d4T | 0.07 (0.00 to 1.61) | 0.00 to 1.93 |
| LOP+RIT vs ddl | 0.30 (0.00 to 20.75) | 0.00 to 22.80 |
| LOP+RIT vs d4T+ddI | 0.05 (0.00 to 1.10) | 0.00 to 1.32 |
| LOP+RIT vs EFV | 0.01 (0.00 to 1.58) | 0.00 to 1.84 |
| LOP+RIT vs 3TC+d4T | 0.72 (0.03 to 40.09) | 0.03 to 42.89 |
| LOP+RIT vs ZDV+3TC | 0.28 (0.04 to 2.49) | 0.03 to 3.52 |
| LOP+RIT vs ZDV+3TC+ABC | 0.50 (0.06 to 4.35) | 0.04 to 5.63 |
| LOP+RIT vs ZDV+3TC+NFV | 1.82 (0.05 to 1990.00) | 0.04 to 2144.00 |
| LOP+RIT vs NVP | 0.02 (0.00 to 6.36) | 0.00 to 7.23 |
| LOP+RIT vs ZDV+ddI+NVP | 0.12 (0.01 to 2.40) | 0.01 to 2.95 |
| LOP+RIT vs ddI+d4T+NVP | 0.13 (0.01 to 2.44) | 0.01 to 2.84 |
| LOP+RIT vs ZDV+3TC+NVP | 0.28 (0.04 to 1.88) | 0.03 to 2.72 |
| ZDV+3TC+LOP+RIT vs NoT/Plc | 0.40 (0.07 to 2.13) | 0.05 to 3.08 |
| ZDV+3TC+LOP+RIT vs ZDV | 0.60 (0.17 to 2.00) | 0.11 to 3.16 |
| ZDV+3TC+LOP+RIT vs d4T | 0.14 (0.00 to 2.38) | 0.00 to 2.88 |
| ZDV+3TC+LOP+RIT vs ddl | 0.62 (0.01 to 35.45) | 0.01 to 42.57 |
| ZDV+3TC+LOP+RIT vs d4T+ddI | 0.10 (0.00 to 1.57) | 0.00 to 1.94 |
| ZDV+3TC+LOP+RIT vs EFV | 0.02 (0.00 to 2.64) | 0.00 to 2.87 |
| ZDV+3TC+LOP+RIT vs 3TC+d4T | 1.48 (0.10 to 61.64) | 0.08 to 75.53 |
| ZDV+3TC+LOP+RIT vs ZDV+3TC | 0.59 (0.14 to 3.00) | 0.10 to 4.52 |
| ZDV+3TC+LOP+RIT vs ZDV+3TC+ABC | 1.05 (0.21 to 5.52) | 0.15 to 8.01 |
| ZDV+3TC+LOP+RIT vs ZDV+3TC+NFV | 3.78 (0.13 to 3361.00) | 0.10 to 3625.00 |
| ZDV+3TC+LOP+RIT vs NVP | 0.04 (0.00 to 11.07) | 0.00 to 13.05 |
| ZDV+3TC+LOP+RIT vs ZDV+ddI+NVP | 0.27 (0.03 to 3.58) | 0.02 to 4.60 |
| ZDV+3TC+LOP+RIT vs ddI+d4T+NVP | 0.26 (0.02 to 3.58) | 0.02 to 4.60 |
| ZDV+3TC+LOP+RIT vs ZDV+3TC+NVP | 0.57 (0.16 to 2.14) | 0.10 to 3.27 |
| ZDV+3TC+LOP+RIT vs LOP+RIT | 2.11 (0.51 to 8.84) | 0.34 to 13.55 |
| *Common within-network between-study variance* | 0.19 (0.00 to 1.23) |  |
| *Regression coefficient* | 0.80 (0.53 to 1.26) |  |
| *Design-by-treatment interaction model for inconsistency χ² (d.f., P-value, between-study variance)* | 7.93, 6, 0.24, 0.000 | |
| *Model fit measures and diagnostics* | Residual deviance = 49.94 Data points = 48  Effective number of parameters = 42.39 DIC = 92.33 | |
| **Mother-to-child transmission of HIV** | | |
| [NoT]+[ZDV] vs [NoT/PLC]+[NoT/PLC] | 1.17 (0.06 to 8.58) | 0.05 to 11.32 |
| [ZDV]+[NoT] vs [NoT/PLC]+[NoT/PLC] | 0.54 (0.02 to 2.94) | 0.02 to 3.77 |
| [ZDV]+[NoT] vs [NoT]+[ZDV] | 0.42 (0.09 to 2.05) | 0.06 to 2.67 |
| [ZDV]+[ZDV] vs [NoT/PLC]+[NoT/PLC] | 0.53 (0.04 to 1.96) | 0.03 to 2.64 |
| [ZDV]+[ZDV] vs [NoT]+[ZDV] | 0.43 (0.10 to 1.87) | 0.06 to 2.57 |
| [ZDV]+[ZDV] vs [ZDV]+[NoT] | 1.02 (0.35 to 2.65) | 0.20 to 4.47 |
| [NoT]+[NVP] vs [NoT/PLC]+[NoT/PLC] | 0.57 (0.02 to 2.93) | 0.02 to 3.84 |
| [NoT]+[NVP] vs [NoT]+[ZDV] | 0.44 (0.07 to 2.55) | 0.05 to 3.57 |
| [NoT]+[NVP] vs [ZDV]+[NoT] | 1.04 (0.29 to 3.63) | 0.18 to 5.78 |
| [NoT]+[NVP] vs [ZDV]+[ZDV] | 1.01 (0.30 to 3.63) | 0.19 to 6.08 |
| [ZDV]+[NVP] vs [NoT/PLC]+[NoT/PLC] | 0.46 (0.02 to 2.43) | 0.02 to 3.33 |
| [ZDV]+[NVP] vs [NoT]+[ZDV] | 0.36 (0.05 to 2.34) | 0.04 to 3.18 |
| [ZDV]+[NVP] vs [ZDV]+[NoT] | 0.84 (0.20 to 3.60) | 0.13 to 5.77 |
| [ZDV]+[NVP] vs [ZDV]+[ZDV] | 0.82 (0.23 to 3.54) | 0.15 to 5.69 |
| [ZDV]+[NVP] vs [NoT]+[NVP] | 0.82 (0.27 to 2.71) | 0.16 to 4.41 |
| *Common within-network between-study variance* | 0.15 (0.00 to 1.52) |  |
| *Regression coefficient* | 1.16 (0.27 to 2.25) |  |
| *Design-by-treatment interaction model for inconsistency χ² (d.f., P-value, between-study variance)* | 26.52 (5, 0.00, 0.00) | |
| *Model fit measures and diagnostics* | Residual deviance = 27.55 Data points = 25  Effective number of parameters = 20.65 DIC = 48.20 | |
| ***Meta-regression Analysis: Age*** | | |
| **Total Congenital Malformations** | | |
| ZDV vs NoT/Plc | 0.84 (0.42 to 1.74) | 0.19 to 3.81 |
| d4T vs NoT/Plc | 3.55 (0.25 to 172.70) | 0.18 to 219.10 |
| d4T vs ZDV | 4.18 (0.32 to 192.00) | 0.23 to 229.30 |
| ddl vs NoT/Plc | 0.84 (0.02 to 49.46) | 0.01 to 59.39 |
| ddl vs ZDV | 1.00 (0.02 to 56.90) | 0.02 to 66.60 |
| ddl vs d4T | 0.24 (0.01 to 3.10) | 0.01 to 4.07 |
| d4T+ddI vs NoT/Plc | 4.76 (0.37 to 232.90) | 0.28 to 283.40 |
| d4T+ddI vs ZDV | 5.61 (0.49 to 269.10) | 0.37 to 311.30 |
| d4T+ddI vs d4T | 1.35 (0.19 to 11.61) | 0.14 to 15.67 |
| d4T+ddI vs ddl | 5.64 (0.50 to 191.50) | 0.38 to 238.40 |
| EFV vs NoT/Plc | 26.34 (0.20 to 28390.00) | 0.17 to 32220.00 |
| EFV vs ZDV | 31.27 (0.25 to 31700.00) | 0.22 to 36400.00 |
| EFV vs d4T | 6.72 (0.08 to 4487.00) | 0.07 to 5098.00 |
| EFV vs ddl | 31.70 (0.26 to 29050.00) | 0.23 to 33000.00 |
| EFV vs d4T+ddI | 4.60 (0.09 to 2146.00) | 0.08 to 2370.00 |
| 3TC+d4T vs NoT/Plc | 0.36 (0.01 to 4.41) | 0.01 to 6.34 |
| 3TC+d4T vs ZDV | 0.43 (0.01 to 4.64) | 0.01 to 6.09 |
| 3TC+d4T vs d4T | 0.09 (0.00 to 3.53) | 0.00 to 4.26 |
| 3TC+d4T vs ddl | 0.39 (0.00 to 39.26) | 0.00 to 43.68 |
| 3TC+d4T vs d4T+ddI | 0.07 (0.00 to 2.28) | 0.00 to 2.75 |
| 3TC+d4T vs EFV | 0.01 (0.00 to 2.91) | 0.00 to 3.34 |
| ZDV+3TC vs NoT/Plc | 1.27 (0.46 to 3.69) | 0.25 to 7.09 |
| ZDV+3TC vs ZDV | 1.52 (0.53 to 4.37) | 0.30 to 8.55 |
| ZDV+3TC vs d4T | 0.36 (0.01 to 5.96) | 0.01 to 7.22 |
| ZDV+3TC vs ddl | 1.49 (0.02 to 77.83) | 0.02 to 95.50 |
| ZDV+3TC vs d4T+ddI | 0.26 (0.00 to 3.83) | 0.00 to 5.12 |
| ZDV+3TC vs EFV | 0.05 (0.00 to 7.12) | 0.00 to 8.16 |
| ZDV+3TC vs 3TC+d4T | 3.55 (0.27 to 142.50) | 0.20 to 178.30 |
| ZDV+3TC+ABC vs NoT/Plc | 0.46 (0.04 to 5.76) | 0.03 to 7.64 |
| ZDV+3TC+ABC vs ZDV | 0.55 (0.05 to 6.13) | 0.04 to 8.71 |
| ZDV+3TC+ABC vs d4T | 0.12 (0.00 to 4.19) | 0.00 to 5.22 |
| ZDV+3TC+ABC vs ddl | 0.56 (0.00 to 44.03) | 0.00 to 53.15 |
| ZDV+3TC+ABC vs d4T+ddI | 0.09 (0.00 to 3.10) | 0.00 to 3.97 |
| ZDV+3TC+ABC vs EFV | 0.02 (0.00 to 4.30) | 0.00 to 4.93 |
| ZDV+3TC+ABC vs 3TC+d4T | 1.33 (0.04 to 85.83) | 0.04 to 114.10 |
| ZDV+3TC+ABC vs ZDV+3TC | 0.36 (0.03 to 4.89) | 0.02 to 6.81 |
| ZDV+ddI+NVP vs NoT/Plc | 1.95 (0.17 to 19.69) | 0.12 to 26.81 |
| ZDV+ddI+NVP vs ZDV | 2.33 (0.22 to 20.76) | 0.16 to 28.87 |
| ZDV+ddI+NVP vs d4T | 0.51 (0.01 to 15.76) | 0.01 to 21.79 |
| ZDV+ddI+NVP vs ddl | 2.30 (0.02 to 169.70) | 0.02 to 203.70 |
| ZDV+ddI+NVP vs d4T+ddI | 0.38 (0.00 to 10.72) | 0.00 to 12.93 |
| ZDV+ddI+NVP vs EFV | 0.07 (0.00 to 13.38) | 0.00 to 14.55 |
| ZDV+ddI+NVP vs 3TC+d4T | 5.53 (0.30 to 251.00) | 0.25 to 298.10 |
| ZDV+ddI+NVP vs ZDV+3TC | 1.54 (0.12 to 17.46) | 0.09 to 23.65 |
| ZDV+ddI+NVP vs ZDV+3TC+ABC | 4.17 (0.15 to 111.80) | 0.12 to 144.10 |
| ddI+d4T+NVP vs NoT/Plc | 1.89 (0.16 to 16.66) | 0.12 to 23.07 |
| ddI+d4T+NVP vs ZDV | 2.28 (0.21 to 17.67) | 0.15 to 24.16 |
| ddI+d4T+NVP vs d4T | 0.50 (0.01 to 14.80) | 0.01 to 19.54 |
| ddI+d4T+NVP vs ddl | 2.19 (0.02 to 159.90) | 0.02 to 198.20 |
| ddI+d4T+NVP vs d4T+ddI | 0.37 (0.00 to 9.95) | 0.00 to 12.21 |
| ddI+d4T+NVP vs EFV | 0.07 (0.00 to 13.23) | 0.00 to 14.94 |
| ddI+d4T+NVP vs 3TC+d4T | 5.29 (0.30 to 215.60) | 0.23 to 274.80 |
| ddI+d4T+NVP vs ZDV+3TC | 1.48 (0.11 to 14.71) | 0.09 to 19.83 |
| ddI+d4T+NVP vs ZDV+3TC+ABC | 4.04 (0.14 to 94.21) | 0.11 to 126.10 |
| ddI+d4T+NVP vs ZDV+ddI+NVP | 0.97 (0.06 to 14.23) | 0.05 to 18.31 |
| ZDV+3TC+NVP vs NoT/Plc | 0.81 (0.06 to 10.52) | 0.04 to 13.49 |
| ZDV+3TC+NVP vs ZDV | 0.96 (0.08 to 10.37) | 0.06 to 14.32 |
| ZDV+3TC+NVP vs d4T | 0.21 (0.00 to 7.64) | 0.00 to 9.78 |
| ZDV+3TC+NVP vs ddl | 0.94 (0.01 to 78.62) | 0.01 to 96.99 |
| ZDV+3TC+NVP vs d4T+ddI | 0.16 (0.00 to 5.54) | 0.00 to 6.66 |
| ZDV+3TC+NVP vs EFV | 0.03 (0.00 to 8.43) | 0.00 to 9.53 |
| ZDV+3TC+NVP vs 3TC+d4T | 2.34 (0.07 to 159.20) | 0.06 to 204.20 |
| ZDV+3TC+NVP vs ZDV+3TC | 0.63 (0.04 to 8.32) | 0.03 to 11.52 |
| ZDV+3TC+NVP vs ZDV+3TC+ABC | 1.75 (0.28 to 10.49) | 0.19 to 15.17 |
| ZDV+3TC+NVP vs ZDV+ddI+NVP | 0.42 (0.01 to 13.19) | 0.01 to 16.57 |
| ZDV+3TC+NVP vs ddI+d4T+NVP | 0.43 (0.02 to 12.85) | 0.01 to 16.00 |
| LOP+RIT vs NoT/Plc | 0.22 (0.02 to 2.09) | 0.01 to 2.95 |
| LOP+RIT vs ZDV | 0.26 (0.02 to 2.13) | 0.02 to 3.20 |
| LOP+RIT vs d4T | 0.06 (0.00 to 1.74) | 0.00 to 2.08 |
| LOP+RIT vs ddl | 0.26 (0.00 to 19.29) | 0.00 to 23.07 |
| LOP+RIT vs d4T+ddI | 0.04 (0.00 to 1.19) | 0.00 to 1.41 |
| LOP+RIT vs EFV | 0.01 (0.00 to 2.03) | 0.00 to 2.30 |
| LOP+RIT vs 3TC+d4T | 0.63 (0.02 to 37.96) | 0.02 to 49.24 |
| LOP+RIT vs ZDV+3TC | 0.18 (0.01 to 1.73) | 0.01 to 2.43 |
| LOP+RIT vs ZDV+3TC+ABC | 0.48 (0.04 to 4.75) | 0.03 to 6.39 |
| LOP+RIT vs ZDV+ddI+NVP | 0.11 (0.00 to 2.99) | 0.00 to 3.66 |
| LOP+RIT vs ddI+d4T+NVP | 0.12 (0.00 to 2.72) | 0.00 to 3.26 |
| LOP+RIT vs ZDV+3TC+NVP | 0.27 (0.02 to 2.74) | 0.02 to 3.73 |
| ZDV+3TC+LOP+RIT vs NoT/Plc | 0.49 (0.08 to 2.69) | 0.06 to 4.18 |
| ZDV+3TC+LOP+RIT vs ZDV | 0.59 (0.12 to 2.66) | 0.07 to 3.96 |
| ZDV+3TC+LOP+RIT vs d4T | 0.13 (0.00 to 2.56) | 0.00 to 3.30 |
| ZDV+3TC+LOP+RIT vs ddl | 0.59 (0.01 to 32.01) | 0.01 to 37.24 |
| ZDV+3TC+LOP+RIT vs d4T+ddI | 0.10 (0.00 to 1.79) | 0.00 to 2.38 |
| ZDV+3TC+LOP+RIT vs EFV | 0.02 (0.00 to 3.25) | 0.00 to 3.62 |
| ZDV+3TC+LOP+RIT vs 3TC+d4T | 1.41 (0.08 to 66.64) | 0.06 to 82.46 |
| ZDV+3TC+LOP+RIT vs ZDV+3TC | 0.39 (0.06 to 2.38) | 0.04 to 3.45 |
| ZDV+3TC+LOP+RIT vs ZDV+3TC+ABC | 1.06 (0.16 to 6.42) | 0.11 to 9.25 |
| ZDV+3TC+LOP+RIT vs ZDV+ddI+NVP | 0.25 (0.02 to 4.26) | 0.01 to 5.40 |
| ZDV+3TC+LOP+RIT vs ddI+d4T+NVP | 0.26 (0.02 to 4.44) | 0.01 to 5.47 |
| ZDV+3TC+LOP+RIT vs ZDV+3TC+NVP | 0.60 (0.09 to 4.06) | 0.07 to 5.94 |
| ZDV+3TC+LOP+RIT vs LOP+RIT | 2.22 (0.50 to 12.02) | 0.31 to 19.14 |
| *Common within-network between-study variance* | 0.181 (0.00 to 1.83) |  |
| *Regression coefficient* | 1.03 (0.83 to 1.44) |  |
| *Design-by-treatment interaction model for inconsistency χ² (d.f., P-value, between-study variance)* | 1.59 (1, 0.21, 0.00) | |
| *Model fit measures and diagnostics* | Residual deviance = 32.94 Data points = 33  Effective number of parameters = 31.25 DIC = 64.19 | |
| ***Schmitz Model – All study designs*** | | |
| **Total Congenital Malformations** | | |
| ZDV vs NoT/Plc | 1.18 (0.48 to 3.27) | - |
| d4T vs NoT/Plc | 4.88 (0.35 to 249.90) | - |
| d4T vs ZDV | 4.17 (0.29 to 206.40) | - |
| ddl vs NoT/Plc | 1.18 (0.02 to 70.15) | - |
| ddl vs ZDV | 1.00 (0.02 to 57.09) | - |
| ddl vs d4T | 0.24 (0.01 to 3.08) | - |
| d4T+ddI vs NoT/Plc | 6.72 (0.38 to 392.50) | - |
| d4T+ddI vs ZDV | 5.68 (0.31 to 302.30) | - |
| d4T+ddI vs d4T | 1.33 (0.13 to 14.99) | - |
| d4T+ddI vs ddl | 5.66 (0.39 to 226.20) | - |
| EFV vs NoT/Plc | 29.12 (0.22 to 33130.00) | - |
| EFV vs ZDV | 24.65 (0.17 to 29030.00) | - |
| EFV vs d4T | 5.34 (0.07 to 3506.00) | - |
| EFV vs ddl | 23.85 (0.21 to 25500.00) | - |
| EFV vs d4T+ddI | 3.74 (0.08 to 1653.00) | - |
| 3TC+d4T vs NoT/Plc | 0.52 (0.01 to 6.80) | - |
| 3TC+d4T vs ZDV | 0.44 (0.01 to 5.63) | - |
| 3TC+d4T vs d4T | 0.10 (0.00 to 3.96) | - |
| 3TC+d4T vs ddl | 0.40 (0.00 to 45.05) | - |
| 3TC+d4T vs d4T+ddI | 0.07 (0.00 to 3.67) | - |
| 3TC+d4T vs EFV | 0.02 (0.00 to 4.14) | - |
| ZDV+3TC vs NoT/Plc | 1.26 (0.38 to 4.49) | - |
| ZDV+3TC vs ZDV | 1.08 (0.25 to 4.16) | - |
| ZDV+3TC vs d4T | 0.26 (0.00 to 4.66) | - |
| ZDV+3TC vs ddl | 1.07 (0.02 to 65.95) | - |
| ZDV+3TC vs d4T+ddI | 0.19 (0.00 to 4.08) | - |
| ZDV+3TC vs EFV | 0.04 (0.00 to 6.53) | - |
| ZDV+3TC vs 3TC+d4T | 2.47 (0.14 to 107.20) | - |
| ZDV+3TC+ABC vs NoT/Plc | 0.59 (0.08 to 4.57) | - |
| ZDV+3TC+ABC vs ZDV | 0.50 (0.06 to 4.05) | - |
| ZDV+3TC+ABC vs d4T | 0.12 (0.00 to 3.14) | - |
| ZDV+3TC+ABC vs ddl | 0.50 (0.01 to 38.60) | - |
| ZDV+3TC+ABC vs d4T+ddI | 0.09 (0.00 to 2.88) | - |
| ZDV+3TC+ABC vs EFV | 0.02 (0.00 to 3.48) | - |
| ZDV+3TC+ABC vs 3TC+d4T | 1.18 (0.05 to 66.45) | - |
| ZDV+3TC+ABC vs ZDV+3TC | 0.47 (0.04 to 4.59) | - |
| ZDV+3TC+NFV vs NoT/Plc | 0.31 (0.00 to 8.25) | - |
| ZDV+3TC+NFV vs ZDV | 0.26 (0.00 to 7.40) | - |
| ZDV+3TC+NFV vs d4T | 0.05 (0.00 to 4.31) | - |
| ZDV+3TC+NFV vs ddl | 0.22 (0.00 to 39.39) | - |
| ZDV+3TC+NFV vs d4T+ddI | 0.04 (0.00 to 3.24) | - |
| ZDV+3TC+NFV vs EFV | 0.01 (0.00 to 3.64) | - |
| ZDV+3TC+NFV vs 3TC+d4T | 0.58 (0.00 to 76.60) | - |
| ZDV+3TC+NFV vs ZDV+3TC | 0.23 (0.00 to 7.94) | - |
| ZDV+3TC+NFV vs ZDV+3TC+ABC | 0.50 (0.00 to 22.14) | - |
| NVP vs NoT/Plc | 18.21 (0.04 to 28570.00) | - |
| NVP vs ZDV | 15.30 (0.03 to 23030.00) | - |
| NVP vs d4T | 3.19 (0.01 to 2824.00) | - |
| NVP vs ddl | 14.05 (0.04 to 21290.00) | - |
| NVP vs d4T+ddI | 2.26 (0.01 to 1539.00) | - |
| NVP vs EFV | 0.65 (0.02 to 7.46) | - |
| NVP vs 3TC+d4T | 39.32 (0.05 to 124900.00) | - |
| NVP vs ZDV+3TC | 14.51 (0.03 to 23150.00) | - |
| NVP vs ZDV+3TC+ABC | 31.92 (0.05 to 61030.00) | - |
| NVP vs ZDV+3TC+NFV | 85.04 (0.06 to 744200.00) | - |
| ZDV+ddI+NVP vs NoT/Plc | 2.73 (0.19 to 26.46) | - |
| ZDV+ddI+NVP vs ZDV | 2.26 (0.15 to 24.20) | - |
| ZDV+ddI+NVP vs d4T | 0.52 (0.01 to 18.57) | - |
| ZDV+ddI+NVP vs ddl | 2.22 (0.02 to 181.80) | - |
| ZDV+ddI+NVP vs d4T+ddI | 0.38 (0.00 to 16.49) | - |
| ZDV+ddI+NVP vs EFV | 0.09 (0.00 to 19.04) | - |
| ZDV+ddI+NVP vs 3TC+d4T | 5.16 (0.27 to 215.70) | - |
| ZDV+ddI+NVP vs ZDV+3TC | 2.10 (0.14 to 26.92) | - |
| ZDV+ddI+NVP vs ZDV+3TC+ABC | 4.51 (0.19 to 94.47) | - |
| ZDV+ddI+NVP vs ZDV+3TC+NFV | 9.10 (0.14 to 5066.00) | - |
| ZDV+ddI+NVP vs NVP | 0.14 (0.00 to 97.84) | - |
| ddI+d4T+NVP vs NoT/Plc | 2.86 (0.22 to 28.29) | - |
| ddI+d4T+NVP vs ZDV | 2.36 (0.18 to 22.99) | - |
| ddI+d4T+NVP vs d4T | 0.55 (0.01 to 18.66) | - |
| ddI+d4T+NVP vs ddl | 2.40 (0.02 to 204.10) | - |
| ddI+d4T+NVP vs d4T+ddI | 0.39 (0.00 to 15.74) | - |
| ddI+d4T+NVP vs EFV | 0.09 (0.00 to 21.58) | - |
| ddI+d4T+NVP vs 3TC+d4T | 5.63 (0.28 to 228.10) | - |
| ddI+d4T+NVP vs ZDV+3TC | 2.19 (0.15 to 27.92) | - |
| ddI+d4T+NVP vs ZDV+3TC+ABC | 4.81 (0.21 to 94.69) | - |
| ddI+d4T+NVP vs ZDV+3TC+NFV | 9.89 (0.15 to 7919.00) | - |
| ddI+d4T+NVP vs NVP | 0.15 (0.00 to 111.90) | - |
| ddI+d4T+NVP vs ZDV+ddI+NVP | 1.06 (0.07 to 17.53) | - |
| ZDV+3TC+NVP vs NoT/Plc | 1.12 (0.23 to 5.58) | - |
| ZDV+3TC+NVP vs ZDV | 0.94 (0.17 to 5.12) | - |
| ZDV+3TC+NVP vs d4T | 0.22 (0.00 to 4.53) | - |
| ZDV+3TC+NVP vs ddl | 0.90 (0.01 to 64.14) | - |
| ZDV+3TC+NVP vs d4T+ddI | 0.16 (0.00 to 4.02) | - |
| ZDV+3TC+NVP vs EFV | 0.04 (0.00 to 6.44) | - |
| ZDV+3TC+NVP vs 3TC+d4T | 2.16 (0.12 to 99.94) | - |
| ZDV+3TC+NVP vs ZDV+3TC | 0.89 (0.13 to 6.07) | - |
| ZDV+3TC+NVP vs ZDV+3TC+ABC | 1.89 (0.28 to 14.01) | - |
| ZDV+3TC+NVP vs ZDV+3TC+NFV | 3.66 (0.11 to 2002.00) | - |
| ZDV+3TC+NVP vs NVP | 0.06 (0.00 to 34.53) | - |
| ZDV+3TC+NVP vs ZDV+ddI+NVP | 0.42 (0.03 to 7.75) | - |
| ZDV+3TC+NVP vs ddI+d4T+NVP | 0.39 (0.03 to 6.86) | - |
| LOP+RIT vs NoT/Plc | 0.31 (0.04 to 2.80) | - |
| LOP+RIT vs ZDV | 0.26 (0.03 to 2.40) | - |
| LOP+RIT vs d4T | 0.06 (0.00 to 1.59) | - |
| LOP+RIT vs ddl | 0.26 (0.00 to 20.63) | - |
| LOP+RIT vs d4T+ddI | 0.05 (0.00 to 1.32) | - |
| LOP+RIT vs EFV | 0.01 (0.00 to 1.85) | - |
| LOP+RIT vs 3TC+d4T | 0.61 (0.02 to 36.63) | - |
| LOP+RIT vs ZDV+3TC | 0.24 (0.02 to 2.59) | - |
| LOP+RIT vs ZDV+3TC+ABC | 0.51 (0.04 to 5.78) | - |
| LOP+RIT vs ZDV+3TC+NFV | 1.05 (0.02 to 711.40) | - |
| LOP+RIT vs NVP | 0.02 (0.00 to 10.54) | - |
| LOP+RIT vs ZDV+ddI+NVP | 0.12 (0.01 to 3.03) | - |
| LOP+RIT vs ddI+d4T+NVP | 0.11 (0.01 to 2.79) | - |
| LOP+RIT vs ZDV+3TC+NVP | 0.27 (0.03 to 2.60) | - |
| ZDV+3TC+LOP+RIT vs NoT/Plc | 0.65 (0.16 to 3.07) | - |
| ZDV+3TC+LOP+RIT vs ZDV | 0.56 (0.11 to 2.47) | - |
| ZDV+3TC+LOP+RIT vs d4T | 0.13 (0.00 to 2.40) | - |
| ZDV+3TC+LOP+RIT vs ddl | 0.56 (0.01 to 33.71) | - |
| ZDV+3TC+LOP+RIT vs d4T+ddI | 0.10 (0.00 to 2.10) | - |
| ZDV+3TC+LOP+RIT vs EFV | 0.02 (0.00 to 3.16) | - |
| ZDV+3TC+LOP+RIT vs 3TC+d4T | 1.28 (0.08 to 55.66) | - |
| ZDV+3TC+LOP+RIT vs ZDV+3TC | 0.52 (0.08 to 3.12) | - |
| ZDV+3TC+LOP+RIT vs ZDV+3TC+ABC | 1.10 (0.19 to 6.33) | - |
| ZDV+3TC+LOP+RIT vs ZDV+3TC+NFV | 2.19 (0.06 to 1241.00) | - |
| ZDV+3TC+LOP+RIT vs NVP | 0.04 (0.00 to 16.94) | - |
| ZDV+3TC+LOP+RIT vs ZDV+ddI+NVP | 0.25 (0.02 to 4.52) | - |
| ZDV+3TC+LOP+RIT vs ddI+d4T+NVP | 0.24 (0.02 to 3.65) | - |
| ZDV+3TC+LOP+RIT vs ZDV+3TC+NVP | 0.59 (0.11 to 2.94) | - |
| ZDV+3TC+LOP+RIT vs LOP+RIT | 2.13 (0.44 to 10.54) | - |
| *Common within-network between-study variance* | 0.09 (0.00 to 1.82) |  |
| **Major Congenital Malformations** | | |
| ZDV vs NoT/PLC | 0.64 (0.12 to 3.04) | - |
| 3TC+d4T vs NoT/PLC | 0.29 (0.01 to 4.65) | - |
| 3TC+d4T vs ZDV | 0.46 (0.01 to 8.80) | - |
| ZDV+3TCvs NoT/PLC | 1.03 (0.13 to 4.66) | - |
| ZDV+3TCvs ZDV | 1.55 (0.17 to 11.17) | - |
| ZDV+3TCvs 3TC+d4T | 3.41 (0.15 to 135.30) | - |
| ZDV+3TC+NFV vs NoT/PLC | 0.32 (0.00 to 9.91) | - |
| ZDV+3TC+NFV vs ZDV | 0.49 (0.00 to 19.78) | - |
| ZDV+3TC+NFV vs 3TC+d4T | 1.09 (0.00 to 139.80) | - |
| ZDV+3TC+NFV vsZDV+3TC | 0.33 (0.00 to 15.40) | - |
| ZDV+ddI+NVP vs NoT/PLC | 1.69 (0.10 to 21.24) | - |
| ZDV+ddI+NVP vs ZDV | 2.64 (0.14 to 40.51) | - |
| ZDV+ddI+NVP vs 3TC+d4T | 5.66 (0.27 to 238.00) | - |
| ZDV+ddI+NVP vsZDV+3TC | 1.68 (0.10 to 33.95) | - |
| ZDV+ddI+NVP vs ZDV+3TC+NFV | 5.48 (0.07 to 4409.00) | - |
| ddI+d4T+NVP vs NoT/PLC | 1.68 (0.10 to 21.07) | - |
| ddI+d4T+NVP vs ZDV | 2.57 (0.14 to 36.44) | - |
| ddI+d4T+NVP vs 3TC+d4T | 5.66 (0.25 to 243.30) | - |
| ddI+d4T+NVP vsZDV+3TC | 1.63 (0.10 to 32.48) | - |
| ddI+d4T+NVP vs ZDV+3TC+NFV | 5.44 (0.07 to 3710.00) | - |
| ddI+d4T+NVP vs ZDV+ddI+NVP | 0.99 (0.05 to 16.48) | - |
| ZDV+3TC+NVP vs NoT/PLC | 0.33 (0.00 to 10.67) | - |
| ZDV+3TC+NVP vs ZDV | 0.50 (0.00 to 21.90) | - |
| ZDV+3TC+NVP vs 3TC+d4T | 1.12 (0.00 to 145.40) | - |
| ZDV+3TC+NVP vsZDV+3TC | 0.33 (0.00 to 16.18) | - |
| ZDV+3TC+NVP vs ZDV+3TC+NFV | 1.00 (0.00 to 896.50) | - |
| ZDV+3TC+NVP vs ZDV+ddI+NVP | 0.19 (0.00 to 13.93) | - |
| ZDV+3TC+NVP vs ddI+d4T+NVP | 0.19 (0.00 to 16.26) | - |
| *Common within-network between-study variance* | 0.30 (0.00 to 3.80) |  |
| **Mother-to-child transmission of HIV** | | |
| [NoT]+[ZDV] vs [NoT/PLC]+[NoT/PLC] | 0.61 (0.14 to 2.63) | - |
| [ZDV]+[NoT] vs [NoT/PLC]+[NoT/PLC] | **0.35 (0.16 to 0.67)** | - |
| [ZDV]+[NoT] vs [NoT]+[ZDV] | 0.57 (0.14 to 2.40) | - |
| [ZDV]+[ZDV] vs [NoT/PLC]+[NoT/PLC] | 0.33 (0.07 to 1.62) | - |
| [ZDV]+[ZDV] vs [NoT]+[ZDV] | 0.53 (0.08 to 4.27) | - |
| [ZDV]+[ZDV] vs [ZDV]+[NoT] | 0.94 (0.19 to 5.44) | - |
| [NoT]+[NVP] vs [NoT/PLC]+[NoT/PLC] | **0.43 (0.20 to 0.94)** | - |
| [NoT]+[NVP] vs [NoT]+[ZDV] | 0.71 (0.14 to 3.77) | - |
| [NoT]+[NVP] vs [ZDV]+[NoT] | 1.24 (0.46 to 3.84) | - |
| [NoT]+[NVP] vs [ZDV]+[ZDV] | 1.33 (0.23 to 7.72) | - |
| [ZDV]+[NVP] vs [NoT/PLC]+[NoT/PLC] | **0.35 (0.14 to 0.89)** | - |
| [ZDV]+[NVP] vs [NoT]+[ZDV] | 0.58 (0.10 to 3.36) | - |
| [ZDV]+[NVP] vs [ZDV]+[NoT] | 1.01 (0.33 to 3.51) | - |
| [ZDV]+[NVP] vs [ZDV]+[ZDV] | 1.08 (0.18 to 6.72) | - |
| [ZDV]+[NVP] vs [NoT]+[NVP] | 0.82 (0.32 to 2.04) | - |
| *Common within-network between-study variance* | 0.58 (0.00 to 4.57) |  |
| ***Schmitz Model - Observational Studies*** | | |
| **Total Congenital Malformations** | | |
| ZDV vs NoT/Plc | 1.26 (0.65 to 2.76) | - |
| ZDV vs d4T+ddI | 0.18 (0.00 to 4.24) | - |
| ZDV vs EFV | 0.04 (0.00 to 5.97) | - |
| ZDV vs 3TC+d4T | 2.43 (0.22 to 89.26) | - |
| ZDV vs ZDV+3TC | 0.91 (0.39 to 2.93) | - |
| ZDV vs ZDV+3TC+NFV | 4.09 (0.16 to 2241.00) | - |
| ZDV vs NVP | 0.07 (0.00 to 31.35) | - |
| ZDV vs ZDV+ddI+NVP | 0.46 (0.05 to 5.98) | - |
| ZDV vs ddI+d4T+NVP | 0.45 (0.05 to 5.09) | - |
| d4T+ddI vs NoT/Plc | 6.94 (0.33 to 440.30) | - |
| d4T+ddI vs ZDV | 5.58 (0.24 to 320.70) | - |
| d4T+ddI vs EFV | 0.27 (0.00 to 10.12) | - |
| d4T+ddI vs 3TC+d4T | 14.93 (0.24 to 2646.00) | - |
| d4T+ddI vs ZDV+3TC | 5.18 (0.20 to 342.70) | - |
| d4T+ddI vs ZDV+3TC+NFV | 27.45 (0.27 to 40710.00) | - |
| d4T+ddI vs NVP | 0.44 (0.00 to 70.20) | - |
| d4T+ddI vs ZDV+ddI+NVP | 2.73 (0.05 to 322.70) | - |
| d4T+ddI vs ddI+d4T+NVP | 2.56 (0.05 to 283.80) | - |
| EFV vs NoT/Plc | 29.12 (0.22 to 33130.00) | - |
| EFV vs ZDV | 22.79 (0.17 to 22990.00) | - |
| EFV vs d4T+ddI | 3.66 (0.10 to 1448.00) | - |
| EFV vs 3TC+d4T | 63.93 (0.24 to 136600.00) | - |
| EFV vs ZDV+3TC | 21.69 (0.16 to 25170.00) | - |
| EFV vs ZDV+3TC+NFV | 140.60 (0.28 to 1064000.00) | - |
| EFV vs NVP | 1.54 (0.13 to 57.24) | - |
| EFV vs ZDV+ddI+NVP | 11.58 (0.05 to 17400.00) | - |
| EFV vs ddI+d4T+NVP | 11.14 (0.05 to 15470.00) | - |
| 3TC+d4T vs NoT/Plc | 0.52 (0.01 to 6.80) | - |
| 3TC+d4T vs ZDV | 0.41 (0.01 to 4.51) | - |
| 3TC+d4T vs d4T+ddI | 0.07 (0.00 to 4.15) | - |
| 3TC+d4T vs EFV | 0.02 (0.00 to 4.14) | - |
| 3TC+d4T vs ZDV+3TC | 0.38 (0.01 to 6.15) | - |
| 3TC+d4T vs ZDV+3TC+NFV | 1.72 (0.01 to 1264.00) | - |
| 3TC+d4T vs NVP | 0.03 (0.00 to 20.19) | - |
| 3TC+d4T vs ZDV+ddI+NVP | 0.19 (0.00 to 3.72) | - |
| 3TC+d4T vs ddI+d4T+NVP | 0.18 (0.00 to 3.64) | - |
| ZDV+3TC vs NoT/Plc | 1.34 (0.43 to 3.99) | - |
| ZDV+3TC vs ZDV | 1.11 (0.34 to 2.59) | - |
| ZDV+3TC vs d4T+ddI | 0.19 (0.00 to 5.08) | - |
| ZDV+3TC vs EFV | 0.05 (0.00 to 6.38) | - |
| ZDV+3TC vs 3TC+d4T | 2.62 (0.16 to 108.20) | - |
| ZDV+3TC vs ZDV+3TC+NFV | 4.48 (0.14 to 2224.00) | - |
| ZDV+3TC vs NVP | 0.07 (0.00 to 35.33) | - |
| ZDV+3TC vs ZDV+ddI+NVP | 0.51 (0.04 to 7.27) | - |
| ZDV+3TC vs ddI+d4T+NVP | 0.48 (0.04 to 6.21) | - |
| ZDV+3TC+NFV vs NoT/Plc | 0.31 (0.00 to 8.25) | - |
| ZDV+3TC+NFV vs ZDV | 0.24 (0.00 to 6.24) | - |
| ZDV+3TC+NFV vs d4T+ddI | 0.04 (0.00 to 3.79) | - |
| ZDV+3TC+NFV vs EFV | 0.01 (0.00 to 3.64) | - |
| ZDV+3TC+NFV vs 3TC+d4T | 0.58 (0.00 to 76.60) | - |
| ZDV+3TC+NFV vs ZDV+3TC | 0.22 (0.00 to 7.21) | - |
| ZDV+3TC+NFV vs NVP | 0.01 (0.00 to 16.81) | - |
| ZDV+3TC+NFV vs ZDV+ddI+NVP | 0.11 (0.00 to 7.40) | - |
| ZDV+3TC+NFV vs ddI+d4T+NVP | 0.10 (0.00 to 6.78) | - |
| NVP vs NoT/Plc | 18.21 (0.04 to 28570.00) | - |
| NVP vs ZDV | 14.04 (0.03 to 21610.00) | - |
| NVP vs d4T+ddI | 2.28 (0.01 to 1452.00) | - |
| NVP vs EFV | 0.65 (0.02 to 7.46) | - |
| NVP vs 3TC+d4T | 39.32 (0.05 to 124900.00) | - |
| NVP vs ZDV+3TC | 13.62 (0.03 to 21600.00) | - |
| NVP vs ZDV+3TC+NFV | 85.04 (0.06 to 744200.00) | - |
| NVP vs ZDV+ddI+NVP | 7.22 (0.01 to 15580.00) | - |
| NVP vs ddI+d4T+NVP | 6.67 (0.01 to 15520.00) | - |
| ZDV+ddI+NVP vs NoT/Plc | 2.73 (0.19 to 26.46) | - |
| ZDV+ddI+NVP vs ZDV | 2.16 (0.17 to 18.57) | - |
| ZDV+ddI+NVP vs d4T+ddI | 0.37 (0.00 to 19.31) | - |
| ZDV+ddI+NVP vs EFV | 0.09 (0.00 to 19.04) | - |
| ZDV+ddI+NVP vs 3TC+d4T | 5.16 (0.27 to 215.70) | - |
| ZDV+ddI+NVP vs ZDV+3TC | 1.97 (0.14 to 25.04) | - |
| ZDV+ddI+NVP vs ZDV+3TC+NFV | 9.10 (0.14 to 5066.00) | - |
| ZDV+ddI+NVP vs NVP | 0.14 (0.00 to 97.84) | - |
| ZDV+ddI+NVP vs ddI+d4T+NVP | 0.95 (0.06 to 14.86) | - |
| ddI+d4T+NVP vs NoT/Plc | 2.86 (0.22 to 28.29) | - |
| ddI+d4T+NVP vs ZDV | 2.25 (0.20 to 19.05) | - |
| ddI+d4T+NVP vs d4T+ddI | 0.39 (0.00 to 18.29) | - |
| ddI+d4T+NVP vs EFV | 0.09 (0.00 to 21.58) | - |
| ddI+d4T+NVP vs 3TC+d4T | 5.63 (0.28 to 228.10) | - |
| ddI+d4T+NVP vs ZDV+3TC | 2.07 (0.16 to 25.04) | - |
| ddI+d4T+NVP vs ZDV+3TC+NFV | 9.89 (0.15 to 7919.00) | - |
| ddI+d4T+NVP vs NVP | 0.15 (0.00 to 111.90) | - |
| ddI+d4T+NVP vs ZDV+ddI+NVP | 1.06 (0.07 to 17.53) | - |
| ZDV+3TC+NVP vs NoT/Plc | 1.12 (0.27 to 4.77) | - |
| ZDV+3TC+NVP vs ZDV | 0.90 (0.22 to 3.23) | - |
| ZDV+3TC+NVP vs d4T+ddI | 0.16 (0.00 to 4.86) | - |
| ZDV+3TC+NVP vs EFV | 0.04 (0.00 to 6.05) | - |
| ZDV+3TC+NVP vs 3TC+d4T | 2.17 (0.14 to 96.57) | - |
| ZDV+3TC+NVP vs ZDV+3TC | 0.85 (0.17 to 4.39) | - |
| ZDV+3TC+NVP vs ZDV+3TC+NFV | 3.74 (0.12 to 2108.00) | - |
| ZDV+3TC+NVP vs NVP | 0.06 (0.00 to 32.16) | - |
| ZDV+3TC+NVP vs ZDV+ddI+NVP | 0.43 (0.03 to 7.40) | - |
| ZDV+3TC+NVP vs ddI+d4T+NVP | 0.40 (0.03 to 6.47) | - |
| *Common within-network between-study variance* | 0.25 (0.00 to 1.88) |  |
| **Major Congenital Malformations** | | |
| ZDV vs NoT/PLC | 0.74 (0.24 to 2.22) | - |
| 3TC+d4T vs NoT/PLC | 0.29 (0.01 to 4.65) | - |
| 3TC+d4T vs ZDV | 0.40 (0.01 to 5.16) | - |
| ZDV+3TCvs NoT/PLC | 1.03 (0.13 to 4.66) | - |
| ZDV+3TCvs ZDV | 1.41 (0.23 to 4.44) | - |
| ZDV+3TCvs 3TC+d4T | 3.41 (0.15 to 135.30) | - |
| ZDV+3TC+NFV vs NoT/PLC | 0.32 (0.00 to 9.91) | - |
| ZDV+3TC+NFV vs ZDV | 0.43 (0.00 to 13.80) | - |
| ZDV+3TC+NFV vs 3TC+d4T | 1.09 (0.00 to 139.80) | - |
| ZDV+3TC+NFV vsZDV+3TC | 0.33 (0.00 to 15.40) | - |
| ZDV+ddI+NVP vs NoT/PLC | 1.69 (0.10 to 21.24) | - |
| ZDV+ddI+NVP vs ZDV | 2.28 (0.17 to 22.14) | - |
| ZDV+ddI+NVP vs 3TC+d4T | 5.66 (0.27 to 238.00) | - |
| ZDV+ddI+NVP vsZDV+3TC | 1.68 (0.10 to 33.95) | - |
| ZDV+ddI+NVP vs ZDV+3TC+NFV | 5.48 (0.07 to 4409.00) | - |
| ddI+d4T+NVP vs NoT/PLC | 1.68 (0.10 to 21.07) | - |
| ddI+d4T+NVP vs ZDV | 2.27 (0.17 to 21.98) | - |
| ddI+d4T+NVP vs 3TC+d4T | 5.66 (0.25 to 243.30) | - |
| ddI+d4T+NVP vsZDV+3TC | 1.63 (0.10 to 32.48) | - |
| ddI+d4T+NVP vs ZDV+3TC+NFV | 5.44 (0.07 to 3710.00) | - |
| ddI+d4T+NVP vs ZDV+ddI+NVP | 0.99 (0.05 to 16.48) | - |
| ZDV+3TC+NVP vs NoT/PLC | 0.33 (0.00 to 10.67) | - |
| ZDV+3TC+NVP vs ZDV | 0.45 (0.00 to 14.21) | - |
| ZDV+3TC+NVP vs 3TC+d4T | 1.12 (0.00 to 145.40) | - |
| ZDV+3TC+NVP vsZDV+3TC | 0.33 (0.00 to 16.18) | - |
| ZDV+3TC+NVP vs ZDV+3TC+NFV | 1.00 (0.00 to 896.50) | - |
| ZDV+3TC+NVP vs ZDV+ddI+NVP | 0.19 (0.00 to 13.93) | - |
| ZDV+3TC+NVP vs ddI+d4T+NVP | 0.19 (0.00 to 16.26) | - |
| *Common within-network between-study variance* | 0.27 (0.00 to 2.91) |  |
| **Mother-to-child transmission of HIV** | | |
| [NoT]+[ZDV] vs [NoT/PLC]+[NoT/PLC] | 0.61 (0.14 to 2.63) | - |
| [ZDV]+[NoT] vs [NoT/PLC]+[NoT/PLC] | **0.35 (0.16 to 0.67)** | - |
| [ZDV]+[NoT] vs [NoT]+[ZDV] | 0.57 (0.14 to 2.40) | - |
| [ZDV]+[ZDV] vs [NoT/PLC]+[NoT/PLC] | **0.22 (0.08 to 0.52)** | - |
| [ZDV]+[ZDV] vs [NoT]+[ZDV] | 0.35 (0.09 to 1.43) | - |
| [ZDV]+[ZDV] vs [ZDV]+[NoT] | 0.63 (0.23 to 1.61) | - |
| [NoT]+[NVP] vs [NoT/PLC]+[NoT/PLC] | **0.43 (0.20 to 0.94)** | - |
| [NoT]+[NVP] vs [NoT]+[ZDV] | 0.71 (0.14 to 3.77) | - |
| [NoT]+[NVP] vs [ZDV]+[NoT] | 1.24 (0.46 to 3.84) | - |
| [NoT]+[NVP] vs [ZDV]+[ZDV] | 1.99 (0.64 to 7.09) | - |
| [ZDV]+[NVP] vs [NoT/PLC]+[NoT/PLC] | **0.35 (0.14 to 0.88)** | - |
| [ZDV]+[NVP] vs [NoT]+[ZDV] | 0.58 (0.10 to 3.36) | - |
| [ZDV]+[NVP] vs [ZDV]+[NoT] | 1.01 (0.33 to 3.51) | - |
| [ZDV]+[NVP] vs [ZDV]+[ZDV] | 1.63 (0.47 to 6.16) | - |
| [ZDV]+[NVP] vs [NoT]+[NVP] | 0.82 (0.32 to 2.04) | - |
| *Common within-network between-study variance* | 0.07 (0.00 to 1.23) |  |
| ***Schmitz Model – Randomised Control Trials*** | | |
| **Total Congenital Malformations** | | |
| ZDV vs NoT/Plc | 1.10 (0.60 to 2.44) | - |
| ZDV vs d4T | 0.23 (0.01 to 2.92) | - |
| ZDV vs ddl | 0.93 (0.02 to 50.31) | - |
| ZDV vs d4T+ddI | 0.16 (0.00 to 2.20) | - |
| ZDV vs ZDV+3TC | 0.92 (0.32 to 3.04) | - |
| ZDV vs ZDV+3TC+ABC | 1.87 (0.26 to 15.16) | - |
| ZDV vs ZDV+3TC+NVP | 1.02 (0.22 to 4.81) | - |
| ZDV vs LOP+RIT | 3.67 (0.46 to 30.76) | - |
| d4T vs NoT/Plc | 4.88 (0.35 to 249.90) | - |
| d4T vs ZDV | 4.35 (0.34 to 197.30) | - |
| d4T vs ddl | 4.12 (0.33 to 140.00) | - |
| d4T vs d4T+ddI | 0.75 (0.09 to 5.75) | - |
| d4T vs ZDV+3TC | 4.16 (0.25 to 218.40) | - |
| d4T vs ZDV+3TC+ABC | 8.62 (0.32 to 646.30) | - |
| d4T vs ZDV+3TC+NVP | 4.67 (0.23 to 266.00) | - |
| d4T vs LOP+RIT | 16.09 (0.63 to 1268.00) | - |
| ddl vs NoT/Plc | 1.18 (0.02 to 70.15) | - |
| ddl vs ZDV | 1.07 (0.02 to 54.12) | - |
| ddl vs d4T | 0.24 (0.01 to 3.08) | - |
| ddl vs d4T+ddI | 0.18 (0.01 to 2.04) | - |
| ddl vs ZDV+3TC | 1.00 (0.02 to 65.65) | - |
| ddl vs ZDV+3TC+ABC | 1.99 (0.03 to 169.10) | - |
| ddl vs ZDV+3TC+NVP | 1.12 (0.02 to 75.96) | - |
| ddl vs LOP+RIT | 3.89 (0.05 to 348.80) | - |
| d4T+ddI vs NoT/Plc | 6.62 (0.51 to 368.30) | - |
| d4T+ddI vs ZDV | 6.07 (0.46 to 268.70) | - |
| d4T+ddI vs d4T | 1.33 (0.18 to 11.24) | - |
| d4T+ddI vs ddl | 5.56 (0.49 to 199.80) | - |
| d4T+ddI vs ZDV+3TC | 5.56 (0.35 to 293.20) | - |
| d4T+ddI vs ZDV+3TC+ABC | 11.46 (0.44 to 793.70) | - |
| d4T+ddI vs ZDV+3TC+NVP | 6.34 (0.31 to 358.00) | - |
| d4T+ddI vs LOP+RIT | 21.67 (0.88 to 1618.00) | - |
| ZDV+3TC vs NoT/Plc | 1.17 (0.45 to 3.52) | - |
| ZDV+3TC vs ZDV | 1.08 (0.33 to 3.13) | - |
| ZDV+3TC vs d4T | 0.24 (0.00 to 3.98) | - |
| ZDV+3TC vs ddl | 1.00 (0.02 to 61.52) | - |
| ZDV+3TC vs d4T+ddI | 0.18 (0.00 to 2.85) | - |
| ZDV+3TC vs ZDV+3TC+ABC | 1.98 (0.22 to 19.30) | - |
| ZDV+3TC vs ZDV+3TC+NVP | 1.08 (0.18 to 6.18) | - |
| ZDV+3TC vs LOP+RIT | 3.88 (0.39 to 40.11) | - |
| ZDV+3TC+ABC vs NoT/Plc | 0.59 (0.08 to 4.57) | - |
| ZDV+3TC+ABC vs ZDV | 0.54 (0.07 to 3.81) | - |
| ZDV+3TC+ABC vs d4T | 0.12 (0.00 to 3.14) | - |
| ZDV+3TC+ABC vs ddl | 0.50 (0.01 to 38.60) | - |
| ZDV+3TC+ABC vs d4T+ddI | 0.09 (0.00 to 2.28) | - |
| ZDV+3TC+ABC vs ZDV+3TC | 0.51 (0.05 to 4.50) | - |
| ZDV+3TC+ABC vs ZDV+3TC+NVP | 0.55 (0.09 to 3.20) | - |
| ZDV+3TC+ABC vs LOP+RIT | 1.96 (0.17 to 22.28) | - |
| ZDV+3TC+NVP vs NoT/Plc | 1.08 (0.24 to 5.49) | - |
| ZDV+3TC+NVP vs ZDV | 0.98 (0.21 to 4.47) | - |
| ZDV+3TC+NVP vs d4T | 0.21 (0.00 to 4.36) | - |
| ZDV+3TC+NVP vs ddl | 0.89 (0.01 to 61.38) | - |
| ZDV+3TC+NVP vs d4T+ddI | 0.16 (0.00 to 3.20) | - |
| ZDV+3TC+NVP vs ZDV+3TC | 0.93 (0.16 to 5.50) | - |
| ZDV+3TC+NVP vs ZDV+3TC+ABC | 1.84 (0.31 to 11.14) | - |
| ZDV+3TC+NVP vs LOP+RIT | 3.63 (0.40 to 30.52) | - |
| LOP+RIT vs NoT/Plc | 0.31 (0.04 to 2.80) | - |
| LOP+RIT vs ZDV | 0.27 (0.03 to 2.18) | - |
| LOP+RIT vs d4T | 0.06 (0.00 to 1.59) | - |
| LOP+RIT vs ddl | 0.26 (0.00 to 20.63) | - |
| LOP+RIT vs d4T+ddI | 0.05 (0.00 to 1.14) | - |
| LOP+RIT vs ZDV+3TC | 0.26 (0.02 to 2.58) | - |
| LOP+RIT vs ZDV+3TC+ABC | 0.51 (0.04 to 5.78) | - |
| LOP+RIT vs ZDV+3TC+NVP | 0.28 (0.03 to 2.48) | - |
| ZDV+3TC+LOP+RIT vs NoT/Plc | 0.65 (0.16 to 3.07) | - |
| ZDV+3TC+LOP+RIT vs ZDV | 0.60 (0.14 to 2.14) | - |
| ZDV+3TC+LOP+RIT vs d4T | 0.13 (0.00 to 2.40) | - |
| ZDV+3TC+LOP+RIT vs ddl | 0.56 (0.01 to 33.71) | - |
| ZDV+3TC+LOP+RIT vs d4T+ddI | 0.10 (0.00 to 1.70) | - |
| ZDV+3TC+LOP+RIT vs ZDV+3TC | 0.56 (0.10 to 2.93) | - |
| ZDV+3TC+LOP+RIT vs ZDV+3TC+ABC | 1.10 (0.19 to 6.33) | - |
| ZDV+3TC+LOP+RIT vs ZDV+3TC+NVP | 0.60 (0.14 to 2.58) | - |
| ZDV+3TC+LOP+RIT vs LOP+RIT | 2.13 (0.44 to 10.54) | - |
| *Common within-network between-study variance* | 0.19 (0.00 to 2.05) |  |
| **Major Congenital Malformations** | | |
| ZDV vs NoT/PLC | 0.56 (0.14 to 1.96) | - |
| *Common within-network between-study variance* | 1.65 (0.45 to 5.79) |  |
| **Mother-to-child transmission of HIV** | | |
| [ZDV]+[ZDV] vs [NoT/PLC]+[NoT/PLC] | 0.52 (0.17 to 1.23) | - |
| *Common within-network between-study variance* | 0.41 (0.02 to 3.17) |  |
| *Design-by-treatment interaction model for inconsistency χ² (d.f., P-value, between-study variance)* | Not estimable (only 1 RCT available) | |
| ***Sensitivity Analysis: Higher Methodological Study Quality - Comparability for Observational Studies and Randomisation for Randomised Controlled Trials*** | | |
| **Total Congenital Malformations** | | |
| ZDV vs NoT/Plc | 1.02 (0.48 to 2.69) | 0.20 to 6.32 |
| 3TC+d4T vs NoT/Plc | 0.41 (0.01 to 6.47) | 0.01 to 9.92 |
| 3TC+d4T vs ZDV | 0.41 (0.01 to 4.81) | 0.01 to 7.02 |
| ZDV+3TC vs NoT/Plc | 1.38 (0.48 to 5.18) | 0.23 to 10.10 |
| ZDV+3TC vs ZDV | 1.38 (0.40 to 4.47) | 0.19 to 8.76 |
| ZDV+3TC vs 3TC+d4T | 3.43 (0.21 to 145.70) | 0.14 to 181.90 |
| ZDV+ddI+NVP vs NoT/Plc | 2.26 (0.18 to 25.92) | 0.13 to 39.03 |
| ZDV+ddI+NVP vs ZDV | 2.18 (0.19 to 20.23) | 0.13 to 30.41 |
| ZDV+ddI+NVP vs 3TC+d4T | 5.48 (0.26 to 233.20) | 0.19 to 289.40 |
| ZDV+ddI+NVP vs ZDV+3TC | 1.59 (0.11 to 19.37) | 0.07 to 27.84 |
| ddI+d4T+NVP vs NoT/Plc | 2.26 (0.16 to 25.82) | 0.12 to 39.02 |
| ddI+d4T+NVP vs ZDV | 2.22 (0.17 to 19.59) | 0.11 to 31.30 |
| ddI+d4T+NVP vs 3TC+d4T | 5.54 (0.25 to 222.60) | 0.18 to 303.50 |
| ddI+d4T+NVP vs ZDV+3TC | 1.59 (0.10 to 19.46) | 0.07 to 30.57 |
| ddI+d4T+NVP vs ZDV+ddI+NVP | 0.99 (0.06 to 16.79) | 0.05 to 22.63 |
| ZDV+3TC+NVP vs NoT/Plc | 1.29 (0.15 to 12.60) | 0.10 to 19.18 |
| ZDV+3TC+NVP vs ZDV | 1.26 (0.15 to 9.54) | 0.10 to 15.89 |
| ZDV+3TC+NVP vs 3TC+d4T | 3.16 (0.12 to 192.60) | 0.09 to 236.40 |
| ZDV+3TC+NVP vs ZDV+3TC | 0.92 (0.08 to 9.94) | 0.06 to 14.68 |
| ZDV+3TC+NVP vs ZDV+ddI+NVP | 0.58 (0.03 to 13.29) | 0.02 to 18.59 |
| ZDV+3TC+NVP vs ddI+d4T+NVP | 0.58 (0.03 to 13.11) | 0.02 to 20.84 |
| ZDV+3TC+LOP+RIT vs NoT/Plc | 0.60 (0.10 to 4.61) | 0.06 to 7.96 |
| ZDV+3TC+LOP+RIT vs ZDV | 0.60 (0.10 to 3.30) | 0.06 to 5.72 |
| ZDV+3TC+LOP+RIT vs 3TC+d4T | 1.50 (0.07 to 74.27) | 0.05 to 100.50 |
| ZDV+3TC+LOP+RIT vs ZDV+3TC | 0.44 (0.05 to 3.56) | 0.03 to 5.54 |
| ZDV+3TC+LOP+RIT vs ZDV+ddI+NVP | 0.27 (0.02 to 5.34) | 0.01 to 7.38 |
| ZDV+3TC+LOP+RIT vs ddI+d4T+NVP | 0.28 (0.02 to 5.53) | 0.01 to 7.78 |
| ZDV+3TC+LOP+RIT vs ZDV+3TC+NVP | 0.47 (0.03 to 6.94) | 0.03 to 9.89 |
| *Common within-network between-study variance* | 0.27 (0.00 to 2.22) |  |
| *Design-by-treatment interaction model for inconsistency χ² (d.f., P-value, between-study variance)* | 2.36 (1, 0.13, 0.011) | |
| **Major Congenital Malformations** | | |
| ZDV vs NoT/PLC | 0.83 (0.19 to 3.41) | 0.07 to 8.43 |
| 3TC+d4T vs NoT/PLC | 0.34 (0.01 to 7.17) | 0.00 to 11.53 |
| 3TC+d4T vs ZDV | 0.41 (0.01 to 6.02) | 0.01 to 10.47 |
| ZDV+3TCvs NoT/PLC | 1.71 (0.14 to 20.15) | 0.08 to 36.53 |
| ZDV+3TCvs ZDV | 2.06 (0.28 to 15.27) | 0.13 to 31.01 |
| ZDV+3TCvs 3TC+d4T | 5.09 (0.19 to 317.10) | 0.11 to 474.70 |
| ZDV+ddI+NVP vs NoT/PLC | 1.86 (0.08 to 30.52) | 0.05 to 50.99 |
| ZDV+ddI+NVP vs ZDV | 2.26 (0.15 to 25.07) | 0.09 to 47.84 |
| ZDV+ddI+NVP vs 3TC+d4T | 5.43 (0.22 to 278.80) | 0.14 to 393.60 |
| ZDV+ddI+NVP vsZDV+3TC | 1.08 (0.04 to 23.80) | 0.03 to 36.60 |
| ddI+d4T+NVP vs NoT/PLC | 1.85 (0.08 to 33.31) | 0.05 to 56.03 |
| ddI+d4T+NVP vs ZDV | 2.26 (0.14 to 26.25) | 0.09 to 50.78 |
| ddI+d4T+NVP vs 3TC+d4T | 5.45 (0.23 to 272.90) | 0.14 to 403.30 |
| ddI+d4T+NVP vsZDV+3TC | 1.08 (0.04 to 25.48) | 0.03 to 45.07 |
| ddI+d4T+NVP vs ZDV+ddI+NVP | 1.01 (0.05 to 18.76) | 0.03 to 30.76 |
| *Common within-network between-study variance* | 0.37 (0.00 to 3.71) |  |
| *Design-by-treatment interaction model for inconsistency χ² (d.f., P-value, between-study variance)* | Not Applicable - No independent closed loops | |
| **Mother-to-child transmission of HIV** | | |
| [NoT]+[ZDV] vs [NoT/PLC]+[NoT/PLC] | 0.97 (0.20 to 4.71) | 0.14 to 7.25 |
| [ZDV]+[NoT] vs [NoT/PLC]+[NoT/PLC] | 0.42 (0.16 to 1.25) | 0.09 to 2.27 |
| [ZDV]+[NoT] vs [NoT]+[ZDV] | 0.43 (0.09 to 2.08) | 0.06 to 3.04 |
| [ZDV]+[ZDV] vs [NoT/PLC]+[NoT/PLC] | **0.43 (0.16 to 0.80)** | 0.08 to 1.62 |
| [ZDV]+[ZDV] vs [NoT]+[ZDV] | 0.43 (0.09 to 1.79) | 0.05 to 2.59 |
| [ZDV]+[ZDV] vs [ZDV]+[NoT] | 1.01 (0.28 to 2.39) | 0.14 to 4.27 |
| [NoT]+[NVP] vs [NoT/PLC]+[NoT/PLC] | 0.43 (0.16 to 1.15) | 0.08 to 2.13 |
| [NoT]+[NVP] vs [NoT]+[ZDV] | 0.44 (0.07 to 2.68) | 0.05 to 3.90 |
| [NoT]+[NVP] vs [ZDV]+[NoT] | 1.03 (0.23 to 3.91) | 0.14 to 6.27 |
| [NoT]+[NVP] vs [ZDV]+[ZDV] | 1.00 (0.35 to 4.23) | 0.21 to 7.27 |
| [ZDV]+[NVP] vs [NoT/PLC]+[NoT/PLC] | 0.35 (0.11 to 1.19) | 0.06 to 1.98 |
| [ZDV]+[NVP] vs [NoT]+[ZDV] | 0.37 (0.05 to 2.48) | 0.04 to 3.23 |
| [ZDV]+[NVP] vs [ZDV]+[NoT] | 0.84 (0.17 to 3.68) | 0.11 to 5.74 |
| [ZDV]+[NVP] vs [ZDV]+[ZDV] | 0.82 (0.23 to 4.17) | 0.14 to 6.68 |
| [ZDV]+[NVP] vs [NoT]+[NVP] | 0.82 (0.24 to 2.75) | 0.14 to 4.74 |
| *Common within-network between-study variance* | 0.14 (0.00 to 1.89) |  |
| *Design-by-treatment interaction model for inconsistency χ² (d.f., P-value, between-study variance)* | Not Applicable - No independent closed loops | |
| ***Sensitivity Analysis: Higher Methodological Study Quality - Adequacy of Follow-Up for Observational Studies and Incomplete Outcome Data for Randomised Controlled Trials*** | | |
| **Total Congenital Malformations** | | |
| ZDV vs NoT/Plc | 1.07 (0.59 to 2.39) | 0.29 to 4.71 |
| d4T vs NoT/Plc | 4.49 (0.31 to 182.70) | 0.25 to 226.10 |
| d4T vs ZDV | 4.09 (0.31 to 149.40) | 0.25 to 173.30 |
| ddl vs NoT/Plc | 1.06 (0.03 to 61.33) | 0.02 to 70.27 |
| ddl vs ZDV | 0.97 (0.02 to 52.92) | 0.02 to 56.98 |
| ddl vs d4T | 0.24 (0.01 to 3.12) | 0.01 to 4.15 |
| d4T+ddI vs NoT/Plc | 5.97 (0.52 to 260.60) | 0.41 to 306.90 |
| d4T+ddI vs ZDV | 5.48 (0.50 to 209.80) | 0.39 to 263.70 |
| d4T+ddI vs d4T | 1.36 (0.20 to 10.61) | 0.15 to 14.56 |
| d4T+ddI vs ddl | 5.64 (0.49 to 176.10) | 0.41 to 220.20 |
| 3TC+d4T vs NoT/Plc | 0.45 (0.01 to 5.26) | 0.01 to 6.78 |
| 3TC+d4T vs ZDV | 0.41 (0.01 to 4.26) | 0.01 to 5.53 |
| 3TC+d4T vs d4T | 0.09 (0.00 to 3.45) | 0.00 to 4.12 |
| 3TC+d4T vs ddl | 0.39 (0.00 to 35.27) | 0.00 to 37.59 |
| 3TC+d4T vs d4T+ddI | 0.06 (0.00 to 2.30) | 0.00 to 2.64 |
| ZDV+3TC vs NoT/Plc | 2.20 (0.55 to 10.99) | 0.37 to 17.30 |
| ZDV+3TC vs ZDV | 2.08 (0.54 to 7.86) | 0.33 to 12.62 |
| ZDV+3TC vs d4T | 0.50 (0.01 to 9.11) | 0.01 to 11.71 |
| ZDV+3TC vs ddl | 2.15 (0.03 to 101.10) | 0.03 to 122.20 |
| ZDV+3TC vs d4T+ddI | 0.37 (0.01 to 5.92) | 0.01 to 7.28 |
| ZDV+3TC vs 3TC+d4T | 5.01 (0.36 to 192.70) | 0.29 to 228.50 |
| ZDV+3TC+ABC vs NoT/Plc | 0.66 (0.09 to 5.08) | 0.06 to 7.31 |
| ZDV+3TC+ABC vs ZDV | 0.62 (0.08 to 3.80) | 0.06 to 5.47 |
| ZDV+3TC+ABC vs d4T | 0.15 (0.00 to 3.55) | 0.00 to 4.27 |
| ZDV+3TC+ABC vs ddl | 0.63 (0.01 to 37.82) | 0.01 to 42.71 |
| ZDV+3TC+ABC vs d4T+ddI | 0.11 (0.00 to 2.45) | 0.00 to 2.90 |
| ZDV+3TC+ABC vs 3TC+d4T | 1.52 (0.07 to 72.61) | 0.05 to 88.27 |
| ZDV+3TC+ABC vs ZDV+3TC | 0.29 (0.03 to 2.89) | 0.02 to 3.84 |
| ZDV+ddI+NVP vs NoT/Plc | 2.47 (0.21 to 22.50) | 0.16 to 30.24 |
| ZDV+ddI+NVP vs ZDV | 2.29 (0.21 to 17.44) | 0.16 to 23.19 |
| ZDV+ddI+NVP vs d4T | 0.52 (0.01 to 15.66) | 0.01 to 18.62 |
| ZDV+ddI+NVP vs ddl | 2.30 (0.02 to 163.70) | 0.02 to 186.20 |
| ZDV+ddI+NVP vs d4T+ddI | 0.39 (0.00 to 10.24) | 0.00 to 12.08 |
| ZDV+ddI+NVP vs 3TC+d4T | 5.42 (0.29 to 228.10) | 0.24 to 272.80 |
| ZDV+ddI+NVP vs ZDV+3TC | 1.11 (0.07 to 12.45) | 0.06 to 15.82 |
| ZDV+ddI+NVP vs ZDV+3TC+ABC | 3.67 (0.18 to 64.90) | 0.15 to 78.78 |
| ddI+d4T+NVP vs NoT/Plc | 2.59 (0.24 to 22.17) | 0.19 to 29.52 |
| ddI+d4T+NVP vs ZDV | 2.39 (0.23 to 17.32) | 0.18 to 23.14 |
| ddI+d4T+NVP vs d4T | 0.54 (0.01 to 15.60) | 0.01 to 18.13 |
| ddI+d4T+NVP vs ddl | 2.34 (0.03 to 162.50) | 0.02 to 189.90 |
| ddI+d4T+NVP vs d4T+ddI | 0.39 (0.01 to 10.24) | 0.00 to 12.10 |
| ddI+d4T+NVP vs 3TC+d4T | 5.61 (0.31 to 230.30) | 0.25 to 276.20 |
| ddI+d4T+NVP vs ZDV+3TC | 1.14 (0.08 to 12.69) | 0.06 to 16.64 |
| ddI+d4T+NVP vs ZDV+3TC+ABC | 3.78 (0.20 to 64.88) | 0.15 to 82.72 |
| ddI+d4T+NVP vs ZDV+ddI+NVP | 1.04 (0.07 to 13.95) | 0.06 to 17.71 |
| ZDV+3TC+NVP vs NoT/Plc | 1.26 (0.28 to 6.35) | 0.20 to 9.56 |
| ZDV+3TC+NVP vs ZDV | 1.18 (0.29 to 4.74) | 0.19 to 7.15 |
| ZDV+3TC+NVP vs d4T | 0.28 (0.01 to 5.20) | 0.01 to 6.41 |
| ZDV+3TC+NVP vs ddl | 1.20 (0.02 to 59.38) | 0.02 to 66.39 |
| ZDV+3TC+NVP vs d4T+ddI | 0.21 (0.00 to 3.51) | 0.00 to 4.48 |
| ZDV+3TC+NVP vs 3TC+d4T | 2.89 (0.18 to 115.80) | 0.14 to 133.80 |
| ZDV+3TC+NVP vs ZDV+3TC | 0.57 (0.08 to 3.85) | 0.06 to 5.57 |
| ZDV+3TC+NVP vs ZDV+3TC+ABC | 1.89 (0.37 to 10.65) | 0.26 to 15.13 |
| ZDV+3TC+NVP vs ZDV+ddI+NVP | 0.52 (0.04 to 7.95) | 0.03 to 10.38 |
| ZDV+3TC+NVP vs ddI+d4T+NVP | 0.50 (0.04 to 7.61) | 0.03 to 9.20 |
| LOP+RIT vs NoT/Plc | 0.31 (0.04 to 2.65) | 0.03 to 3.84 |
| LOP+RIT vs ZDV | 0.29 (0.04 to 2.01) | 0.03 to 2.77 |
| LOP+RIT vs d4T | 0.07 (0.00 to 1.84) | 0.00 to 2.21 |
| LOP+RIT vs ddl | 0.30 (0.00 to 17.93) | 0.00 to 21.60 |
| LOP+RIT vs d4T+ddI | 0.05 (0.00 to 1.17) | 0.00 to 1.52 |
| LOP+RIT vs 3TC+d4T | 0.74 (0.03 to 37.53) | 0.03 to 42.88 |
| LOP+RIT vs ZDV+3TC | 0.14 (0.01 to 1.56) | 0.01 to 2.04 |
| LOP+RIT vs ZDV+3TC+ABC | 0.48 (0.05 to 4.63) | 0.04 to 5.61 |
| LOP+RIT vs ZDV+ddI+NVP | 0.13 (0.01 to 2.80) | 0.01 to 3.37 |
| LOP+RIT vs ddI+d4T+NVP | 0.12 (0.01 to 2.53) | 0.01 to 3.15 |
| LOP+RIT vs ZDV+3TC+NVP | 0.25 (0.03 to 1.85) | 0.02 to 2.68 |
| ZDV+3TC+LOP+RIT vs NoT/Plc | 0.67 (0.17 to 3.10) | 0.11 to 4.77 |
| ZDV+3TC+LOP+RIT vs ZDV | 0.63 (0.18 to 2.21) | 0.11 to 3.60 |
| ZDV+3TC+LOP+RIT vs d4T | 0.15 (0.00 to 2.70) | 0.00 to 3.24 |
| ZDV+3TC+LOP+RIT vs ddl | 0.65 (0.01 to 28.97) | 0.01 to 33.13 |
| ZDV+3TC+LOP+RIT vs d4T+ddI | 0.11 (0.00 to 1.76) | 0.00 to 2.22 |
| ZDV+3TC+LOP+RIT vs 3TC+d4T | 1.53 (0.11 to 60.41) | 0.09 to 73.17 |
| ZDV+3TC+LOP+RIT vs ZDV+3TC | 0.30 (0.05 to 1.92) | 0.03 to 2.81 |
| ZDV+3TC+LOP+RIT vs ZDV+3TC+ABC | 1.03 (0.19 to 5.60) | 0.14 to 7.46 |
| ZDV+3TC+LOP+RIT vs ZDV+ddI+NVP | 0.28 (0.02 to 4.09) | 0.02 to 5.29 |
| ZDV+3TC+LOP+RIT vs ddI+d4T+NVP | 0.27 (0.02 to 3.93) | 0.02 to 4.87 |
| ZDV+3TC+LOP+RIT vs ZDV+3TC+NVP | 0.54 (0.13 to 2.16) | 0.09 to 3.39 |
| ZDV+3TC+LOP+RIT vs LOP+RIT | 2.14 (0.48 to 9.29) | 0.34 to 13.56 |
| *Common within-network between-study variance* | 0.14 (0.00 to 1.73) |  |
| *Design-by-treatment interaction model for inconsistency χ² (d.f., P-value, between-study variance)* | 0.03 (1, 0.87, 0.00) | |
| **Major Congenital Malformations** | | |
| ZDV vs NoT/PLC | 0.80 (0.30 to 1.79) | 0.13 to 4.06 |
| 3TC+d4T vs NoT/PLC | 0.32 (0.01 to 3.98) | 0.01 to 5.33 |
| 3TC+d4T vs ZDV | 0.42 (0.01 to 4.64) | 0.01 to 6.24 |
| ZDV+3TCvs NoT/PLC | 1.63 (0.26 to 8.63) | 0.15 to 13.81 |
| ZDV+3TCvs ZDV | 2.05 (0.45 to 9.26) | 0.27 to 15.79 |
| ZDV+3TCvs 3TC+d4T | 5.01 (0.30 to 228.60) | 0.22 to 262.80 |
| ZDV+ddI+NVP vs NoT/PLC | 1.77 (0.13 to 16.87) | 0.09 to 23.45 |
| ZDV+ddI+NVP vs ZDV | 2.28 (0.19 to 18.97) | 0.14 to 29.03 |
| ZDV+ddI+NVP vs 3TC+d4T | 5.38 (0.29 to 202.60) | 0.22 to 246.50 |
| ZDV+ddI+NVP vsZDV+3TC | 1.09 (0.06 to 14.96) | 0.05 to 19.39 |
| ddI+d4T+NVP vs NoT/PLC | 1.76 (0.13 to 17.64) | 0.09 to 23.87 |
| ddI+d4T+NVP vs ZDV | 2.24 (0.19 to 19.49) | 0.14 to 28.68 |
| ddI+d4T+NVP vs 3TC+d4T | 5.39 (0.26 to 221.40) | 0.21 to 293.90 |
| ddI+d4T+NVP vsZDV+3TC | 1.09 (0.06 to 14.97) | 0.05 to 20.66 |
| ddI+d4T+NVP vs ZDV+ddI+NVP | 1.02 (0.07 to 15.49) | 0.05 to 21.16 |
| *Common within-network between-study variance* | 0.16 (0.00 to 2.40) |  |
| *Design-by-treatment interaction model for inconsistency χ² (d.f., P-value, between-study variance)* | Not Applicable - No independent closed loops | |
| **Mother-to-child transmission of HIV** | | |
| [NoT]+[ZDV] vs [NoT/PLC]+[NoT/PLC] | 1.27 (0.17 to 10.11) | 0.08 to 22.51 |
| [ZDV]+[NoT] vs [NoT/PLC]+[NoT/PLC] | 0.63 (0.07 to 6.03) | 0.03 to 12.24 |
| [ZDV]+[NoT] vs [NoT]+[ZDV] | 0.51 (0.06 to 4.11) | 0.03 to 8.61 |
| [ZDV]+[ZDV] vs [NoT/PLC]+[NoT/PLC] | **0.43 (0.13 to 0.95)** | 0.04 to 2.93 |
| [ZDV]+[ZDV] vs [NoT]+[ZDV] | 0.33 (0.05 to 1.94) | 0.02 to 4.11 |
| [ZDV]+[ZDV] vs [ZDV]+[NoT] | 0.66 (0.07 to 4.65) | 0.04 to 9.36 |
| *Common within-network between-study variance* | 0.52 (0.00 to 3.33) |  |
| *Design-by-treatment interaction model for inconsistency χ² (d.f., P-value, between-study variance)* | 3.45 (3, 0.33, 0.05) | |
| ***Sensitivity Analysis: Antenatal Care*** | | |
| **Total Congenital Malformations** | | |
| ZDV vs NoT/Plc | 1.18 (0.47 to 3.94) | 0.17 to 11.60 |
| d4T vs NoT/Plc | 5.03 (0.25 to 267.00) | 0.16 to 371.60 |
| d4T vs ZDV | 4.20 (0.24 to 170.30) | 0.14 to 256.60 |
| ddl vs NoT/Plc | 1.23 (0.02 to 76.85) | 0.02 to 114.50 |
| ddl vs ZDV | 1.01 (0.02 to 51.64) | 0.01 to 77.32 |
| ddl vs d4T | 0.24 (0.01 to 4.32) | 0.00 to 6.75 |
| d4T+ddI vs NoT/Plc | 6.83 (0.41 to 331.10) | 0.25 to 464.80 |
| d4T+ddI vs ZDV | 5.60 (0.37 to 215.10) | 0.22 to 316.80 |
| d4T+ddI vs d4T | 1.34 (0.13 to 15.77) | 0.07 to 27.93 |
| d4T+ddI vs ddl | 5.56 (0.36 to 226.90) | 0.22 to 306.20 |
| ZDV+3TC vs NoT/Plc | 1.12 (0.25 to 3.73) | 0.10 to 8.92 |
| ZDV+3TC vs ZDV | 0.96 (0.17 to 2.73) | 0.07 to 6.35 |
| ZDV+3TC vs d4T | 0.22 (0.00 to 4.41) | 0.00 to 6.37 |
| ZDV+3TC vs ddl | 0.92 (0.01 to 49.03) | 0.01 to 66.28 |
| ZDV+3TC vs d4T+ddI | 0.16 (0.00 to 2.82) | 0.00 to 4.34 |
| ZDV+3TC+ABC vs NoT/Plc | 0.71 (0.06 to 11.60) | 0.04 to 19.65 |
| ZDV+3TC+ABC vs ZDV | 0.61 (0.06 to 6.70) | 0.03 to 12.01 |
| ZDV+3TC+ABC vs d4T | 0.15 (0.00 to 5.89) | 0.00 to 8.67 |
| ZDV+3TC+ABC vs ddl | 0.61 (0.01 to 57.73) | 0.00 to 84.33 |
| ZDV+3TC+ABC vs d4T+ddI | 0.11 (0.00 to 4.04) | 0.00 to 6.24 |
| ZDV+3TC+ABC vs ZDV+3TC | 0.65 (0.05 to 13.41) | 0.03 to 23.52 |
| ZDV+3TC+NVP vs NoT/Plc | 1.40 (0.21 to 12.33) | 0.11 to 25.42 |
| ZDV+3TC+NVP vs ZDV | 1.19 (0.20 to 6.68) | 0.10 to 14.69 |
| ZDV+3TC+NVP vs d4T | 0.28 (0.00 to 7.62) | 0.00 to 12.56 |
| ZDV+3TC+NVP vs ddl | 1.18 (0.02 to 87.19) | 0.01 to 123.90 |
| ZDV+3TC+NVP vs d4T+ddI | 0.21 (0.00 to 5.37) | 0.00 to 8.37 |
| ZDV+3TC+NVP vs ZDV+3TC | 1.26 (0.18 to 15.81) | 0.09 to 31.14 |
| ZDV+3TC+NVP vs ZDV+3TC+ABC | 1.94 (0.25 to 15.62) | 0.13 to 28.67 |
| LOP+RIT vs NoT/Plc | 0.35 (0.03 to 6.77) | 0.01 to 12.50 |
| LOP+RIT vs ZDV | 0.30 (0.02 to 4.00) | 0.01 to 7.30 |
| LOP+RIT vs d4T | 0.07 (0.00 to 3.30) | 0.00 to 5.05 |
| LOP+RIT vs ddl | 0.30 (0.00 to 31.40) | 0.00 to 43.12 |
| LOP+RIT vs d4T+ddI | 0.05 (0.00 to 2.25) | 0.00 to 3.32 |
| LOP+RIT vs ZDV+3TC | 0.32 (0.02 to 8.05) | 0.01 to 14.25 |
| LOP+RIT vs ZDV+3TC+ABC | 0.49 (0.03 to 8.81) | 0.02 to 14.36 |
| LOP+RIT vs ZDV+3TC+NVP | 0.26 (0.02 to 3.50) | 0.01 to 6.23 |
| ZDV+3TC+LOP+RIT vs NoT/Plc | 0.74 (0.12 to 6.08) | 0.06 to 11.56 |
| ZDV+3TC+LOP+RIT vs ZDV | 0.63 (0.12 to 3.33) | 0.05 to 6.94 |
| ZDV+3TC+LOP+RIT vs d4T | 0.15 (0.00 to 4.01) | 0.00 to 6.27 |
| ZDV+3TC+LOP+RIT vs ddl | 0.62 (0.01 to 43.40) | 0.01 to 59.79 |
| ZDV+3TC+LOP+RIT vs d4T+ddI | 0.11 (0.00 to 2.63) | 0.00 to 4.12 |
| ZDV+3TC+LOP+RIT vs ZDV+3TC | 0.66 (0.10 to 7.98) | 0.06 to 14.68 |
| ZDV+3TC+LOP+RIT vs ZDV+3TC+ABC | 1.03 (0.13 to 8.54) | 0.06 to 16.57 |
| ZDV+3TC+LOP+RIT vs ZDV+3TC+NVP | 0.53 (0.09 to 3.07) | 0.04 to 6.34 |
| ZDV+3TC+LOP+RIT vs LOP+RIT | 2.08 (0.29 to 15.12) | 0.15 to 31.75 |
| *Common within-network between-study variance* | 0.47 (0.00 to 2.99) |  |
| *Design-by-treatment interaction model for inconsistency χ² (d.f., P-value, between-study variance)* | 2.26 (4, 0.69, 0.53) | |
| **Major Congential Malformations** | | |
| ZDV vs NoT/PLC | 0.75 (0.14 to 3.70) | 0.04 to 11.41 |
| ZDV+3TCvs NoT/PLC | 0.83 (0.04 to 6.23) | 0.02 to 13.22 |
| ZDV+3TCvs ZDV | 1.14 (0.08 to 4.85) | 0.03 to 12.70 |
| *Common within-network between-study variance* | 0.69 (0.00 to 4.66) |  |
| *Design-by-treatment interaction model for inconsistency χ² (d.f., P-value, between-study variance)* | 1.17 (2, 0.56, 0.76) | |
| **Mother-to-child transmission of HIV** | | |
| [NoT]+[ZDV] vs [NoT/PLC]+[NoT/PLC] | 1.22 (0.28 to 6.52) | 0.20 to 10.82 |
| [ZDV]+[NoT] vs [NoT/PLC]+[NoT/PLC] | 0.45 (0.20 to 1.53) | 0.11 to 2.77 |
| [ZDV]+[NoT] vs [NoT]+[ZDV] | 0.38 (0.08 to 1.85) | 0.05 to 2.74 |
| [ZDV]+[ZDV] vs [NoT/PLC]+[NoT/PLC] | 0.57 (0.27 to 1.17) | 0.13 to 2.34 |
| [ZDV]+[ZDV] vs [NoT]+[ZDV] | 0.46 (0.09 to 1.94) | 0.06 to 2.85 |
| [ZDV]+[ZDV] vs [ZDV]+[NoT] | 1.24 (0.35 to 3.01) | 0.19 to 5.12 |
| *Common within-network between-study variance* | 0.14 (0.00 to 1.93) |  |
| *Design-by-treatment interaction model for inconsistency χ² (d.f., P-value, between-study variance)* | 4.48 (2, 0.11, 0.01) | |
| ***Sensitivity Analysis: LMIC*** | | |
| **Total Congenital Malformations** | | |
| *ZDV vs NoT/Plc* | 1.12 (0.14 to 10.47) | 0.04 to 39.68 |
| *ZDV+3TC vs NoT/Plc* | 1.04 (0.07 to 14.36) | 0.03 to 43.00 |
| *ZDV+3TC vs ZDV* | 0.93 (0.03 to 23.78) | 0.01 to 64.84 |
| *ZDV+3TC+NVP vs NoT/Plc* | 1.41 (0.04 to 62.33) | 0.02 to 140.30 |
| *ZDV+3TC+NVP vs ZDV* | 1.28 (0.07 to 25.46) | 0.03 to 71.95 |
| *ZDV+3TC+NVP vs ZDV+3TC* | 1.35 (0.02 to 140.90) | 0.01 to 303.60 |
| *ZDV+3TC+LOP+RIT vs NoT/Plc* | 0.66 (0.02 to 24.02) | 0.01 to 61.20 |
| *ZDV+3TC+LOP+RIT vs ZDV* | 0.61 (0.03 to 9.56) | 0.01 to 28.53 |
| *ZDV+3TC+LOP+RIT vs ZDV+3TC* | 0.64 (0.01 to 56.74) | 0.00 to 116.30 |
| *ZDV+3TC+LOP+RIT vs ZDV+3TC+NVP* | 0.48 (0.01 to 25.76) | 0.00 to 58.12 |
| *Common within-network between-study variance* | 1.01 (0.01 to 5.91) |  |
| *Design-by-treatment interaction model for inconsistency χ² (d.f., P-value, between-study variance)* | Not Applicable - No independent closed loops | |
| **Major Congential Malformations** | | |
| ZDV vs NoT/PLC | 0.33 (0.06 to 1.64) | - |
| *Common within-network between-study variance* | N/A |  |
| *Design-by-treatment interaction model for inconsistency χ² (d.f., P-value, between-study variance)* | N/A | |
| **Mother-to-child transmission of HIV** | | |
| [ZDV]+[ZDV] vs [NoT/PLC]+[NoT/PLC] | 0.70 (0.39 to 1.24) | - |
| *Common within-network between-study variance* | N/A |  |
| *Design-by-treatment interaction model for inconsistency χ² (d.f., P-value, between-study variance)* | N/A | |
| ***Sensitivity Analysis: Illicit Drugs*** | | |
| **Total Congenital Malformations** | | |
| *ZDV vs NoT/Plc* | 1.34 (0.28 to 6.35) | 0.14 to 12.98 |
| *ZDV+3TC vs NoT/Plc* | 2.77 (0.24 to 29.41) | 0.16 to 49.30 |
| *ZDV+3TC vs ZDV* | 2.07 (0.36 to 12.19) | 0.18 to 24.35 |
| *Common within-network between-study variance* | 0.24 (0.00 to 3.27) |  |
| *Design-by-treatment interaction model for inconsistency χ² (d.f., P-value, between-study variance)* | Not Applicable - No independent closed loops | |
| **Major Congential Malformations** | | |
| ZDV vs NoT/PLC | 1.02 (0.02 to 19.04) | 0.01 to 36.87 |
| ZDV+3TCvs NoT/PLC | 2.05 (0.03 to 78.07) | 0.02 to 131.40 |
| ZDV+3TCvs ZDV | 2.07 (0.22 to 18.78) | 0.10 to 49.26 |
| *Common within-network between-study variance* | 0.45 (0.00 to 5.00) |  |
| *Design-by-treatment interaction model for inconsistency χ² (d.f., P-value, between-study variance)* | N/A | |
| **Mother-to-child transmission of HIV** | | |
| [NoT]+[ZDV] vs [NoT/PLC]+[NoT/PLC] | 0.80 (0.11 to 5.48) | 0.05 to 9.52 |
| [ZDV]+[NoT] vs [NoT/PLC]+[NoT/PLC] | 0.39 (0.10 to 1.60) | 0.04 to 3.59 |
| [ZDV]+[NoT] vs [NoT]+[ZDV] | 0.50 (0.08 to 3.12) | 0.04 to 6.63 |
| [ZDV]+[ZDV] vs [NoT/PLC]+[NoT/PLC] | **0.30 (0.06 to 0.81)** | 0.03 to 1.87 |
| [ZDV]+[ZDV] vs [NoT]+[ZDV] | 0.36 (0.05 to 1.75) | 0.02 to 3.57 |
| [ZDV]+[ZDV] vs [ZDV]+[NoT] | 0.75 (0.14 to 2.53) | 0.06 to 5.93 |
| *Common within-network between-study variance* | 0.31 (0.00 to 3.36) |  |
| *Design-by-treatment interaction model for inconsistency χ² (d.f., P-value, between-study variance)* | 1.99 (2, 0.3699, 0.481) | |
| ***Sensitivity Analysis: TB Co-Infection*** | | |
| **Total Congenital Malformations** | | |
| ZDV vs NoT/Plc | 1.56 (0.12 to 28.77) | 0.05 to 56.25 |
| d4T vs NoT/Plc | 6.93 (0.10 to 883.10) | 0.07 to 1390.00 |
| d4T vs ZDV | 4.27 (0.19 to 215.10) | 0.10 to 374.40 |
| ddl vs NoT/Plc | 1.58 (0.01 to 243.70) | 0.01 to 375.70 |
| ddl vs ZDV | 0.99 (0.01 to 62.77) | 0.01 to 97.48 |
| ddl vs d4T | 0.24 (0.00 to 4.98) | 0.00 to 9.27 |
| d4T+ddI vs NoT/Plc | 9.37 (0.17 to 1067.00) | 0.10 to 1613.00 |
| d4T+ddI vs ZDV | 5.81 (0.27 to 283.70) | 0.13 to 465.20 |
| d4T+ddI vs d4T | 1.37 (0.10 to 20.33) | 0.05 to 40.54 |
| d4T+ddI vs ddl | 5.66 (0.29 to 318.50) | 0.15 to 504.30 |
| *Common within-network between-study variance* | 0.46 (0.00 to 5.03) |  |
| *Design-by-treatment interaction model for inconsistency χ² (d.f., P-value, between-study variance)* | Not Applicable - No independent closed loops | |
| ***Sensitivity Analysis: Smoke*** | | |
| **Total Congenital Malformations** | | |
| ZDV+3TC+LOP+RIT vs LOP+RIT | 2.11 (0.88 to 5.05) | - |
| ZDV vs NoT/Plc | 1.37 (0.15 to 12.66) | - |
| *Common within-network between-study variance* | N/A |  |
| *Design-by-treatment interaction model for inconsistency χ² (d.f., P-value, between-study variance)* | N/A | |
| ***Sensitivity Analysis: Alcohol*** | | |
| **Total Congenital Malformations** | | |
| ZDV vs NoT/Plc | 1.37 (0.15 to 12.66) | - |
| *Common within-network between-study variance* | N/A |  |
| *Design-by-treatment interaction model for inconsistency χ² (d.f., P-value, between-study variance)* | N/A | |
| **Mother-to-child transmission of HIV** | | |
| [NoT]+[NVP] vs [NoT/PLC]+[NoT/PLC] | 0.41 (0.14 to 1.22) | - |
| [ZDV]+[ZDV] vs [NoT]+[ZDV] | **0.06 (0.00 to 0.73)** | - |
| *Common within-network between-study variance* | N/A |  |
| *Design-by-treatment interaction model for inconsistency χ² (d.f., P-value, between-study variance)* | N/A | |
|  | ***Sensitivity Analysis: Smoke/ Alcohol*** |  |
| **Major Congential Malformations** | | |
| ZDV vs NoT/Plc | 1.37 (0.15 to 12.66) | - |
| *Common within-network between-study variance* | N/A |  |
| *Design-by-treatment interaction model for inconsistency χ² (d.f., P-value, between-study variance)* | N/A | |
|  | ***Sensitivity Analysis: CD4+ count (<200)*** |  |
| **Total Congenital Malformations** | | |
| ZDV vs NoT/Plc | 1.02 (0.52 to 2.31) | 0.28 to 4.48 |
| ZDV+3TC+ABC vs NoT/Plc | 0.61 (0.08 to 4.79) | 0.06 to 6.44 |
| ZDV+3TC+ABC vs ZDV | 0.60 (0.09 to 3.96) | 0.07 to 5.50 |
| ZDV+3TC+NVP vs NoT/Plc | 1.20 (0.25 to 6.20) | 0.18 to 9.50 |
| ZDV+3TC+NVP vs ZDV | 1.16 (0.28 to 4.79) | 0.19 to 7.49 |
| ZDV+3TC+NVP vs ZDV+3TC+ABC | 1.95 (0.38 to 10.65) | 0.26 to 15.11 |
| LOP+RIT vs NoT/Plc | 0.30 (0.04 to 2.49) | 0.03 to 3.28 |
| LOP+RIT vs ZDV | 0.29 (0.04 to 1.91) | 0.03 to 2.73 |
| LOP+RIT vs ZDV+3TC+ABC | 0.49 (0.05 to 4.47) | 0.04 to 6.08 |
| LOP+RIT vs ZDV+3TC+NVP | 0.25 (0.03 to 1.85) | 0.02 to 2.58 |
| ZDV+3TC+LOP+RIT vs NoT/Plc | 0.64 (0.16 to 2.83) | 0.10 to 4.46 |
| ZDV+3TC+LOP+RIT vs ZDV | 0.62 (0.18 to 2.15) | 0.11 to 3.37 |
| ZDV+3TC+LOP+RIT vs ZDV+3TC+ABC | 1.03 (0.19 to 5.67) | 0.14 to 8.26 |
| ZDV+3TC+LOP+RIT vs ZDV+3TC+NVP | 0.53 (0.13 to 2.10) | 0.08 to 3.18 |
| ZDV+3TC+LOP+RIT vs LOP+RIT | 2.13 (0.51 to 8.83) | 0.32 to 13.35 |
| *Common within-network between-study variance* | 0.14 (0.00 to 1.69) |  |
| *Design-by-treatment interaction model for inconsistency χ² (d.f., P-value, between-study variance)* | 0.03 (1, 0.87, 0.00) | |
| **Major Congential Malformations** | | |
| ZDV+3TCvs ZDV | 0.79 (0.52 to 1.21) | - |
| *Common within-network between-study variance* | 0.00 |  |
| *Design-by-treatment interaction model for inconsistency χ² (d.f., P-value, between-study variance)* | N/A | |
| **Mother-to-child transmission of HIV** | | |
| [NoT]+[ZDV] vs [NoT/PLC]+[NoT/PLC] | 0.79 (0.11 to 5.91) | 0.06 to 11.56 |
| [ZDV]+[NoT] vs [NoT/PLC]+[NoT/PLC] | 0.39 (0.09 to 1.62) | 0.04 to 3.85 |
| [ZDV]+[NoT] vs [NoT]+[ZDV] | 0.48 (0.07 to 3.29) | 0.04 to 6.44 |
| [ZDV]+[ZDV] vs [NoT/PLC]+[NoT/PLC] | **0.30 (0.06 to 0.84)** | 0.02 to 1.96 |
| [ZDV]+[ZDV] vs [NoT]+[ZDV] | 0.36 (0.05 to 1.86) | 0.02 to 3.52 |
| [ZDV]+[ZDV] vs [ZDV]+[NoT] | 0.76 (0.13 to 2.60) | 0.06 to 5.88 |
| [NoT]+[NVP] vs [NoT/PLC]+[NoT/PLC] | 0.44 (0.06 to 3.80) | 0.03 to 6.83 |
| [NoT]+[NVP] vs [NoT]+[ZDV] | 0.56 (0.03 to 10.25) | 0.02 to 18.44 |
| [NoT]+[NVP] vs [ZDV]+[NoT] | 1.15 (0.09 to 15.27) | 0.05 to 25.13 |
| [NoT]+[NVP] vs [ZDV]+[ZDV] | 1.51 (0.17 to 23.75) | 0.10 to 39.32 |
| *Common within-network between-study variance* | 0.32 (0.00 to 3.36) |  |
| *Design-by-treatment interaction model for inconsistency χ² (d.f., P-value, between-study variance)* | 2.04 (2, 0.36, 0.44) | |
| **Treatment Abbreviations:** ART, Antiretroviral Therapy; HAART, Highly Active Anti-Retroviral; ABC, Abacavir; ddI, Didanosine; CM, Congenital Malformations; IND, Indinavir; 3TC, Lamivudine; LMIC, Low and Middle-Income Countries; LOP, Lopinavir; MTCT, Mother to Child Transmission; N/A, Not Applicable; NVP, Nevirapine; NLF Nelfinavir; NoT, No Treatment; Plc, Placebo; SAQ, Saquinavir; d4T Stavudine; EFV, Sustiva; RIT, Ritonavir; ZDV, Zidovudine. | | |
| **Note:** Statistically significant results are **bolded**. | | |
