# Supplementary material for: Comparative safety and effectiveness of perinatal antiretroviral therapies for HIV-infected women and their children: Systematic review and network meta-analysis including different study designs
Source: PLoS One. 2018 Jun 18;13(6):e0198447. doi: 10.1371/journal.pone.0198447 (PMC6005568; doi:10.1371/journal.pone.0198447)
Supplement: S22 Appendix — (DOCX) [file pone.0198447.s022.docx]

# S22 Appendix. Additional Analyses: Drug Categories

**Subgroup, Sensitivity, Meta-regression, and Schmitz Network Meta-analysis results (All outcomes) Including Antiretroviral Therapy Drug Categories**

| ***Treatment Comparison*** | ***NMA Odds Ratio Estimate (Credible Interval)*** | ***Predictive Intervals*** |
| --- | --- | --- |
| ***Subgroup Analysis: Large Studies Only (>300 patients per study)*** | | |
| **Total Congenital Malformations** | | |
| ART-mono vs NoT/PLC | 0.85 (0.45 to 1.92) | 0.23 to 3.53 |
| ART-dual vs NoT/PLC | 1.40 (0.68 to 3.67) | 0.38 to 6.64 |
| ART-dual vs ART-mono | 1.65 (0.78 to 3.64) | 0.43 to 6.69 |
| HAART vs NoT/PLC | 0.74 (0.26 to 2.25) | 0.16 to 3.57 |
| HAART vs ART-mono | 0.87 (0.33 to 2.17) | 0.19 to 3.75 |
| HAART vs ART-dual | 0.53 (0.16 to 1.51) | 0.10 to 2.32 |
| *Common within-network between-study variance* | 0.15 (0.00 to 1.48) |  |
| *Design-by-treatment interaction model for inconsistency χ² (d.f., P-value, between-study variance)* | 4.01 (4, 0.41, 0.01) | |
| **Major Congenital Malformations** | | |
| ART-mono vs NoT/PLC | 0.81 (0.27 to 2.16) | 0.12 to 4.68 |
| ART-dual vs NoT/PLC | 1.76 (0.35 to 7.60) | 0.19 to 13.96 |
| ART-dual vs ART-mono | 2.17 (0.59 to 7.56) | 0.30 to 15.47 |
| HAART vs NoT/PLC | 0.98 (0.17 to 4.50) | 0.10 to 7.82 |
| HAART vs ART-mono | 1.21 (0.25 to 5.07) | 0.13 to 9.47 |
| HAART vs ART-dual | 0.56 (0.10 to 3.06) | 0.06 to 5.31 |
| *Common within-network between-study variance* | 0.21 (0.00 to 2.65) |  |
| *Design-by-treatment interaction model for inconsistency χ² (d.f., P-value, between-study variance)* | 3.27 (2, 0.20, 0.00) | |
| **Mother-to-child transmission of HIV** | | |
| [NoT]+[ART-mono] vs [NoT/PLC]+[NoT/PLC] | 0.41 (0.15 to 1.57) | 0.06 to 4.17 |
| [ART-mono]+[NoT] vs [NoT/PLC]+[NoT/PLC] | 0.57 (0.12 to 3.15) | 0.06 to 6.86 |
| [ART-mono]+[NoT] vs [NoT]+[ART-mono] | 1.40 (0.19 to 8.26) | 0.09 to 16.84 |
| [ART-mono]+[ART-mono] vs [NoT/PLC]+[NoT/PLC] | **0.28 (0.08 to 0.78)** | 0.03 to 2.12 |
| [ART-mono]+[ART-mono] vs [NoT]+[ART-mono] | 0.67 (0.14 to 2.02) | 0.06 to 4.83 |
| [ART-mono]+[ART-mono] vs [ART-mono]+[NoT] | 0.48 (0.06 to 2.76) | 0.03 to 5.60 |
| [ART-dual]+[NoT] vs [NoT/PLC]+[NoT/PLC] | **0.06 (0.01 to 0.47)** | 0.00 to 0.89 |
| [ART-dual]+[NoT] vs [NoT]+[ART-mono] | 0.14 (0.01 to 1.25) | 0.01 to 2.35 |
| [ART-dual]+[NoT] vs [ART-mono]+[NoT] | **0.10 (0.01 to 0.85)** | 0.00 to 1.64 |
| [ART-dual]+[NoT] vs [ART-mono]+[ART-mono] | 0.21 (0.02 to 2.34) | 0.01 to 4.13 |
| [HAART]+[NoT] vs [NoT/PLC]+[NoT/PLC] | **0.19 (0.07 to 0.55)** | 0.03 to 1.51 |
| [HAART]+[NoT] vs [NoT]+[ART-mono] | 0.46 (0.12 to 1.33) | 0.05 to 3.28 |
| [HAART]+[NoT] vs [ART-mono]+[NoT] | 0.33 (0.06 to 1.68) | 0.03 to 3.60 |
| [HAART]+[NoT] vs [ART-mono]+[ART-mono] | 0.68 (0.21 to 2.93) | 0.09 to 6.80 |
| [HAART]+[NoT] vs [ART-dual]+[NoT] | 3.22 (0.39 to 36.57) | 0.21 to 67.54 |
| *Common within-network between-study variance* | 0.51 (0.09 to 2.67) |  |
| *Design-by-treatment interaction model for inconsistency χ² (d.f., P-value, between-study variance)* | 26.52 (5, 0.00, 0.00) | |
| **Preterm Births** | | |
| ART-mono vs NoT/PLC | **0.52 (0.30 to 0.90)** | 0.07 to 3.73 |
| ART-dual vs NoT/PLC | **0.14 (0.05 to 0.41)** | 0.02 to 1.24 |
| ART-dual vs ART-mono | **0.27 (0.09 to 0.76)** | 0.03 to 2.46 |
| HAART vs NoT/PLC | 0.70 (0.34 to 1.43) | 0.09 to 5.32 |
| HAART vs ART-mono | 1.33 (0.67 to 2.71) | 0.18 to 9.94 |
| HAART vs ART-dual | **4.83 (1.74 to 14.75)** | 0.58 to 43.61 |
| *Common within-network between-study variance* | 0.85 (0.43 to 1.84) |  |
| *Design-by-treatment interaction model for inconsistency χ² (d.f., P-value, between-study variance)* | N/A | |
| ***Subgroup Analysis: Infants only*** | | |
| **Infant and Child Deaths** | | |
| ART-mono vs NoT/PLC | 0.64 (0.33 to 1.44) | 0.13 to 3.68 |
| ART-dual vs NoT/PLC | 0.92 (0.32 to 6.16) | 0.17 to 11.41 |
| ART-dual vs ART-mono | 1.44 (0.42 to 9.12) | 0.22 to 16.90 |
| HAART vs NoT/PLC | 0.44 (0.16 to 1.35) | 0.08 to 2.94 |
| HAART vs ART-mono | 0.68 (0.25 to 1.77) | 0.11 to 4.11 |
| HAART vs ART-dual | 0.47 (0.07 to 1.78) | 0.04 to 3.23 |
| *Common within-network between-study variance* | 0.29 (0.00 to 2.16) |  |
| *Design-by-treatment interaction model for inconsistency χ² (d.f., P-value, between-study variance)* | N/A | |
| ***Meta-regression Analysis: Baseline Risk*** | | |
| **Total Congenital Malformations** | | |
| ART-mono vs NoT/PLC | 1.69 (0.68 to 5.19) | 0.34 to 10.33 |
| ART-dual vs NoT/PLC | 2.01 (0.71 to 6.64) | 0.38 to 12.59 |
| ART-dual vs ART-mono | 1.20 (0.55 to 2.32) | 0.24 to 5.19 |
| HAART vs NoT/PLC | 2.33 (0.66 to 9.74) | 0.38 to 16.79 |
| HAART vs ART-mono | 1.38 (0.65 to 2.74) | 0.29 to 5.95 |
| HAART vs ART-dual | 1.16 (0.51 to 2.69) | 0.24 to 5.86 |
| *Common within-network between-study variance* | 0.35 (0.01 to 1.37) |  |
| *Regression coefficient* | 1.17 (0.91 to 1.57) |  |
| Model fit measures and diagnostics | Residual deviance =47.31 Data points = 41  Effective number of parameters = 33.59 DIC = 80.9 | |
| **Mother-to-child transmission of HIV** | | |
| [NoT]+[ART-mono] vs [NoT/PLC]+[NoT/PLC] | 0.43 (0.15 to 1.43) | 0.06 to 3.99 |
| [ART-mono]+[NoT] vs [NoT/PLC]+[NoT/PLC] | 0.68 (0.13 to 3.22) | 0.06 to 6.98 |
| [ART-mono]+[NoT] vs [NoT]+[ART-mono] | 1.56 (0.30 to 6.88) | 0.13 to 14.36 |
| [ART-mono]+[ART-mono] vs [NoT/PLC]+[NoT/PLC] | 0.49 (0.13 to 1.60) | 0.05 to 3.78 |
| [ART-mono]+[ART-mono] vs [NoT]+[ART-mono] | 1.11 (0.28 to 3.68) | 0.11 to 8.72 |
| [ART-mono]+[ART-mono] vs [ART-mono]+[NoT] | 0.72 (0.22 to 2.23) | 0.08 to 6.07 |
| [ART-dual]+[NoT] vs [NoT/PLC]+[NoT/PLC] | **0.05 (0.00 to 0.40)** | 0.00 to 0.80 |
| [ART-dual]+[NoT] vs [NoT]+[ART-mono] | 0.12 (0.01 to 1.02) | 0.00 to 1.87 |
| [ART-dual]+[NoT] vs [ART-mono]+[NoT] | **0.07 (0.01 to 0.77)** | 0.00 to 1.46 |
| [ART-dual]+[NoT] vs [ART-mono]+[ART-mono] | 0.10 (0.01 to 1.06) | 0.00 to 1.93 |
| [HAART]+[NoT] vs [NoT/PLC]+[NoT/PLC] | **0.21 (0.05 to 0.80)** | 0.02 to 1.88 |
| [HAART]+[NoT] vs [NoT]+[ART-mono] | 0.49 (0.11 to 1.57) | 0.04 to 3.63 |
| [HAART]+[NoT] vs [ART-mono]+[NoT] | 0.31 (0.09 to 1.06) | 0.03 to 2.66 |
| [HAART]+[NoT] vs [ART-mono]+[ART-mono] | 0.43 (0.16 to 1.19) | 0.05 to 3.34 |
| [HAART]+[NoT] vs [ART-dual]+[NoT] | 4.10 (0.42 to 54.58) | 0.22 to 87.80 |
| *Common within-network between-study variance* | 0.65 (0.18 to 2.18) |  |
| *Regression coefficient* | 1.27 (0.79 to 1.97) |  |
| *Model fit measures and diagnostics* | Residual deviance = 36.21 Data points = 32  Effective number of parameters = 29.94 DIC = 66.15 | |
| ***Meta-regression Analysis: Age*** | | |
| **Total Congenital Malformations** | | |
| ART-mono vs NoT/PLC | 0.58 (0.27 to 1.28) | 0.18 to 2.07 |
| ART-dual vs NoT/PLC | 1.20 (0.62 to 2.29) | 0.37 to 3.83 |
| ART-dual vs ART-mono | 2.06 (0.92 to 4.30) | 0.59 to 6.62 |
| HAART vs NoT/PLC | 0.67 (0.27 to 1.66) | 0.18 to 2.51 |
| HAART vs ART-mono | 1.14 (0.59 to 2.18) | 0.35 to 3.55 |
| HAART vs ART-dual | 0.55 (0.25 to 1.29) | 0.16 to 2.01 |
| *Common within-network between-study variance* | 0.13 (0.00 to 0.89) |  |
| *Regression coefficient* | 1.16 (0.85 to 1.54) |  |
| *Model fit measures and diagnostics* | Residual deviance = 27.98 Data points = 28  Effective number of parameters = 20.34 DIC = 48.32 | |
| ***Schmitz Model – All study designs*** | | |
| **Total Congenital Malformations** | | |
| ART-mono vs NoT/PLC | 0.99 (0.44 to 2.38) | - |
| ART-dual vs NoT/PLC | 1.16 (0.46 to 3.12) | - |
| ART-dual vs ART-mono | 1.17 (0.38 to 3.57) | - |
| HAART vs NoT/PLC | 1.16 (0.43 to 3.13) | - |
| HAART vs ART-mono | 1.17 (0.36 to 3.57) | - |
| HAART vs ART-dual | 1.00 (0.30 to 3.22) | - |
| *Common within-network between-study variance* | 0.05 (0.00 to 1.27) |  |
| **Major Congenital Malformations** | | |
| ART-mono vs NoT/PLC | 0.58 (0.12 to 2.41) | - |
| ART-dual vs NoT/PLC | 1.25 (0.43 to 3.32) | - |
| ART-dual vs ART-mono | 2.15 (0.47 to 11.02) | - |
| HAART vs NoT/PLC | 0.72 (0.31 to 1.69) | - |
| HAART vs ART-mono | 1.25 (0.28 to 6.18) | - |
| HAART vs ART-dual | 0.58 (0.24 to 1.57) | - |
| *Common within-network between-study variance* | 0.25 (0.00 to 3.63) |  |
| ***Schmitz Model - Observational Studies*** | | |
| **Total Congenital Malformations** | | |
| ART-mono vs NoT/PLC | 0.97 (0.50 to 1.98) | - |
| ART-dual vs NoT/PLC | 1.21 (0.52 to 2.89) | - |
| ART-dual vs ART-mono | 1.28 (0.52 to 2.89) | - |
| HAART vs NoT/PLC | 1.15 (0.52 to 2.65) | - |
| HAART vs ART-mono | 1.20 (0.54 to 2.56) | - |
| HAART vs ART-dual | 0.94 (0.39 to 2.41) | - |
| *Common within-network between-study variance* | 0.49 (0.00 to 2.32) |  |
| **Major Congenital Malformations** | | |
| ART-mono vs NoT/PLC | 0.63 (0.27 to 1.45) | - |
| ART-dual vs NoT/PLC | 1.25 (0.43 to 3.32) | - |
| ART-dual vs ART-mono | 1.99 (0.81 to 4.24) | - |
| HAART vs NoT/PLC | 0.72 (0.31 to 1.69) | - |
| HAART vs ART-mono | 1.15 (0.50 to 2.57) | - |
| HAART vs ART-dual | 0.58 (0.24 to 1.57) | - |
| *Common within-network between-study variance* | 0.10 (0.00 to 1.30) |  |
| ***Schmitz Model – Randomised Control Trials*** | | |
| **Total Congenital Malformations** | | |
| ART-mono vs NoT/PLC | 1.02 (0.55 to 1.98) | - |
| ART-dual vs NoT/PLC | 1.10 (0.53 to 2.59) | - |
| ART-dual vs ART-mono | 1.09 (0.48 to 2.56) | - |
| HAART vs NoT/PLC | 1.16 (0.48 to 2.93) | - |
| HAART vs ART-mono | 1.14 (0.47 to 2.75) | - |
| HAART vs ART-dual | 1.06 (0.41 to 2.62) | - |
| *Common within-network between-study variance* | 0.23 (0.00 to 1.79) |  |
| **Major Congenital Malformations** | | |
| ART-mono vs NoT/PLC | 0.53 (0.14 to 1.67) | - |
| *Common within-network between-study variance* | 1.64 (0.44 to 5.78) |  |
| ***Sensitivity Analysis: Higher Methodological Study Quality - Comparability for Observational Studies and Randomisation for Randomised Controlled Trials*** | | |
| **Total Congenital Malformations** | | |
| ART-mono vs NoT/PLC | 0.99 (0.53 to 2.15) | 0.31 to 3.93 |
| ART-dual vs NoT/PLC | 1.50 (0.77 to 3.85) | 0.47 to 6.65 |
| ART-dual vs ART-mono | 1.53 (0.70 to 3.51) | 0.42 to 5.97 |
| HAART vs NoT/PLC | 0.72 (0.32 to 1.99) | 0.20 to 3.28 |
| HAART vs ART-mono | 0.73 (0.32 to 1.66) | 0.19 to 2.83 |
| HAART vs ART-dual | 0.48 (0.17 to 1.24) | 0.11 to 2.02 |
| *Common within-network between-study variance* | 0.13 (0.00 to 1.18) |  |
| *Design-by-treatment interaction model for inconsistency χ² (d.f., P-value, between-study variance)* | 3.27 (4, 0.51, 0.03) | |
| **Major Congenital Malformations** | | |
| ART-mono vs NoT/PLC | 0.91 (0.26 to 3.06) | 0.12 to 6.68 |
| ART-dual vs NoT/PLC | 2.11 (0.49 to 10.08) | 0.23 to 20.58 |
| ART-dual vs ART-mono | 2.28 (0.66 to 9.53) | 0.31 to 19.47 |
| HAART vs NoT/PLC | 0.76 (0.15 to 3.93) | 0.08 to 7.36 |
| HAART vs ART-mono | 0.82 (0.16 to 4.81) | 0.08 to 9.18 |
| HAART vs ART-dual | 0.36 (0.06 to 2.02) | 0.03 to 3.68 |
| *Common within-network between-study variance* | 0.24 (0.00 to 2.93) |  |
| *Design-by-treatment interaction model for inconsistency χ² (d.f., P-value, between-study variance)* | 0.17 (2, 0.92, 0.76) | |
| **Mother-to-child transmission of HIV** | | |
| [NoT]+[ART-mono] vs [NoT/PLC]+[NoT/PLC] | 0.37 (0.12 to 1.19) | 0.04 to 3.78 |
| [ART-mono]+[NoT] vs [NoT/PLC]+[NoT/PLC] | 0.39 (0.09 to 1.51) | 0.03 to 4.12 |
| [ART-mono]+[NoT] vs [NoT]+[ART-mono] | 1.08 (0.21 to 4.59) | 0.08 to 11.90 |
| [ART-mono]+[ART-mono] vs [NoT/PLC]+[NoT/PLC] | **0.26 (0.10 to 0.59)** | 0.03 to 2.09 |
| [ART-mono]+[ART-mono] vs [NoT]+[ART-mono] | 0.70 (0.21 to 2.08) | 0.07 to 6.10 |
| [ART-mono]+[ART-mono] vs [ART-mono]+[NoT] | 0.66 (0.16 to 2.76) | 0.06 to 6.97 |
| [ART-dual]+[NoT] vs [NoT/PLC]+[NoT/PLC] | **0.05 (0.00 to 0.38)** | 0.00 to 0.86 |
| [ART-dual]+[NoT] vs [NoT]+[ART-mono] | 0.12 (0.01 to 1.17) | 0.00 to 2.35 |
| [ART-dual]+[NoT] vs [ART-mono]+[NoT] | 0.12 (0.01 to 1.10) | 0.00 to 2.22 |
| [ART-dual]+[NoT] vs [ART-mono]+[ART-mono] | 0.18 (0.01 to 1.76) | 0.01 to 3.64 |
| [HAART]+[NoT] vs [NoT/PLC]+[NoT/PLC] | **0.12 (0.04 to 0.31)** | 0.01 to 1.03 |
| [HAART]+[NoT] vs [NoT]+[ART-mono] | **0.33 (0.08 to 0.98)** | 0.03 to 2.91 |
| [HAART]+[NoT] vs [ART-mono]+[NoT] | 0.31 (0.07 to 1.30) | 0.03 to 3.40 |
| [HAART]+[NoT] vs [ART-mono]+[ART-mono] | 0.47 (0.14 to 1.39) | 0.05 to 4.33 |
| [HAART]+[NoT] vs [ART-dual]+[NoT] | 2.61 (0.28 to 32.68) | 0.13 to 64.40 |
| *Common within-network between-study variance* | 0.77 (0.21 to 2.64) |  |
| *Design-by-treatment interaction model for inconsistency χ² (d.f., P-value, between-study variance)* | 27.77 (9, 0.00, 0.05) | |
| ***Sensitivity Analysis: Higher Methodological Study Quality - Adequacy of Follow-Up for Observational Studies and Incomplete Outcome Data for Randomised Controlled Trials*** | | |
| **Total Congenital Malformations** | | |
| ART-mono vs NoT/PLC | 1.11 (0.55 to 2.57) | 0.25 to 5.71 |
| ART-dual vs NoT/PLC | 1.55 (0.46 to 6.14) | 0.25 to 10.92 |
| ART-dual vs ART-mono | 1.40 (0.50 to 3.99) | 0.24 to 8.10 |
| HAART vs NoT/PLC | 1.47 (0.42 to 5.76) | 0.23 to 10.27 |
| HAART vs ART-mono | 1.32 (0.46 to 3.81) | 0.23 to 7.82 |
| HAART vs ART-dual | 0.94 (0.29 to 3.08) | 0.14 to 5.95 |
| *Common within-network between-study variance* | 0.30 (0.00 to 1.72) |  |
| *Design-by-treatment interaction model for inconsistency χ² (d.f., P-value, between-study variance)* | 7.18 (1, 0.01, 0.00) | |
| **Major Congenital Malformations** | | |
| ART-mono vs NoT/PLC | 0.79 (0.30 to 1.80) | 0.14 to 3.88 |
| ART-dual vs NoT/PLC | 1.63 (0.26 to 8.50) | 0.16 to 13.93 |
| ART-dual vs ART-mono | 2.06 (0.44 to 9.23) | 0.24 to 16.39 |
| *Common within-network between-study variance* | 0.16 (0.00 to 2.43) |  |
| *Design-by-treatment interaction model for inconsistency χ² (d.f., P-value, between-study variance)* | N/A – no closed loops | |
| **Mother-to-child transmission of HIV** | | |
| [NoT]+[ART-mono] vs [NoT/PLC]+[NoT/PLC] | 1.26 (0.16 to 10.67) | 0.08 to 21.75 |
| [ART-mono]+[NoT] vs [NoT/PLC]+[NoT/PLC] | 0.65 (0.07 to 6.25) | 0.03 to 12.59 |
| [ART-mono]+[NoT] vs [NoT]+[ART-mono] | 0.51 (0.06 to 4.43) | 0.03 to 8.26 |
| [ART-mono]+[ART-mono] vs [NoT/PLC]+[NoT/PLC] | **0.43 (0.13 to 0.96)** | 0.04 to 3.05 |
| [ART-mono]+[ART-mono] vs [NoT]+[ART-mono] | 0.33 (0.04 to 1.91) | 0.02 to 4.06 |
| [ART-mono]+[ART-mono] vs [ART-mono]+[NoT] | 0.65 (0.07 to 4.46) | 0.03 to 9.47 |
| *Common within-network between-study variance* | 0.52 (0.00 to 3.31) |  |
| *Design-by-treatment interaction model for inconsistency χ² (d.f., P-value, between-study variance)* | 2.7 (1, 0.10, 0.06) | |
| ***Sensitivity Analysis: Antenatal Care*** | | |
| **Total Congenital Malformations** | | |
| ART-mono vs NoT/PLC | 0.97 (0.41 to 2.47) | 0.15 to 6.69 |
| ART-dual vs NoT/PLC | 1.10 (0.39 to 3.03) | 0.16 to 7.89 |
| ART-dual vs ART-mono | 1.14 (0.44 to 2.64) | 0.16 to 7.21 |
| HAART vs NoT/PLC | 1.54 (0.54 to 5.00) | 0.23 to 12.42 |
| HAART vs ART-mono | 1.59 (0.67 to 4.00) | 0.24 to 11.01 |
| HAART vs ART-dual | 1.40 (0.54 to 4.30) | 0.22 to 10.77 |
| *Common within-network between-study variance* | 0.51 (0.02 to 2.17) |  |
| *Design-by-treatment interaction model for inconsistency χ² (d.f., P-value, between-study variance)* | 3.55 (5, 0.62, 0.40) | |
| **Major Congenital Malformations** | | |
| ART-mono vs NoT/PLC | 0.57 (0.19 to 1.74) | 0.08 to 4.03 |
| ART-dual vs NoT/PLC | 0.91 (0.17 to 3.57) | 0.08 to 6.61 |
| ART-dual vs ART-mono | 1.61 (0.38 to 4.48) | 0.17 to 9.01 |
| HAART vs NoT/PLC | 0.83 (0.21 to 3.56) | 0.10 to 7.11 |
| HAART vs ART-mono | 1.46 (0.44 to 5.11) | 0.20 to 11.32 |
| HAART vs ART-dual | 0.91 (0.25 to 5.36) | 0.13 to 10.53 |
| *Common within-network between-study variance* | 0.27 (0.00 to 2.67) |  |
| *Design-by-treatment interaction model for inconsistency χ² (d.f., P-value, between-study variance)* | 0.74 (2, 0.69, 0.13) | |
| **Minor Congenital Malformations** | | |
| ART-mono vs NoT/PLC | 1.86 (0.08 to 40.00) | - |
| ART-dual vs NoT/PLC | Zero events - excluded | - |
| ART-dual vs ART-mono | 8.47 (0.41 to 166.67) | - |
| HAART vs NoT/PLC | 0.41 (0.02 to 8.85) | - |
| HAART vs ART-mono | 5.10 (1.10 to 23.26) | - |
| HAART vs ART-dual | 11.11 (0.55 to 200.00) | - |
| *Common within-network between-study variance* | N/A |  |
| *Design-by-treatment interaction model for inconsistency χ² (d.f., P-value, between-study variance)* | N/A | |
| **Mother-to-child transmission of HIV** | | |
| [NoT]+[ART-mono] vs [NoT/PLC]+[NoT/PLC] | 1.22 (0.26 to 6.57) | 0.19 to 10.57 |
| [ART-mono]+[NoT] vs [NoT/PLC]+[NoT/PLC] | 0.45 (0.19 to 1.53) | 0.11 to 2.91 |
| [ART-mono]+[NoT] vs [NoT]+[ART-mono] | 0.38 (0.08 to 1.79) | 0.05 to 2.64 |
| [ART-mono]+[ART-mono] vs [NoT/PLC]+[NoT/PLC] | 0.57 (0.26 to 1.16) | 0.13 to 2.34 |
| [ART-mono]+[ART-mono] vs [NoT]+[ART-mono] | 0.46 (0.09 to 1.98) | 0.06 to 2.82 |
| [ART-mono]+[ART-mono] vs [ART-mono]+[NoT] | 1.25 (0.36 to 3.08) | 0.20 to 5.52 |
| [ART-dual]+[NoT] vs [NoT/PLC]+[NoT/PLC] | **0.04 (0.00 to 0.24)** | 0.00 to 0.35 |
| [ART-dual]+[NoT] vs [NoT]+[ART-mono] | **0.03 (0.00 to 0.30)** | 0.00 to 0.40 |
| [ART-dual]+[NoT] vs [ART-mono]+[NoT] | **0.09 (0.01 to 0.47)** | 0.01 to 0.68 |
| [ART-dual]+[NoT] vs [ART-mono]+[ART-mono] | **0.08 (0.01 to 0.48)** | 0.01 to 0.74 |
| [HAART]+[NoT] vs [NoT/PLC]+[NoT/PLC] | **0.07 (0.02 to 0.28)** | 0.01 to 0.48 |
| [HAART]+[NoT] vs [NoT]+[ART-mono] | **0.06 (0.01 to 0.37)** | 0.01 to 0.52 |
| [HAART]+[NoT] vs [ART-mono]+[NoT] | **0.16 (0.04 to 0.55)** | 0.02 to 0.90 |
| [HAART]+[NoT] vs [ART-mono]+[ART-mono] | **0.13 (0.03 to 0.57)** | 0.02 to 0.98 |
| [HAART]+[NoT] vs [ART-dual]+[NoT] | 1.73 (0.29 to 16.83) | 0.21 to 22.80 |
| *Common within-network between-study variance* | 0.14 (0.00 to 1.98) |  |
| *Design-by-treatment interaction model for inconsistency χ² (d.f., P-value, between-study variance)* | 4.48 (2, 0.11, 0.01) | |
| ***Sensitivity Analysis: LMIC*** | | |
| **Total Congenital Malformations** | | |
| ART-mono vs NoT/PLC | 1.11 (0.17 to 9.06) | 0.06 to 24.65 |
| ART-dual vs NoT/PLC | 1.03 (0.11 to 10.39) | 0.05 to 24.50 |
| ART-dual vs ART-mono | 0.94 (0.04 to 18.62) | 0.02 to 37.61 |
| HAART vs NoT/PLC | 0.88 (0.07 to 13.66) | 0.03 to 31.33 |
| HAART vs ART-mono | 0.80 (0.14 to 4.69) | 0.05 to 14.98 |
| HAART vs ART-dual | 0.86 (0.03 to 29.76) | 0.01 to 62.13 |
| *Common within-network between-study variance* | 0.68 (0.00 to 4.68) |  |
| *Design-by-treatment interaction model for inconsistency χ² (d.f., P-value, between-study variance)* | Not applicable – no closed loops | |
| **Major Congential Malformations** | | |
| ZDV vs NoT/PLC | 0.33 (0.06 to 1.64) | - |
| *Common within-network between-study variance* | N/A |  |
| *Design-by-treatment interaction model for inconsistency χ² (d.f., P-value, between-study variance)* | N/A | |
| **Mother-to-child transmission of HIV** | | |
| [ART-mono]+[ART-mono] vs [NoT/PLC]+[NoT/PLC] | 0.70 (0.39 to 1.24) | - |
| *Common within-network between-study variance* | N/A |  |
| *Design-by-treatment interaction model for inconsistency χ² (d.f., P-value, between-study variance)* | N/A | |
| ***Sensitivity Analysis: Illicit Drugs*** | | |
| **Total Congenital Malformations** | | |
| ART-mono vs NoT/PLC | 0.79 (0.29 to 2.18) | 0.18 to 3.54 |
| ART-dual vs NoT/PLC | 1.92 (0.62 to 6.04) | 0.41 to 9.56 |
| ART-dual vs ART-mono | 2.44 (0.96 to 5.95) | 0.60 to 10.43 |
| HAART vs NoT/PLC | 0.84 (0.30 to 2.35) | 0.19 to 3.73 |
| HAART vs ART-mono | 1.06 (0.39 to 2.74) | 0.23 to 4.47 |
| HAART vs ART-dual | 0.44 (0.15 to 1.24) | 0.09 to 1.91 |
| *Common within-network between-study variance* | 0.11 (0.00 to 1.44) | - |
| *Design-by-treatment interaction model for inconsistency χ² (d.f., P-value, between-study variance)* | 2.14 (2, 0.34, 0.00) | |
| **Major Congential Malformations** | | |
| ART-mono vs NoT/PLC | 0.54 (0.14 to 2.10) | 0.08 to 3.37 |
| ART-dual vs NoT/PLC | 1.45 (0.39 to 5.47) | 0.25 to 8.32 |
| ART-dual vs ART-mono | **2.61 (1.03 to 7.53)** | 0.56 to 14.50 |
| HAART vs NoT/PLC | 0.69 (0.23 to 2.15) | 0.13 to 3.75 |
| HAART vs ART-mono | 1.25 (0.43 to 4.08) | 0.24 to 6.57 |
| HAART vs ART-dual | 0.48 (0.15 to 1.46) | 0.09 to 2.26 |
| *Common within-network between-study variance* | 0.14 (0.00 to 1.83) |  |
| *Design-by-treatment interaction model for inconsistency χ² (d.f., P-value, between-study variance)* | 1.22 (1, 0.2686, 0.000) | |
| **Mother-to-child transmission of HIV** | | |
| [NoT]+[ART-mono] vs [NoT/PLC]+[NoT/PLC] | 0.61 (0.06 to 6.18) | 0.02 to 15.67 |
| [ART-mono]+[NoT] vs [NoT/PLC]+[NoT/PLC] | 0.28 (0.04 to 1.45) | 0.01 to 4.35 |
| [ART-mono]+[NoT] vs [NoT]+[ART-mono] | 0.46 (0.04 to 4.07) | 0.02 to 9.70 |
| [ART-mono]+[ART-mono] vs [NoT/PLC]+[NoT/PLC] | **0.19 (0.04 to 0.69)** | 0.01 to 2.58 |
| [ART-mono]+[ART-mono] vs [NoT]+[ART-mono] | 0.31 (0.04 to 2.26) | 0.01 to 6.05 |
| [ART-mono]+[ART-mono] vs [ART-mono]+[NoT] | 0.68 (0.11 to 4.17) | 0.04 to 12.99 |
| [ART-dual]+[NoT] vs [NoT/PLC]+[NoT/PLC] | **0.03 (0.00 to 0.27)** | 0.00 to 0.68 |
| [ART-dual]+[NoT] vs [NoT]+[ART-mono] | **0.04 (0.00 to 0.84)** | 0.00 to 1.81 |
| [ART-dual]+[NoT] vs [ART-mono]+[NoT] | 0.10 (0.01 to 1.05) | 0.00 to 2.65 |
| [ART-dual]+[NoT] vs [ART-mono]+[ART-mono] | 0.14 (0.01 to 1.86) | 0.00 to 4.01 |
| [HAART]+[NoT] vs [NoT/PLC]+[NoT/PLC] | **0.03 (0.00 to 0.14)** | 0.00 to 0.43 |
| [HAART]+[NoT] vs [NoT]+[ART-mono] | **0.05 (0.00 to 0.56)** | 0.00 to 1.24 |
| [HAART]+[NoT] vs [ART-mono]+[NoT] | **0.11 (0.01 to 0.67)** | 0.00 to 1.93 |
| [HAART]+[NoT] vs [ART-mono]+[ART-mono] | **0.16 (0.01 to 0.97)** | 0.01 to 2.89 |
| [HAART]+[NoT] vs [ART-dual]+[NoT] | 1.12 (0.07 to 17.80) | 0.03 to 36.77 |
| *Common within-network between-study variance* | 0.92 (0.07 to 4.27) |  |
| *Design-by-treatment interaction model for inconsistency χ² (d.f., P-value, between-study variance)* | 4.42 (4, 0.3521, 0.481) | |
| ***Sensitivity Analysis: TB Co-Infection*** | | |
| **Total Congenital Malformations** | | |
| ART-mono vs NoT/PLC | 1.50 (0.11 to 28.31) | 0.06 to 52.56 |
| ART-dual vs NoT/PLC | 3.61 (0.10 to 159.20) | 0.05 to 255.70 |
| ART-dual vs ART-mono | 2.35 (0.19 to 28.50) | 0.08 to 65.88 |
| *Common within-network between-study variance* | 0.45 (0.00 to 5.09) |  |
| *Design-by-treatment interaction model for inconsistency χ² (d.f., P-value, between-study variance)* | Not Applicable - No closed loops | |
| **Mother-to-child transmission of HIV** | | |
| [ART-mono]+[ART-mono] vs [NoT/PLC]+[NoT/PLC] | **0.08 (0.02 to 0.24)** | - |
| [HAART]+[NoT] vs [NoT/PLC]+[NoT/PLC] | **0.01 (0.00 to 0.12)** | - |
| [HAART]+[NoT] vs [ART-mono]+[ART-mono] | 0.08 (0.00 to 1.53) | - |
| *Common within-network between-study variance* | N/A |  |
| *Design-by-treatment interaction model for inconsistency χ² (d.f., P-value, between-study variance)* | N/A | |
| ***Sensitivity Analysis: Smoke*** | | |
| **Total Congenital Malformations** | | |
| ART-mono vs NoT/PLC | 0.96 (0.02 to 25.46) | 0.01 to 49.82 |
| ART-dual vs NoT/PLC | 1.29 (0.12 to 20.27) | 0.05 to 54.13 |
| ART-dual vs ART-mono | 1.37 (0.07 to 84.08) | 0.03 to 171.20 |
| HAART vs NoT/PLC | 1.18 (0.12 to 15.64) | 0.04 to 44.16 |
| HAART vs ART-mono | 1.25 (0.07 to 66.50) | 0.03 to 132.60 |
| HAART vs ART-dual | 0.94 (0.13 to 5.61) | 0.04 to 17.97 |
| *Common within-network between-study variance* | 0.88 (0.01 to 5.32) |  |
| *Design-by-treatment interaction model for inconsistency χ² (d.f., P-value, between-study variance)* | N/A – No independent closed loops | |
| ***Sensitivity Analysis: Alcohol*** | | |
| **Total Congenital Malformations** | | |
| ART-mono vs NoT/PLC | 1.37 (0.15 to 12.66) | - |
| ART-dual vs NoT/PLC | 2.90 (0.74 to 11.36) | - |
| ART-dual vs ART-mono | 2.12 (0.20 to 21.74) | - |
| HAART vs NoT/PLC | 0.84 (0.34 to 2.02) | - |
| HAART vs ART-mono | 0.61 (0.08 to 4.93) | - |
| HAART vs ART-dual | 0.29 (0.09 to 0.89) | - |
| *Common within-network between-study variance* | N/A |  |
| *Design-by-treatment interaction model for inconsistency χ² (d.f., P-value, between-study variance)* | N/A | |
| **Mother-to-child transmission of HIV** | | |
| [NoT]+[ART-mono] vs [NoT/PLC]+[NoT/PLC] | 0.44 (0.04 to 5.19) | 0.02 to 11.20 |
| [ART-mono]+[ART-mono] vs [NoT/PLC]+[NoT/PLC] | 0.02 (0.00 to 1.18) | 0.00 to 2.07 |
| [ART-mono]+[ART-mono] vs [NoT]+[ART-mono] | 0.04 (0.00 to 1.31) | 0.00 to 2.30 |
| [HAART]+[NoT] vs [NoT/PLC]+[NoT/PLC] | 0.30 (0.03 to 3.52) | 0.01 to 8.26 |
| [HAART]+[NoT] vs [NoT]+[ART-mono] | 0.68 (0.07 to 5.97) | 0.03 to 16.38 |
| [HAART]+[NoT] vs [ART-mono]+[ART-mono] | 15.23 (0.29 to 1297.00) | 0.15 to 2083.00 |
| Common within-network between-study variance | 0.46 (0.00 to 5.18) |  |
| *Design-by-treatment interaction model for inconsistency χ² (d.f., P-value, between-study variance)* | N/A – closed loop informed by one multi-arm study | |
| ***Sensitivity Analysis: Smoke/ Alcohol*** | | |
| **Major Congential Malformations** | | |
| ART-mono vs NoT/PLC | 1.37 (0.15 to 12.66) | - |
| ART-dual vs NoT/PLC | 2.90 (0.74 to 11.36) | - |
| ART-dual vs ART-mono | 2.12 (0.20 to 21.74) | - |
| HAART vs NoT/PLC | 0.84 (0.34 to 2.02) | - |
| HAART vs ART-mono | 0.61 (0.08 to 4.93) | - |
| HAART vs ART-dual | 0.29 (0.09 to 0.89) | - |
| *Common within-network between-study variance* | N/A |  |
| *Design-by-treatment interaction model for inconsistency χ² (d.f., P-value, between-study variance)* | N/A | |
| ***Sensitivity Analysis: CD4+ count (<200)*** | | |
| **Total Congenital Malformations** | | |
| ART-mono vs NoT/PLC | 1.08 (0.53 to 2.46) | 0.24 to 5.56 |
| ART-dual vs NoT/PLC | 0.93 (0.24 to 5.08) | 0.16 to 9.10 |
| ART-dual vs ART-mono | 0.86 (0.22 to 4.37) | 0.14 to 8.01 |
| HAART vs NoT/PLC | 0.93 (0.35 to 3.10) | 0.19 to 6.45 |
| HAART vs ART-mono | 0.86 (0.35 to 2.48) | 0.17 to 5.08 |
| HAART vs ART-dual | 1.00 (0.27 to 3.18) | 0.14 to 5.55 |
| *Common within-network between-study variance* | 0.26 (0.00 to 1.77) |  |
| *Design-by-treatment interaction model for inconsistency χ² (d.f., P-value, between-study variance)* | 7.67 (3, 0.05, 0.00) | |
| **Major Congential Malformations** | | |
| ART-mono vs NoT/PLC | 0.79 (0.52 to 1.21) | - |
| *Common within-network between-study variance* | 0.00 |  |
| *Design-by-treatment interaction model for inconsistency χ² (d.f., P-value, between-study variance)* | N/A | |
| **Mother-to-child transmission of HIV** | | |
| [NoT]+[ART-mono] vs [NoT/PLC]+[NoT/PLC] | 0.40 (0.10 to 2.18) | 0.04 to 5.87 |
| [ART-mono]+[NoT] vs [NoT/PLC]+[NoT/PLC] | 0.41 (0.09 to 1.86) | 0.03 to 4.92 |
| [ART-mono]+[NoT] vs [NoT]+[ART-mono] | 1.02 (0.16 to 4.75) | 0.06 to 11.45 |
| [ART-mono]+[ART-mono] vs [NoT/PLC]+[NoT/PLC] | **0.23 (0.05 to 0.74)** | 0.02 to 2.25 |
| [ART-mono]+[ART-mono] vs [NoT]+[ART-mono] | 0.56 (0.09 to 2.29) | 0.03 to 5.35 |
| [ART-mono]+[ART-mono] vs [ART-mono]+[NoT] | 0.57 (0.10 to 2.47) | 0.04 to 6.51 |
| [ART-dual]+[NoT] vs [NoT/PLC]+[NoT/PLC] | **0.05 (0.00 to 0.45)** | 0.00 to 0.99 |
| [ART-dual]+[NoT] vs [NoT]+[ART-mono] | 0.12 (0.01 to 1.25) | 0.00 to 2.52 |
| [ART-dual]+[NoT] vs [ART-mono]+[NoT] | 0.12 (0.01 to 1.15) | 0.01 to 2.63 |
| [ART-dual]+[NoT] vs [ART-mono]+[ART-mono] | 0.21 (0.02 to 2.78) | 0.01 to 5.78 |
| [HAART]+[NoT] vs [NoT/PLC]+[NoT/PLC] | **0.14 (0.03 to 0.65)** | 0.01 to 1.74 |
| [HAART]+[NoT] vs [NoT]+[ART-mono] | 0.36 (0.06 to 1.54) | 0.02 to 4.15 |
| [HAART]+[NoT] vs [ART-mono]+[NoT] | 0.35 (0.07 to 1.83) | 0.03 to 4.56 |
| [HAART]+[NoT] vs [ART-mono]+[ART-mono] | 0.62 (0.13 to 4.19) | 0.05 to 10.68 |
| [HAART]+[NoT] vs [ART-dual]+[NoT] | 2.93 (0.30 to 39.81) | 0.14 to 75.39 |
| *Common within-network between-study variance* | 0.68 (0.06 to 3.24) |  |
| *Design-by-treatment interaction model for inconsistency χ² (d.f., P-value, between-study variance)* | 5.15 (4, 0.27, 0.44) | |
| **Treatment Abbreviations:** ART, Antiretroviral Therapy; HAART, Highly Active Anti-Retroviral; ABC, Abacavir; ddI, Didanosine; CM, Congenital Malformations; IND, Indinavir; 3TC, Lamivudine; LMIC, Low and Middle-Income Countries; LOP, Lopinavir; MTCT, Mother to Child Transmission; N/A, Not Applicable; NVP, Nevirapine; NLF Nelfinavir; NoT, No Treatment; Plc, Placebo; SAQ, Saquinavir; d4T Stavudine; EFV, Sustiva; RIT, Ritonavir; ZDV, Zidovudine. | | |
| **Note:** Statistically significant results are **bolded**. | | |
